# Synthesis of chiral lactams by asymmetric nitrogen insertion

**DOI:** 10.1039/d5sc08417b

**Published:** 2025-11-10

**Authors:** Jasmin Hammes, Clara Mañas, Abhilash Pedada, Marlene Arnold, Johannes M. Wahl

**Affiliations:** a Department Chemie, Johannes Gutenberg-Universität Duesbergweg 10-14 55128 Mainz Germany wahl@uni-mainz.de

## Abstract

Asymmetric nitrogen insertion into prochiral and meso cycloalkanones is achieved using diphenylphosphinyl hydroxylamine (DPPH) as the nitrogen source. A combination of Brønsted acid catalyst and Lewis acid promoter enables high yields and selectivity in the syntheses of a range of 5- to 7-membered lactams (19 examples, up to 97 : 3 er). Mechanistically, the sequence follows a Beckmann pathway involving an asymmetric condensation followed by a stereospecific rearrangement. The utility of the method is showcased by the syntheses of the drugs phenotropil, rolipram, pregabalin, and baclofen.

## Abstract 2

An asymmetric Beckmann sequence enables the synthesis of enantioenriched 5–7-membered lactams from prochiral and meso cycloalkanones. The selectivity originates from an enantioselective condensation followed by a stereospecific rearrangement.

## Graphical abstract

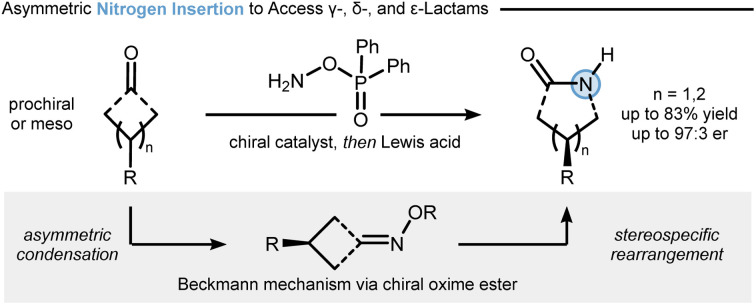

## Introduction

**Fig. 1:**
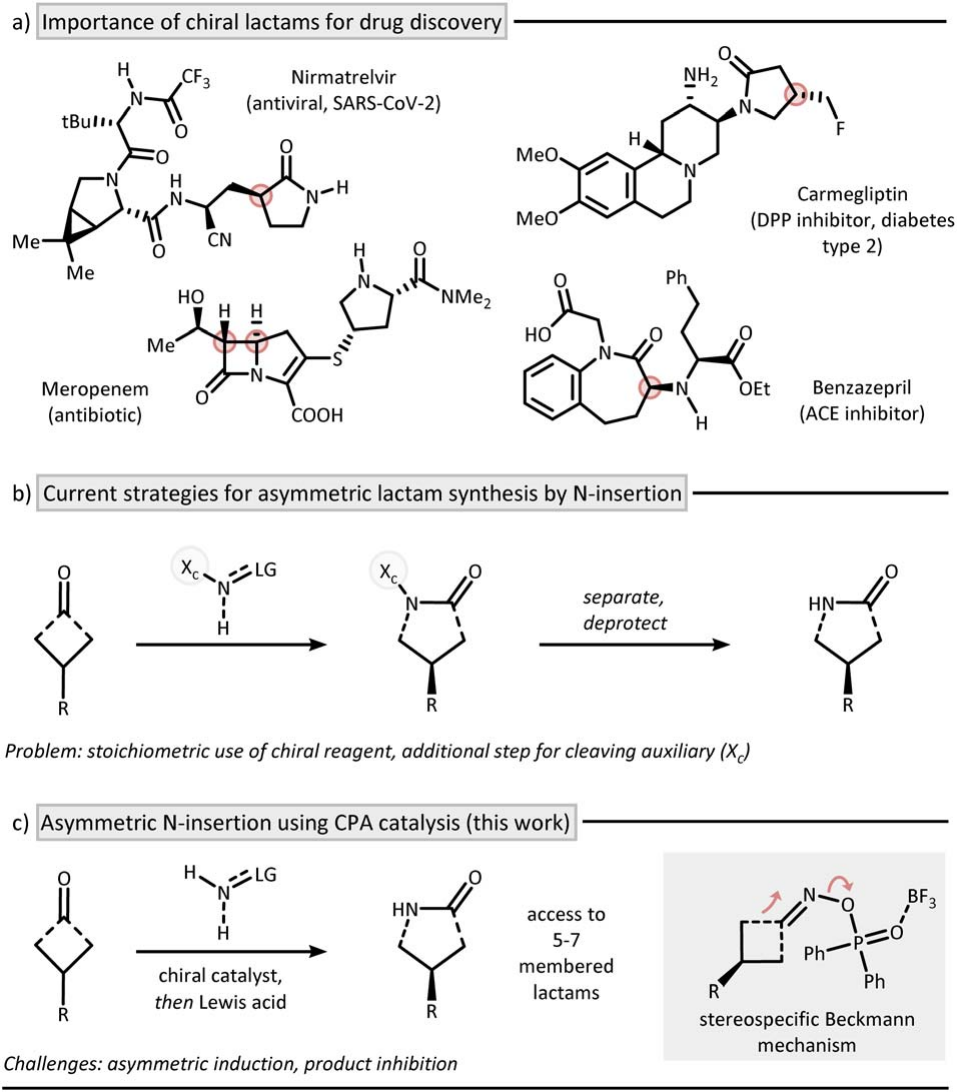
Accessing chiral lactams by asymmetric nitrogen insertion. (a) Chiral lactams as key scaffold in drug discovery. (b) Asymmetric synthesis by using chiral reagents. (c) Asymmetric access using catalysis for stereoinduction.

The direct insertion of a nitrogen atom into a cyclic ketone represents a powerful synthetic strategy to forge lactams as an important class of nitrogen heterocycles.^1,2^ A prominent example is the large-scale production of ε-caprolactam from cyclohexanone, which serves as a key precursor in Nylon manufacturing.^3^ Traditionally, nitrogen atom insertion is achieved *via* the venerable Beckmann^4^ and Schmidt^5^ reactions. The relatively harsh reaction conditions of these methods have hindered the development of enantioselective protocols, yet the prevalence of chiral lactams in modern drugs makes asymmetric variants particularly compelling (Fig. 1a). Most existing asymmetric protocols thus rely on chiral reagents for induction (Fig. 1b).^6–9^ It is striking that, while the related catalytic asymmetric oxygen insertion *via* Baeyer–Villiger rearrangement has been successfully implemented both enzymatically^10,11^ and by using small molecule catalysts,^12^ no broadly applicable approach exists for nitrogen insertion.^13^ This absence of catalytic asymmetric strategies may stem from the fundamental mechanistic differences between the Beckmann and Baeyer–Villiger rearrangements, along with the difficulties in establishing catalysis for nitrogen insertion.^14^ One major obstacle is product inhibition, an inherent issue in any ketone-to-amide conversion.^15,16^ In addition, no enzymatic pathways for nitrogen insertion are known, eliminating Nature as a potential blueprint for catalyst design.

Inspiration for our work can be found in the pioneering reports on asymmetric oxime formation by Antilla and co-workers,^17^ as well as on the elegant multistep chirality transfer approaches from oxime ethers to lactams recently described by the groups of Tan^18^ and Shi.^19^ However, a general, two-step Beckmann process has to the best of our knowledge not been disclosed and represents a long-time goal in nitrogen insertion chemistry providing access to chiral 3D-nitrogen heterocycles. In this work, we address this challenge by presenting a mild and enantioselective nitrogen insertion method for synthesizing 5- to 7-membered lactams, which operates within the Beckmann mechanistic framework (Fig. 1c).

## Results and discussion

Our strategy for synthesizing chiral lactams relies on the use of hydroxylamine-derived reagents.^20,21^ We recently discovered that *O*-phosphinyl hydroxylamines undergo efficient nitrogen insertion, offering a practical approach due to their commercial availability.^22^ Nitrogen insertion at cyclobutanones with hydroxylamine derivatives has been shown to proceed *via* an aza-Baeyer–Villiger pathway.^22–24^ Similar findings were also reported for cyclopropanones by Lindsay and coworkers.^25^ Given this mechanistic peculiarity, along with the synthetic importance of γ-lactams in drug discovery,^26^ we deemed prochiral cyclobutanones as an ideal starting point for developing a catalytic asymmetric nitrogen insertion protocol.

Based on the privileged role of chiral phosphoric acids (CPAs) in organocatalyzed condensation^27^ and insertion reactions,^28–31^ we initiated a systematic evaluation of the reaction conditions using prochiral phenylcyclobutanone 1a as the model substrate (see SI for full details). The optimization identified diphenylphosphinyl hydroxylamine (3) as the optimal nitrogen source, *R*-TCYP (4) as the most effective CPA catalyst, and boron trifluoride as essential promoter to forge γ-lactam 2a in 82% yield and an enantiomeric ratio (er) of 87 : 13 (Table 1, entry 1). Several key insights emerged from the optimization process. Importantly, the combination of Brønsted acid and Lewis acid was crucial for a successful outcome. Low yields were obtained in the absence of boron trifluoride (entry 2), while alternative promoters such as *para*-toluenesulfonic acid (*p*TsOH) or heat afforded the lactam with low selectivity (entries 3 & 4). High enantioselectivity was also dependent on the sequential addition of Brønsted and Lewis acids as can be extracted from the simultaneous addition experiment (entry 5).

**Table 1:** Reaction optimization using DPPH (3) as the nitrogen source

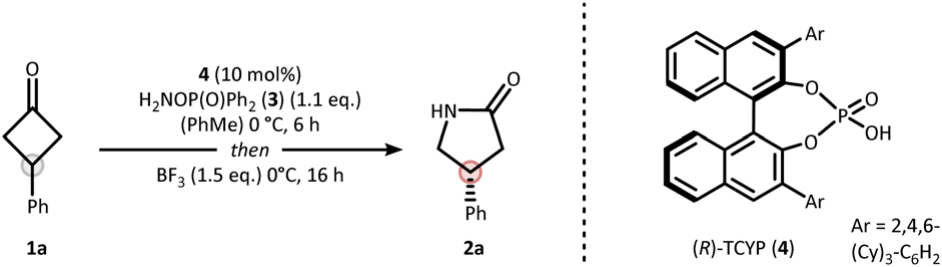			
Entry	Changes from standard condition^a^	Yield^b^	er^c^
1	—	82%	87 : 13
2	No BF_3_ was added after 6 h	4%	82 : 18
3	*p*TsOH instead of BF_3_	41%	54 : 46
4	Heated to 80 °C instead of BF_3_ addition	14%	52 : 48
5	BF_3_ added from the beginning	94%	59 : 41
6	0.5 equivalents (eq.) BF_3_ used	<5%	—
7	Wet PhMe was used	80%	85 : 15
8	4 Å MS added from the beginning	56%	89 : 11

a Reactions were run on a 0.1 mmol scale using 2.0 mL of solvent (0.05 M).

b Determined by ^1^H NMR using CH_2_Br_2_ as an internal standard.

c Determined by HPLC analysis using a chiral column.

Moreover, BF_3_ must be used in stoichiometric amounts to effectively promote the insertion; catalytic quantities proved insufficient (entry 6). Interestingly, the reaction showed notable tolerance to residual water (entry 7). In contrast, strictly anhydrous conditions using 4 Å molecular sieves (MS) led to a reduced yield (entry 8), providing an initial hint about the underlying mechanism (*vide infra*).

**Fig. 2:**
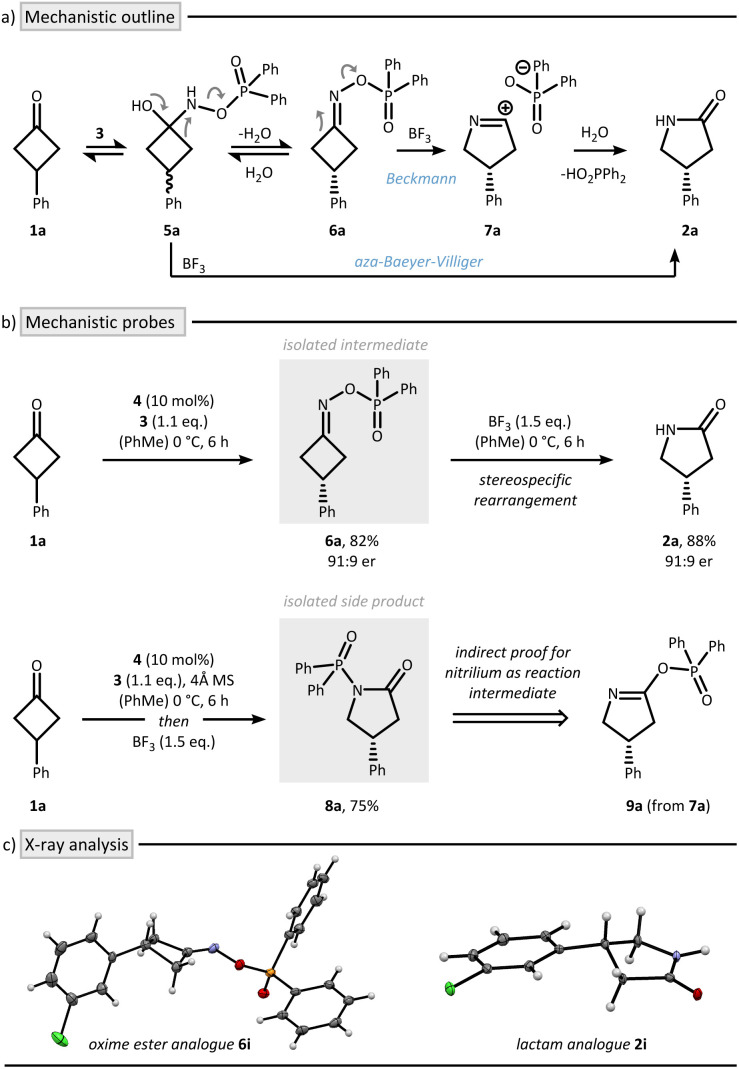
Mechanistic analysis of the reaction mechanism. (a) Reasonable mechanistic pathways. (b) Isolated intermediates and side products supporting a Beckmann mechanism for the asymmetric nitrogen insertion. (c) X-ray analysis of the isolated compounds.

Two mechanistic scenarios are plausible for the nitrogen insertion sequence: (i) asymmetric condensation followed by stereospecific rearrangement (Beckmann pathway), or (ii) enantioselective insertion prior to condensation from prochiral hemiaminal 5a (aza-Baeyer–Villiger pathway) (Fig. 2a). To probe these possibilities, we conducted a series of carefully designed control experiments (Fig. 2b). Isolation of oxime ester intermediate 6a was possible in 82% yield and 91 : 9 er when conducting the reaction in the absence of BF_3_. The role of the CPA within this asymmetric condensation process was recently elucidated by density functional theory (DFT) calculations, suggesting the formation of the depicted absolute configuration of 6a.^30^ Upon treatment with BF_3_ a stereospecific rearrangement to lactam 2a was initiated. X-ray crystallographic analysis of the respective chloro-derivatives 6i and 2i enabled assignment of the absolute configuration and confirmed their structural identity (Fig. 2c).

These observations support a Beckmann pathway, in which the CPA catalyzes the initial asymmetric condensation,^31,32^ while BF_3_ promotes the subsequent stereospecific rearrangement, explaining the importance of sequential addition which circumvents the problem of product inhibition of the CPA. Under rigorously anhydrous conditions, we were successful in isolating side product 8a. This species likely arises from nitrilium ion capture by the phosphinic acid (7a → 9a), followed by Chapman-type rearrangement^33,34^ a process only consistent with a Beckmann mechanism.^35,36^

**Fig. 3:**
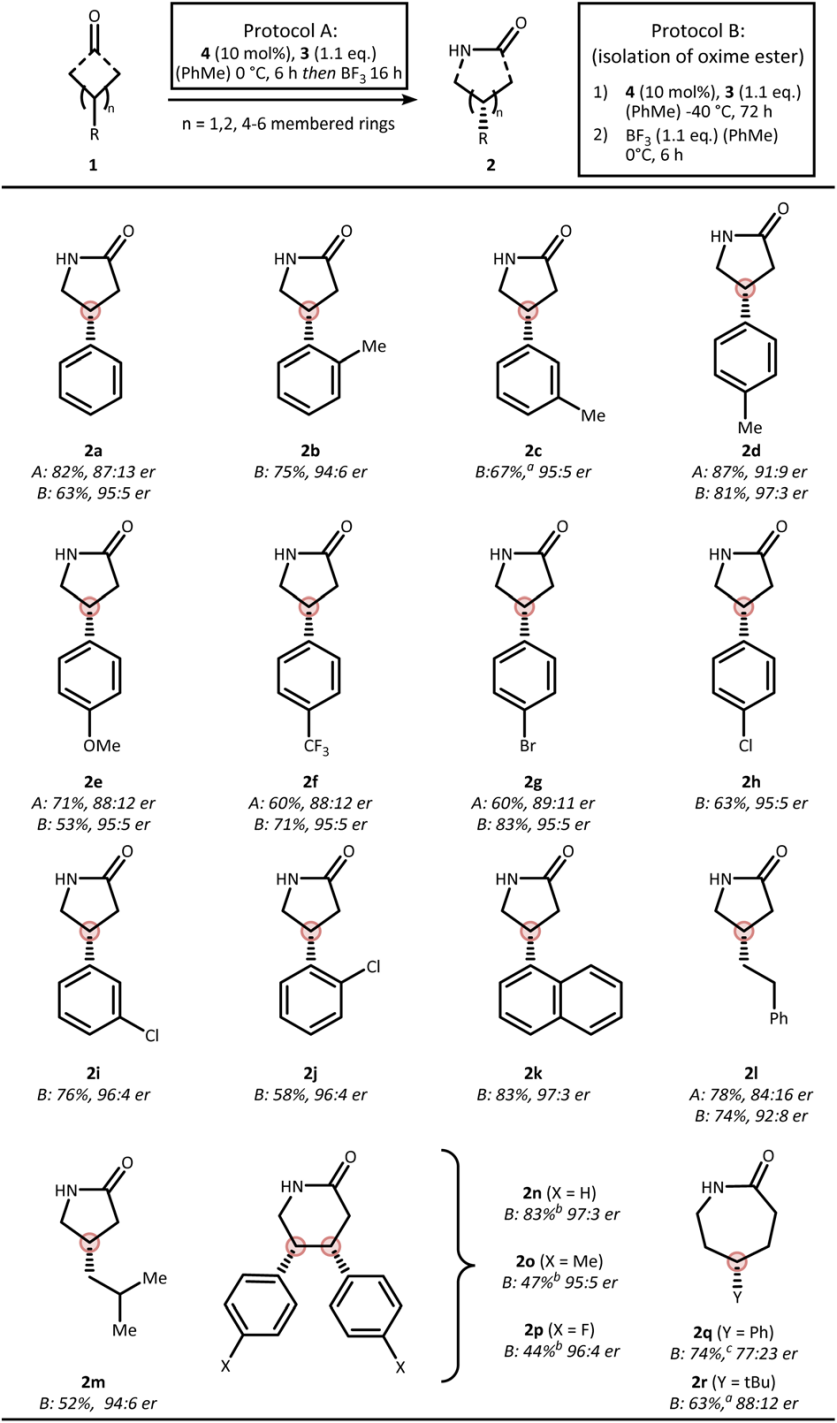
Scope of the asymmetric nitrogen insertion using meso or prochiral cycloalkanones. All reactions were run on a 0.2 mmol scale in toluene as the solvent (0.05 M). Enantiomeric ratio was determined by HPLC using a chiral column. ^*a*^ The condensation was run for 24 hours at –20 °C. ^*b*^ The rearrangement was conducted at rt using 2.5 eq. of BF_3_. ^*c*^ The rearrangement was conducted using Et_2_AlCl (1.0 eq.) as the Lewis acid.

During the mechanistic analysis, we realized that performing the reaction in a two-step fashion led to a small increase in enantioselectivity for lactam 2a, improving from 87 : 13 er to 91 : 9 er (*cf.*Table 1 and Fig. 2b). We attribute this enhancement to the avoidance of undesired racemization of the chiral oxime ester intermediate, likely caused by the formation of a highly acidic environment from residual Brønsted acid and BF_3_.^37^ We further found that lowering the temperature to −40 °C during the initial enantio-determining condensation further enhances the enantioselectivity to 95 : 5 er. Based on these observations, we decided to explore the scope using both a one-pot protocol A (as described in the optimization) as well as a two-step protocol B, which involves low temperature condensation and removal of the residual CPA after the initial step by a short silica gel purification (Fig. 3).

During our scope evaluation, we found that steric and electronic perturbations on the aromatic ring were well tolerated using both Beckmann protocols, affording the corresponding γ-lactams 2a–f in >87 : 13 er using protocol A and consistently >94 : 6 er when using protocol B. Halogenation at various positions of the aryl ring – offering synthetic handles for further derivatization – proceeded smoothly to furnish lactams 2g–j with high selectivity observed in all cases (protocol A > 89 : 11 er, protocol B > 95 : 5 er). A naphthyl-substituted substrate was also compatible, delivering γ-lactam 2k in 83% yield and 97 : 3 er using the two-step protocol. Alkyl-substituted cyclobutanones were tolerated as well, affording 2l and 2m in good yields, albeit with slightly lower enantioselectivity. Notably, the nitrogen insertion was not restricted to cyclobutanones : meso-cyclopentanones also participated in the transformation when using protocol B, providing δ-lactam 2n, 2o, and 2p in good yield and excellent enantioselectivity (>95 : 5 er). Extension to six-membered prochiral cyclohexanones was also feasible using either Et_2_AlCl or BF_3_ as Lewis acids. Under these conditions, ε-lactam 2q and 2r were obtained in 74% and 63% yield, albeit with a reduced selectivity of 77 : 23 er and 88 : 12 er. A key advantage of this protocol lies in its straightforward applicability to less strained cycloalkanones as evidenced by the successful synthesis of lactams 2n–r.

**Fig. 4:**
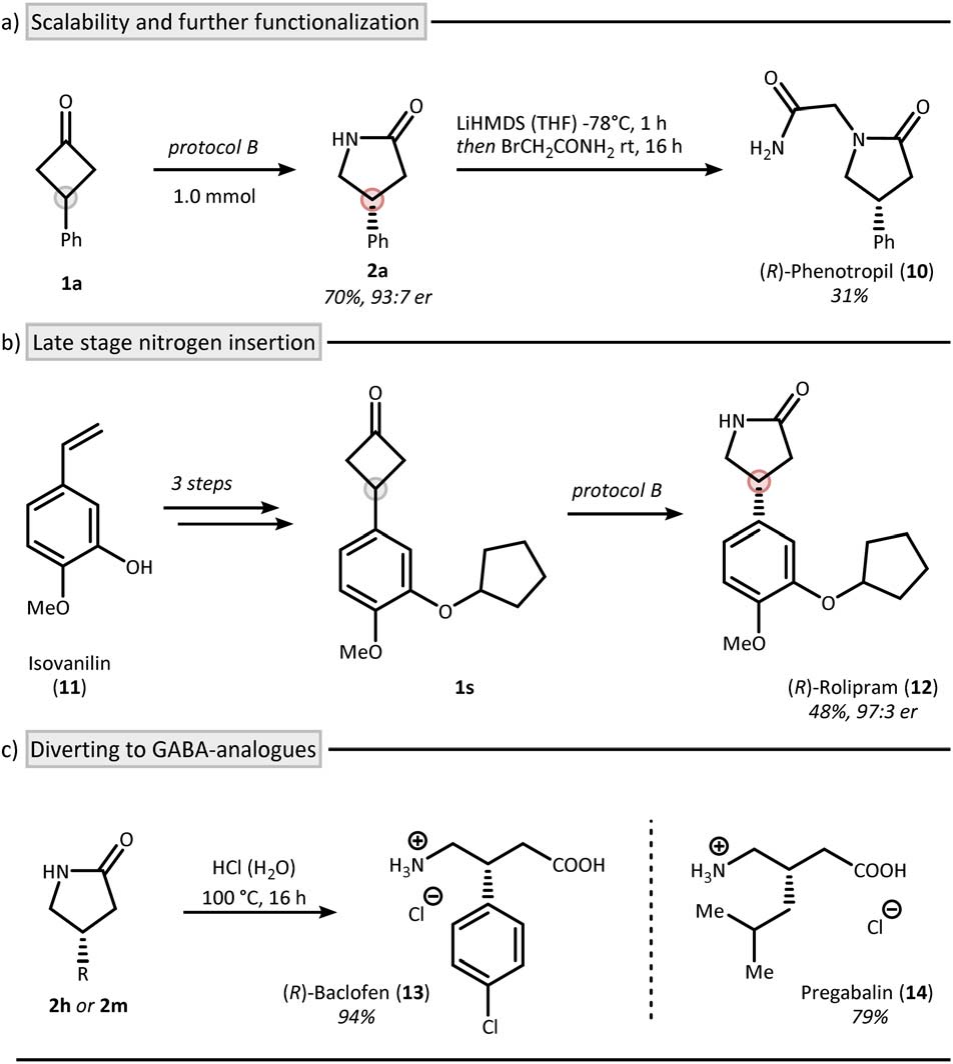
Application of the method through the syntheses of bioactive γ-lactams. Synthesis of phenotropil (a) and rolipram (b), as well as the GABA-derivatives baclofen and pregabalin (c). Protocol B: see Fig. 3.

The reaction was also found to be scalable as shown by the synthesis of lactam 2a on 1 mmol scale using protocol B (Fig. 4a). We were able to recover 91% of CPA 4 during this process.^38^ Interestingly, lactam 2a can be transferred to *R*-phenotropil (10) in one additional step.^39^ To further highlight the synthetic utility of an asymmetric Beckmann reaction, we applied protocol B to the synthesis of the phosphordiesterase-4 (PDE-4) inhibitor rolipram (12) starting from isovanillin (11) (Fig. 4b).^40–42^ The nitrogen insertion was achieved at the final stage of the synthesis in 48% yield and 97 : 3 er highlighting the applicability of the method for late-stage functionalization (1s → 12). Furthermore, baclofen (13, trade name: Lioresal^®^), a clinically used drug for muscle spasticity, can be accessed in its enantiopure form from lactam 2h (Fig. 4c).^43^ This is particularly noteworthy given that baclofen is typically administered as a racemate, despite the well-established differences in biological activity between its enantiomers.^44^ Similarly, pregabalin (14, trade name: Lyrica®), another γ-aminobutyric acid (GABA) derivative used primarily as an anticonvulsant, was obtained *via* hydrolysis of lactam 2m in 79% yield.^45–47^

## Conclusions

In summary, this study introduces an asymmetric Beckmann strategy for nitrogen insertion into cyclic ketones *via* a sequence of enantioselective condensation and stereospecific rearrangement. Key to its success is the combination of DPPH as the nitrogen source, along with a CPA organocatalyst and BF_3_ as a stereospecific Lewis acid promoter. The sequence can be run in a slightly less selective one-pot protocol as well as a in a two-step process based on the good stability of the oxime ester intermediates. Thus, the developed protocol provides robust access to enantioenriched γ-, δ-, and ε-lactams. The utility of the method is highlighted through the enantioselective synthesis of four pharmaceutically relevant compounds. In light of the fact that 81% of all small-molecule drugs feature nitrogen-containing heterocycles, this work is expected to stimulate further advances in the development of mild and selective nitrogen insertion methods.

## Author contributions

M. A. and J. M. W. conceived the project. J. H., C. M., A. P., and M. A. carried out the method development and experimental investigations. J. M. W. supervised the project and secured funding. All authors contributed to data analysis, participated in writing the manuscript, and approved the final version.

## Conflicts of interest

There are no conflicts to declare.

## Supplementary Material

SC-017-D5SC08417B-s001

SC-017-D5SC08417B-s002

## Data Availability

Experimental data including detailed procedures, characterization of new compounds as well as NMR and HPLC spectra is accessible in the SI. CCDC 2465049 (6i) and 2465050 (2i) contain the supplementary crystallographic data for this paper.^48*a*,*b*^ Supplementary information: experimental data including detailed procedures, characterisation of new compounds as well as NMR and HPLC spectra is accessible in the SI. See DOI: https://doi.org/10.1039/d5sc08417b.
