# Supplementary material for: Synthesis of chiral lactams by asymmetric nitrogen insertion
Source: Chem Sci. 2025 Nov 10;17(1):597–601. doi: 10.1039/d5sc08417b (PMC12598879; doi:10.1039/d5sc08417b)

Supplementary Information (SI) for Chemical Science.
This journal is © The Royal Society of Chemistry 2025

Supplementary Information

Synthesis of chiral Lactams by Asymmetric Nitrogen Insertion

Jasmin Hammes+,[a] Clara Mañas+,[a] Abhilash Pedada,[a] Marlene Arnold,[a] and Johannes M. Wahl*[a]

[a] Department Chemie, Johannes Gutenberg-Universität, Duesbergweg 10-14, 55128 Mainz, Germany

* E-mail: wahl@uni-mainz.de

+ These authors contributed equally to this work

Contents

1. General 1
2. Synthesis of starting materials 3
3. Optimization for enantioselective *N*-Insertion 10
4. Mechanistic investigations 14
5. Substrate scope of lactams 16
6. Applications 34
7. Crystallographic data 36
8. References 38
9. NMR Spectra and HPLC Traces 39

1. General

Reaction Set-up: Chemicals were purchased from *Alfa Aesar*, *Acros Organics*, *Sigma Aldrich*, *BLDpharm*, *FluoroChem*, *Carbolution* or *ABCR* and (unless otherwise stated) used as received. All reactions involving air or moisture sensitive reagents were carried out in oven- (125 °C) and flame-dried glassware under nitrogen atmosphere using standard *Schlenk* techniques. Dry solvents were collected from an *MBraun MB SPS-800* (toluene: MB-KOL-A and MB-KOL-C). A positive argon pressure was used to pass the solvents through the columns. Unless otherwise noted, all work-up and purification procedures were carried out with pre-distilled technical grade solvents. Purification was performed with standard column chromatography techniques using *Geduran*® Si 60 silica gel (0.063-0.200 mm, *Merck*), on an automated flash chromatography system *Biotage Isolera One* utilizing *Biotage Sfär Silica D-Duo* 60 µm columns (5 g, 25 g, 100 g). Glass silica gel plates 60 F254 (*Merck*) were used for analytic thin layer chromatography applying either UV light (254/366 nm), $KMnO_4$ (1.5 g $KMnO_4$, 5 g $NaHCO_3$ and 5 mL NaOH 10% in 200 mL H_2O) for detection.

Analytical methods: Melting points (**M.P.**) were measured on a *Büchi* B-540 melting-point apparatus and are reported uncorrected. Infrared (**IR**) spectra were obtained on a Tensor 27 spectrometer (*Bruker*) using a diamond ATR unit and are reported in wavenumbers (cm-1). Bands are characterized as broad (br), strong (s), medium (m), and weak (w). Nuclear magnetic resonance (**NMR**) spectra were recorded by the analytical department of the Department Chemie at Johannes Gutenberg-Universität Mainz. The following spectrometers were used: Avance III HD 300 (*Bruker*), Avance II 400 (*Bruker*), Avance III HD 400 (*Bruker*), and Avance III 600 equipped with a cryo-probe head (*Bruker*). Spectra were recorded at 26 °C (unless otherwise noted). Chemical shifts are reported in ppm with the solvent resonance as the internal standard (^{1}H NMR $CHCl_3$: δ = 7.26 ppm, $(CHD_2)(CD_3)SO$: δ = 2.50 ppm, CHD_2OD: δ = 3.31 ppm; ^{13}C NMR $CDCl_3$: δ = 77.16 ppm, $(CD_3)_2SO$: δ = 39.52 ppm, CD_3OD: δ = 49.0 ppm). The data is reported as follows: chemical shift, multiplicity (s = singlet, d = doublet, t = triplet, q = quartet, p = pentet, br = broad, m = multiplet or combinations of these), coupling constants (Hz) and integration. Apparent multiplicity, which occurs as a result of accidental equality of coupling constants to magnetically non-equivalent protons, is marked as *app.* High Resolution Mass Spectrometry (**HRMS**) was performed by the analytical department of the Department Chemie at Johannes Gutenberg-Universität Mainz. Spectra were recorded on a *Thermo-Fisher Scientific* DFS (GC-MS, ionization via electron ionization (EI) or chemical ionization (CI)) or on an *Agilent* 6545 Q-ToF (LC-MS, ionization via electron spray ionization (ESI), atmospheric-pressure chemical ionization (APCI)). Signals are reported as mass to charge ratio *m/z.*

Optical rotations were measured on a Perkin-Elmer 241 polarimeter at 589 nm wavelength (Na D-line) using a standard 10 cm cell (1 mL). Specific rotations, [α]DT, are reported in °·mL/(g·dm) at the specific temperature. Concentrations (c) are given in grams per 100 mL of the specific solvent. Analytical high-performance liquid chromatography (**HPLC**) measurements were performed on the *Agilent Technologies* 1260 Infinity II HPLC-System with

a binary pump, high performance degassing unit, automized liquid sampler, thermostatic column oven and diode array detector or on a *Shimadzu* Nexera LC-40D lite HPLC-System with a quaternary pump, high performance degassing unit, automized liquid sampler, thermostatic column oven and diode array detector. Separation was performed using Lux® Cellulose-1 (4.6 x 250 nm x 5 µm, Phenomenex Ltd.), Lux® Amylose-1 (4.6 x 250 nm x 5 µm, Phenomenex Ltd.), Lux® i-Amylose-3 (4.6 x 250 nm x 5 µm, Phenomenex Ltd.), or Reprosil Chiral-AMS (4.6 x 250 nm x 5 µm, Dr Maisch GmBH.).

2. Synthesis of starting materials

Cyclobutanones **1a** to **1h** and **1l** to **1m** as well as **1s** were prepared according to the methods reported in the literature. [2,3] Cyclopentanone **1n** was prepared according to the method reported in the literature.[4] Cyclohexanones **1q** was purchased from *BLDPharm* and used as received. Cyclohexanones **1r** was purchased from *BLDPharm* as a racemate and recrystallized according to literature to afford the meso compound.[5]

General procedure A (GP-A) for the [2+2] cycloaddition of ketene iminium salt:

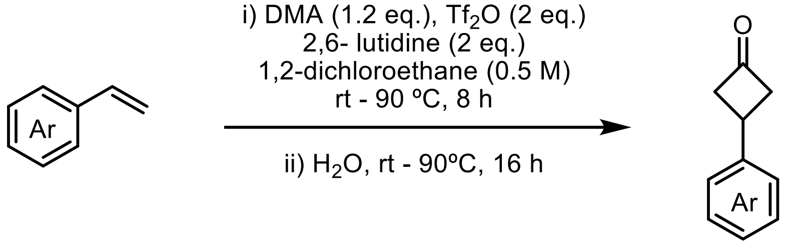

A Schlenk tube was charged with dimethylacetamide (1.20 eq.) in 1,2-dichloroethane (0.5 M). The reaction solution was cooled to room temperature with a water bath. Tf_2O (2.00 eq.) was added dropwise, and the reaction mixture was stirred at room temperature for 10 min. A solution of the corresponding alkene (1.00 eq.) and 2,6-lutidine (2.00 eq.) in 1,2-dichloroethane (2.0 M) was added dropwise to the reaction mixture. The solution was stirred at 90 °C for 8 h. After cooling to room temperature water (20 mL) was added. The reaction mixture was stirred at 90 °C for 16 h. The mixture was allowed to cool to room temperature and the layers were separated. The aqueous layer was extracted with CH_2Cl_2 (4 × 50 mL). The combined organic layers were dried over $MgSO_4$, filtered and the solvent was removed under reduced pressure. The product was separated via flash column chromatography with the conditions given in the corresponding entry.

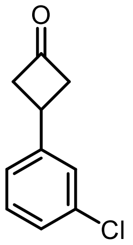

3-(3-Chlorophenyl)cyclobutan-1-one [1i]

Following **GP-A** using 3-chlorostyrene (1.00 g, 7.22 mmol, 1.00 eq.) the product (0.38 g, 2.10 mmol, 29%) was obtained via flash column chromatography (SiO_2, hexane:EtOAc, 90:10, stained with $KMnO_4$) as a colorless oil.

IR (neat): ṽ = 1782 (s), 1597 (m), 1573 (w), 1480 (w), 1433 (w), 1379 (w), 1104 (m), 1080 (m), 997 (w), 874 (w), 783 (m), 697 (m), 439 (w). **^{1}H NMR (400 MHz, CDCl$_3$)** δ (ppm) = 7.34 – 7.27 (m, 2H), 7.25 (dt, *J* = 8.2, 1.5 Hz, 1H), 7.20 (dt, *J* = 7.2, 1.3 Hz, 1H), 3.68 (p, *J* = 7.9 Hz, 1H), 3.59 – 3.44 (m, 2H), 3.33 – 3.18 (m, 2H). **^{13}C NMR (101 MHz, CDCl$_3$)** δ (ppm) = 205.9, 145.7, 134.7,

130.1, 127.0, 124.8, 54.72, 28.3. **HRMS (APCI):** calculated for $C_{10}H_8ClO$ $[M-H]^-$: 179.0269, found: 179.0277.

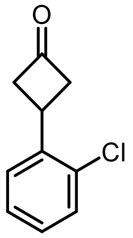

3-(2-Chlorophenyl)cyclobutan-1-one [1j]

Following **GP-A** using 2-chlorostyrene (1.39 g, 10.0 mmol, 1.00 eq.) the product (1.02 g, 5.66 mmol, 57%) was obtained via flash column chromatography (SiO_2, hexane:EtOAc, 90:10, stained with $KMnO_4$) as a colorless oil.

IR (neat): ṽ = 1783 (s), 1476 (w), 1441 (w), 1379 (w), 1103 (w), 1040 (m), 752 (s), 694 (w), 650 (w), 447 (m). **^{1}H NMR (400 MHz, $CDCl_3$)** δ (ppm) = 7.41 (dd, *J* = 7.8, 1.4 Hz, 1H), 7.36 (dd, *J* = 7.7, 1.8 Hz, 1H), 7.29 (td, *J* = 7.5, 1.5 Hz, 1H), 7.22 (td, *J* = 7.6, 1.8 Hz, 1H), 3.96 (p, *J* = 8.4 Hz, 1H), 3.59 – 3.45 (m, 2H), 3.32 – 3.19 (m, 2H). **^{13}C NMR (101 MHz, $CDCl_3$)** δ (ppm) = 206.4, 140.1, 134.5, 130.0, 128.2, 127.2, 126.7, 53.1, 26.8. **HRMS (ESI):** calculated for $C_{10}H_{10}ClO$ $[M+H]^+$: 181.0415, found: 181.0407.

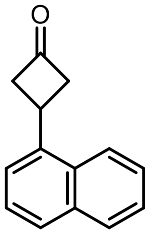

3-(Naphthalen-1-yl)cyclobutan-1-one [1k]

Following **GP-A** using 1-vinylnaphthalene (0.90 g, 5.80 mmol, 1.00 eq.) the product (683 mg, 3.48 mmol, 60%) was obtained via flash column chromatography (SiO_2, hexane:EtOAc, 90:10, stained with $KMnO_4$) as a colorless oil.

^{1}H NMR (400 MHz, $CDCl_3$) δ (ppm) = 7.98 – 7.89 (m, 2H), 7.84 – 7.77 (m, 1H), 7.62 – 7.41 (m, 4H), 4.26 (p, *J* = 8.3 Hz, 1H), 3.69 – 3.56 (m, 2H), 3.46 – 3.34 (m, 2H). **^{13}C NMR (101 MHz, $CDCl_3$)** δ (ppm) = 206.6, 138.2, 134.1, 131.7, 129.1, 127.7, 126.4, 126.0, 125.4, 123.8, 122.4, 53.1, 26.1. The spectroscopic data were in agreement to those previously reported.[6]

General procedure B (GP-B) for the synthesis of cyclopentanones **1o** and **1p**:

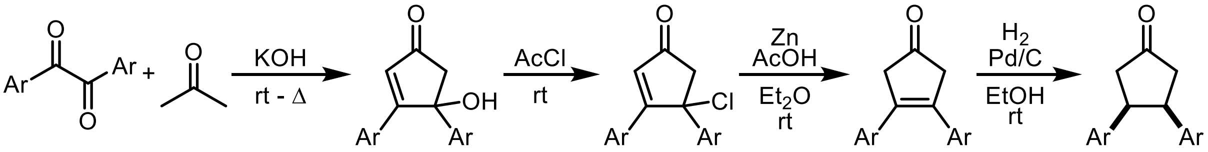

1. A slightly modified literature procedure was followed.[4] The corresponding benzil (1.0 eq.) was dissolved in acetone (2 eq.), 0.1 mL of 32% aqueous solution of KOH was added and the reaction was stirred at room temperature for 5 min. Afterwards 2 mL of 32% aqueous solution of KOH were added and the reaction was heated to reflux for 1 h. The mixture was allowed to cool down to room temperature, poured into water and extracted with CH_2Cl_2 (3 × 10 mL). The combined organic layers were dried over $MgSO_4$, filtered and the solvent was removed under reduced pressure. The residue was crystallized from boiling toluene to yield the corresponding product.

2. A solution of the corresponding 4-hydroxydiarylcyclopent-3-enone (1 eq.) was dissolved in AcCl (0.3 M) and stirred at room temperature for 5 h. The volatile components were removed under reduced pressure and the product was separated via flash column chromatography with the conditions given in the corresponding entry.

3. A solution of the corresponding 4-chlorodiarylcyclopent-3-enone (1 eq.) in Et_2O (0.13 M) was added to a suspension of zinc dust (33 eq.) in Et_2O (3.4 M) and AcOH (8.5 eq.). The mixture was stirred for 30 min, filtered over Celite and washed with Et_2O. The organic layer was washed with water and a saturated solution of $NaHCO_3$, dried over $MgSO_4$, filtered and the solvent was removed under reduced pressure.

4. The corresponding diarylcyclopent-3-one (1eq.) and Pd/C (10 mol%, 10% Pd) in ethanol was stirred under H_2 (balloon) atmosphere for 4 h. The mixture was filtered over celite, washed with ethyl acetate and evaporated under reduced pressure. The product was separated via flash column chromatography with the conditions given in the corresponding entry.

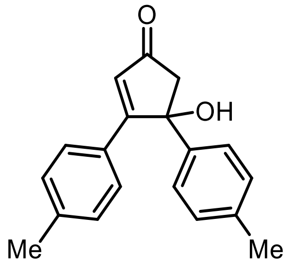

4-Hydroxy-3,4-di-*p*-tolylcyclopent-2-en-1-one [S1o]

Following **GP-B-1** using 4,4´-dimethylbenzil (5.00 g, 20.9 mmol, 1.00 eq.) the product (2.27 g, 8.15 mmol, 39%) was obtained as a yellow solid.

MP: 171-174 °C. **^{1}H NMR ($CDCl_3$, 400 MHz):** δ (ppm) = 7.45 (d, *J* = 8.3 Hz, 2H), 7.35 (d, *J* = 8.3 Hz, 2H), 7.23 – 7.04 (m, 4H), 6.68 (s, 1H), 3.01 (d, *J* = 18.5 Hz, 2H), 2.88 (d, *J* = 18.5 Hz, 2H), 2.34 (*app.* d, *J* = 5.8 Hz, 6H). **^{13}C NMR ($CDCl_3$, 101 MHz):** δ (ppm) = 204.9, 174.0, 141.6, 141.5, 137.3, 129.7, 129.7, 129.3, 128.7, 128.4, 124.2, 81.8, 56.8, 21.6, 21.2. The spectroscopic data were in agreement to those previously reported.[7] **HRMS (ESI):** calculated for $C_{19}H_{19}O_2$ $[M+H]^+$: 279.1380, found: 279.1373.

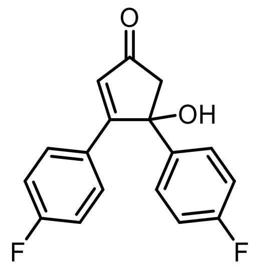

3,4-Bis(4-fluorophenyl)-4-hydroxycyclopent-2-en-1-one [S1p]

Following **GP-B-1** using 4,4´-difluorobenzil (5.00 g, 20.3 mmol, 1.00 eq.) the product (3.93 g, 13.7 mmol, 68%) was obtained as a yellow solid.

MP: 155-156 °C. **^{1}H NMR (CDCl$_3$, 400 MHz):** δ (ppm) = 7.61 – 7.52 (m, 2H), 7.43 – 7.36 (m, 2H), 7.06 – 6.95 (m, 4H), 6.62 (s, 1H), 2.99 (*app.* d, *J* = 18.7 Hz, 2H), 2.87 (d, *J* = 18.7 Hz, 1H). **^{13}C NMR (CDCl$_3$, 101 MHz):** δ (ppm) = 204.6, 172.7, 164.5 (d, *J* = 210.7 Hz), 162.0 (d, *J* = 203.4 Hz), 139.6 (d, *J* = 3.2 Hz), 131.6 (d, *J* = 8.6 Hz), 128.8, 127.5 (d, *J* = 3.6 Hz), 126.2 (d, *J* = 8.1 Hz), 116.2 (d, *J* = 21.6 Hz), 115.9 (d, *J* = 21.6 Hz), 81.4, 56.8. **^{19}F NMR (282 MHz, CDCl$_3$)**: δ (ppm) = -107.57 – -107.73 (m), -114.69 – -114.85 (m). The spectroscopic data were in agreement to those previously reported.[8]

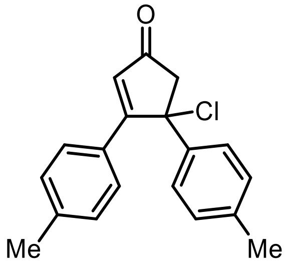

4-Chloro-3,4-di-*p*-tolylcyclopent-2-en-1-one [S2o]

Following **GP-B-2** using 4-hydroxy-3,4-di-*p*-tolylcyclopent-2-en-1-one **[S1o]** (989 mg, 3.56 mmol, 1.00 eq.) the product (766 mg, 2.84 mmol, 84%) was obtained as a yellow oil.

IR (neat): ṽ = 2922 (w), 1781 (w), 1716 (s), 1697 (s), 1609 (m), 1590 (m), 1561 (m), 1510 (m), 1449 (w), 1407 (w), 1324 (w), 1264 (w), 1192 (m), 1020 (w), 962 (m), 816 (s), 735 (m), 724 (m), 683 (w), 560 (w), 504 (m), 473 (w), 439 (w), 419 (w). **^{1}H NMR (CDCl$_3$, 400 MHz)**: δ (ppm) = 7.44 (d, *J* = 8.4 Hz, 2H), 7.36 (d, *J* = 8.3 Hz, 2H), 7.19 – 7.04 (m, 4H), 6.81 (s, 1H), 3.46 (d, *J* = 19.0 Hz, 1H), 3.05 (d, *J* = 19.0 Hz, 1H), 2.33 (s, 6H). **^{13}C NMR (CDCl$_3$, 101 MHz)**: δ (ppm) = 203.1, 173.0, 141.9, 139.5, 138.0, 129.8, 129.6, 129.4, 128.8, 128.1, 125.5, 73.8, 59.7, 21.6, 21.1. **HRMS (ESI)**: calculated for $C_{19}H_{18}ClO$ [M+H]$^+$: 297.1033, found: 297.1041.

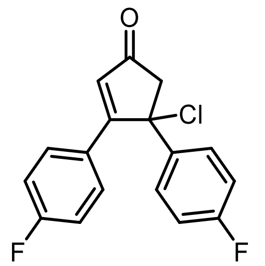

4-Chloro-3,4-bis(4-fluorophenyl)cyclopent-2-en-1-one [S2o]

Following **GP-B-2** using 3,4-bis(4-fluorophenyl)-4-hydroxycyclopent-2-en-1-one **[S1p]** (2.0 g, 6.98 mmol, 1.00 eq.) the product (1.35 g, 4.43 mmol, 63%) was obtained as a yellow oil.

IR (neat): ṽ = 1779 (w), 1715 (s), 1601 (s), 1507 (s), 1409 (w), 1230 (s), 1161 (s), 1099 (w), 1015 (w), 967 (w), 835 (s), 737 (w), 519 (m), 440 (w), 410 (w). **^{1}H NMR ($CDCl_3$, 400 MHz):** δ (ppm) = 7.57 – 7.50 (m, 2H), 7.49 – 7.42 (m, 2H), 7.08 – 6.97 (m, 4H), 6.80 (s, 1H), 3.48 (d, *J* = 19.0 Hz, 1H), 3.05 (d, *J* = 19.0 Hz, 1H). **^{13}C NMR ($CDCl_3$, 101 MHz):** δ (ppm) = 202.2, 171.3, 164.6 (d, *J* = 190.3 Hz), 162.1 (d, *J* = 184.6 Hz), 137.9 (d, *J* = 3.5 Hz), 131.9 (d, *J* = 8.8 Hz), 129.6, 127.6 (d, *J* = 8.3 Hz), 126.8 (d, *J* = 3.6 Hz), 116.1 (d, *J* = 21.8 Hz), 73.1, 59.4. **^{19}F NMR (282 MHz, $CDCl_3$)**: δ (ppm) = -107.14 – -107.49 (m), -113.38 – -113.62 (m). **HRMS (ESI):** calculated for $C_{17}H_{12}ClF_2O$ $[M+H]^+$: 305.0539, found: 305.0526.

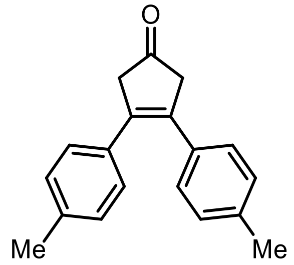

3,4-Di-*p*-tolylcyclopent-3-en-1-one [S3o]

Following **GP-B-3** using 4-chloro-3,4-di-*p*-tolylcyclopent-2-en-1-one **[S2o]** (700 mg, 2.35 mmol, 1.00 eq.) the product (493 mg, 1.89 mmol, 80%) was obtained as a yellow oil.

IR (neat): ṽ = 3024 (w), 2920 (w), 1753 (s), 1690 (w), 1514 (m), 1450 (w), 1393 (w), 1349 (w), 1183 (m), 1116 (w), 1021 (w), 815 (s), 719 (w), 565 (w), 513 (m), 498 (w), 458 (w), 433 (w). **^{1}H NMR (C_6D_6, 400 MHz)**: δ (ppm) = 7.01 (d, *J* = 8.2 Hz, 4H), 6.86 (d, *J* = 8.1 Hz, 4H), 3.07 (s, 4H), 2.06 (s, 6H). **^{13}C NMR (C_6D_6, 101 MHz)**: δ (ppm) = 211.6, 137.1, 134.4, 134.1, 129.3, 128.5, 48.8, 21.2. **HRMS (ESI)**: calculated for $C_{19}H_{19}O$ $[M+H]^+$: 263.1430, found: 263.1424.

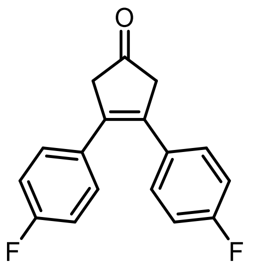

3,4-Bis(4-fluorophenyl)cyclopent-3-en-1-one [S3p]

Following **GP-B-3** using 4-chloro-3,4-di-p-tolylcyclopent-2-en-1-one **[S2p]** (700 mg, 2.35 mmol, 1.00 eq.) the product (493 mg, 1.89 mmol, 80%) was obtained as a yellow oil.

IR (neat): ṽ = 1755 (m), 1687 (m), 1602 (m), 1508 (s), 1232 (s), 1162 (m), 836 (s), 574 (m), 556 (m), 525 (m), 510 (m), 488 (m), 453 (m), 434 (m), 419 (s), 407 (m). **^{1}H NMR (C_6D_6, 400 MHz)**: δ (ppm) = 6.76 – 6.68 (m, 4H), 6.68 – 6.61 (m, 4H), 2.89 (s, 4H). **^{13}C NMR (C_6D_6, 101 MHz)**: δ (ppm) = 210.7, 162.4 (d, *J* = 247.4 Hz), 133.6, 132.6 (d, *J* = 3.4 Hz), 130.2 (d, *J* = 7.8 Hz), 115.5 (d, *J* = 21.4 Hz), 48.5. **^{19}F NMR (282 MHz, C_6D_6)**: δ = -113.49 – -113.65 (m). **HRMS (ESI)**: calculated for $C_{17}H_{13}F_2O$ $[M+H]^+$: 271.0929, found: 271.0936.

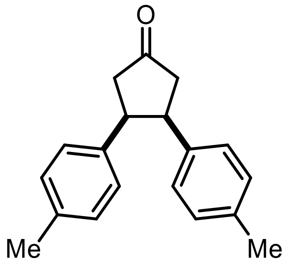

3,4-Di-*p*-tolylcyclopentan-1-one [S4o]

Following **GP-B-4** using 3,4-di-*p*-tolylcyclopent-3-en-1-one **[S3o]** (200 mg, 0.76 mmol, 1.00 eq.) the product (96 mg, 0.37 mmol, 48%) was obtained as a yellow oil.

IR (neat): ṽ = 2920 (w), 1743 (s), 1515 (m), 1150 (w), 816 (m), 599 (m), 569 (m), 550 (m), 527 (m), 502 (m), 485 (s), 475 (m), 437 (m), 427 (m). **^{1}H NMR (C_6D_6, 400 MHz)**: δ (ppm) = 6.77 (d, *J* = 7.9 Hz, 4H), 6.55 (d, *J* = 8.1 Hz, 4H), 3.32 – 3.20 (m, 2H), 2.61 – 2.50 (m, 2H), 2.32 – 2.19 (m, 2H), 2.00 (s, 6H). **^{13}C NMR (C_6D_6, 101 MHz)**: δ (ppm) = 216.2, 137.3, 135.9, 128.9, 128.3, 47.1, 43.4, 21.0. **HRMS (ESI)**: calculated for $C_{19}H_{21}O$ $[M+H]^+$: 265.1587, found: 265.1583.

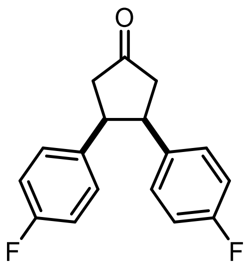

3,4-Bis(4-fluorophenyl)cyclopentan-1-one [S4p]

Following **GP-B-4** using 3,4-bis(4-fluorophenyl)cyclopent-3-en-1-one **[S3p]** (350 mg, 1.29 mmol, 1.00 eq.) the product (136 mg, 0.50 mmol, 39%) was obtained as a yellow oil.

^{1}H NMR (C_6D_6, 400 MHz): δ (ppm) = 6.55 (t, *J* = 8.7 Hz, 4H), 6.28 – 6.20 (m, 4H), 3.06 – 2.95 (m, 2H), 2.30 (ddd, *J* = 19.0, 4.6, 1.6 Hz, 2H), 2.11 (ddd, *J* = 18.9, 6.0, 2.2 Hz, 2H). **^{13}C NMR (C_6D_6, 101 MHz)**: δ (ppm) = 215.3, 162.0 (d, *J* = 244.8 Hz), 135.5 (d, *J* = 3.3 Hz), 129.6 (d, *J* = 7.8 Hz), 114.9 (d, *J* = 21.2 Hz), 46.5, 43.0. **^{19}F NMR (282 MHz, C_6D_6)**: δ (ppm) = -117.12 – -117.26 (m). The spectroscopic data were in agreement to those previously reported.[9]

3. Optimization for enantioselective *N*-Insertion

For all optimizations, *O*-(diphenylphosphinyl)hydroxylamine (DPPH) was used, based on prior results which identified it as the most effective nitrogen inserting agent in these systems.[10] Optimization was initiated by identifying the optimal catalyst to investigate the enantioselective condensation and subsequent enantiospecific rearrangement. For these experiments, the focus was placed on catalyst optimization with respect to enantioselectivity; reaction yields were not determined and are therefore not reported. All reactions, however, provided the desired product in acceptable yields.

Table *S1*. Catalyst optimization, reactions were carried out on a 0.05 mmol scale. The enantiomeric purity was established by HPLC analysis using a chiral column.

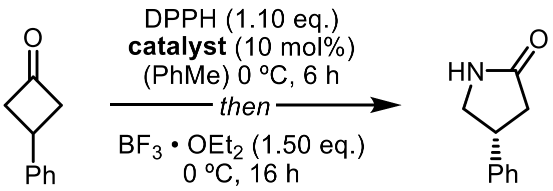

entry	catalyst	er
1	**S5**	15:85
2	**S6**	35:65
3	**S7**	27:73
4	**S8**	30:70
5	**4**	91:9
6	**S9**	84:16

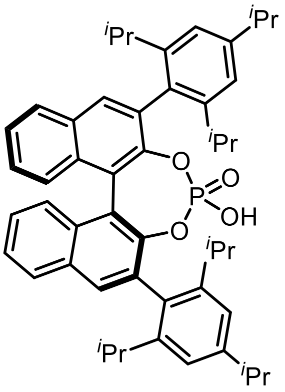

(*S*)-TRIP (**S5**)

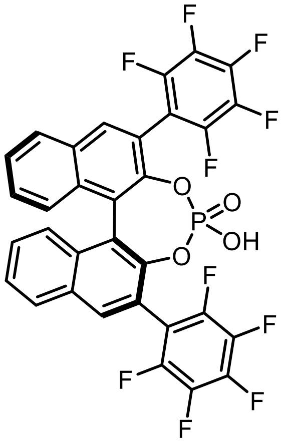

(*S*)-3,3′-C_6F_5-BINOL-P (**S6**)

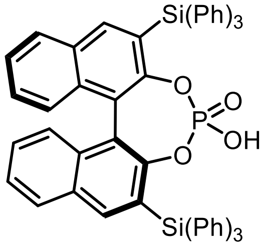

(*S*)-3,3'-$SiPh_3$-BINOL-P (**S7**)

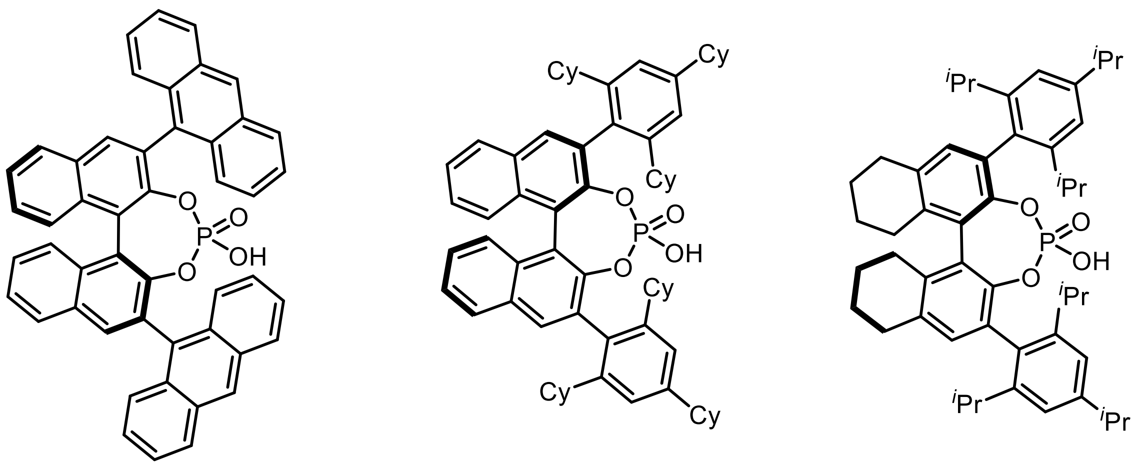

(*S*)-3,3´-(9-antracenyl)-BINOL-P (**S8**) (*R*)-TCYP (**4**) (*R*)-H8-TRIP (**S9**)

The enantiospecific ring expansion was investigated using the chiral oxime ester as a starting material, thereby eliminating interference of the catalyst. Under these conditions, the effects of different Lewis acids, the equivalents and the water content of the reagents were systematically examined to enhance the yield of the rearrangement.

Table *S2.* Optimization of Lewis acid, reactions were carried out at 0.1 mmol scale, yield based on ^{1}H NMR experiments using CH_2Br_2 as an internal standard.

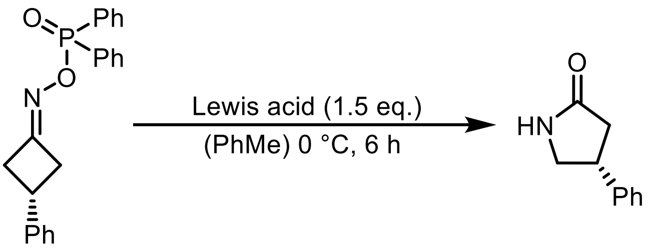

entry	Lewis acid	Conversion	Yield
1	BF_3·OEt_2	100%	96%
2	BF_3·THF	85%	65%
3	Al_2O_3 (basic)	0%	0%
4	Al_2O_3 (neutral)	0%	0%
5	Al_2O_3 (acidic)	0%	0%

For the experiments regarding the added equivalents of BF_3·OEt_2 we were interested in whether a sub stoichiometric amount could be used to achieve a catalytic rearrangement (**Table *S3***). Unfortunately, when the amount of BF_3·OEt_2 was reduced to a substoichiometric amount the yield dropped significantly. The best results were obtained with a slight excess of BF_3·OEt_2.

Table *S3*. Optimization of equivalents of $BF_3{\cdot}OEt_2$, reactions were carried out at 0.2 mmol scale, yield based on ^{1}H NMR experiments using CH_2Br_2 as an internal standard.

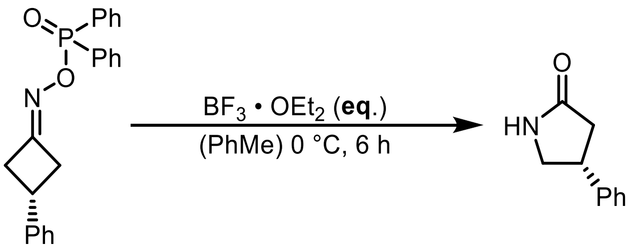

entry	$BF_3{\cdot}OEt_2$ (eq.)	Yield	er oxime ester	er lactam
1	0.50	<5%	94:6	-
2	1.00	86%	94:6	93:7
3	1.50	96%	94:6	94:6
4	10.0	73%	94:6	93:7

Table *S4*. Water content optimization, reactions were carried out at 0.2 mmol scale, yield based on ^{1}H NMR experiments using CH_2Br_2 as an internal standard. The enantiomeric purity was established by HPLC analysis using a chiral column.

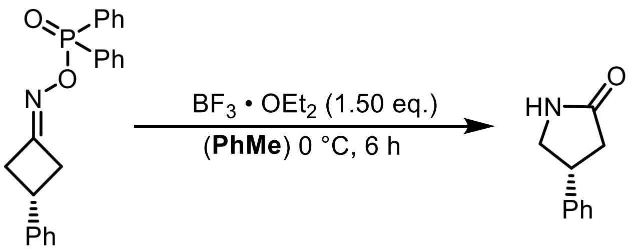

entry	toluene	$BF_3{\cdot}OEt_2$	yield	er oxime ester	er lactam
1	Dry	Bottle	63%	93:7	93:7
2	Dry	Distilled	71%	87:13	82:18
3	Saturated with H_2O	Bottle	96%	93:7	93:7

During the optimization of the reaction, we encountered reproducibility issues. Mechanistic considerations suggested that water plays a crucial role in the rearrangement step. Although $BF_3{\cdot}OEt_2$ is dry, it is known to gradually absorb moisture, leading to variable water content and, consequently, inconsistent reaction outcomes. With freshly distilled boron trifluoride over CaH_2 the yield of the lactam improved slightly but the enantiomeric ratio decreased.

To address this issue, we pre-saturated the solvent (toluene) with water prior to the reaction. This approach resulted in improved yields and significantly enhanced reproducibility. In contrast, when using dried toluene, we observed lower yields and the formation of side product **8a** (for further information see 4. Mechanistic investigations).

We also investigated quenching of the residual Brønsted acid before the addition of $BF_3 \cdot OEt_2$ to lower the interference of the two acids. Both attempts resulted in lower enantiomeric ratios. Attempt with TMS diazomethane also showed a significant decreased in the yield.

Table *S5*. Attempts of quenching the residual Brønsted acid before the addition of $BF_3 \cdot OEt_2$. Reactions were carried out at 0.2 mmol scale, yield based on ^{1}H NMR experiments using CH_2Br_2 as an internal standard. The enantiomeric purity was established by HPLC analysis using a chiral column.

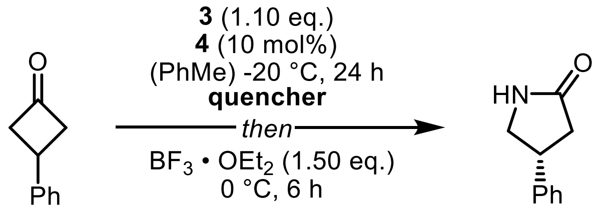

entry	quencher	yield	er
1	triethylamine	87%	90:10
2	TMS diazomethane	66%	66:34

Finally, the impact of temperature on the condensation step was investigated in a stepwise fashion with the aim of improving the enantiomeric ratio of the resulting lactam. As expected, lower temperatures led to slower conversion compared to 0 °C. However, this adjustment resulted in a significant improvement in enantioselectivity, increasing the enantiomeric ratio from 83:17 to 96:4.

Table *S6*. Temperature optimization, reactions were carried out at 0.2 mmol scale. The enantiomeric purity was established by HPLC analysis using a chiral column.

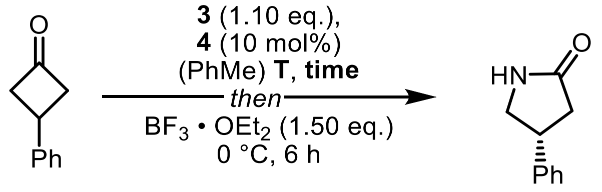

entry	temperature	time	er
1	0 °C	6 h	83:17
2	–10 °C	16 h	90:10
3	–20 °C	24 h	91:9
4	–30 °C	3 d	95:5
5	–40 °C	3 d	96:4

4. Mechanistic investigations

We were able to isolate a sideproduct [**8a**] of the reaction while working in strictly anhydrous conditions (dried toluene with additional activated 4 Å molecular sieves). This sideproduct – to our knowledge – can only be formed through a trapping of a formed nitrilium ion [**7a**] by the released phosphinic acid leading to structure [**9a**] which undergoes a Chapman-type rearrangement towards the shown sideproduct **8a**.

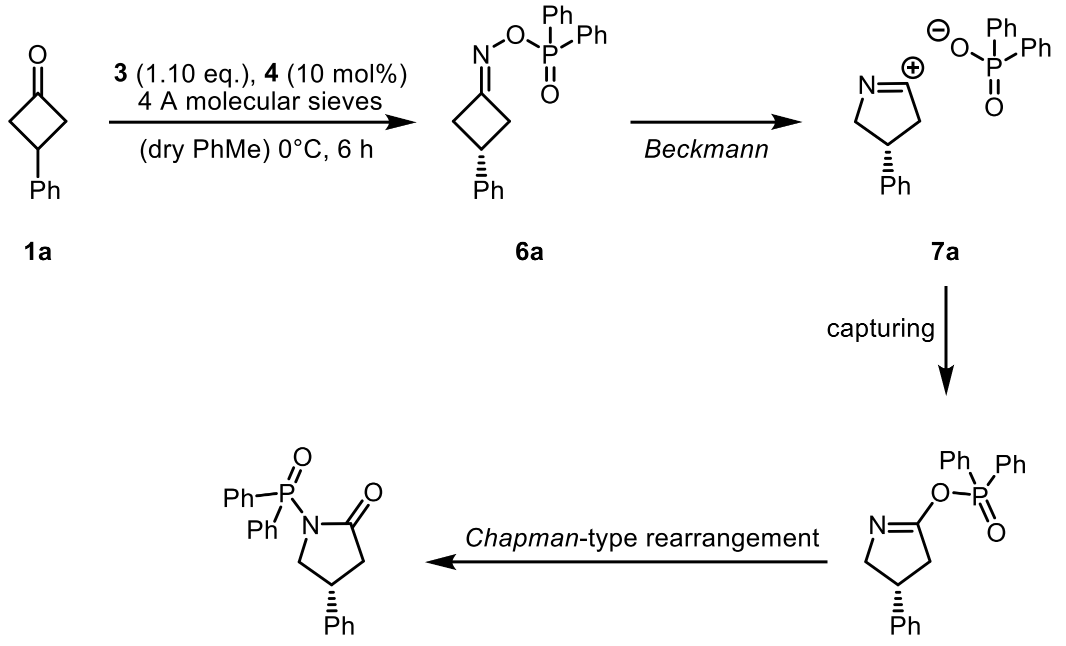

Scheme *S1*: Mechanistic proposal for the formation of sideproduct 8a.

To further proof the correct assignment of the proposed sideproduct **8a** we synthesized it by a lactam substitution route. Identical spectroscopic data were measured for the independently synthesized phosphinyl lactams. In addition, NOE data shows that the phosphinyl group is attached to the nitrogen (and not oxygen). The respective data are attached at the end of this document.

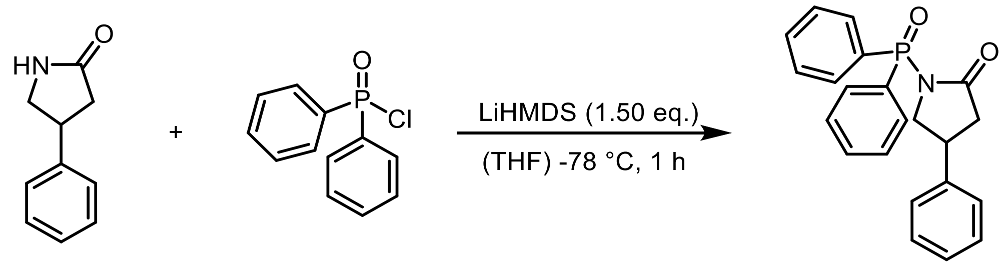

1-(Diphenylphosphoryl)-4-phenylpyrrolidin-2-one [*rac*-8a]

To a solution of 4-phenylpyrrolidin-2-one [**2a**] (32.2 mg, 200 µmol, 1.00 eq.) in THF (1.0 mL) LiHMDS (300 µL, 300 µmol, 1.00 M in THF, 1.50 eq.) was added under Ar atmosphere at -78 °C.

After stirring at -78 °C for 30 min, diphenylphosphinic chloride (56.8 mg, 45.8 µL, 240 µmol, 1.20 eq.) was added. Then the reaction mixture was allowed to warm to room temperature and stirred for 30 min. The reaction was diluted with water (5 mL) mixture was extracted with DCM (2 x 10 mL), the organic phase was washed with water and saturated brine, dried with anhydrous Na_2SO_4, the solvent was evaporated under reduced pressure and the crude residue was purified *via* flash column chromatography (SiO_2, EtOAc) to give the product (32.5 mg, 89.9 µmol, 45%) as a colorless oil.

IR (neat): ṽ = 3059 (w), 1715 (s), 1484 (w), 1438 (m), 1350 (w), 1216 (s), 1125 (s), 1030 (w), 963 (w), 752 (m), 731 (s), 697 (s), 601 (w), 548 (s), 538 (s), 519 (m), 511 (m). **^{1}H NMR (400 MHz, CDCl$_3$)**: δ (ppm) = 7.93 – 7.78 (m, 4H), 7.58 (ddq, *J* = 8.7, 4.5, 1.5 Hz, 2H), 7.53 – 7.43 (m, 3H), 7.37 – 7.30 (m, 2H), 7.29 – 7.24 (m, 1H), 7.24 – 7.20 (m, 2H), 4.28 – 4.15 (m, 1H), 3.92 – 3.58 (m, 2H), 2.94 – 2.83 (m, 1H), 2.77 – 2.67 (m, 1H). **^{13}C NMR (101 MHz, CDCl$_3$)**: δ (ppm) = 177.4, 140.5, 132.8 (d, *J* = 3.0 Hz), 132.1 (d, *J* = 5.1 Hz), 132.0 (d, *J* = 5.2 Hz), 130.3 (d, *J* = 126.9 Hz), 130.2 (d, *J* = 126.1 Hz), 129.1, 128.7 (d, *J* = 1.8 Hz), 128.6 (d, *J* = 1.7 Hz), 127.5, 126.8, 53.1 (d, *J* = 2.6 Hz), 40.4 (d, *J* = 5.0 Hz), 39.6 (d, *J* = 6.1 Hz). **^{31}P NMR (162 MHz, CDCl$_3$)**: δ (ppm) = 26.55. **HRMS (ESI)**: calculated for $C_{22}H_{20}NNaO_2P$ [M+Na]$^+$: 384.1124, Found: 384.1108.

We also tried trapping the formed nitrilium ion [**7a**] using methanol and trimethoxybenzene as nucleophiles. These nucleophiles were chosen inspired by literature precedent.[11] However, we were not able to detect any species arising from an intercepted Beckmann rearrangement.

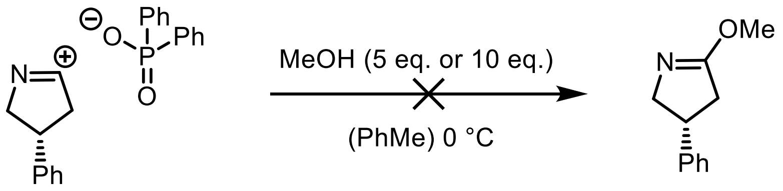

7a

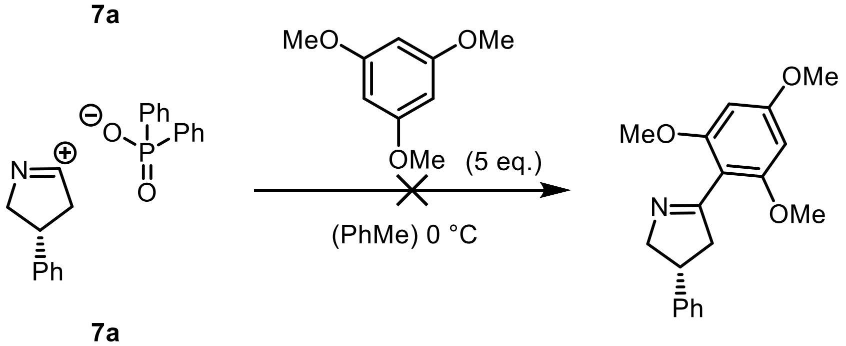

7a

5. Substrate scope of lactams

The racemic γ-lactams were prepared according to a slightly modified procedure reported in the literature.[3]

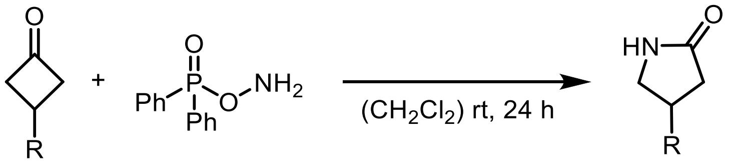

3

An oven dried tube was charged with *O*-(diphenylphosphinyl)hydroxylamine (**3**, 220 µmol, 1.10 eq.) and suspended in CH_2Cl_2 (0.7 mL, 0.33 M). The corresponding cyclobutanone (200 µmol, 1.00 eq.) was dissolved in CH_2Cl_2 (0.4 mL, 0.50 M) and added to the mixture. The mixture was stirred at room temperature for 24 h. The solvent was concentrated under reduced pressure and the product was separated from the corresponding oxime byproduct by silica gel column chromatography.

General procedure C (GP-C) for the synthesis of the γ-lactams:

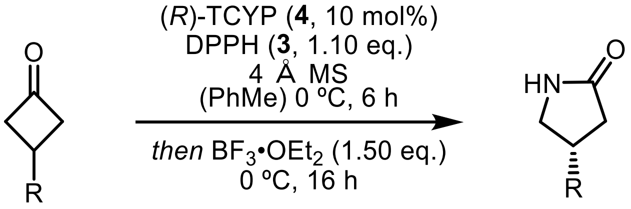

In a tube, (*R*)-TCYP (**4**, 19.9 mg, 0.02 mmol, 10 mol%) and the corresponding cyclobutanone (0.20 mmol, 1.00 eq.) were dissolved in dry toluene (0.05 M) under N_2-atmosphere. 4 Å molecular sieves was added and the mixture was cooled to 0 °C. *O*-Diphenylphosphorylhydroxylamine (**3**, 51.3 mg, 0.22 mmol, 1.10 eq.) was added and the reaction mixture was stirred at 0 °C for 6 h. Triethylamine (20.2 mg, 27.9 µL, 0.22 mmol, 1.00 eq.) was added and the reaction mixture was stirred for 15 min. Boron trifluoride etherate (1.50 eq.) was added and the reaction mixtures was stirred at 0 °C for 16 h. After quenching with an aqueous saturated solution of $NaHCO_3$ (4 mL), the aqueous phase was extracted with CH_2Cl_2 (3 x 40 mL) and $CHCl_3$/^{i}PrOH (3:1, 1 x 40 mL). The combined organic phases were dried over Na_2SO_4, filtered and concentrated under reduced pressure. The crude product was purified via column chromatography (SiO_2, EtOAc:MeOH, 100:0 to 95:5, stained with $KMnO_4$).

General procedure D (GP-D) for the synthesis of the γ-lactams:

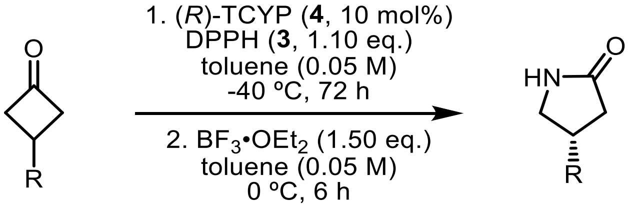

In a tube, (*R*)-TCYP (**4**, 19.9 mg, 0.02 mmol, 10 mol%) and the corresponding cyclobutanone (0.20 mmol, 1.00 eq.) were dissolved in toluene (0.05 M) and cooled to -40 °C. *O*-Diphenylphosphorylhydroxylamine (**3**, 51.3 mg, 0.22 mmol, 1.10 eq.) was added and the reaction mixture was stirred at -40 °C for 72 h. Triethylamine (20.2 mg, 27.9 µL, 0.22 mmol, 1.00 eq.) was added and the crude was concentrated under reduced pressure. The corresponding oxime ester was purified via column chromatography (SiO_2, *n*-hexane:EtOAc 100:0 to 0:100).

In a Schlenk tube, the corresponding purified oxime ester was dissolved in toluene (0.05 M) which was stirred with water for 30 min. The mixture was cooled to 0 °C in a water/ice bath and boron trifluoride etherate (1.50 eq.) was added. The reaction mixture was stirred for 6 h and quenched with an aqueous saturated solution of $NaHCO_3$ (4 mL). The aqueous phase was extracted with CH_2Cl_2 (3 x 40 mL) and $CHCl_3$/^{i}PrOH (3:1, 1 x 40 mL). The combined organic phases were dried over Na_2SO_4, filtered and concentrated under reduced pressure. The crude product was purified via column chromatography (SiO_2, EtOAc:MeOH, 100:0 to 95:5, stained with $KMnO_4$).

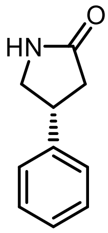

(*R*)-4-Phenylpyrrolidin-2-one [2a]

Following **GP-C** using 3-phenylcyclobutan-1-one **[1a]** (29.2 mg, 200 µmol, 1.00 eq.) the product (26.4 mg, 164 µmol, 82%) was obtained via flash column chromatography (SiO_2, EtOAc:MeOH, 100:0 to 95:5, stained with $KMnO_4$) as a colorless solid.

^{1}H NMR (400 MHz, $CDCl_3$): δ (ppm) = 7.41 – 7.33 (m, 2H), 7.32 – 7.24 (m, 3H), 7.05 (s, 1H), 3.86 – 3.77 (m, 1H), 3.71 (*app.* p, *J* = 8.5 Hz, 1H), 3.45 (dd, *J* = 9.4, 7.2 Hz, 1H), 2.76 (dd, *J* = 16.9, 8.9 Hz, 1H), 2.53 (dd, *J* = 16.9, 8.9 Hz, 1H).**^{13}C NMR (101 MHz, $CDCl_3$)**: δ (ppm) = 178.1, 142.2, 129.0, 127.2, 126.9, 49.73, 40.4, 38.2. The spectroscopic data were in agreement to those previously reported.[3] **Optical Rotation**: $[\alpha]_D^{25}$= –30.5 (c = 0.54, $CHCl_3$) for an enantiomerically enriched sample of 87:13 *er*. The enantiomeric purity was established by HPLC analysis using a chiral column (Lux® *i*Amylose-3, 40 °C, 1 mL/min, 90:10 *n*-hexane:isopropanol, 214 nm, t = 13.061 min and 14.444 min).

Following **GP-D** using 3-phenylcyclobutan-1-one **[1a]** (29.2 mg, 200 µmol, 1.00 eq.) the product (20.0 mg, 124 µmol, 63%) was obtained via flash column chromatography (SiO_2, EtOAc:MeOH, 100:0 to 95:5, stained with $KMnO_4$) as a colorless solid.

Optical Rotation: $[\alpha]_D^{25}$= –33.9 (c = 0.5, $CHCl_3$) for an enantiomerically enriched sample of 95:05 *er*. The enantiomeric purity was established by HPLC analysis using a chiral column (Lux®

*i*Amylose-3, 40 °C, 1 mL/min, 90:10 *n*-hexane:isopropanol, 214 nm, t = 13.061 min and 14.444 min).

1.0 mmol scale:

Following **GP-D** using 3-phenylyclobut-1-one [**1a**] (146 mg, 1.00 mmol, 1.00 eq.), (*R*)- TCYP [**4**] (101 mg, 100 µmol, 10 mol%) and *O*-(diphenylphosphinyl)hydroxylamine [**3**] (257 mg, 1.10 mmol, 1.10 eq.) the product (109 mg, 676 µmol, 70%) was obtained via automated flash column chromatography (SiO_2, EtOAc:MeOH, 100:0 to 95:5, stained with $KMnO_4$) as colorless solid.

Optical Rotation: $[\alpha]_D^{25}$= –34.8 (c = 1.0, $CHCl_3$) for an enantiomerically enriched sample of 93:07 er. The enantiomeric purity was established by HPLC analysis using a chiral column (Lux® *i*Amylose-3, 40 °C, 1 mL/min, 90:10 *n*-hexane:isopropanol, 214 nm, t = 13.061 min and 14.444 min).

Reisolation of the catalyst [**4**]:

Following a procedure from Klussmann *et al.*[12], the catalyst isolated by flash column chromatography was dissolved in CH_2Cl_2 (20 mL). The organic phase was washed with 1.0 M HCl (3 x 10 mL), dried over Mg_2SO_4 and concentrated under reduced pressure. (*R*)-TCYP [**4**] (93 mg, 93.6 µmol, 91%) was obtained after recrystallization from acetonitrile as a colorless solid.

^{1}H NMR (400 MHz, $CDCl_3$): δ (ppm) = 7.84 (d, *J* = 8.2 Hz, 2H), 7.70 (s, 2H), 7.44 (ddd, *J* = 8.1, 6.4, 1.5 Hz, 2H), 7.31 – 7.17 (m, 4H), 6.93 – 6.86 (m, 4H), 2.51 – 2.35 (m, 2H), 2.24 – 2.01 (m, 4H), 1.97 – 0.40 (m, 60H). **^{13}C NMR (101 MHz, $CDCl_3$)**: δ (ppm) = 147.0, 146.6, 146.5, 146.4, 146.3, 132.3, 132.2, 131.9, 131.8, 131.0, 128.2, 126.8, 126.2, 125.6, 122.4, 121.8, 121.6, 44.9, 42.3, 41.9, 37.1, 35.2, 34.8, 34.3, 33.3, 32.7, 27.5, 27.3, 27.3, 27.10, 26.8, 26.5, 26.4, 25.9. **^{31}P NMR (162 MHz, $CDCl_3$)**: δ (ppm) = 1.27. The spectroscopic data were in agreement to those previously reported.[13]

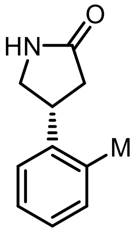

(*R*)-4-(2-Methylphenyl)pyrrolidin-2-one [2b]

Following **GP-D** using 3-(2-methylphenyl)cyclobutan-1-one **[1b]** (32.1 mg, 200 µmol, 1.00 eq.) the product (24.7 mg, 141 µmol, 75%) was obtained via flash column chromatography (SiO_2, EtOAc:MeOH, 100:0 to 95:5, stained with $KMnO_4$) as a colorless solid.

^{1}H NMR (400 MHz, $CDCl_3$): δ (ppm) = 7.29 (dd, *J* = 7.4, 1.3 Hz, 1H), 7.25 – 7.13 (m, 3H), 6.68 (s, 1H), 3.97 – 3.84 (m, 1H), 3.77 (ddd, *J* = 9.1, 8.0, 0.9 Hz, 1H), 3.41 (dd, *J* = 9.6, 6.5 Hz, 1H), 2.73

(dd, J = 16.9, 9.0 Hz, 1H), 2.48 (dd, J = 17.0, 7.8 Hz, 1H), 2.35 (s, 3H). **^{13}C NMR (101 MHz, $CDCl_3$)**: δ (ppm) = 178.0, 140.4, 135.8, 130.8, 127.0, 126.8, 125.4, 48.9, 37.6, 36.2, 19.8. The spectroscopic data were in agreement to those previously reported.[14] **Optical Rotation**: $[\alpha]_D^{25}$ = –30.9 (c = 0.5, $CHCl_3$) for an enantiomerically enriched sample of 94:06 *er*. The enantiomeric purity was established by HPLC analysis using a chiral column (Lux® Cellulose-1, 40 °C, 1 mL/min, 90:10 *n*-hexane:isopropanol, 214 nm, t = 16.549 min and 18.904 min).

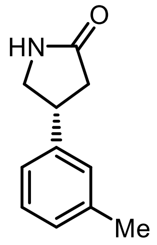

(*R*)-4-(3-Methylphenyl)pyrrolidin-2-one [2c]

Following **GP-D** with a slight modification (condensation reaction was perfomed at -20 °C for 24 h) using 3-(3-methylphenyl)cyclobutan-1-one **[1c]** (32.1 mg, 200 µmol, 1.00 eq.) the product (22.8 mg, 130 µmol, 67%) was obtained via flash column chromatography (SiO_2, EtOAc:MeOH, 100:0 to 95:5, stained with $KMnO_4$) as a colorless solid.

^{1}H NMR (400 MHz, $CDCl_3$): δ (ppm) = 7.24 (td, J = 7.5, 1.2 Hz, 1H), 7.12 – 7.01 (m, 3H), 6.09 (s, 1H), 3.77 (ddd, J = 9.2, 8.2, 1.1 Hz, 1H), 3.73 – 3.60 (m, 1H), 3.42 (dd, J = 9.3, 7.3 Hz, 1H), 2.72 (dd, J = 16.9, 8.9 Hz, 1H), 2.51 (dd, J = 16.9, 8.9 Hz, 1H), 2.35 (d, J = 0.7 Hz, 3H). **^{13}C NMR (101 MHz, $CDCl_3$)**: δ (ppm) = 177.7, 142.2, 138.7, 128.9, 128.0, 127.7, 123.9, 49.6, 40.4, 38.0, 21.6. The spectroscopic data were in agreement to those previously reported.[15] **Optical Rotation**: $[\alpha]_D^{25}$ = –36.2 (c = 1.0, $CHCl_3$) for an enantiomerically enriched sample of 95:05 *er*. The enantiomeric purity was established by HPLC analysis using a chiral column (Lux® Amylose-1, 40 °C, 1 mL/min, 90:10 *n*-hexane:isopropanol, 214 nm, t = 9.111 min and 11.372 min).

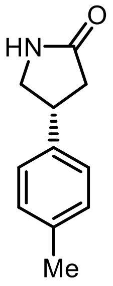

(*R*)-4-(4-Methylphenyl)pyrrolidin-2-one [2d]

Following **GP-C** using 3-(4-methylphenyl)cyclobutan-1-one **[1d]** (32.1 mg, 200 µmol, 1.00 eq.) the product (30.5 mg, 174 µmol, 87%) was obtained via flash column chromatography (SiO_2, EtOAc:MeOH, 100:0 to 95:5, stained with $KMnO_4$) as a colorless solid.

^{1}H NMR (400 MHz, $CDCl_3$): δ (ppm) = 7.15 (s, 4H), 6.28 (s, 1H), 3.76 (ddd, J = 9.3, 8.1, 1.1 Hz, 1H), 3.73 – 3.60 (m, 1H), 3.44 – 3.35 (m, 1H), 2.71 (dd, J = 16.9, 8.8 Hz, 1H), 2.49 (dd, J = 16.9,

9.0 Hz, 1H), 2.34 (s, 3H). **^{13}C NMR (101 MHz, $CDCl_3$)**: δ (ppm) = 177.8, 139.2, 136.9, 129.6, 126.8, 49.7, 40.2, 38.1, 21.1. The spectroscopic data were in agreement to those previously reported.[16] **Optical Rotation**: $[\alpha]_D^{25}$ = –28.6 (c = 0.33, $CHCl_3$) for an enantiomerically enriched sample of 91:09 *er*. The enantiomeric purity was established by HPLC analysis using a chiral column (Lux® Amylose-1, 40 °C, 1 mL/min, 90:10 *n*-hexane:isopropanol, 214 nm, t = 10.092 min and 11.037 min).

Following **GP-D** using 3-(4-methylphenyl)cyclobutan-1-one **[1d]** (32.1 mg, 200 µmol, 1.00 eq.) the product (27.4 mg, 156 µmol, 81%) was obtained via flash column chromatography (SiO_2, EtOAc:MeOH, 100:0 to 95:5, stained with $KMnO_4$) as a colorless solid.

Optical Rotation: $[\alpha]_D^{25}$ = –38.9 (c = 1, $CHCl_3$) for an enantiomerically enriched sample of 97:03 *er*. The enantiomeric purity was established by HPLC analysis using a chiral column (Lux® Amylose-1, 40 °C, 1 mL/min, 90:10 *n*-hexane:isopropanol, 214 nm, t = 10.092 min and 11.037 min).

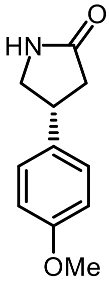

(*R*)-4-(4-Methoxyphenyl)pyrrolidin-2-one [2e]

Following **GP-C** using 3-(4-methoxyphenyl)cyclobutan-1-one **[1e]** (35.2 mg, 199 µmol, 1.00 eq.) the product (27.0 mg, 141 µmol, 71%) was obtained via flash column chromatography (SiO_2, EtOAc:MeOH, 100:0 to 95:5, stained with $KMnO_4$) as a colorless solid.

^{1}H NMR (400 MHz, $CDCl_3$): δ (ppm) = 7.15 (d, *J* = 8.5 Hz, 2H), 6.91 – 6.83 (m, 2H), 6.62 (br s, 1H), 3.79 (s, 3H), 3.71 (t, *J* = 9.3 Hz, 1H), 3.61 (p, *J* = 8.4 Hz, 1H), 3.34 (t, *J* = 8.3 Hz, 1H), 2.68 (dd, *J* = 16.9, 8.8 Hz, 1H), 2.44 (dd, *J* = 16.9, 9.0 Hz, 1H). **^{13}C NMR (101 MHz, $CDCl_3$)**: δ (ppm) = 178.0, 158.7, 134.2, 127.9, 114.3, 55.4, 49.9, 39.8, 38.2. The spectroscopic data were in agreement to those previously reported.[16] **Optical Rotation**: $[\alpha]_D^{25}$ = –24.1 (c = 0.513, $CHCl_3$) for an enantiomerically enriched sample of 88:12 *er*. The enantiomeric purity was established by HPLC analysis using a chiral column (Lux® Amylose-1, 40 °C, 1 mL/min, 90:10 *n*-hexane:isopropanol, 214 nm, t = 14.578 min and 16.141 min).

Following **GP-D** using 3-(4-methoxyphenyl)cyclobutan-1-one **[1e]** (36.0 mg, 204 µmol, 1.00 eq.) the product (21.3 mg, 111 µmol, 53%) was obtained via flash column chromatography (SiO_2, EtOAc:MeOH, 100:0 to 95:5, stained with $KMnO_4$) as a colorless solid.

Optical Rotation: $[\alpha]_D^{25}$ = –35.5 (c = 0.5, $CHCl_3$) for an enantiomerically enriched sample of 95:05 *er*. The enantiomeric purity was established by HPLC analysis using a chiral column (Lux®

Amylose-1, 40 °C, 1 mL/min, 90:10 *n*-hexane:isopropanol, 214 nm, t = 14.578 min and 16.141 min).

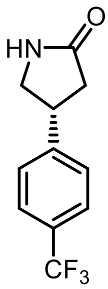

(*R*)-4-(4-(Trifluoromethyl)phenyl)pyrrolidin-2-one [2f]

Following **GP-C** using 3-(4-(trifluoromethyl)phenyl)cyclobutan-1-one **[1f]** (42.8 mg, 199 µmol, 1.00 eq.) the product (27.4 mg, 119 µmol, 60%) was obtained via flash column chromatography (SiO_2, EtOAc:MeOH, 100:0 to 95:5, stained with $KMnO_4$) as a colorless solid.

^{1}H NMR (400 MHz, $CDCl_3$): δ (ppm) = 7.63 – 7.56 (m, 2H), 7.41 – 7.34 (m, 2H), 6.85 (s, 1H), 3.87 – 3.71 (m, 2H), 3.43 (dd, *J* = 9.3, 6.6 Hz, 1H), 2.78 (dd, *J* = 16.9, 8.8 Hz, 1H), 2.49 (dd, *J* = 17.0, 8.3 Hz, 1H). **^{13}C NMR (101 MHz, $CDCl_3$)**: δ (ppm) = 177.4, 146.4, 129.6 (q, *J* = 32.6 Hz), 127.3, 126.0 (q, *J* = 3.8 Hz), 124.1 (q, *J* = 271.9 Hz), 49.36, 40.11, 37.92. **^{19}F NMR (377 MHz, $CDCl_3$)**: δ (ppm) = -62.54. The spectroscopic data were in agreement to those previously reported.[17] **Optical Rotation**: $[\alpha]_D^{25}$ = –20.7 (c = 0.53, $CHCl_3$) for an enantiomerically enriched sample of 88:12 *er*. The enantiomeric purity was established by HPLC analysis using a chiral column (Lux® Amylose-1, 40 °C, 1 mL/min, 90:10 *n*-hexane:isopropanol, 214 nm, t = 9.560 min and 11.920 min).

Following **GP-D** using 3-(4-(trifluoromethyl)phenyl)cyclobutan-1-one **[1f]** (42.7 mg, 199 µmol, 1.00 eq.) the product (32.6 mg, 142 µmol, 71%) was obtained via flash column chromatography (SiO_2, EtOAc:MeOH, 100:0 to 95:5, stained with $KMnO_4$) as a colorless solid.

Optical Rotation: $[\alpha]_D^{25}$ = –25.9 (c = 1.0, $CHCl_3$) for an enantiomerically enriched sample of 95:05 *er*. The enantiomeric purity was established by HPLC analysis using a chiral column (Lux® Amylose-1, 40 °C, 1 mL/min, 90:10 *n*-hexane:isopropanol, 214 nm, t = 9.560 min and 11.920 min).

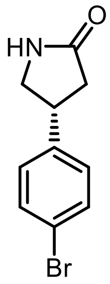

(*R*)-4-(4-Bromophenyl)pyrrolidin-2-one [2g]

Following **GP-C** using 3-(4-bromophenyl)cyclobutan-1-one **[1g]** (45.0 mg, 200 µmol, 1.00 eq.) the product (28.8 mg, 120 µmol, 60%) was obtained via flash column chromatography (SiO_2, EtOAc:MeOH, 100:0 to 95:5, stained with $KMnO_4$) as a colorless solid.

^{1}H NMR (400 MHz, $CDCl_3$): δ (ppm) = 7.49 – 7.41 (m, 2H), 7.16 – 7.08 (m, 2H), 6.88 (s, 1H), 3.78 (ddd, *J* = 9.5, 8.1, 1.0 Hz, 1H), 3.71 – 3.58 (m, 1H), 3.37 (dd, *J* = 9.6, 7.0 Hz, 1H), 2.73 (dd, *J* = 16.9, 9.0 Hz, 1H), 2.44 (dd, *J* = 16.9, 8.5 Hz, 1H). **^{13}C NMR (101 MHz, $CDCl_3$)**: δ (ppm) = 177.7, 141.3, 132.1, 128.6, 121.0, 49.5, 39.8, 38.0. The spectroscopic data were in agreement to those previously reported.[14] **Optical Rotation**: $[\alpha]_D^{25}$ = –23.7 (c = 0.5, $CHCl_3$) for an enantiomerically enriched sample of 89:11 *er*. The enantiomeric purity was established by HPLC analysis using a chiral column (Lux® Amylose-1, 40 °C, 1 mL/min, 90:10 *n*-hexane:isopropanol, 214 nm, t = 12.483 min and 14.974 min).

Following **GP-D** using 3-(4-bromophenyl)cyclobutan-1-one **[1g]** (45.0 mg, 0.2 mmol, 1.00 eq.) the product (38.9 mg, 0.16 mmol, 83%) was obtained via flash column chromatography (SiO_2, EtOAc:MeOH, 100:0 to 95:5, stained with $KMnO_4$) as a colorless solid.

Optical Rotation: $[\alpha]_D^{25}$ = –31.9 (c = 0.5, $CHCl_3$) for an enantiomerically enriched sample of 95:05 *er*. The enantiomeric purity was established by HPLC analysis using a chiral column (Lux® Amylose-1, 40 °C, 1 mL/min, 90:10 *n*-hexane:isopropanol, 214 nm, t = 12.483 min and 14.974 min).

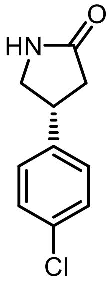

(*R*)-4-(4-Chlorophenyl)pyrrolidin-2-one [2h]

Following **GP-D** using 3-(4-chlorophenyl)cyclobutan-1-one **[1h]** (36.1 mg, 0.2 mmol, 1.00 eq.) the product (23.3 mg, 119 µmol, 63%) was obtained via flash column chromatography (SiO_2, EtOAc:MeOH, 100:0 to 95:5, stained with $KMnO_4$) as a colorless solid.

^{1}H NMR (400 MHz, $CDCl_3$) δ (ppm) = 7.35 – 7.27 (m, 2H), 7.23 – 7.15 (m, 2H), 6.36 (s, 1H), 3.78 (ddd, *J* = 9.3, 8.1, 1.0 Hz, 1H), 3.73 – 3.61 (m, 1H), 3.38 (dd, *J* = 9.4, 7.0 Hz, 1H), 2.73 (dd, *J* = 16.9, 8.9 Hz, 1H), 2.45 (dd, *J* = 16.9, 8.6 Hz, 1H). **^{13}C NMR (101 MHz, $CDCl_3$)** δ (ppm) = 177.4, 140.7, 133.1, 129.2, 128.3, 49.5, 39.8, 38.0. The spectroscopic data were in agreement to those previously reported.[18] **Optical Rotation**: $[\alpha]_D^{25}$ = –32.7 (c = 1.0, $CHCl_3$) for an enantiomerically enriched sample of 95:05 *er*. The enantiomeric purity was established by HPLC analysis using a chiral column (Lux® Amylose-1, 40 °C, 1 mL/min, 90:10 *n*-hexane:isopropanol, 214 nm, t = 12.682 min and 15.002 min).

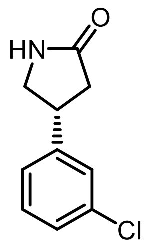

(*R*)-4-(3-Chlorophenyl)pyrrolidin-2-one [2i]

Following **GP-D** using 3-(3-chlorophenyl)cyclobutan-1-one **[1i]** (36.1 mg, 200 µmol, 1.00 eq.) the product (27.8 mg, 142 µmol, 76%) was obtained via flash column chromatography (SiO_2, EtOAc:MeOH, 100:0 to 95:5, stained with $KMnO_4$) as a colorless solid.

M.P.: 97 – 99 °C. **IR (neat)**: ṽ = 3244 (m), 2871 (w), 1692 (s), 1661 (s), 1598 (w), 1573 (w), 1477 (w), 1421 (w), 1343 (w), 1263 (m), 1084 (w), 781 (s), 724 (m). **^{1}H NMR (400 MHz, $CDCl_3$)**: δ (ppm) = 7.31 – 7.22 (m, 3H), 7.17 – 7.10 (m, 1H), 6.48 (s, 1H), 3.84 – 3.75 (m, 1H), 3.73 – 3.61 (m, 1H), 3.41 (dd, *J* = 9.5, 7.0 Hz, 1H), 2.74 (dd, *J* = 16.9, 8.9 Hz, 1H), 2.47 (dd, *J* = 16.9, 8.5 Hz, 1H). **^{13}C NMR (101 MHz, $CDCl_3$)**: δ (ppm) = 177.3, 144.2, 134.7, 130.2, 127.4, 127.1, 125.0, 49.2, 39.9, 37.7. **HRMS (ESI)**: calculated for $C_{10}H_{11}ClNO$ $[M+H]^+$: 196.0524, Found: 196.0513. **Optical Rotation**: $[\alpha]_D^{25}$ = –30.9 (c = 0.5, $CHCl_3$) for an enantiomerically enriched sample of 96:04 *er*. The enantiomeric purity was established by HPLC analysis using a chiral column (Lux® *i*Amylose-3, 40 °C, 1 mL/min, 90:10 *n*-hexane:isopropanol, 214 nm, t = 13.671 min and 14.927 min).

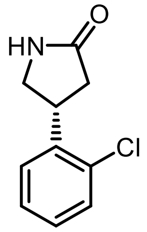

(*R*)-4-(2-Chlorophenyl)pyrrolidin-2-one [2j]

Following **GP-D** using 3-(2-chlorophenyl)cyclobutan-1-one **[1j]** (36.1 mg, 200 µmol, 1.00 eq.) the product (22.3 mg, 114 µmol, 58%) was obtained via flash column chromatography (SiO_2, EtOAc:MeOH, 100:0 to 95:5, stained with $KMnO_4$) as a colorless solid.

^{1}H NMR (400 MHz, $CDCl_3$): δ (ppm) = 7.40 (dd, *J* = 7.8, 1.5 Hz, 1H), 7.34 (dd, *J* = 7.7, 1.8 Hz, 1H), 7.30 – 7.24 (m, 1H), 7.21 (td, *J* = 7.6, 1.8 Hz, 1H), 6.07 (s, 1H), 4.16 (dtd, *J* = 9.0, 7.6, 6.0 Hz, 1H), 3.85 (ddd, *J* = 9.7, 8.0, 0.9 Hz, 1H), 3.40 (ddd, *J* = 9.7, 6.0, 0.7 Hz, 1H), 2.77 (dd, *J* = 17.0, 9.1 Hz, 1H), 2.52 (dd, *J* = 17.0, 7.3 Hz, 1H). **^{13}C NMR (101 MHz, $CDCl_3$)**: δ (ppm) = 177.4, 139.5, 133.9, 130.1, 128.5, 127.5, 127.3, 48.2, 36.9, 36.6. The spectroscopic data were in agreement to those previously reported.[15] **Optical Rotation**: $[\alpha]_D^{25}$ = –45.9 (c = 0.5, $CHCl_3$) for an enantiomerically enriched sample of 96:04 *er*. The enantiomeric purity was established by HPLC analysis using a chiral column (Lux® *i*Amylose-3, 40 °C, 1 mL/min, 90:10 *n*-hexane:isopropanol, 214 nm, t = 14.306 min and 15.246 min).

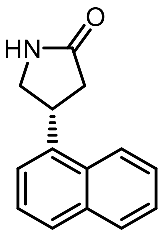

(*R*)-4-(Naphthalen-1-yl)pyrrolidin-2-one [2k]

Following **GP-D** using 3-(naphthalen-1-yl)cyclobutan-1-one **[1k]** (39.3 mg, 200 µmol, 1.00 eq.) the product (35.1 mg, 166 µmol, 87%) was obtained via flash column chromatography (SiO_2, EtOAc:MeOH, 100:0 to 95:5, stained with $KMnO_4$) as a colorless solid.

IR (neat): ṽ = 3218 (w), 1692 (m), 1509 (w), 1272 (w), 779 (w), 499 (w), 431 (w). **^{1}H NMR (400 MHz, $CDCl_3$)**: δ (ppm) = 8.05 – 7.98 (m, 1H), 7.93 – 7.87 (m, 1H), 7.79 (dd, *J* = 5.6, 3.8 Hz, 1H), 7.59 – 7.49 (m, 2H), 7.48 – 7.43 (m, 2H), 6.87 (s, 1H), 4.46 (dtd, *J* = 8.9, 7.6, 6.1 Hz, 1H), 3.95 (ddd, *J* = 9.9, 8.0, 0.9 Hz, 1H), 3.56 (dd, *J* = 9.8, 6.1 Hz, 1H), 2.89 (dd, *J* = 16.9, 9.0 Hz, 1H), 2.70 (dd, *J* = 16.9, 7.3 Hz, 1H). **^{13}C NMR (101 MHz, $CDCl_3$)**: δ (ppm) = 177.9, 137.7, 134.1, 131.3, 129.2, 127.8, 126.4, 125.8, 125.6, 122.9, 122.8, 48.9, 37.3, 35.9. The spectroscopic data were in agreement to those previously reported.[19] **HRMS (ESI)**: calculated for $C_{14}H_{14}NO$ $[M+H]^+$: 212.1070, Found: 212.1067. **Optical Rotation**: $[\alpha]_D^{25}$ = –52.2 (c = 0.5, $CHCl_3$) for an enantiomerically enriched sample of 97:03 *er*. The enantiomeric purity was established by HPLC analysis using a chiral column (Lux® Amylose-1, 40 °C, 1 mL/min, 90:10 *n*-hexane:isopropanol, 214 nm, t = 15.697 min and 18.837 min).

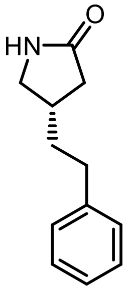

(*S*)-4-Phenethylpyrrolidin-2-one [2l]

Following **GP-C** using 3-phenethylcyclobutanone **[1l]** (34.9 mg, 200 µmol, 1.00 eq.) the product (29.5 mg, 156 µmol, 78%) was obtained via flash column chromatography (SiO_2, EtOAc:MeOH, 100:0 to 95:5, stained with $KMnO_4$) as a colorless solid.

^{1}H NMR (400 MHz, $CDCl_3$): δ (ppm) = 7.35 – 7.27 (m, 2H), 7.27 – 7.14 (m, 3H), 6.41 (s, 1H), 3.56 – 3.46 (m, 1H), 3.11 – 3.03 (m, 1H), 2.72 – 2.59 (m, 2H), 2.53 – 2.41 (m, 2H), 2.11 – 2.02 (m, 1H), 1.88 – 1.78 (m, 2H). **^{13}C NMR (101 MHz, $CDCl_3$)**: δ (ppm) = 177.9, 142.2, 138.7, 128.9, 128.0, 127.7, 123.9, 49.7, 40.4, 38.1, 21.6. The spectroscopic data were in agreement to those previously reported.[20] **Optical Rotation**: $[\alpha]_D^{25}$ = 6.4 (c = 0.513, $CHCl_3$) for an enantiomerically enriched sample of 84:16 *er*. The enantiomeric purity was established by HPLC analysis using

a chiral column (Lux® Amylose-1, 40 °C, 1 mL/min, 90:10 *n*-hexane:isopropanol, 214 nm, t = 10.566 min and 11.168 min).

Following **GP-D** using 3-phenethylcyclobutanone **[1l]** (34.9 mg, 200 µmol, 1.00 eq.) the product (27.6 mg, 146 µmol, 74%) was obtained via flash column chromatography (SiO_2, EtOAc:MeOH, 100:0 to 95:5, stained with $KMnO_4$) as a colorless solid.

Optical Rotation: $[\alpha]_D^{25}$ = 3.7 (c = 0.5, $CHCl_3$) for an enantiomerically enriched sample of 92:08 *er*. The enantiomeric purity was established by HPLC analysis using a chiral column (Lux® Amylose-1, 40 °C, 1 mL/min, 90:10 *n*-hexane:isopropanol, 214 nm, t = 10.566 min and 11.168 min).

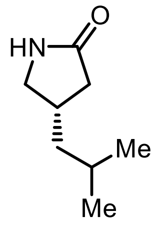

(*S*)-4-Isobutylpyrrolidin-2-one [2m]

Following **GP-D** using 3-isobutylcyclobutan-1-one **[1m]** (25.4 mg, 200 µmol, 1.00 eq.) the product (13.8 mg, 98 µmol, 52%) was obtained via flash column chromatography (SiO_2, EtOAc:MeOH, 100:0 to 95:5, stained with $KMnO_4$) as a colorless liquid.

^{1}H NMR (400 MHz, $CDCl_3$): δ (ppm) = 6.29 (br s, 1H), 3.47 (dd, *J* = 9.4, 7.8 Hz, 1H), 2.99 (dd, *J* = 9.5, 7.1 Hz, 1H), 2.62 – 2.46 (m, 1H), 2.41 (dd, *J* = 16.6, 8.6 Hz, 1H), 1.98 (dd, *J* = 16.6, 8.4 Hz, 1H), 1.64 – 1.49 (m, 1H), 1.34 (app. td, *J* = 7.4, 1.9 Hz, 2H), 0.90 (d, *J* = 4.3 Hz, 3H), 0.89 (d, *J* = 4.3 Hz, 3H). **^{13}C NMR (101 MHz, $CDCl_3$)**: δ (ppm) = 178.7, 48.4, 44.0, 37.1, 33.1, 26.3, 22.8, 22.6. The spectroscopic data were in agreement to those previously reported.[2] **Optical Rotation**: $[\alpha]_D^{25}$ = –0.5 (c = 0.5, $CHCl_3$) for an enantiomerically enriched sample of 94:06 *er*. The enantiomeric purity was established by HPLC analysis using a chiral column (Lux® *i*Amylose-3, 40 °C, 1 mL/min, 95:05 *n*-hexane:isopropanol, 214 nm, t = 16.946 min and 18.114 min).

Procedure for cyclopentanones:

The racemic δ-oxime esters **[rac-6n]** were prepared according to a slightly modified procedure reported in the literature.[10]

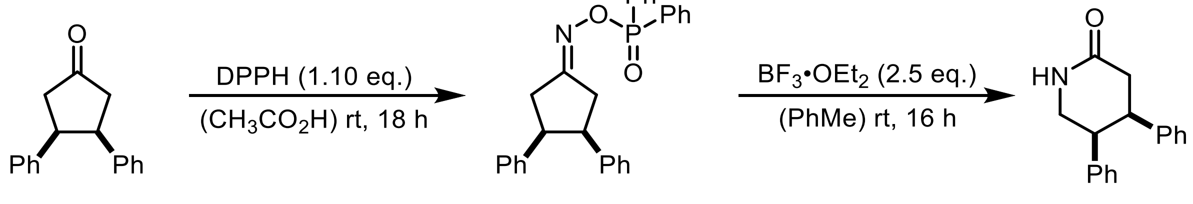

In a round bottom flask, the corresponding cyclopentanone (500 µmol, 1.00 eq.) was dissolved in CH_3CO_2H (0.05 M), then *O*-(diphenylphosphinyl)hydroxylamine (550 µmol, 1.10 eq.) was

added to the solution and the reaction mixture was stirred at room temperature for 18 h. After 18 h, the reaction mixture was quenched with an aqueous saturated $NaHCO_3$ solution(5 mL), then diluted with water (15 mL). The reaction mixture was extracted with CH_2Cl_2 (3 x 20 mL) and the combined organic layers were dried over Na_2SO_4. The solvent was removed under reduced pressure and the crude product was purified *via* automated flash column chromatography (SiO_2, *n*-hexane:EtOAc, 50:50 to 30:70).

In a round bottom flask, the corresponding oxime ester (100 µmol, 1.00 eq.) was dissolved in toluene (0.05 M) then boron trifluoride etherate (250 µmol, 2.50 eq.) was added and stirred at room temperature for 16 h. After 16 h, the reaction mixture was quenched with an aqueous saturated $NaHCO_3$ solution (1 mL) then dilute with water (10 mL). The reaction mixture was extracted with chloroform:isopropanol mixture (1:3, 3 x 10 mL) and the combined organic layers were dried over Na_2SO_4. The solvent was removed under reduced pressure and the crude product was purified *via* automated flash column chromatography (SiO_2, EtOAc:MeOH, 100:0 to 90:10).

General procedure E (GP-E) for the synthesis of the δ-lactams:

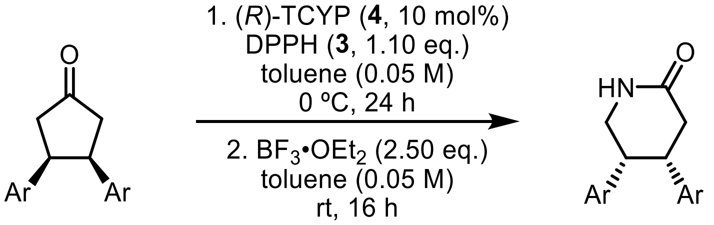

1: In a round bottom flask, (*R*)-TCYP (19.9 mg, 0.02 mmol, 10 mol%) and the corresponding cyclopentanone (0.20 mmol, 1.00 eq.) were dissolved in toluene (0.05 M). The mixture was cooled to 0 °C. *O*-Diphenylphosphinylhydroxylamine (51.3 mg, 0.22 mmol, 1.10 eq.) was added and the reaction mixture was stirred at 0 °C for 24 h. After 24 h, the reaction mixture was quenched with NEt_3 (27.9 mL, 20.2 mg, 0.20 µmol, 1.00 eq.). The solvent was removed under reduced pressure and the corresponding oxime ester was purified via column chromatography (SiO_2, *n*-hexane:EtOAc 100:0 to 0:100).

2: In a round bottom flask, the corresponding purified oxime ester was dissolved in toluene (0.05 M), then boron trifluoride etherate (250 µmol, 2.50 eq.) was added and the reaction mixture was stirred at room temperature for 16 h. After 16 h, the reaction mixture was quenched with an aqueous saturated $NaHCO_3$ solution (1 mL) and then diluted with water (10 mL). The reaction mixture was extracted with chloroform:isopropanol mixture (1:3, 3 x 10 mL) and the combined organic layers were dried over Na_2SO_4. The solvent was removed under reduced pressure and the corresponding δ-lactam was obtained *via* flash column chromatography (SiO_2, EtOAc:MeOH, 100:0 to 90:10 stained with $KMnO_4$).

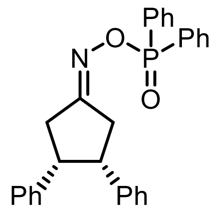

((((3*R*,4*S*,*E*)-3,4-Diphenylcyclopentylidene)amino)oxy)diphenylphosphine oxide [6n]

Following **GP-E-1** using 3,4-diphenylcyclopentan-1-one **[1n]** (47.2 mg, 200 µmol, 1.00 eq.) the product (86.6 mg, 192 µmol, 96%) was obtained via flash column chromatography (SiO_2, EtOAc:MeOH, 50:50 to 30:70, stained with $KMnO_4$) as a colorless solid.

M.P.: 123-128 °C. **IR (neat)**: ṽ = 3736 (s), 3647 (s), 3060 (br), 2363 (s), 2335 (br), 2184 (m), 2144 (s), 1994 (m), 1437 (s), 1233 (br), 1130 (m), 893 (m), 696 (s), 554 (s). **^{1}H NMR (400 MHz, CDCl$_3$)**: δ (ppm) = 8.02 – 7.84 (m, 4H), 7.65 – 7.40 (m, 6H), 7.09 (td, *J* = 7.1, 3.4 Hz, 6H), 6.71 (ddd, *J* = 11.4, 7.2, 2.0 Hz, 4H), 3.68 (h, *J* = 6.9 Hz, 2H), 3.29 – 3.11 (m, 2H), 2.97 (qd, *J* = 17.7, 6.7 Hz, 2H). **^{13}C NMR (101 MHz, CDCl$_3$)**: δ (ppm) = 175.3 (d, *J* = 11.3 Hz), 139.2 (d, *J* = 11.3 Hz), 132.4 (d, *J* = 3.1 Hz), 132.1 (dd, *J* = 16.0, 9.9 Hz), 130.9 (d, *J* = 136.2 Hz), 128.6 (dd, *J* = 13.2, 2.6 Hz), 128.1 (d, *J* = 7.7 Hz), 127.9, 126.7 (d, *J* = 5.8 Hz), 48.9, 48.5, 35.4, 34.3. **^{31}P NMR (162 MHz, CDCl$_3$)**: δ (ppm) = 34.77. The spectroscopic data were in agreement to those previously reported.[21] **HRMS (ESI)**: Calculated for $C_{29}H_{27}NO_2P$ $[M+H]^+$: 452.1779, Found: 452.1775. **Optical Rotation**: $[\alpha]_D^{25}$ = –73.5 (c = 0.5, $CHCl_3$) for an enantiomerically enriched sample of 98:02 *er*. The enantiomeric purity was established by HPLC analysis using a chiral column (Lux® Amylose-1, 40 °C, 1 mL/min, 85:15 *n*-hexane:isopropanol, 214 nm, t = 25.178 min and 28.414 min).

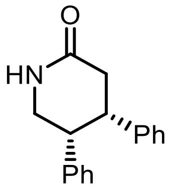

(4*S*,5*R*)-4,5-Diphenylpiperidin-2-one [2n]

Following **GP-E-2** using ((((3*R*,4*S*,*E*)-3,4-diphenylcyclopentylidene)amino)oxy)diphenyl-phosphine oxide **[6n]** (45.1 mg, 100 µmol, 1.00 eq.) the product (20.9 mg, 83.2 µmol, 83%) was obtained via flash column chromatography (SiO_2, EtOAc:MeOH, 100:0 to 90:10, stained with $KMnO_4$) as a colorless solid.

^{1}H NMR (400 MHz, CDCl$_3$): δ (ppm) = 7.27 – 7.16 (m, 6H), 6.85 – 6.76 (m, 4H), 3.68 (dt, *J* = 7.9, 2.1 Hz, 2H), 3.49 (dt, *J* = 7.9, 4.6 Hz, 2H), 2.88 (dd, *J* = 17.9, 6.0 Hz, 1H), 2.75 (dd, *J* = 17.9, 6.0 Hz, 1H). **^{13}C NMR (101 MHz, CDCl$_3$)**: δ (ppm) = 172.4, 139.6, 139.1, 128.4, 128.2, 128.2, 128.1, 127.1, 127.0, 43.9, 43.3, 43.2, 35.3. The spectroscopic data were in agreement to those previously reported.[14] **Optical Rotation**: $[\alpha]_D^{25}$ = +6.7 (c = 1.0, $CHCl_3$) for an enantiomerically enriched sample of 97:03 *er*. The enantiomeric purity was established by HPLC analysis using

a chiral column (Lux® Amylose-1, 40 °C, 1 mL/min, 80:20 *n*-hexane:isopropanol, 214 nm, t = 8.046 min and 9.131 min).

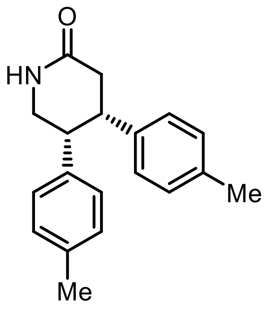

(4*S*,5*R*)-4,5-Di-*p*-tolylpiperidin-2-one [2o]

Following **GP-E** using 3,4-di-*p*-tolylcyclopentan-1-one **[S4o]** (50.0 mg, 189 µmol, 1.00 eq.) the product (24.8 mg, 130 µmol, 47%) was obtained via flash column chromatography (SiO_2, EtOAc:MeOH, 100:0 to 95:5, stained with $KMnO_4$) as a colorless solid.

M.P.: 164-166 °C. **IR (neat)**: ṽ = 2922 (w), 1662 (s), 1515 (m), 1495 (m), 1455 (w), 1408 (w), 1369 (w), 1330 (w), 1247 (w), 1127 (w), 822 (m), 736 (w), 520 (w), 486 (w), 440 (w). **^{1}H NMR (400 MHz, $CDCl_3$)**: δ (ppm) = 7.00 (dd, *J* = 7.9, 5.1 Hz, 4H), 6.87 (s, 1H), 6.68 (dd, *J* = 8.2, 2.9 Hz, 4H), 3.64 – 3.57 (m, 2H), 3.50 – 3.36 (m, 2H), 2.82 (dd, *J* = 17.8, 5.9 Hz, 1H), 2.69 (dd, *J* = 17.9, 6.0 Hz, 1H), 2.30 (*app.* d, *J* = 2.6 Hz, 6H). **^{13}C NMR (101 MHz, $CDCl_3$)**: δ (ppm) = 172.6, 136.7, 136.7, 136.6, 136.2, 128.8, 128.8, 128.4, 128.3, 44.2, 43.1, 42.8, 35.6, 21.1 (two overlapping C). **HRMS (ESI)**: calculated for $C_{19}H_{22}NO$ $[M+H]^+$: 280.1696, Found: 280.1697. **Optical Rotation**: $[\alpha]_D^{25}$ = +19.3 (c = 1.0, $CHCl_3$) for an enantiomerically enriched sample of 95:05 *er*. The enantiomeric purity was established by HPLC analysis using a chiral column (Lux® Amylose-1, 40 °C, 1 mL/min, 80:20 *n*-hexane:isopropanol, 214 nm, t = 6.331 min and 7.332 min).

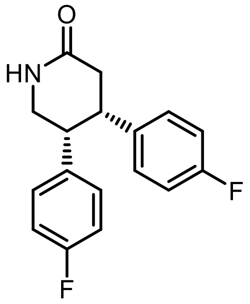

(4*S*,5*R*)-4,5-Bis(4-fluorophenyl)piperidin-2-one [2p]

Following **GP-E** using 3,4-bis(4-fluorophenyl)cyclopentan-1-one **[S4p]** (54.5 mg, 200 µmol, 1.00 eq.) the product (23.6 mg, 82 µmol, 55%) was obtained via flash column chromatography (SiO_2, EtOAc:MeOH, 100:0 to 95:5, stained with $KMnO_4$) as a colorless solid.

M.P.: 190-192 °C. **IR (neat)**: ṽ = 3053 (w), 2915 (w), 2037 (w), 2029 (w), 1663 (s), 1606 (m), 1510 (s), 1421 (w), 1332 (w), 1265 (m), 1223 (m), 1159 (m), 1102 (w), 838 (s), 811 (m), 733 (s), 704 (m), 665 (w), 530 (m), 481 (m), 456 (m), 420 (m). **^{1}H NMR (400 MHz, $CDCl_3$)**: δ (ppm) = 7.09 (s, 1H), 6.98 – 6.84 (m, 4H), 6.81 – 6.71 (m, 4H), 3.71 – 3.62 (m, 2H), 3.54 – 3.40 (m, 2H),

2.85 (dd, J = 17.9, 6.0 Hz, 1H), 2.68 (dd, J = 17.9, 6.2 Hz, 1H). **^{13}C NMR (101 MHz, CDCl$_3$)**: δ (ppm) = 172.1, 162.0 (d, J = 246 Hz, 2 overlapping C), 135.0 (d, J = 56 Hz), 134.9 (d, J = 56 Hz), 129.9 (d, J = 8.0 Hz), 129.8 (d, J = 7.8 Hz), 115.3 (d, J = 2.4 Hz), 115.1 (d, J = 2.6 Hz), 44.1, 42.7, 42.6, 35.3. **^{19}F NMR (377 MHz, CDCl$_3$)**: δ (ppm) = -116.21 – 116.28 (m, 1F), -116.42 – 116.49 (m, 1F). The spectroscopic data were in agreement to those previously reported.[9] **HRMS (ESI)**: calculated for $C_{17}H_{16}F_2NO$ [M+H]$^+$: 288.1194, Found: 288.1195. **Optical Rotation**: $[\alpha]_D^{25}$ = +5.2 (c = 0.5, CHCl$_3$) for an enantiomerically enriched sample of 96:04 *er*. The enantiomeric purity was established by HPLC analysis using a chiral column (Lux® Amylose-1, 40 °C, 1 mL/min, 80:20 *n*-hexane:isopropanol, 214 nm, t = 7.288 min and 9.608 min).

Procedure for cyclohexanones:

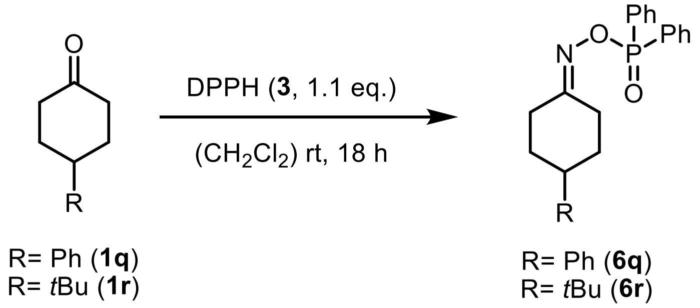

To obtain the racemic samples, 4-phenylcyclohexan-1-one (**1q**, 1.00 eq.) or 4-(tert-butyl)cyclohexan-1-one (**1r**, 1.00 eq.) was dissolved in CH_2Cl_2 (0.05 M), then *O*-(diphenylphosphinyl)hydroxylamine (1.10 eq.) was added to the solution and the reaction mixture was stirred at room temperature for 18 h. The solvent was removed under reduced pressure and the oxime ester was obtained *via* automated flash column chromatography (SiO_2, *n*-hexane:EtOAc, 50:50 to 30:70) as a colorless solid (**6q**) or colorless oil (**6r**).

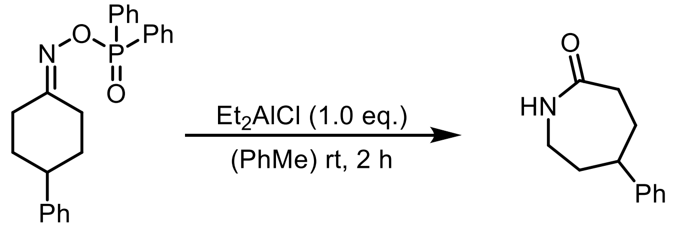

In a round bottom flask, diphenyl(((4-phenylcyclohexylidene)amino)oxy)phosphine oxide (77.9 mg, 0.20 mmol, 1.00 eq.) was dissolved in toluene (0.05 M). Diethyl aluminium chloride solution (1.00 M in toluene, 0.20 mL, 2.02 mmol, 1.01 eq.) was added to the solution and the reaction mixture was stirred at room temperature for 2 h. The reaction mixture was quenched with an aqueous saturated $NaHCO_3$ solution (5 mL), then diluted with water (15 mL). The reaction mixture was extracted with a chloroform:isopropanol mixture (1:3, 3 x 20 mL) and the combined organic layers were dried over Na_2SO_4. The solvent was removed under reduced

pressure and the product (29.2 mg, 0.15 mmol, 77%) was obtained *via* automated flash column chromatography (SiO_2, EtOAc:MeOH, 100:0 to 90:10) as a colorless solid.

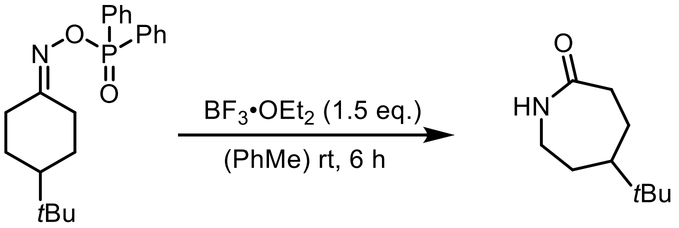

In a round bottom flask, (((4-(tert-butyl)cyclohexylidene)amino)oxy)diphenylphosphine oxide (1.0 eq.) was dissolved in toluene (0.05 M). Boron trifluoride etherate (1.50 eq.) was added to the solution and the reaction mixture was stirred at room temperature for 6 h. The reaction mixture was quenched with an aqueous saturated $NaHCO_3$ solution (5 mL), then diluted with water (15 mL). The reaction mixture was extracted with a chloroform:isopropanol mixture (1:3, 3 x 20 mL) and the combined organic layers were dried over Na_2SO_4. The solvent was removed under reduced pressure and the product was obtained *via* automated flash column chromatography (SiO_2, EtOAc:MeOH, 100:0 to 90:10) as a colorless solid.

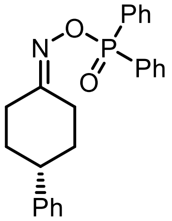

(*R*,*Z*)-Diphenyl(((4-phenylcyclohexylidene)amino)oxy)phosphine oxide [6q]

In a round bottom flask, (*R*)-TCYP (19.9 mg, 0.02 mmol, 0.10 eq.) and 4-phenylcyclohexan-1-one **[1q]** (34.9 mg, 0.20 mmol, 1.00 eq.) were dissolved in toluene (0.05 M). The mixture was cooled to –78 °C. *O*-(Diphenylphosphinyl)hydroxylamine (51.3 mg, 0.22 mmol, 1.10 eq.) was added and the reaction mixture was stirred at –78 °C for 24 h. The reaction mixture was quenched with NEt_3 (27.9 mL, 0.20 µmol, 1.00 eq.). The solvent was removed under reduced pressure and the oxime ester (71.1 mg, 0.18 mmol, 91%) was obtained *via* automated flash column chromatography (SiO_2, *n*-hexane: EtOAc, 50:50 to 30:70) as a colorless solid.

M.P.: 169-171 °C. **IR (neat)**: ṽ = 2926 (br), 2361 (s), 2337 (s), 1438 (m), 1233 (m), 1130 (m), 885 (s), 729(s). **^{1}H NMR (400 MHz, $CDCl_3$)**: δ (ppm) = 7.94 – 7.82 (m, 4H), 7.54 (m, 2H), 7.47 (m, 4H), 7.35 – 7.27 (m, 2H), 7.24 – 7.16 (m, 3H), 3.72 – 3.45 (m, 1H), 2.79 (tt, *J* = 12.3, 3.3 Hz, 1H), 2.64 (ddt, *J* = 14.2, 4.4, 2.4 Hz, 1H), 2.26 (td, *J* = 13.8, 4.8 Hz, 1H), 2.07 (m, 3H), 1.79 – 1.60 (m, 2H). **^{13}C NMR (101 MHz, $CDCl_3$)**: δ (ppm) = 168.5 (d, *J* = 11.1 Hz), 145.2, 132.3 (t, *J* = 2.7 Hz), 132.2 (d, *J* = 9.9 Hz), 131.1 (d, *J* = 136.9 Hz), 128.7, 128.6 (d, *J* = 13.2 Hz), 126.8, 126.7, 43.5, 33.8, 32.9, 31.9, 26.4. **^{31}P NMR (162 MHz, $CDCl_3$)**: δ (ppm) = 34.75. **HRMS (ESI)**: calculated for $C_{24}H_{25}NO_2P$ $[M+H]^+$: 390.1618, Found: 390.1610. **Optical Rotation**: $[\alpha]_D^{25}$ = –16.1 (c = 1.0, $CHCl_3$) for an enantiomerically enriched sample of 80:20 *er*. The enantiomeric purity was

established by HPLC analysis using a chiral column (ReproSil Chiral-AMS, 40 °C, 1 mL/min, 85:15 *n*-hexane:isopropanol, 214 nm, t = 16.655 min and 20.982 min).

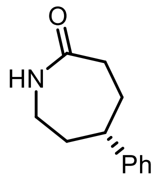

(*S*)-5-Phenylazepan-2-one [2q]

In a round bottom flask, diphenyl(((4-phenylcyclohexylidene)amino)oxy)phosphine oxide **[6q]** (70.0 mg, 0.18 mmol, 1.00 eq.) was dissolved in toluene (0.05 M), then Et_2AlCl (0.20 mL, 1.00 M, 1.11 mmol, 1.11 eq.) was added to the solution and the reaction mixture was stirred at 0 °C for 4 h. The reaction mixture was quenched with an aqueous saturated $NaHCO_3$ solution (5 mL) and diluted with water (15 mL). The reaction mixture was extracted with a chloroform:isopropanol mixture (1:3, 3 x 20 mL) and the combined organic layers were dried over Na_2SO_4. The solvent was removed under reduced pressure and the product (29.6 mg, 0.16 mmol, 87%). was obtained *via* automated flash column chromatography (SiO_2, EtOAc:MeOH, 100:0 to 90:10) as a colorless solid.

^{1}H NMR (400 MHz, $CDCl_3$): δ (ppm) = 7.31 (dd, *J* = 8.1, 6.8 Hz, 2H), 7.24 – 7.15 (m, 3H), 6.53 (s, 1H), 3.52 – 3.22 (m, 2H), 2.76 (tt, *J* = 12.1, 3.5 Hz, 1H), 2.69 – 2.50 (m, 2H), 2.08 – 1.93 (m, 2H), 1.87 – 1.65 (m, 2H). **^{13}C NMR (101 MHz, $CDCl_3$)**: δ (ppm) = 178.7, 146.4, 128.8, 126.8, 126.7, 49.0, 42.3, 37.5, 36.0, 30.6. The spectroscopic data were in agreement to those previously reported.[22] **Optical Rotation**: $[\alpha]_D^{25}$ = –8.7 (c = 0.5, $CHCl_3$) for an enantiomerically enriched sample of 77:23 *er*. The enantiomeric purity was established by HPLC analysis using a chiral column (ReproSil Chiral-AMS, 40 °C, 1 mL/min, 85:15 *n*-hexane:isopropanol, 214 nm, t = 7.291 min and 15.634 min).

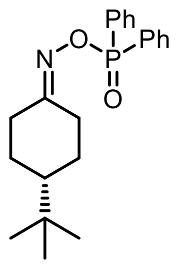

(*R*,*Z*)-(((4-(*tert*-Butyl)cyclohexylidene)amino)oxy)diphenylphosphine oxide [6r]

In a round bottom flask, (*R*)-TCYP (19.9 mg, 0.02 mmol, 0.10 eq.) and 4-(*tert*-butyl)cyclohexan-1-one **[1r]** (30.9 mg, 0.20 mmol, 1.00 eq.) were dissolved in toluene (0.05 M). The mixture was cooled to –20 °C. *O*-(Diphenylphosphinyl)hydroxylamine (51.3 mg, 0.22 mmol, 1.10 eq.) was added and the reaction mixture was stirred at –20 °C for 24 h. The reaction mixture was quenched with NEt_3 (27.9 mL, 0.20 µmol, 1.00 eq.). The solvent was removed under reduced pressure and the oxime ester (60.4 mg, 0.16 mmol, 82%) was obtained *via* flash column chromatography (SiO_2, *n*-hexane: EtOAc, 50:50) as a colorless oil.

IR (neat): ṽ = 2956 (s), 2905 (w), 2867 (m), 2354 (w), 1640 (w), 1439 (s), 1366 (m), 1236 (s), 1128 (s), 1112 (s), 888 (s), 728 (s), 696 (s). **^{1}H NMR (400 MHz, $CDCl_3$)**: δ (ppm) = 7.84 (dddd, *J* = 12.2, 8.4, 3.8, 1.4 Hz, 4H), 7.51 (ddq, *J* = 8.7, 3.3, 1.6 Hz, 2H), 7.47 – 7.40 (m, 4H), 3.46 (ddt, *J* = 14.7, 4.8, 2.4 Hz, 1H), 2.61 – 2.45 (m, 1H), 2.10 – 2.00 (m, 1H), 1.99 – 1.80 (m, 3H), 1.30 – 1.11 (m, 3H), 0.85 (s, 9H). **^{13}C NMR (101 MHz, $CDCl_3$)**: δ (ppm) = 169.68 (d, *J* = 11.2 Hz), 132.26 – 132.12 (m), 132.06 (d, *J* = 1.7 Hz), 131.11 (d, *J* = 136.5 Hz), 128.51 (d, *J* = 13.1 Hz), 47.28, 32.55, 31.77, 27.60, 27.44, 26.53, 26.41. **^{31}P NMR (162 MHz, $CDCl_3$)**: δ (ppm) = 34.45. **HRMS (ESI)**: calculated for $C_{22}H_{29}NO_2P$ $[M+H]^+$: 370.1931, found: 370.1927. **Optical Rotation**: $[\alpha]_D^{25}$ = –30.0 (c = 0.5, $CHCl_3$) for an enantiomerically enriched sample of 87:13 *er*. The enantiomeric purity was established by HPLC analysis using a chiral column (Cellulose-1, 40 °C, 1 mL/min, 90:10 *n*-hexane:isopropanol, 214 nm, t = 7.855 min and 8.499 min).

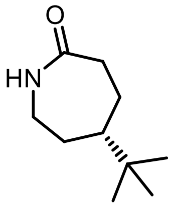

(*S*)-5-(*tert*-Butyl)azepan-2-one [2r]

In a round bottom flask, (*R*,*Z*)-(((4-(*tert*-butyl)cyclohexylidene)amino)oxy)diphenylphosphine oxide **[6r]** (59.0 mg, 0.16 mmol, 1.00 eq.) was dissolved in toluene (0.05 M), then boron trifluoride etherate (1.50 eq.) was added to the solution and the reaction mixture was stirred at 0 °C for 6 h. The reaction mixture was quenched with an aqueous saturated $NaHCO_3$ solution (5 mL) and diluted with water (15 mL). The reaction mixture was extracted with dichloromethane (3 x 20 mL) and a chloroform:isopropanol mixture (3:1, 1 x 20 mL). The combined organic layers were dried over Na_2SO_4, the solvent was removed under reduced pressure and the product (20.7 mg, 0.12 mmol, 77%). was obtained *via* automated flash column chromatography (SiO_2, EtOAc:MeOH, 100:0 to 90:10) as a colorless solid.

^{1}H NMR (400 MHz, $CDCl_3$): δ (ppm) = 6.97 (s, 1H), 3.38 – 3.01 (m, 2H), 2.56 – 2.26 (m, 2H), 1.98 – 1.84 (m, 2H), 1.32 – 1.07 (m, 3H), 0.84 (s, 9H). **^{13}C NMR (101 MHz, $CDCl_3$)**: δ (ppm) = 179.4, 52.4, 42.3, 35.8, 33.3, 30.7, 27.7, 24.0. The spectroscopic data were in agreement to those previously reported.[22] **Optical Rotation**: $[\alpha]_D^{25}$ = –12.0 (c = 0.5, $CHCl_3$) for an enantiomerically enriched sample of 87:13 *er*. For the HPLC analysis a benzyl protection was carried out according to literature to install a chromophore visible at 214 nm.[22] The enantiomeric purity of the *N*-benzyl protected lactam was established by HPLC analysis using a chiral column (ReproSil Chiral-AMS, 40 °C, 1 mL/min, 85:15 *n*-hexane:isopropanol, 214 nm, t = 9.909 min and 19.716 min).

5.1. Limitations

We also tried the condensation reaction for meso cyclohexanones such as cis-2,6-diphenyl cyclohexanone according to the procedure used for cyclohexanone **1q**.

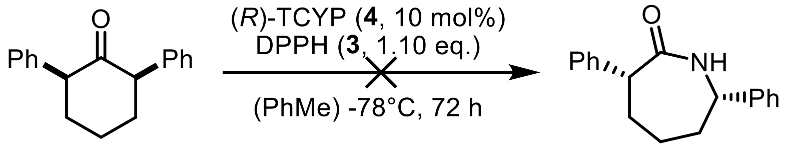

Unfortunately, even after 72 hours instead of 24 hours no conversion could be detected. We attribute this to the steric demand of this substrate which prevents the coordination to the chiral catalyst.

6. Applications

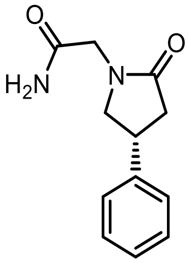

(*R*)-Phenotropil [10]

In a Schlenk flask, (*R*)-4-phenylpyrrolidin-2-one **[2a]** (64.5 mg, 400 µmol, 1.00 eq.) was dissolved in THF and the reaction mixture was cooled down to -78 °C. LiHMDS (600 µL, 600 µmol, 1.00 M in toluene, 1.50 eq.) was added and the reaction mixture was stirred at -78 °C for 1.0 h. 2-Bromoacetamide (67.6 mg, 480 µmol, 1.20 eq.) was added and the reaction mixture was left overnight to warm to room temperature. The mixture was filtered through a pad of silica and eluted with EtOAc:MeOH (90:10) and the solvent was removed under reduced pressure. The product (27.2 mg, 0.12 mmol, 31%) was obtained *via* column chromatography (SiO_2, EtOAc:MeOH, 100:0 to 90:10, stained with $KMnO_4$) as a colorless oil.

^{1}H NMR (400 MHz, $CDCl_3$): δ (ppm) = 7.38 – 7.31 (m, 2H), 7.30 – 7.22 (m, 3H), 6.33 (s, 1H), 5.82 (s, 1H), 4.08 – 3.94 (m, 2H), 3.89 (dd, *J* = 9.4, 8.1 Hz, 1H), 3.71 – 3.61 (m, 1H), 3.56 (dd, *J* = 9.3, 7.2 Hz, 1H), 2.87 (dd, *J* = 17.0, 9.0 Hz, 1H), 2.62 (dd, *J* = 17.0, 8.4 Hz, 1H). **^{13}C NMR (101 MHz, $CDCl_3$)**: δ (ppm) = 178.2, 173.7, 145.0, 132.2, 130.5, 130.0, 58.7, 49.7, 41.6, 40.6. The spectroscopic data were in agreement to those previously reported.[23] **Optical Rotation**: $[\alpha]_D^{25}$ = –1.4 (c = 1.0, $CHCl_3$) for an enantiomerically enriched sample of 93:07 *er*. The enantiomeric purity was established by HPLC analysis using a chiral column (Lux® Amylose-1, 40 °C, 1 mL/min, 95:05 to 90:10 *n*-hexane:isopropanol, 214 nm, t = 65.021 min and 66.463 min).

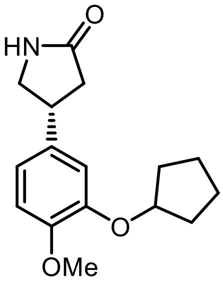

(*R*)-Rolipram [12]

Following **GP-D** using 3-(3-(cyclopentyloxy)-4-methoxyphenyl)cyclobutan-1-one **[1s]** (39.1 mg, 150 µmol, 1.00 eq.) the product (18.2 mg, 66.1 µmol, 43%) was obtained via flash column chromatography (SiO_2, EtOAc:MeOH, 100:0 to 95:5, stained with $KMnO_4$) as a colorless solid.

^{1}H NMR (400 MHz, $CDCl_3$): δ (ppm) = 6.85 – 6.80 (m, 1H), 6.80 – 6.73 (m, 2H), 4.76 (tt, *J* = 6.2, 3.5 Hz, 1H), 3.85 – 3.80 (m, 3H), 3.80 – 3.70 (m, 1H), 3.61 (*app*. p, *J* = 8.3 Hz, 1H), 3.38 (dd, *J* = 9.4, 7.4 Hz, 1H), 2.70 (dd, *J* = 16.8, 8.9 Hz, 1H), 2.47 (dd, *J* = 16.9, 8.9 Hz, 1H), 1.97 – 1.76 (m,

6H), 1.67 – 1.54 (m, 2H). **^{13}C NMR (101 MHz, $CDCl_3$)**: δ (ppm) = 178.0, 149.3, 148.0, 134.7, 118.9, 113.9, 112.3, 56.3, 49.9, 40.1, 38.3, 32.9, 24.1. The spectroscopic data were in agreement to those previously reported.[21] **Optical Rotation**: $[\alpha]_D^{25}$ = –24.6 (c = 0.5, $CHCl_3$) for an enantiomerically enriched sample of 97:03 *er*. The enantiomeric purity was established by HPLC analysis using a chiral column (Lux® *i*Amylose-3, 40 °C, 1 mL/min, 90:10 *n*-hexane:isopropanol, 214 nm, t = 16.925 min and 18.215 min).

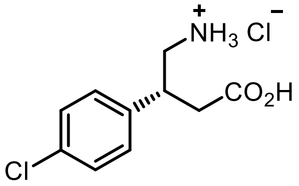

(*R*)-Baclofen [13]

Prepared according to the method reported in the literature.[24] (*R*)-4-(4-Chlorophenyl)pyrrolidin-2-one **[2h]** (19.6 mg, 100 µmol, 1.00 eq.) was suspended in 6 N HCl (2 mL) and stirred at 100 °C for 16 h. After washings with ethyl acetate the aqueous phase was concentrated *in vacuo* to afford the product (23.6 mg, 94 µmol, 94%) as a colorless solid.

^{1}H NMR (400 MHz, DMSO-d6): δ (ppm) = 12.25 (s, 1H), 8.17 (s, 3H), 7.49 – 7.23 (m, 4H), 3.15 – 3.04 (m, 1H), 3.02 – 2.91 (m, 1H), 2.87 (dd, *J* = 16.4, 5.4 Hz, 1H), 2.57 (dd, *J* = 16.3, 9.5 Hz, 1H). **^{13}C NMR (101 MHz, DMSO-d6)**: δ (ppm) = 172.3, 139.4, 131.8, 130.0, 128.5, 43.2, 37.8. The spectroscopic data were in agreement to those previously reported.[25] **Optical Rotation**: $[\alpha]_D^{25}$ = –2.6 (c = 0.5, H_2O). Reported $[\alpha]_D^{25}$ for (*R*)-baclofen (H_2O, c= 0.6)= –3.8, 99 % ee.[25]

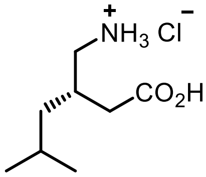

(*S*)-Pregabalin [14]

Prepared according to the method reported in the literature.[24] (*S*)-4-Isobutylpyrrolidin-2-one **[2m]** (14.2 mg, 100 µmol, 1.00 eq.) was suspended in 6 N HCl (2 mL) and stirred at 100 °C for 16 h. After washings with ethyl acetate the aqueous phase was concentrated *in vacuo* to afford the product (15.4 mg, 79 µmol, 79%) as a colorless solid.

^{1}H NMR (400 MHz, CD_3OD): δ (ppm) = 3.03 – 2.89 (m, 2H), 2.51 – 2.34 (m, 2H), 2.21 (hept, *J* = 6.7 Hz, 1H), 1.70 (hept, *J* = 6.7 Hz, 1H), 1.27 (t, *J* = 7.2 Hz, 2H), 0.96 (d, *J* = 6.6 Hz, 3H), 0.93 (d, *J* = 6.5 Hz, 3H).**^{13}C NMR (101 MHz, CD_3OD)**: δ (ppm) = 175.8, 44.5, 42.1, 37.1, 32.6, 26.2, 23.2, 22.5. The spectroscopic data were in agreement to those previously reported.[24] **Optical Rotation**: $[\alpha]_D^{25}$ = +8.3 (c = 0.5, H_2O). Reported $[\alpha]_D^{20}$ for (*S*)-Pregabalin (H_2O, c= 1.1)= +7.15.[26]

7. Crystallographic data

7.1. X-ray crystal structure of [6i] (mar647)

A colorless plate like specimen of $C_{22}H_{19}ClNO_2P$, approximate dimensions 0.030 x 0.190 x 0.290 mm³, was used for the X-ray crystallographic analysis. The x-ray intensity data were measured on a STOE IPDS 2T Diffractometer system. CCDC number: 2465049

Table *S6*. Crystal data and structure refinement for [6i] (mar647)

Identification code	mar647	
Empirical formula	$C_{22}H_{19}ClNO_2P$	
Moiety formula	$C_{22}H_{19}ClNO_2P$	
Formula weight	395.80	
Temperature	120(2) K	
Wavelength, radiation type	0.71073Å, MoKα	
Diffractometer	STOE IPDS 2T	
Crystal type	Triklinic	
Space group name, number	P 1, (1)	
Unit cell dimensions	a = 8.2954(5) Å	α = 73.851(5)°
	b = 9.0584(5) Å	β = 85.864(5)°
	c = 13.3668(8) Å	γ = 79.040(4)°
Volume	946.99(10) $Å^3$	
Number of reflections	34553	
And range used for lattice	2.50° <=Θ<= 28.58°	
Z	2	
Density (calculated)	1.388 Mg/m^3	
Absorption coefficient	0.304 mm^{-1}	
Absorption correction	integration	
Max. and min. transmission	0.9886 and 0.9288	
F(000)	412	
Crystal size, form und color	0.030 x 0.190 x 0.290 mm³, colorless plate	
Theta range for data collection	2.935 to 28.409°.	
Index ranges	-10<=h<=11, -12<=k<=12, -17<=l<=17	
Number of reflections:		
collected	22786	
independent	8806 [R_{int} = 0.0470]	
observed [I>2sigma(I)]	6632	
Completeness of $Θ_{max}$= 25.2°	99.9%	
Refinement method	Full-matrix least-squares on F^2	
Data / restraints / parameters	8806 / 48 / 545	
Goodness-of-fit on F^2	1.066	
Final R indices [I>2sigma(I)]	R1 = 0.0657, wR2 = 0.1267	
R indices (all data)	R1 = 0.1022, wR2 = 0.1479	
Absolut structure parameter	-0.07(9)	
Largest diff. peak and hole	0.310 und -0.376 $eÅ^{-3}$	
Comment	Structure contains 2 independent disordered molecules A and B, where the disordering of B at C7B leads to a change of the enantiomer. Ratio A,B:C,D 0.91:0.09.	

7.2. X-ray crystal structure of [2i] (jha223)

A colorless needle like specimen of $C_{10}H_{10}ClNO$, approximate dimensions 0.070 x 0.160 x 0.510 mm^3, was used for the X-ray crystallographic analysis. The x-ray intensity data were measured on a STOE IPDS 2T Diffractometer system. CCDC number: 2465050

Table *S7*. Crystal data and structure refinement for [2i] (jha223)

Identification code	jha223	
Empirical formula	$C_{10}H_{10}ClNO$	
Moiety formula	$C_{10}H_{10}ClNO$	
Formula weight	195.64	
Temperature	120(2) K	
Wavelength, radiation type	0.71073Å, MoKα	
Diffractometer	STOE IPDS 2T	
Crystal type	Monoclinic	
Space group name, number	$P\ 2_1$, (4)	
Unit cell dimensions	a = 10.118(3) Å	α = 90°
	b = 6.0259(6) Å	β = 107.408(16)°
	c = 15.268(3) Å	γ = 90°
Volume	888.3(3) $Å^3$	
Number of reflections	9997	
And range used for lattice	2.86° <=Θ<= 28.31°	
Z	4	
Density (calculated)	1.463 Mg/m^3	
Absorption coefficient	0.383 mm^{-1}	
Absorption correction	integration	
Max. and min. transmission	0.9735 and 0.8886	
F(000)	408	
Crystal size, form und color	0.070 x 0.160 x 0.510 mm^3, colorless needle	
Theta range for data collection	2.859 to 28.082°.	
Index ranges	-13<=h<=13, -7<=k<=7, -20<=l<=15	
Number of reflections:		
collected	7119	
independent	4101 [R_{int} = 0.0332]	
observed [I>2sigma(I)]	3234	
Completeness of $Θ_{max}$= 25.2°	99.5%	
Refinement method	Full-matrix least-squares on F^2	
Data / restraints / parameters	4101 / 3 / 241	
Goodness-of-fit on F^2	1.045	
Final R indices [I>2sigma(I)]	R1 = 0.0509, wR2 = 0.1060	
R indices (all data)	R1 = 0.0742, wR2 = 0.1171	
Absolut structure parameter	-0.16(9)	
Largest diff. peak and hole	0.315 und -0.270 $eÅ^{-3}$	

8. References

1 C. P. Rosenau, B. J. Jelier, A. D. Gossert and A. Togni, *Angew. Chem. Int. Ed.*, 2018, **57**, 9528.

2 J. Sietmann, M. Ong, C. Mück-Lichtenfeld, C. G. Daniliuc and J. M. Wahl, *Angew. Chem. Int. Ed.*, 2021, **60**, 9719.

3 M. Ong, M. Arnold, A. W. Walz and J. M. Wahl, *Org. Lett.*, 2022, **24**, 6171.

4 S. Müller, M. J. Webber and B. List, *J. Am. Chem. Soc.*, 2011, **133**, 18534.

5 M. W. Giuliano, C.-Y. Lin, D. K. Romney, S. J. Miller and E. V. Anslyn, *Adv Synth Catal*, 2015, **357**, 2301.

6 P. P. Poudel, K. Arimitsu and K. Yamamoto, *Chem. Commun.*, 2016, **52**, 4163.

7 K. Marjani, M. Mousavi, O. Arazi, A. Ashouri, S. Bourghani and M. Rajabi, *Monatsh Chem.*, 2009, **140**, 1331.

8 S. Shirakawa, T. Tokuda, S. B. J. Kan and K. Maruoka, *Org. Chem. Front.*, 2015, **2**, 336.

9 I. Tomiya, Y. Wu and K. Hyodo, *Adv. Synth. Catal.*, 2024, **366**, 1606.

10 M. Arnold, J. Hammes, M. Ong, C. Mück-Lichtenfeld and J. M. Wahl, *Angew. Chem. Int. Ed.*, 2025, **64**, e202503056.

11 M. Charaschanya, K. Li, H. F. Motiwala and J. Aubé, *Organic letters*, 2018, **20**, 6354.

12 M. Klussmann, L. Ratjen, S. Hoffmann, V. Wakchaure, R. Goddard and B. List, *Synlett*, 2010, **2010**, 2189.

13 V. Rauniyar, Z. J. Wang, H. E. Burks and F. D. Toste, *J. Am. Chem. Soc.*, 2011, **133**, 8486.

14 Y. Wu, M. Inoue, S. Sakakura and K. Hyodo, *Org. Biomol. Chem.*, 2024, **22**, 4364.

15 I. J. Montoya-Balbás, B. Valentín-Guevara, E. López-Mendoza, I. Linzaga-Elizalde, M. Ordoñez and P. Román-Bravo, *Molecules*, 2015, **20**, 22028.

16 B. Schmidt, N. Elizarov, R. Berger and M. Petersen, *Synthesis*, 2013, **45**, 1174.

17 R. Tomar, D. Bhattacharya and S. A. Babu, *Tetrahedron*, 2019, **75**, 2447.

18 F. Coelho, M. B. M. de Azevedo, R. Boschiero and P. Resende, *Synth. Commun.*, 1997, **27**, 2455.

19 M. Mathé-Allainmat, M. Le Gall, C. Jellimann, J. Andrieux and M. Langlois, *Bioorg. Med. Chem.*, 1999, **7**, 2945.

20 S. Kerres, E. Plut, S. Malcherek, J. Rehbein and O. Reiser, *Adv. Synth. Catal.*, 2019, **361**, 1400.

21 D. Enders, R. Gröbner, G. Raabe and J. Runsink, *Synthesis*, 1996, **1996**, 941.

22 O. Lavinda, C. H. Witt and K. A. Woerpel, *Angew. Chem. Int. Ed.*, 2022, **61**, e202114183.

23 a) *US Pat.*, US2010022784 (A1), 2007; b) A. N. Reznikov, E. V. Golovin and Y. N. Klimochkin, *Russ. J. Org. Chem.*, 2013, **49**, 663;

24 A. Leyva-Pérez, P. García-García and A. Corma, *Angew. Chem. Int. Ed.*, 2014, **126**, 8831.

25 T. Okino, Y. Hoashi, T. Furukawa, X. Xu and Y. Takemoto, *J. Am. Chem. Soc.*, 2005, **127**, 119.

26 J. Liu, X. Wang, Z. Ge, Q. Sun, T. Cheng and R. Li, *Tetrahedron*, 2011, **67**, 636.

9. NMR Spectra and HPLC Traces

[1i] ^1H, $CDCl_3$, 400 MHz

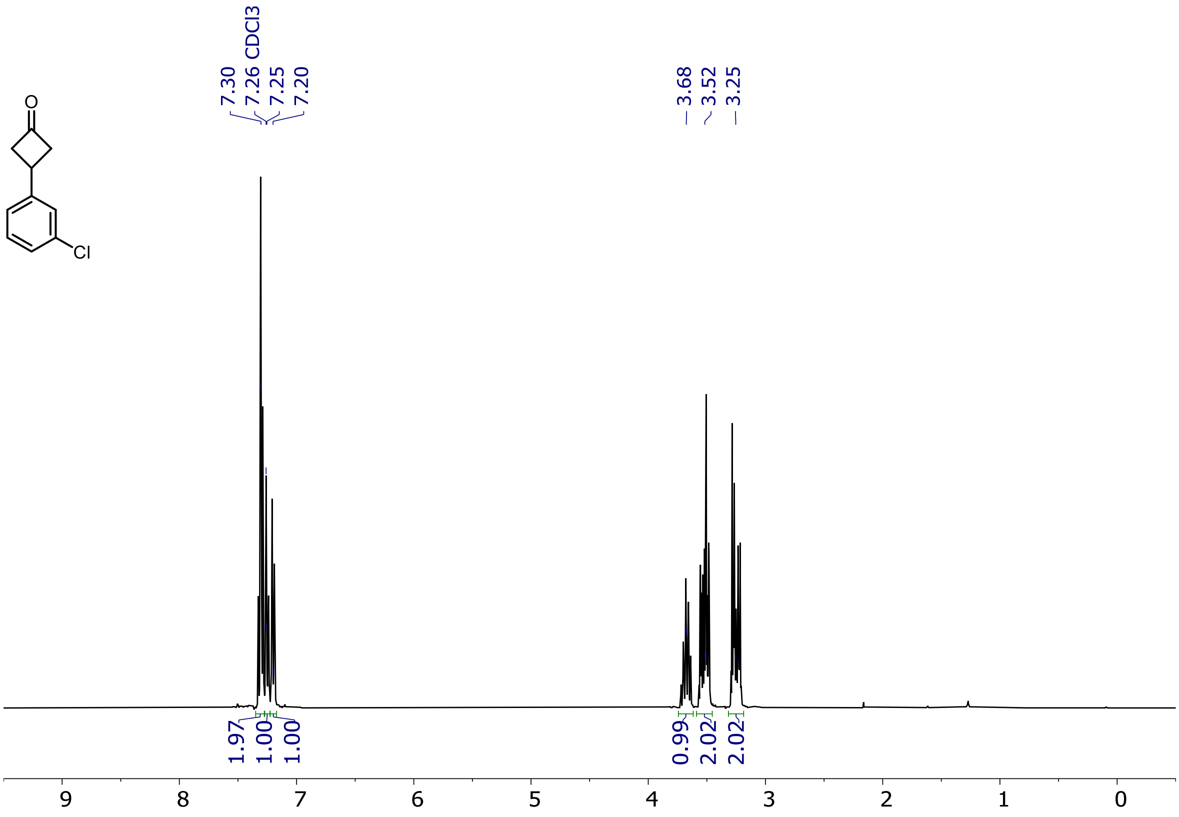

[1i] ^{13}C, $CDCl_3$, 101 MHz

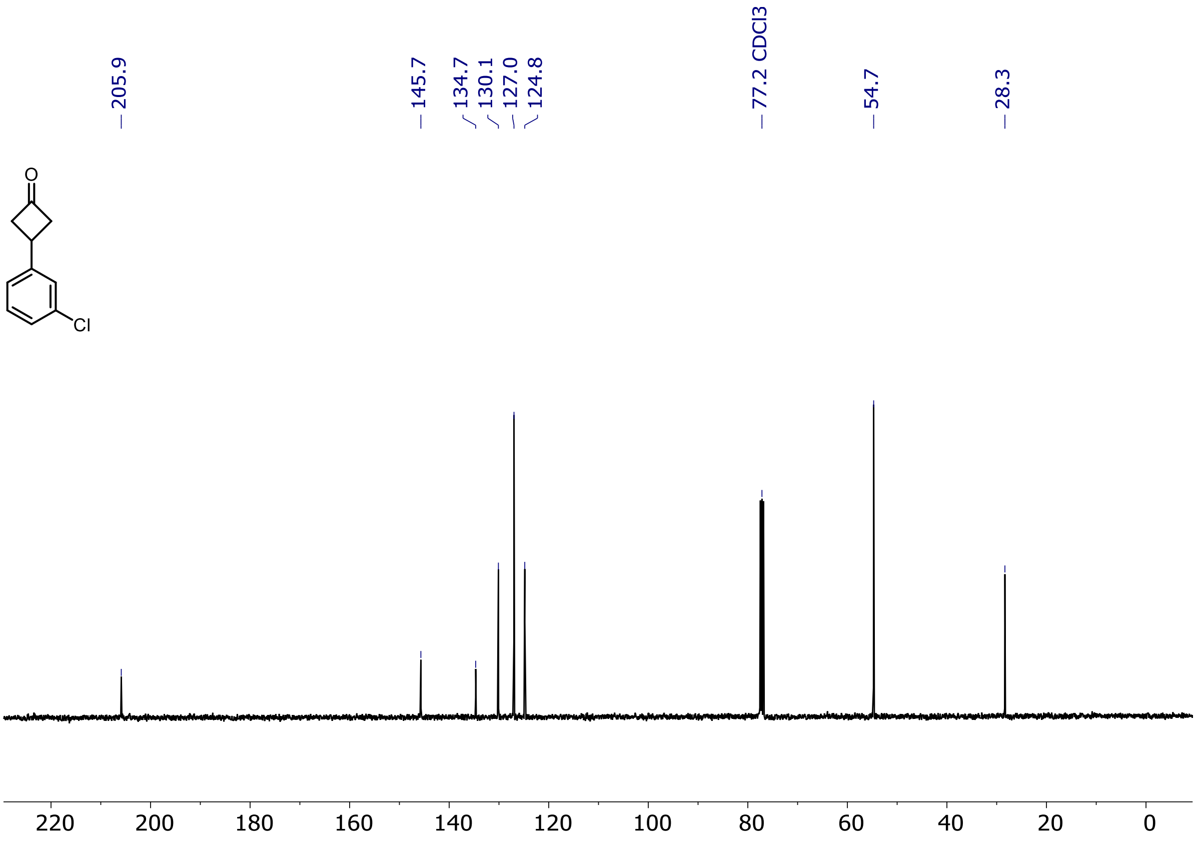

[1j] 1H, $CDCl_3$, 400 MHz

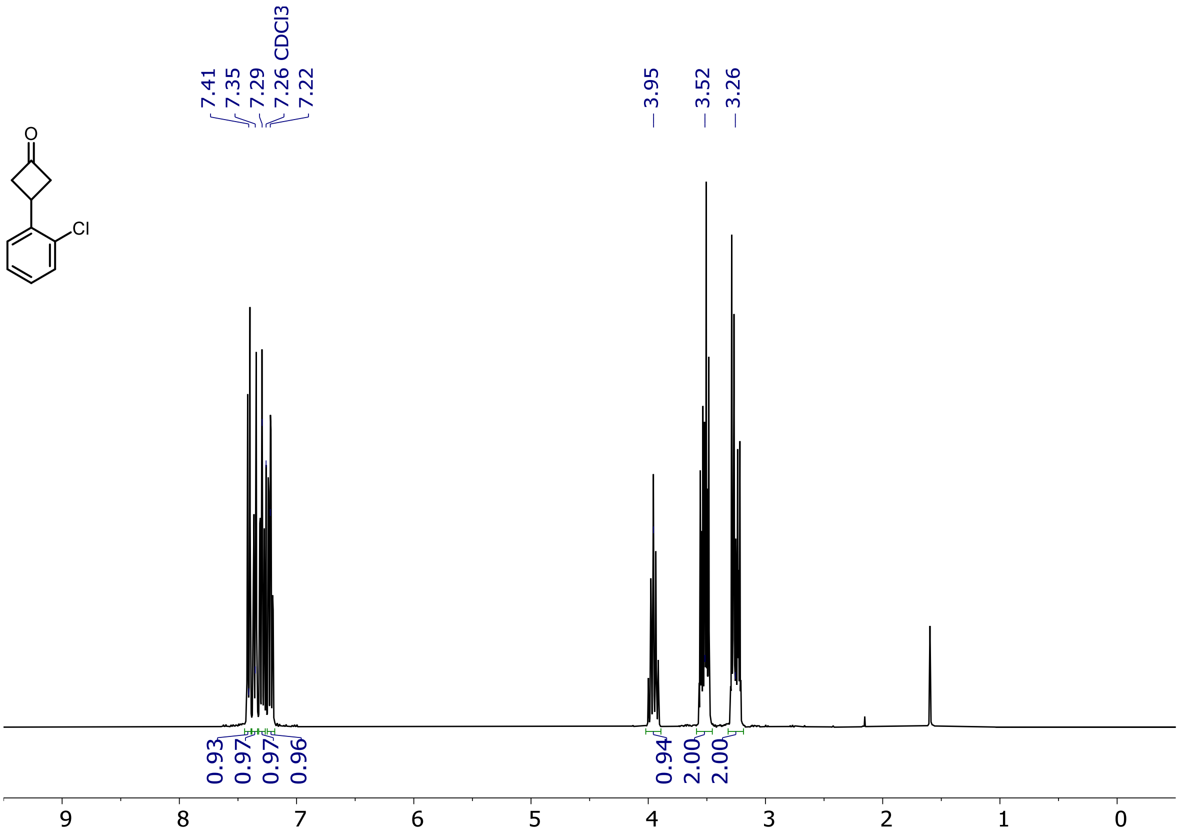

[1j] ^{13}C, $CDCl_3$, 101 MHz

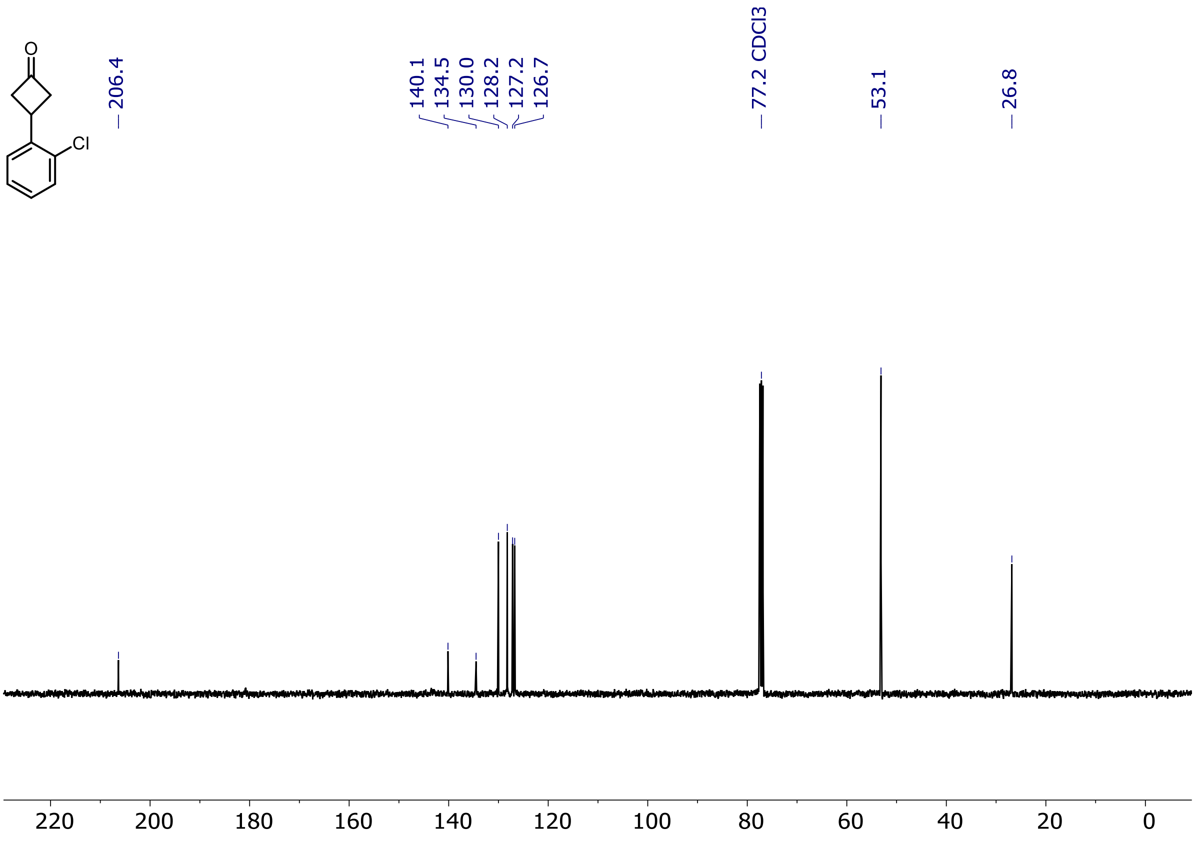

[1k] ^1H, $CDCl_3$, 400 MHz

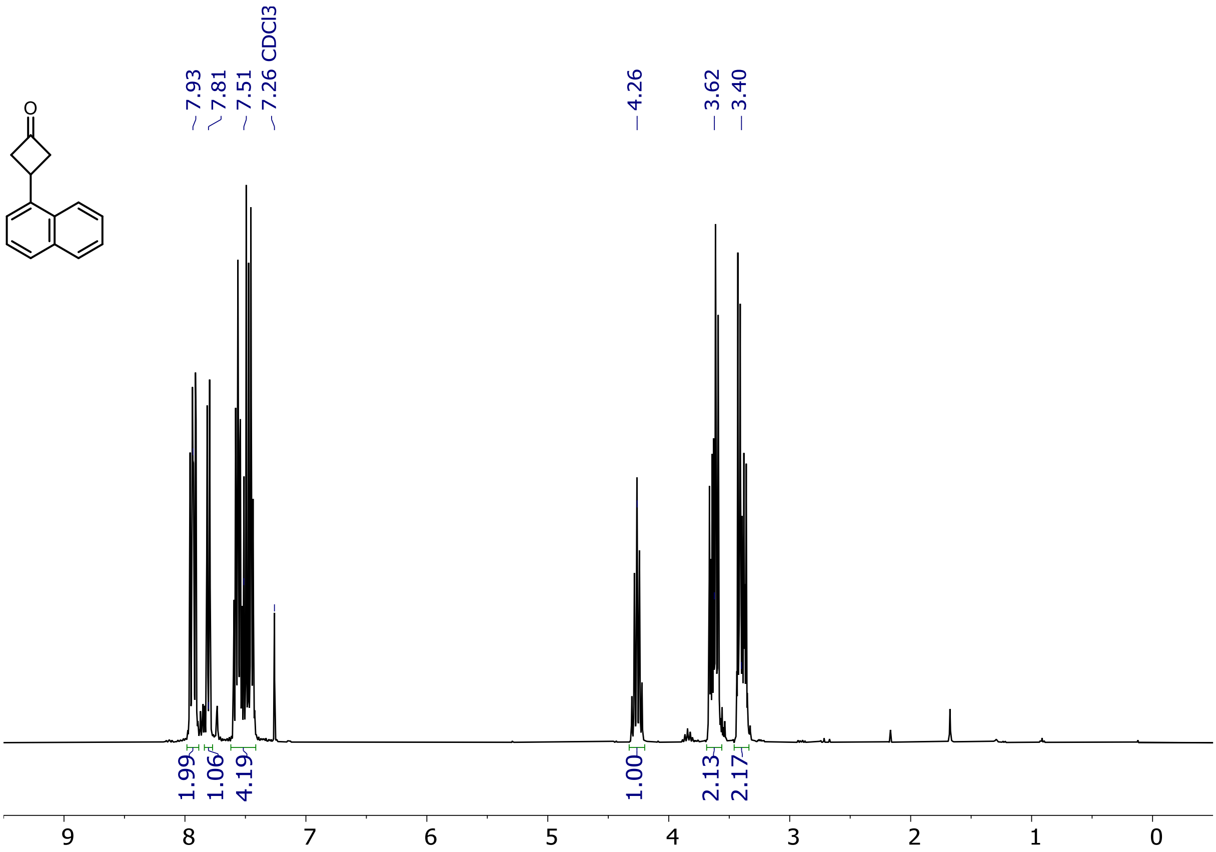

[1k] ^{13}C, $CDCl_3$, 101 MHz

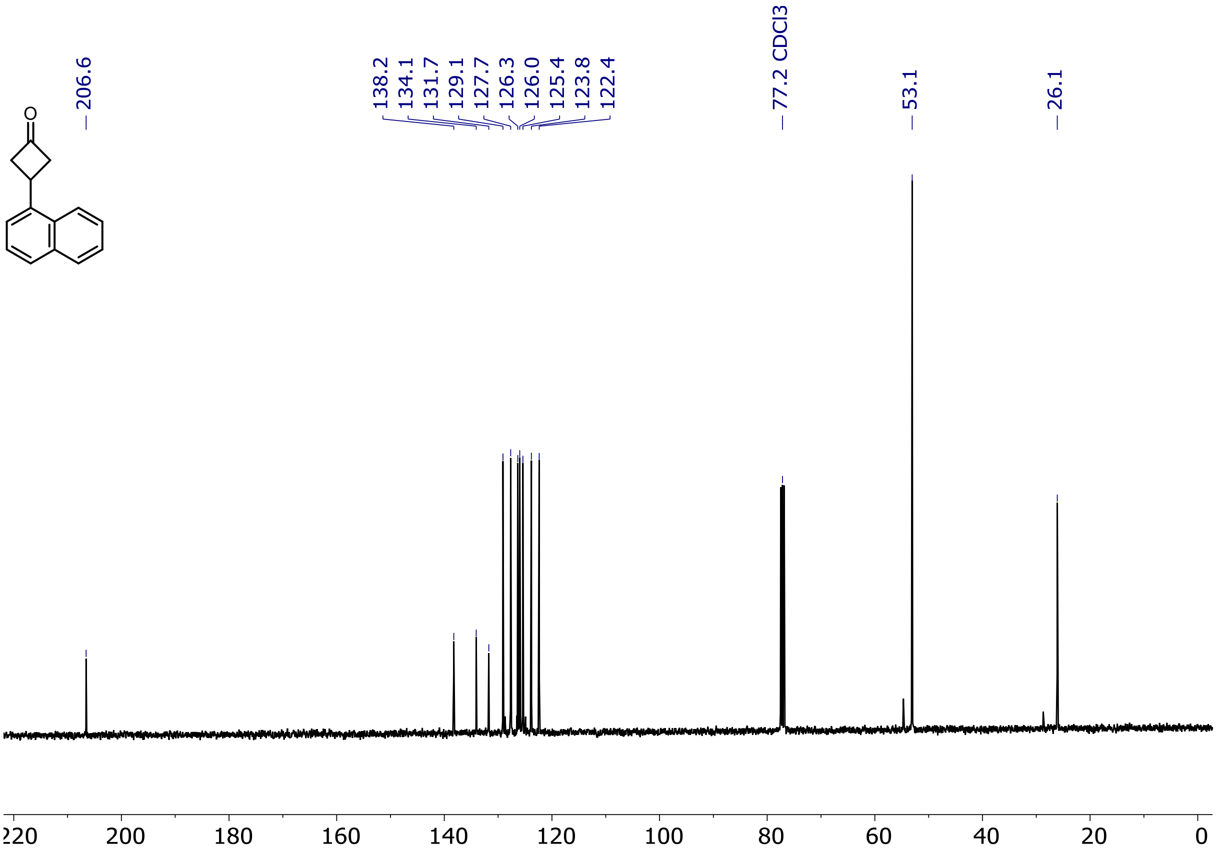

[S1o] ^{1}H, $CDCl_3$, 400 MHz

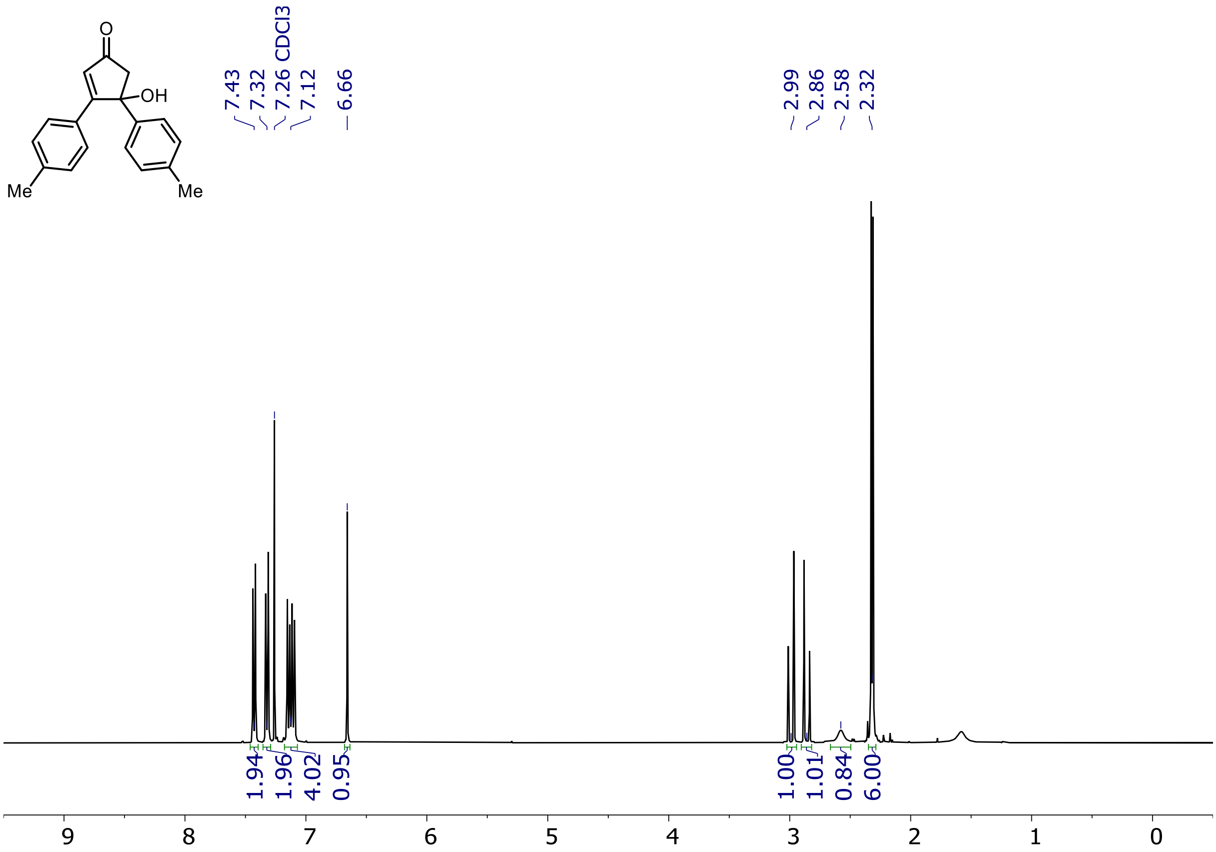

[S1o] ^{13}C, $CDCl_3$, 101 MHz

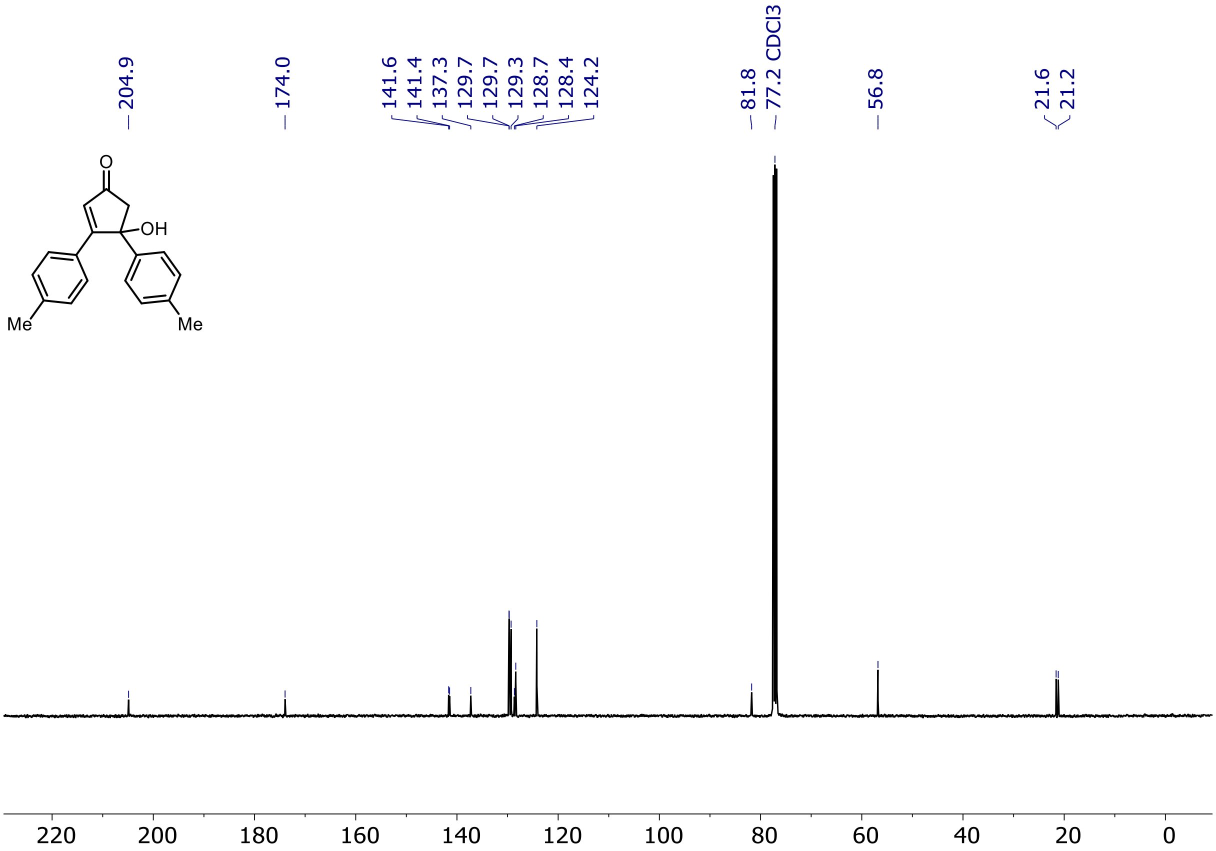

[S1p] ^{1}H, $CDCl_3$, 400 MHz

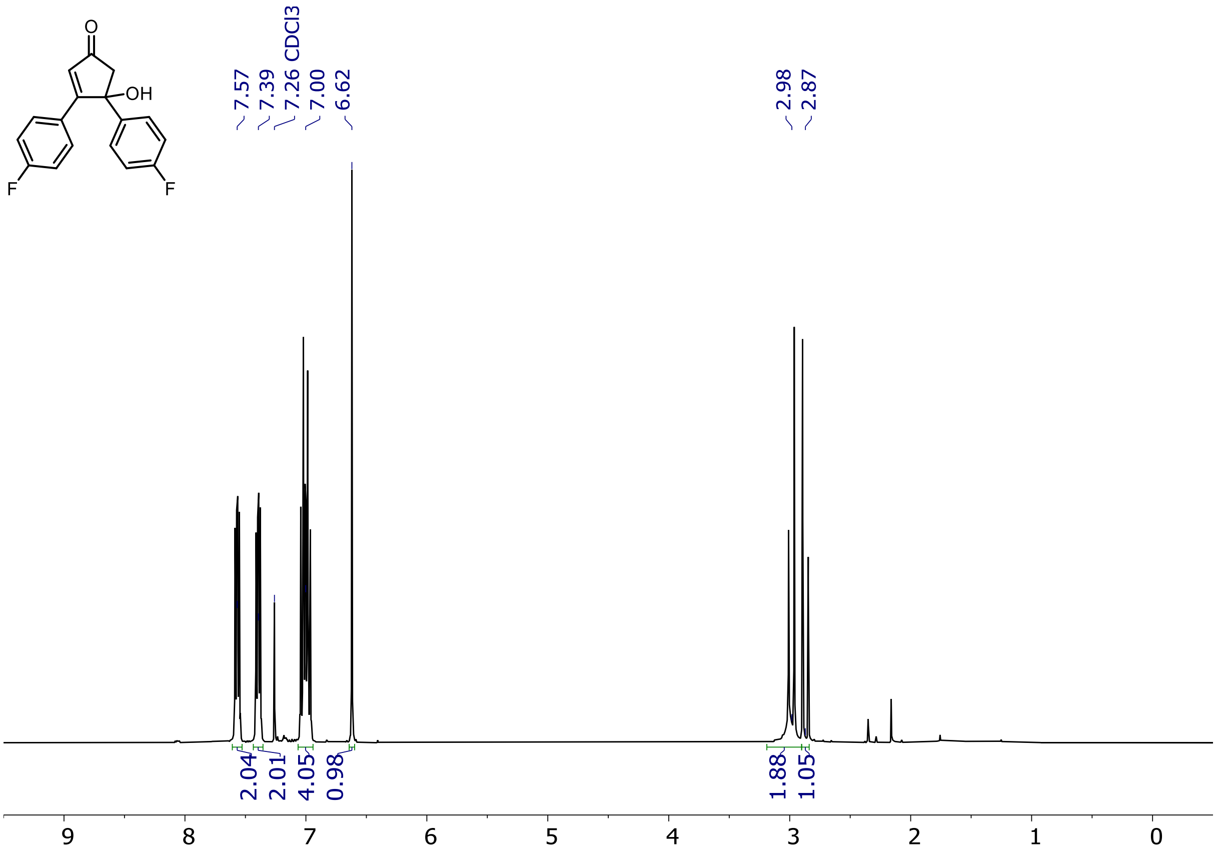

[S1p] ^{13}C, $CDCl_3$, 101 MHz

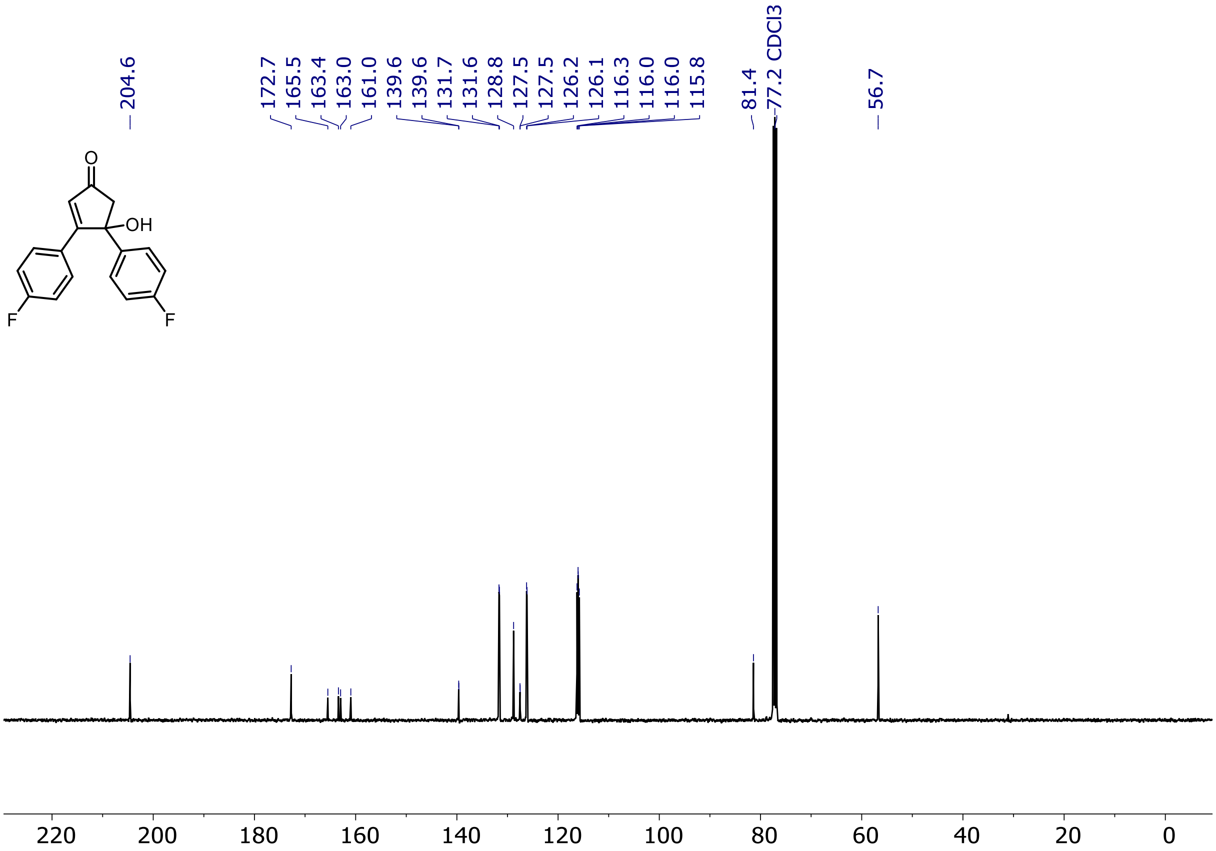

[S1p] ^{19}F, $CDCl_3$, 282 MHz

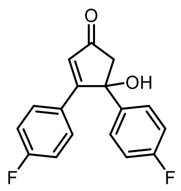

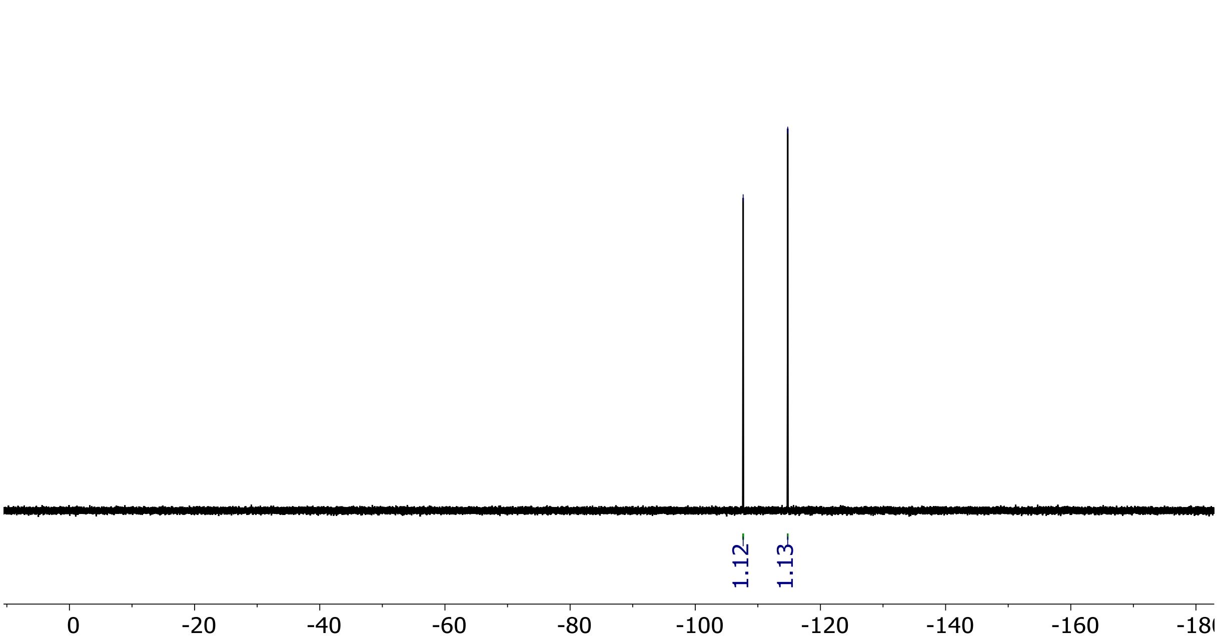

[S2o] ^{1}H, $CDCl_3$, 400 MHz

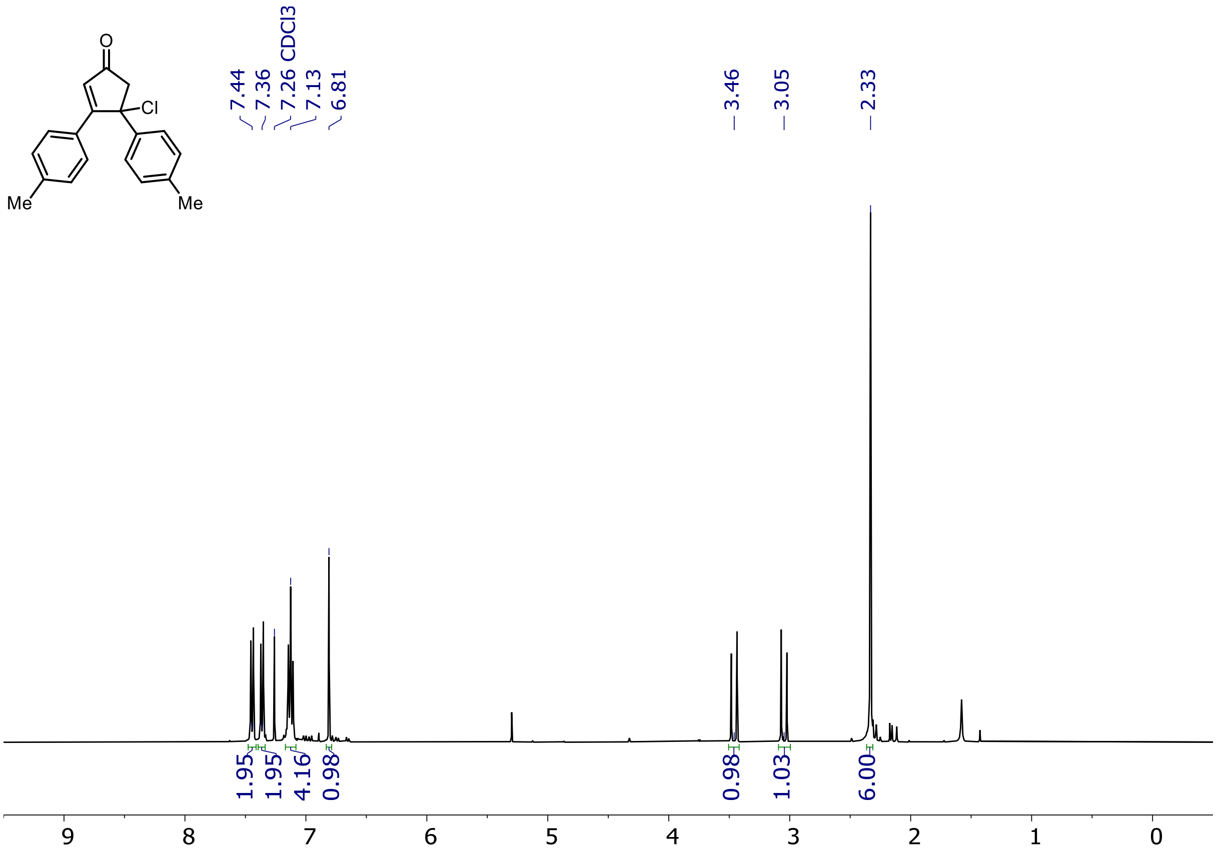

[S2o] ^{13}C, $CDCl_3$, 101 MHz

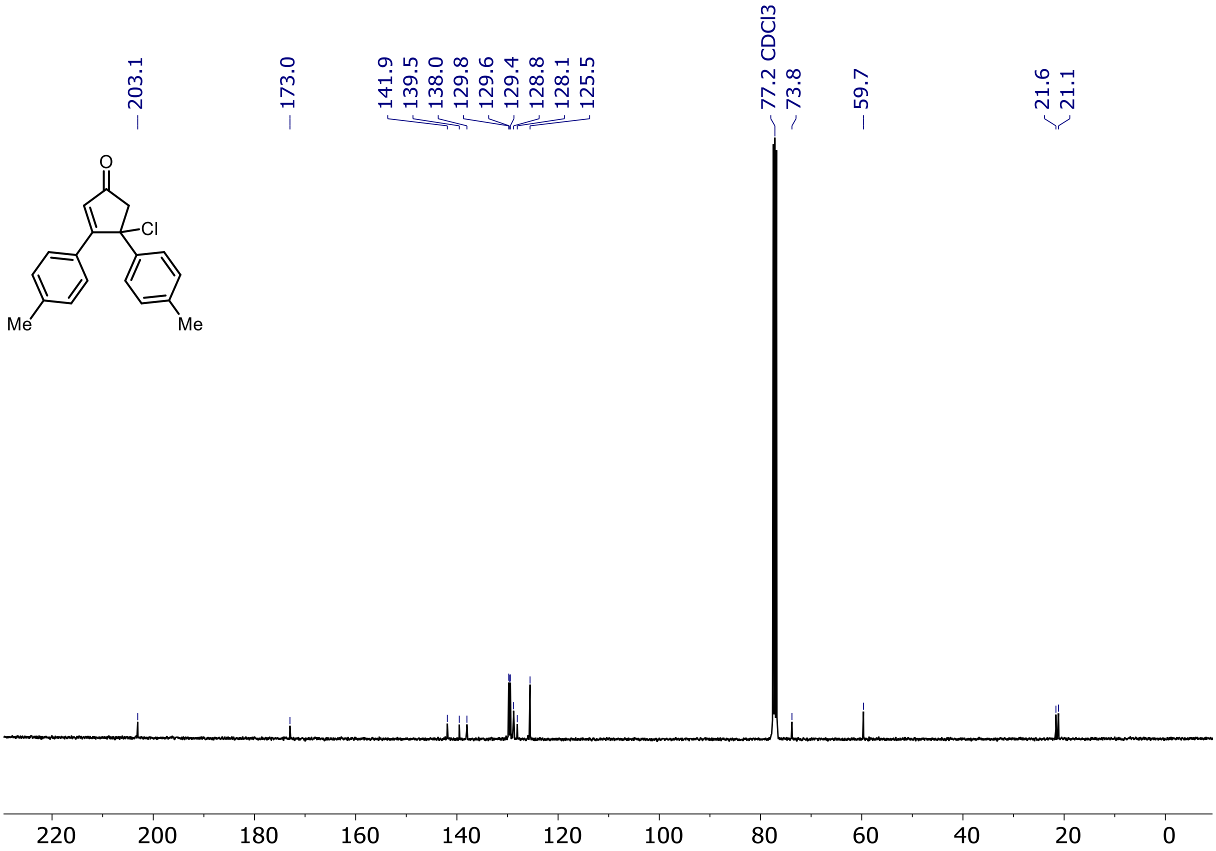

[S2p] ^{1}H, $CDCl_3$, 400 MHz

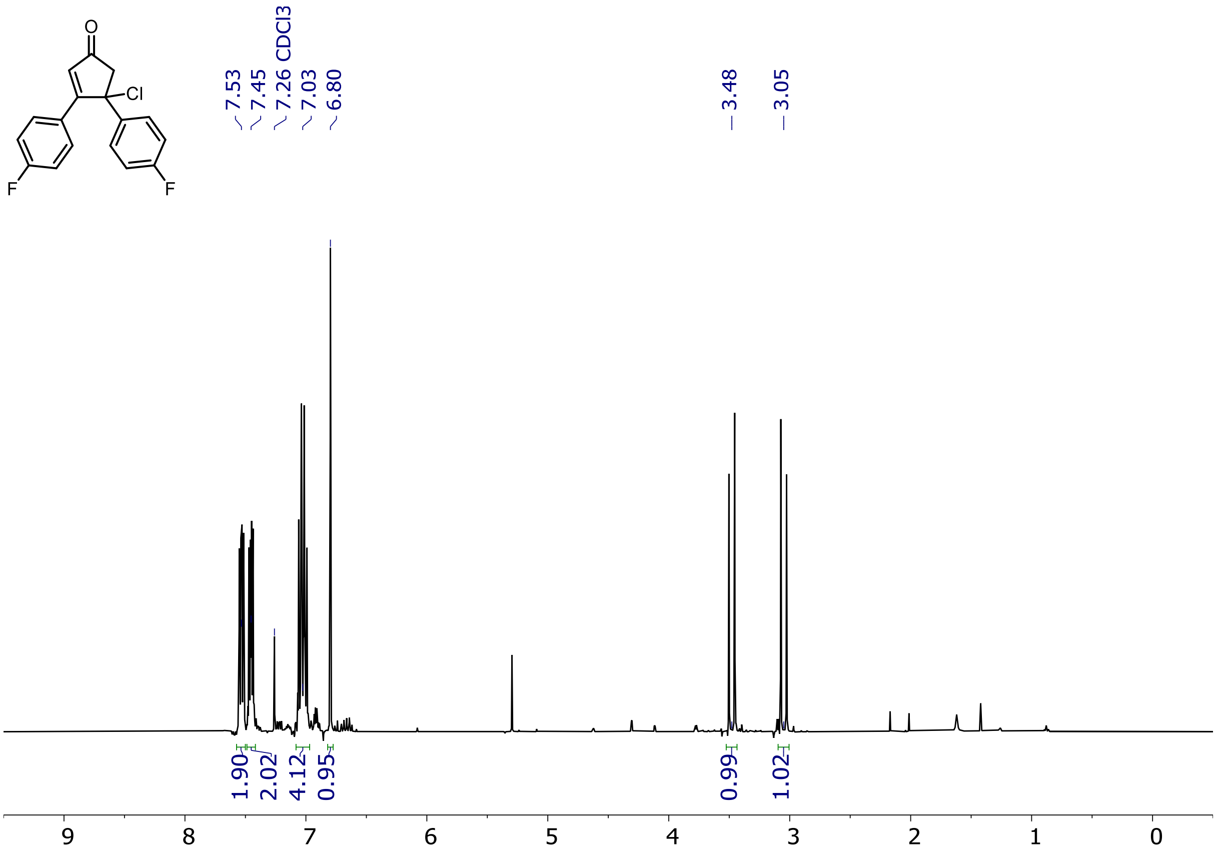

[S2p] ^{13}C, $CDCl_3$, 101 MHz

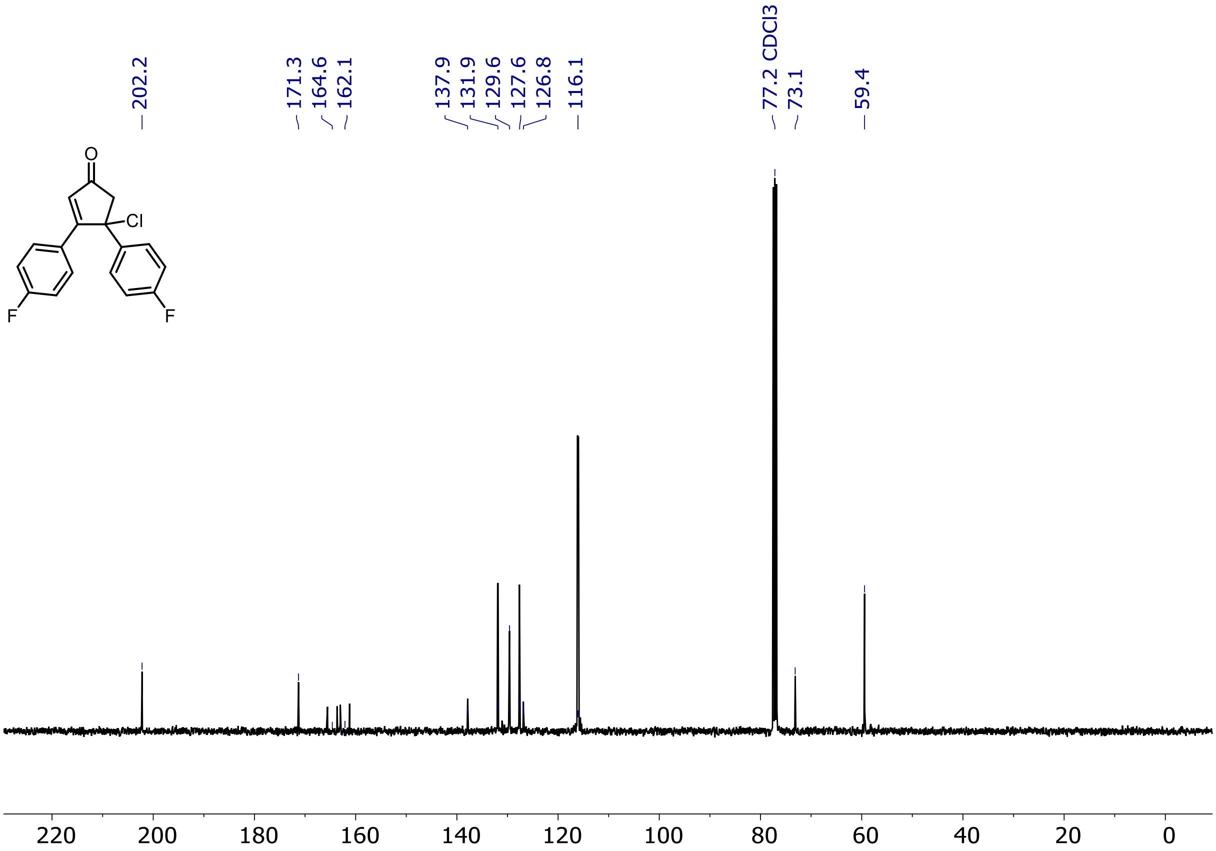

[S2p] ^{19}F, $CDCl_3$, 282 MHz

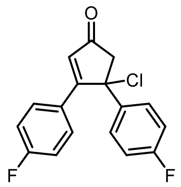

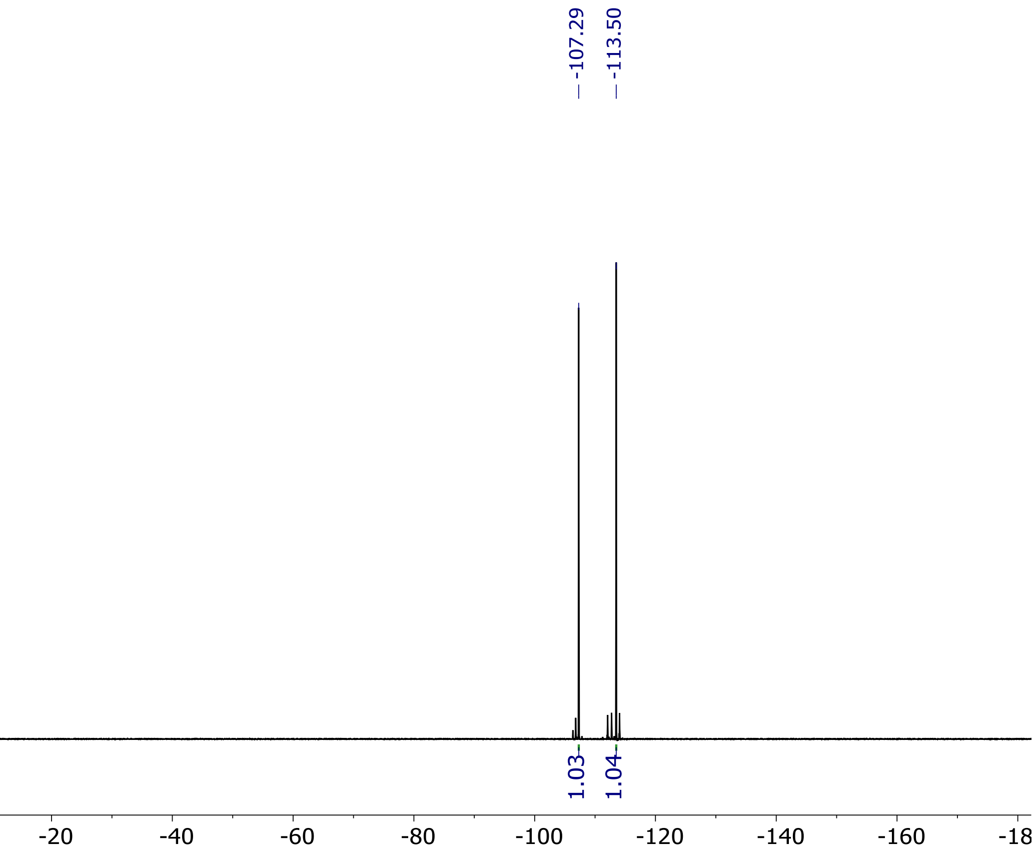

[S3o] ^{1}H, C_6D_6, 400 MHz

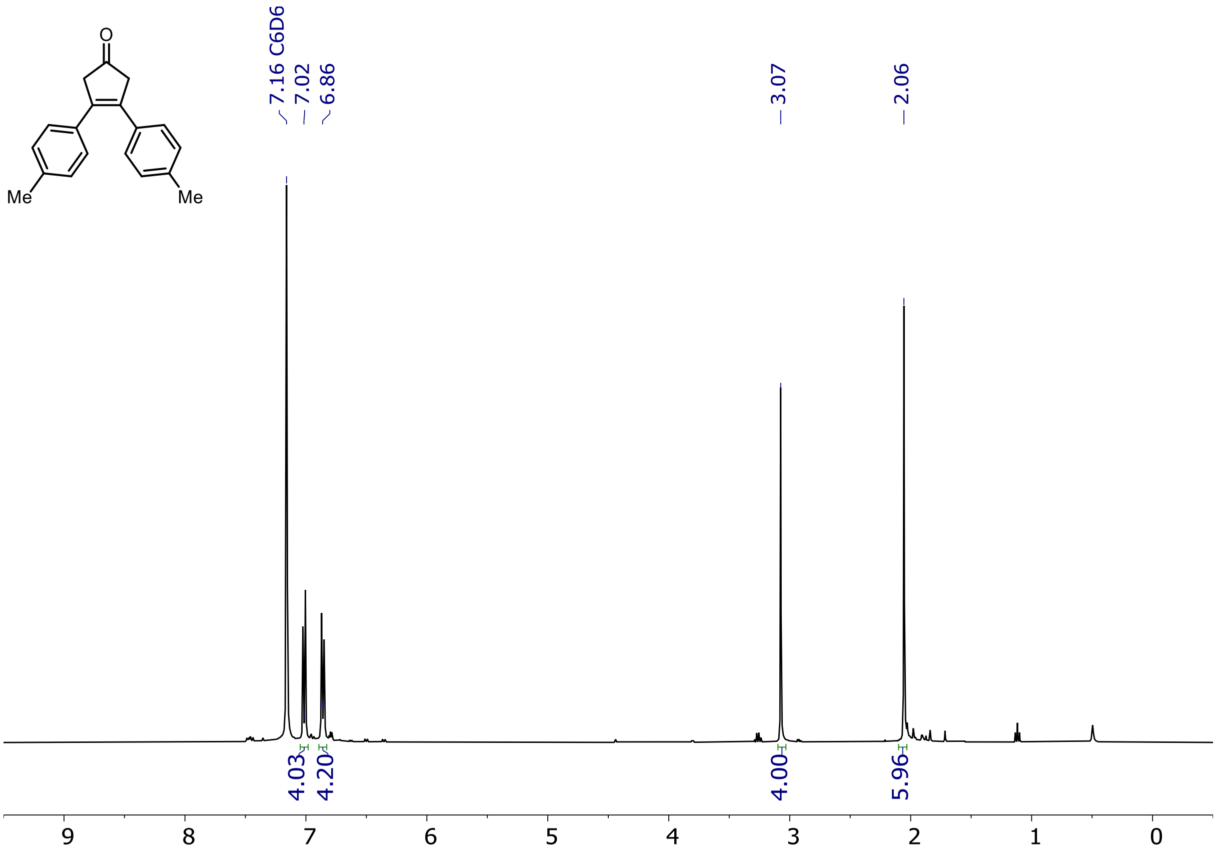

[S3o] ^{13}C, C_6D_6, 101 MHz

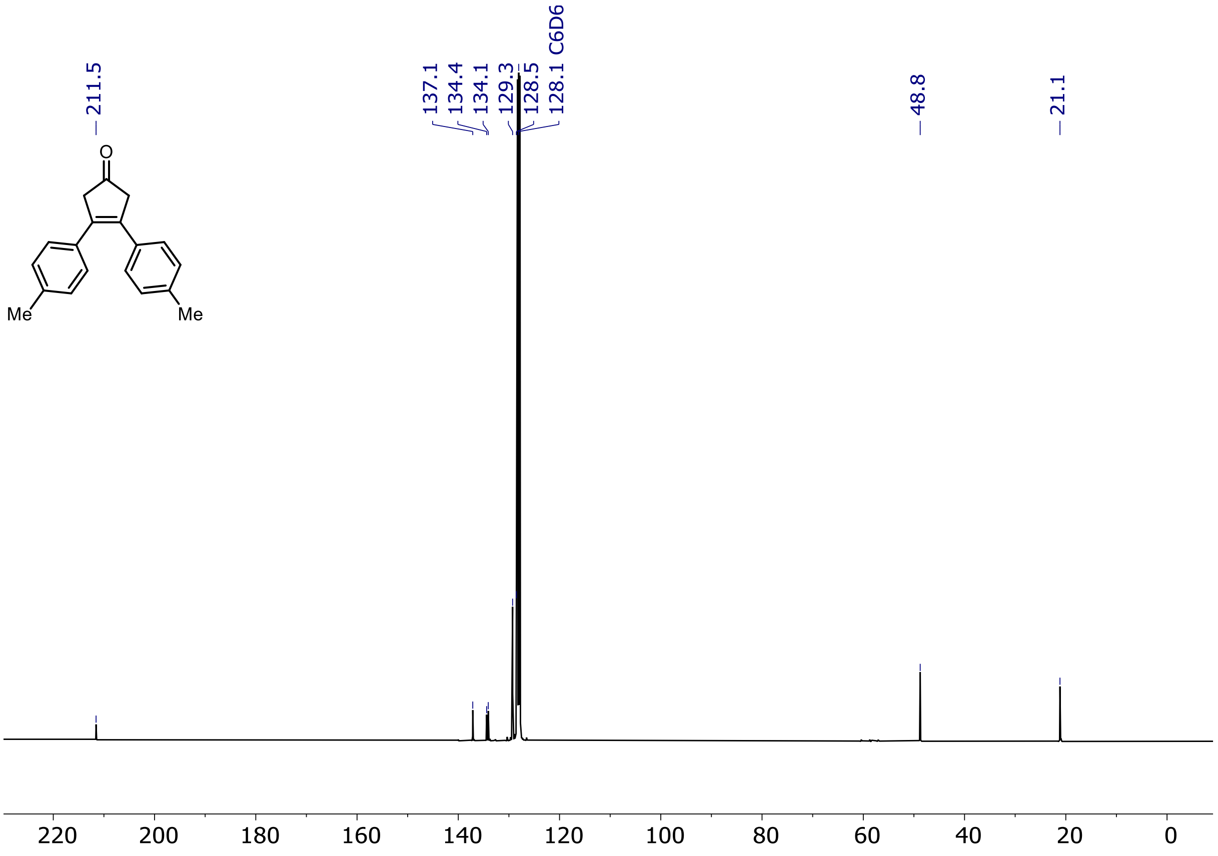

[S3p] ^{1}H, C_6D_6, 400 MHz

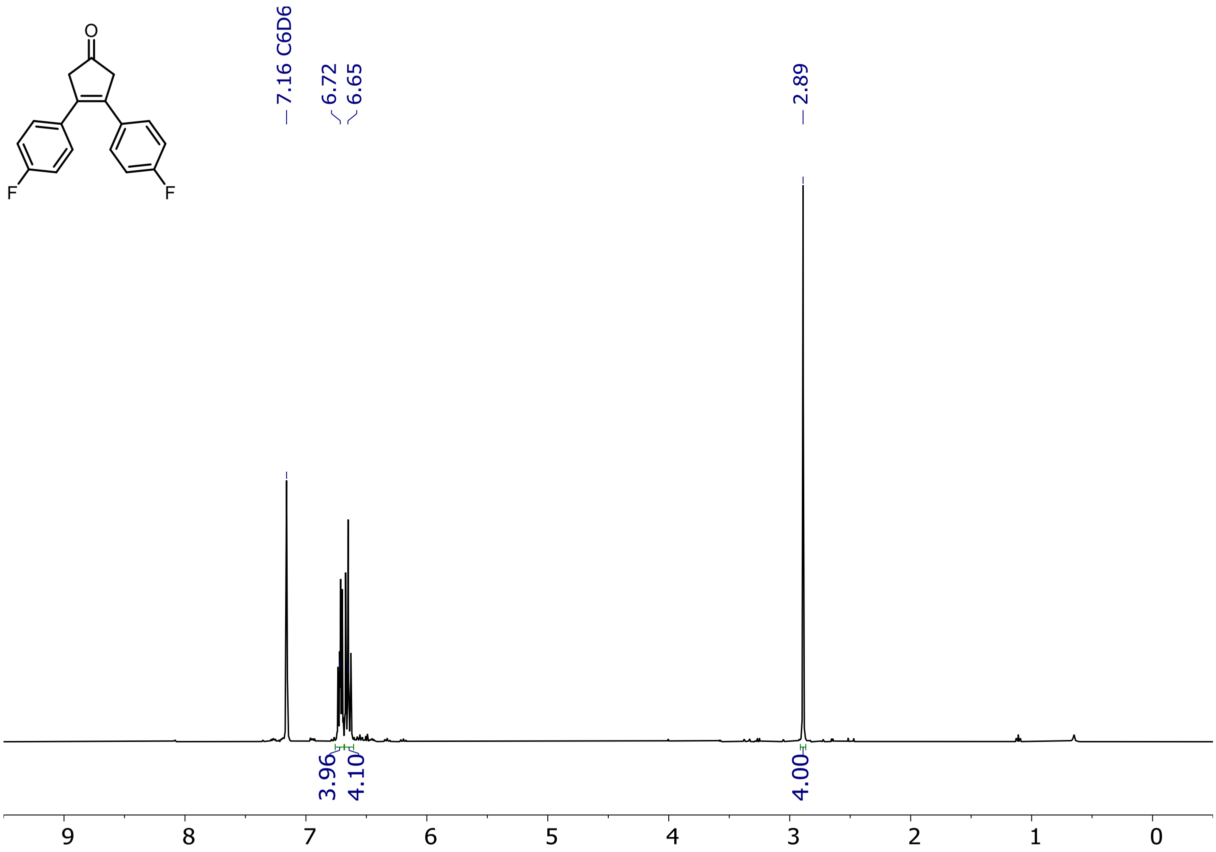

[S3p] ^{13}C, C_6D_6, 101 MHz

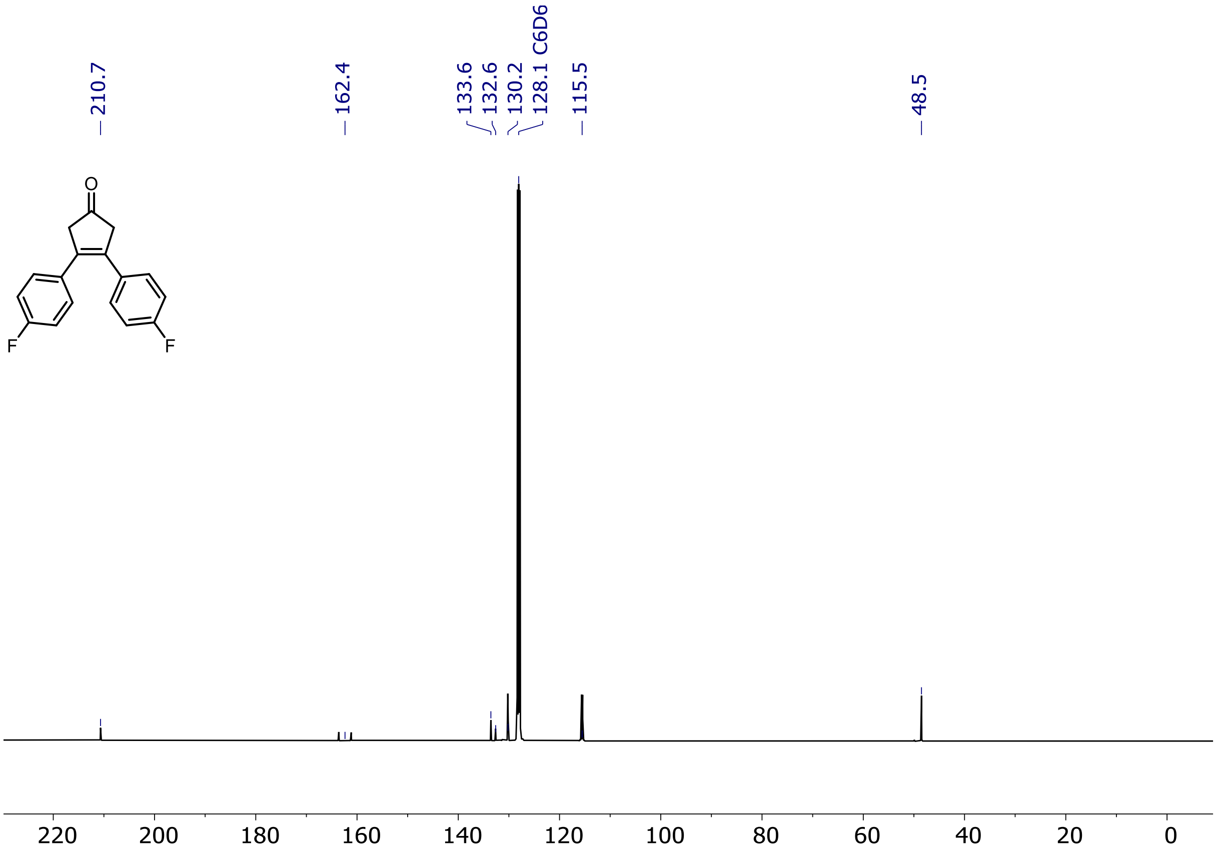

[S3p] ^{19}F, C_6D_6, 282 MHz

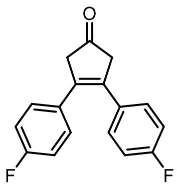

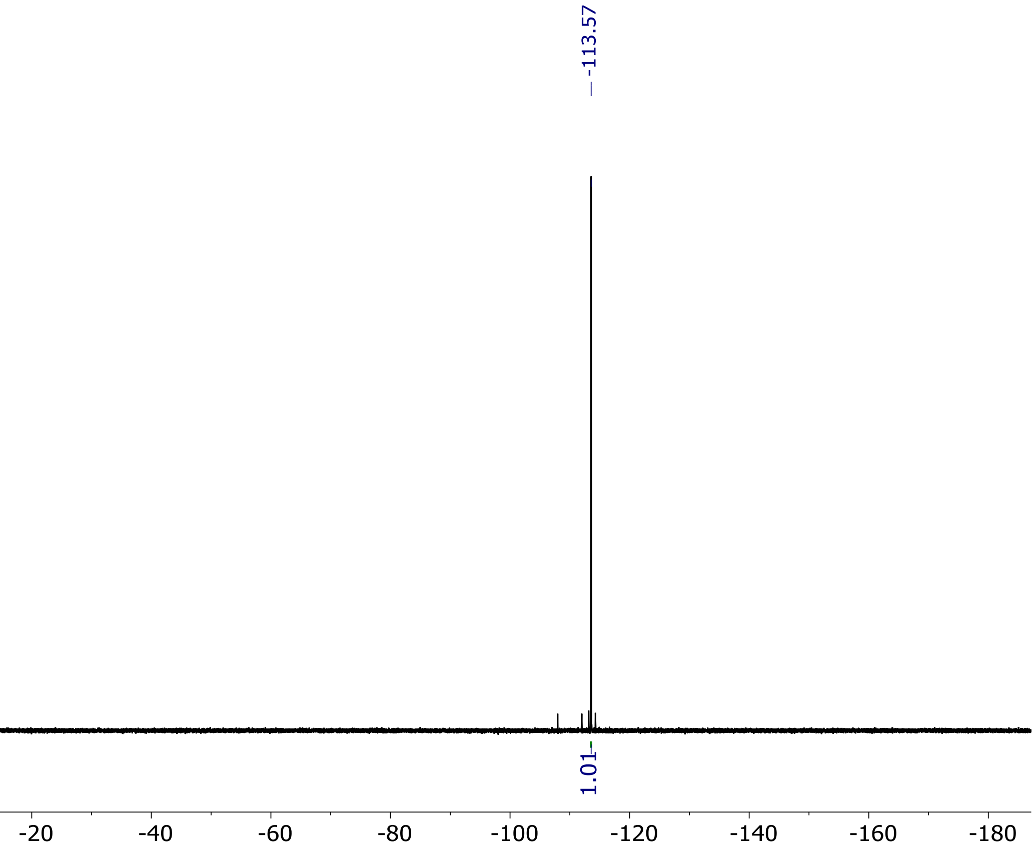

[S4o] ^{1}H, C_6D_6, 400 MHz

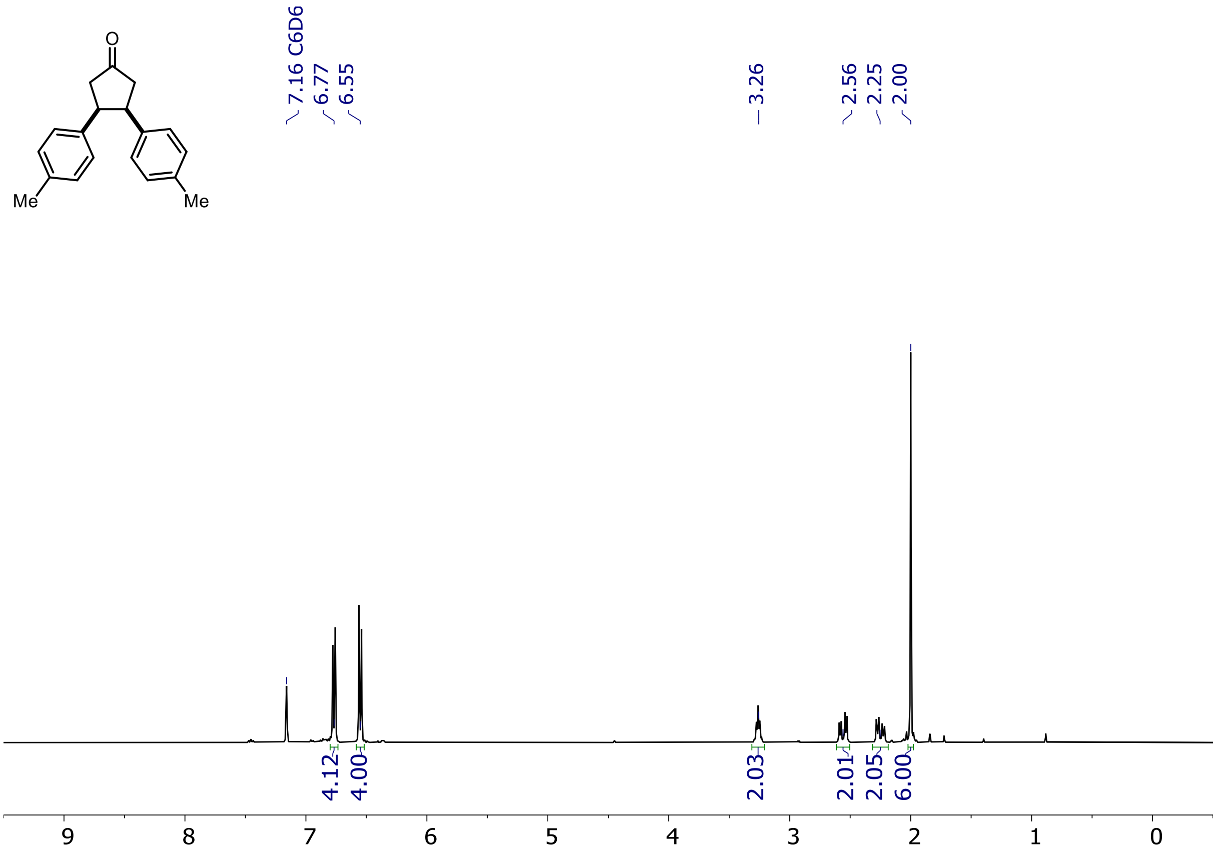

[S3o] ^{13}C, C_6D_6, 101 MHz

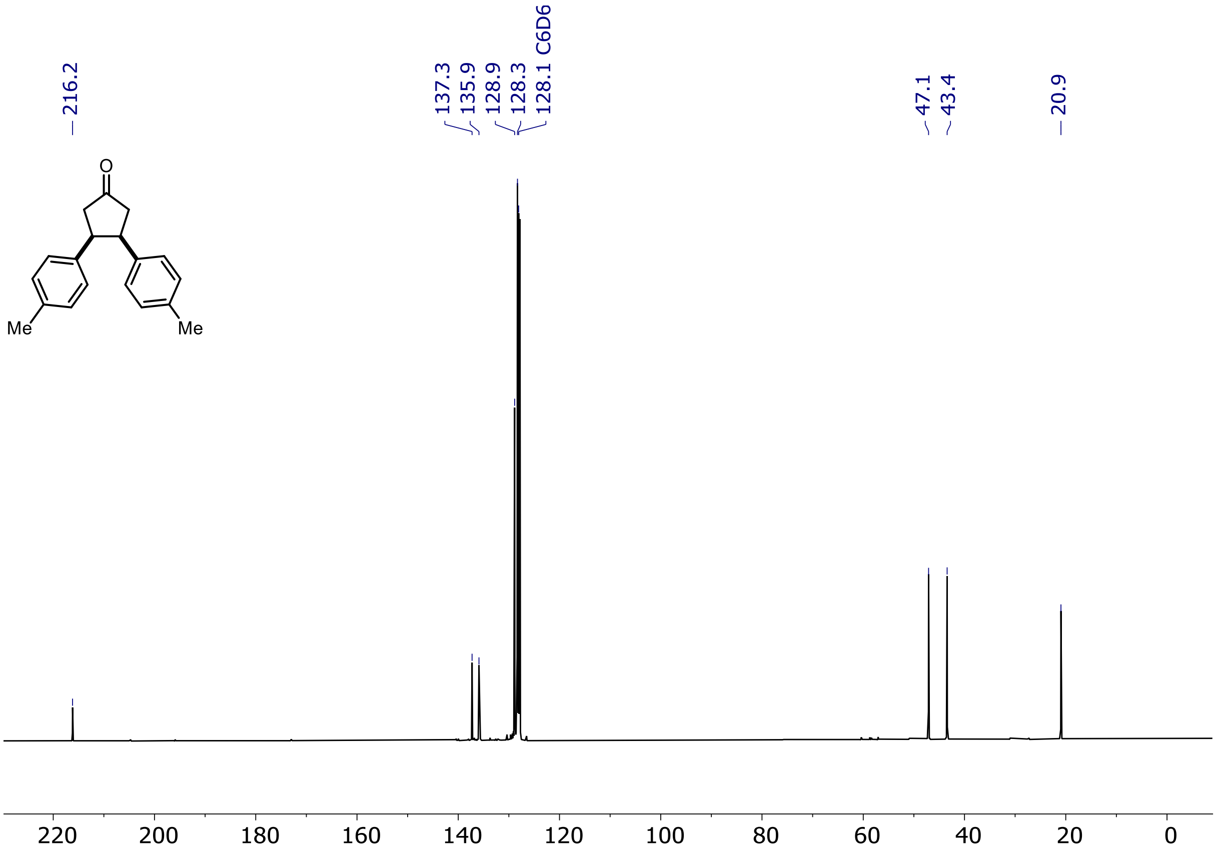

[S4p] ^1H, C_6D_6, 400 MHz

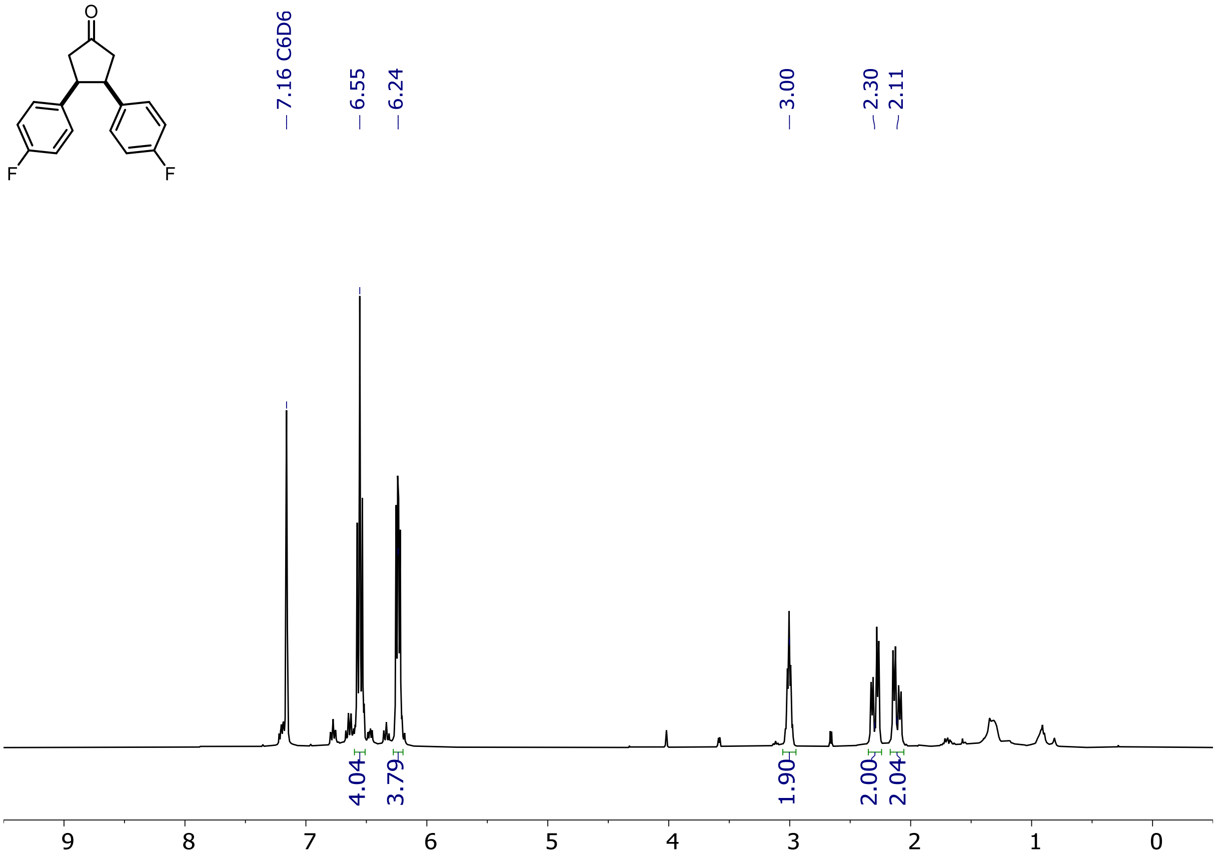

[S4p] ^{13}C, C_6D_6, 101 MHz

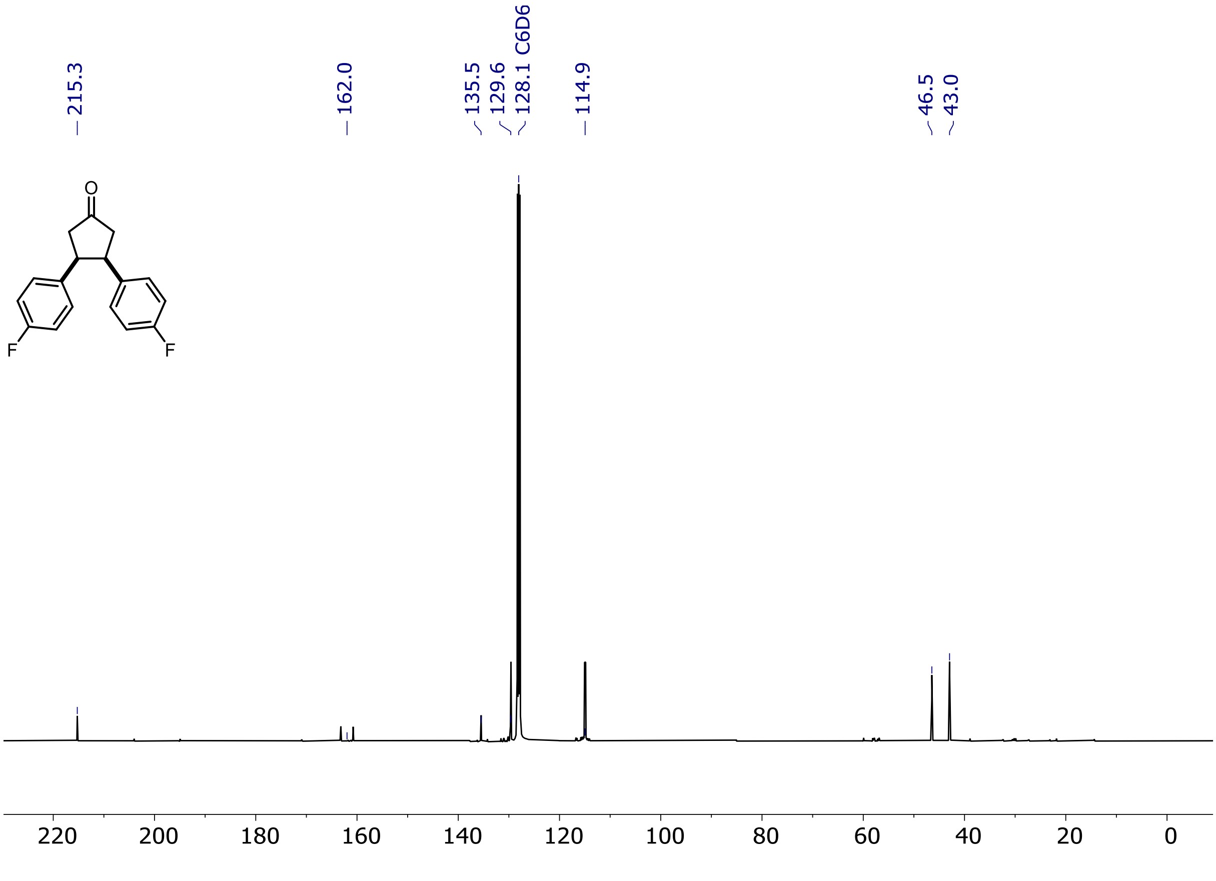

[S4p] ^{19}F, C_6D_6, 282 MHz

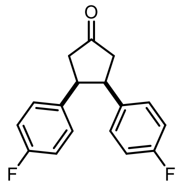

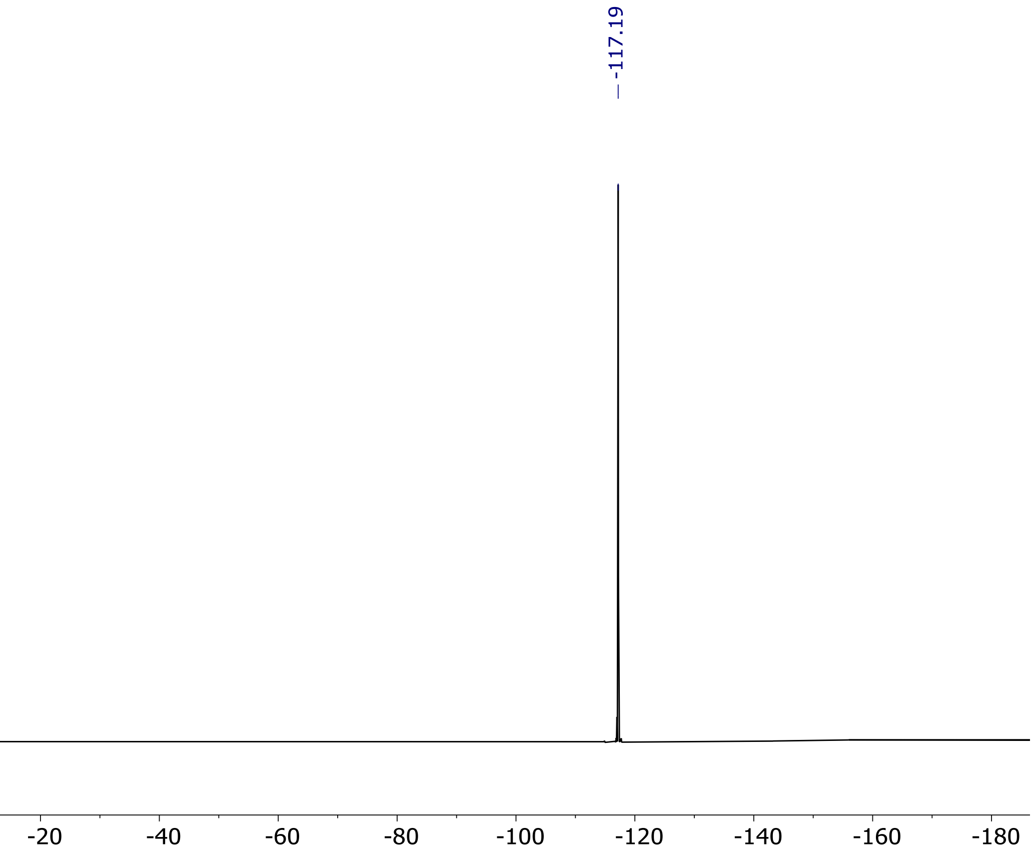

[8a] ^{1}H, $CDCl_3$, 400 MHz

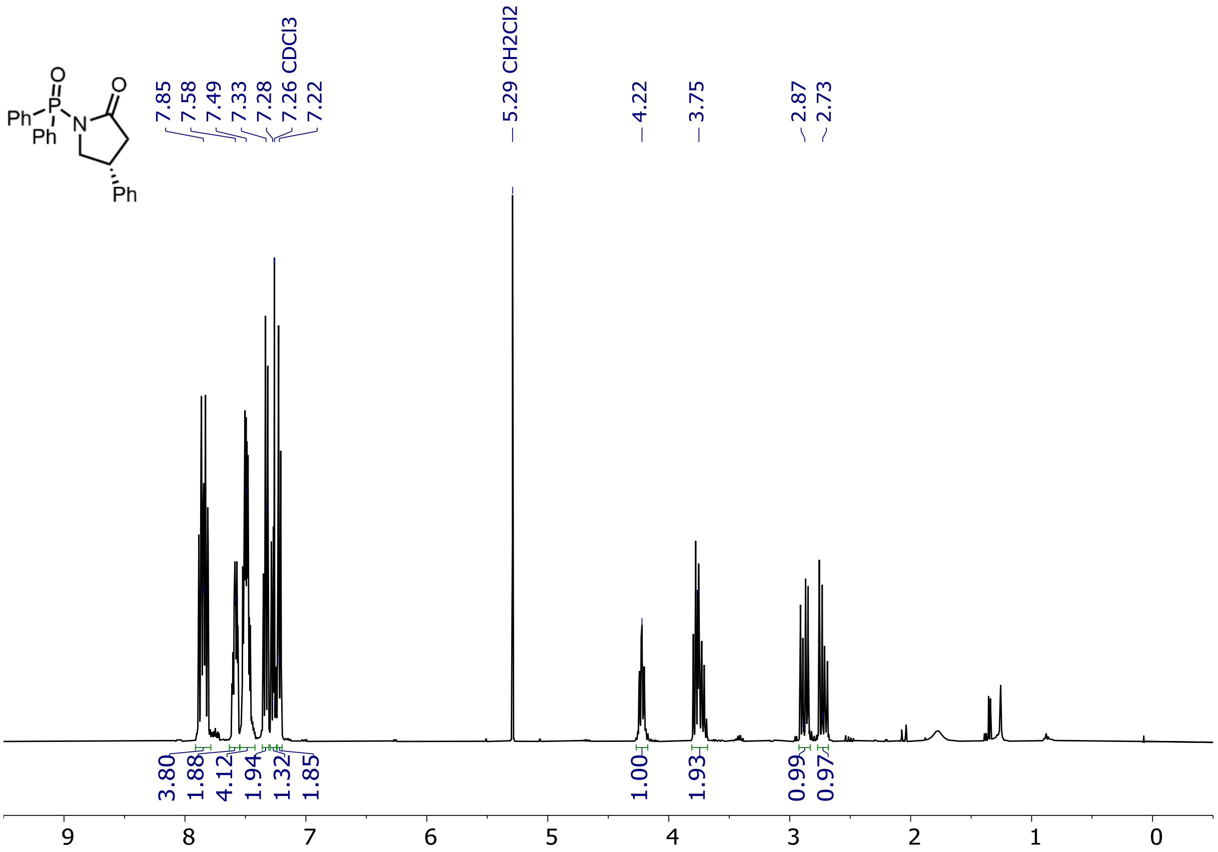

[8a] ^{13}C, $CDCl_3$, 101 MHz

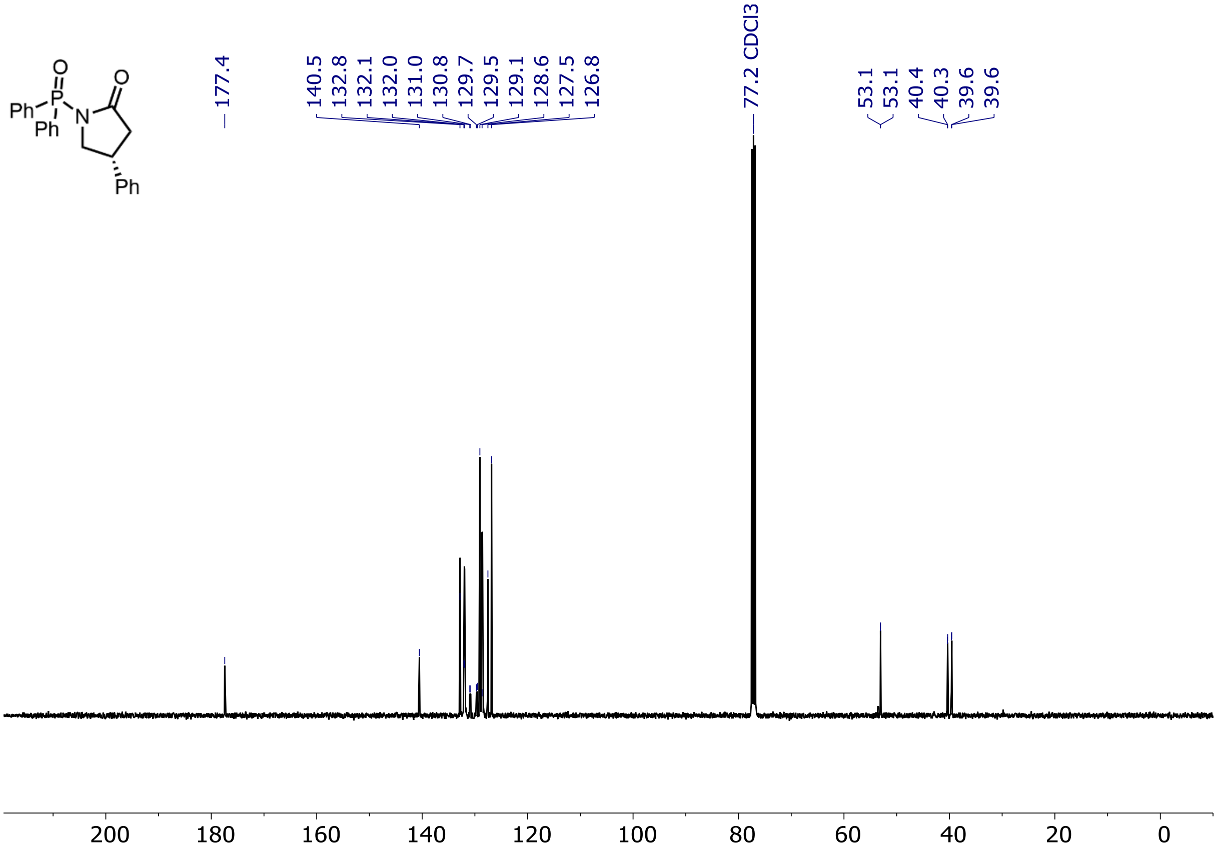

[8a] ^{31}P, $CDCl_3$, 162 MHz

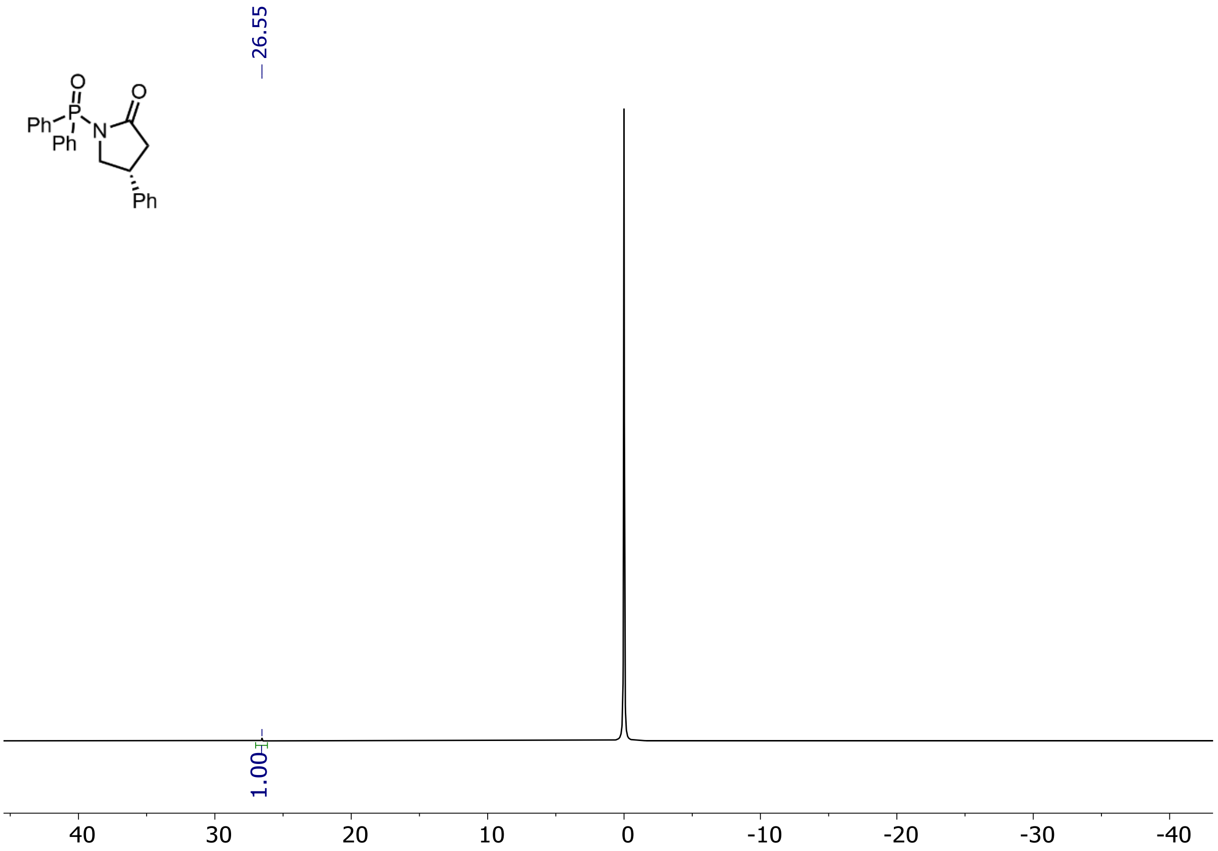

[8a] COSY, $CDCl_3$, 400 MHz

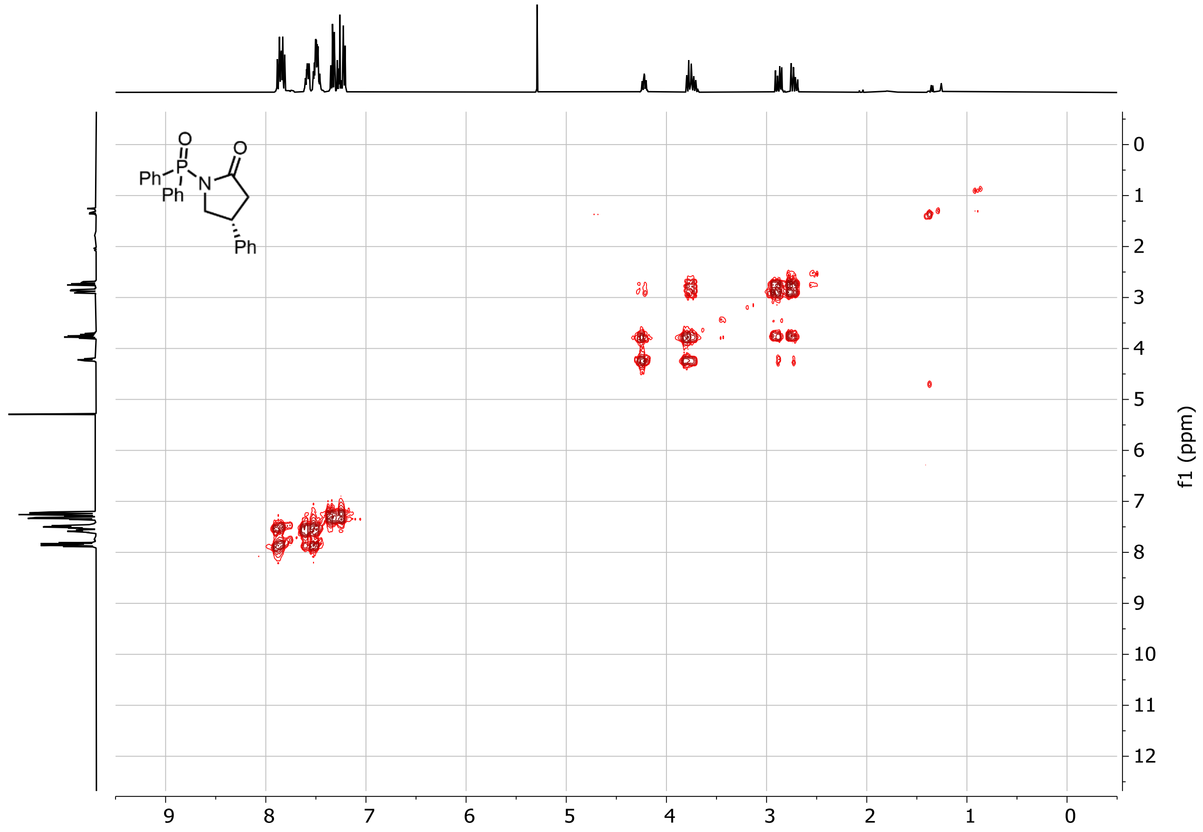

[8a] NOESY, $CDCl_3$, 400 MHz

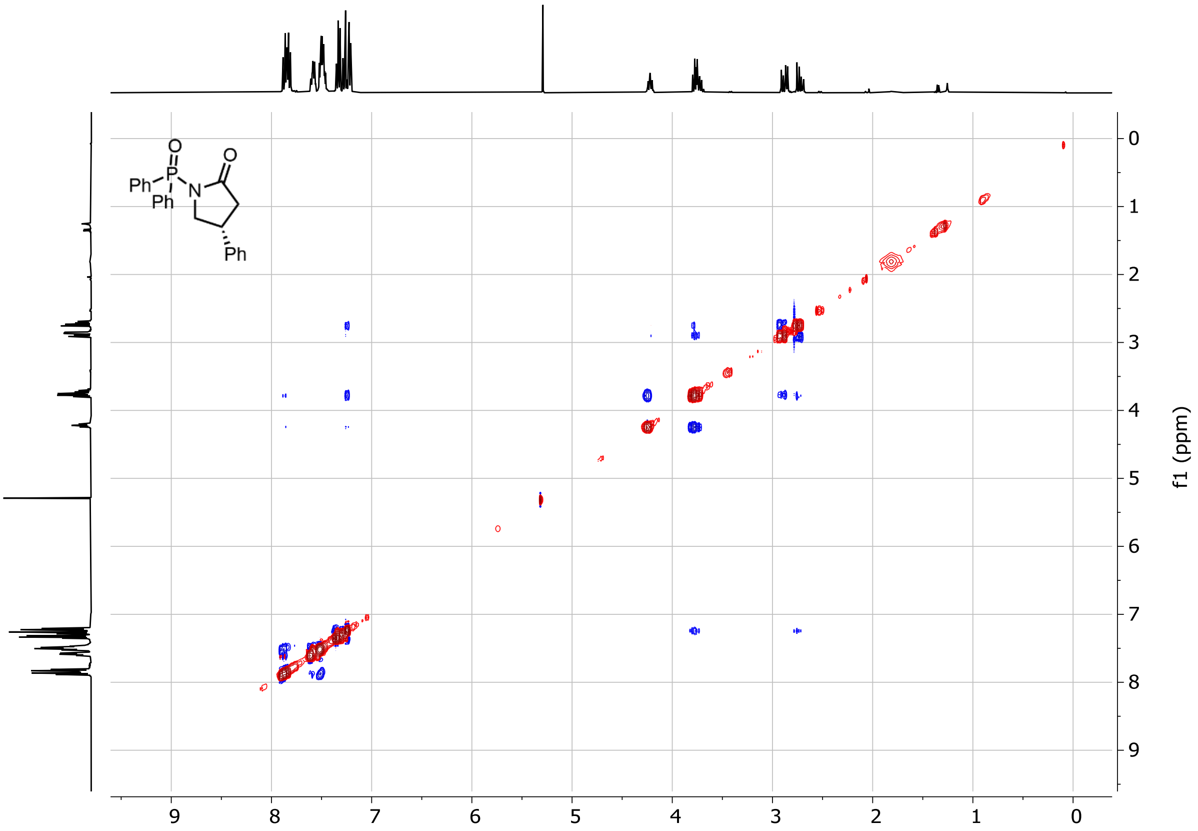

[2a] ^{1}H, $CDCl_3$, 400 MHz

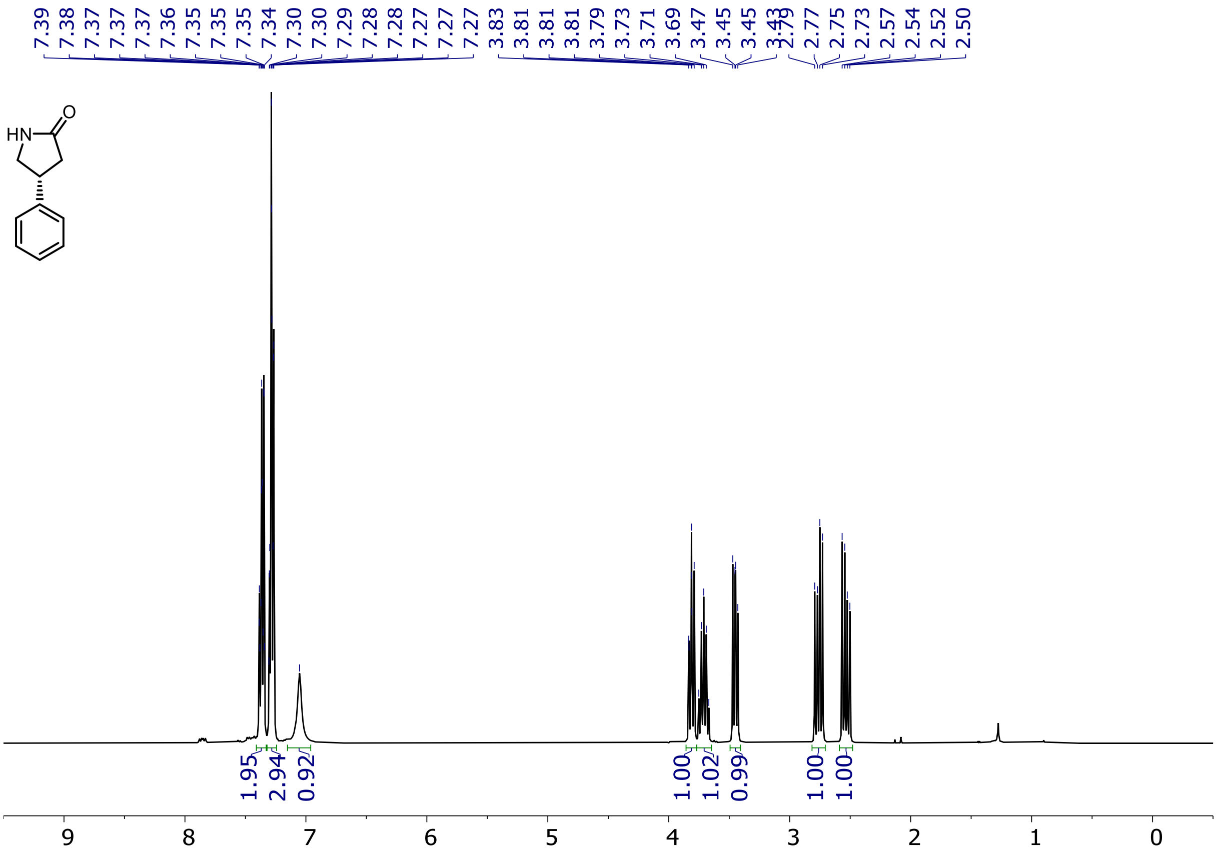

[2a] ^{13}C, $CDCl_3$, 101 MHz

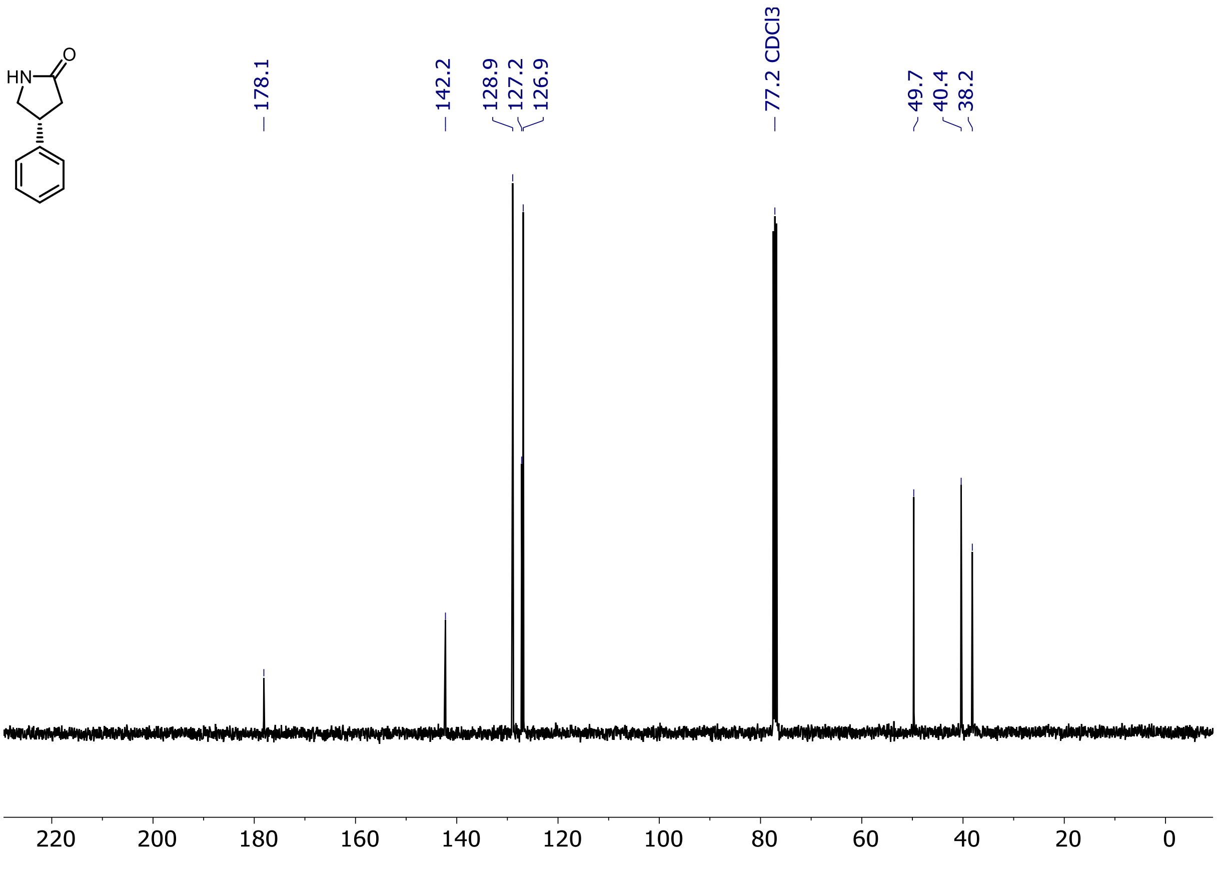

[2b] ^1H, $CDCl_3$, 400 MHz

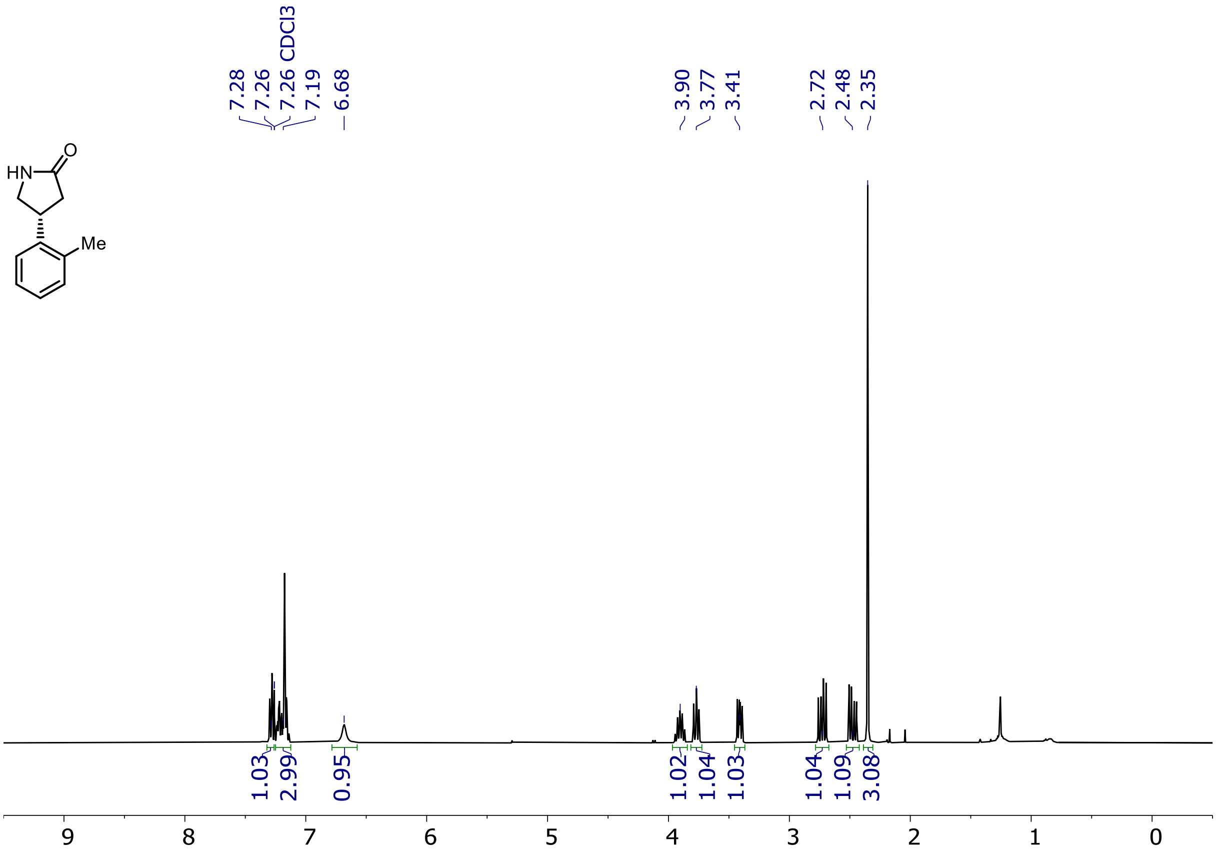

[2b] ^{13}C, $CDCl_3$, 101 MHz

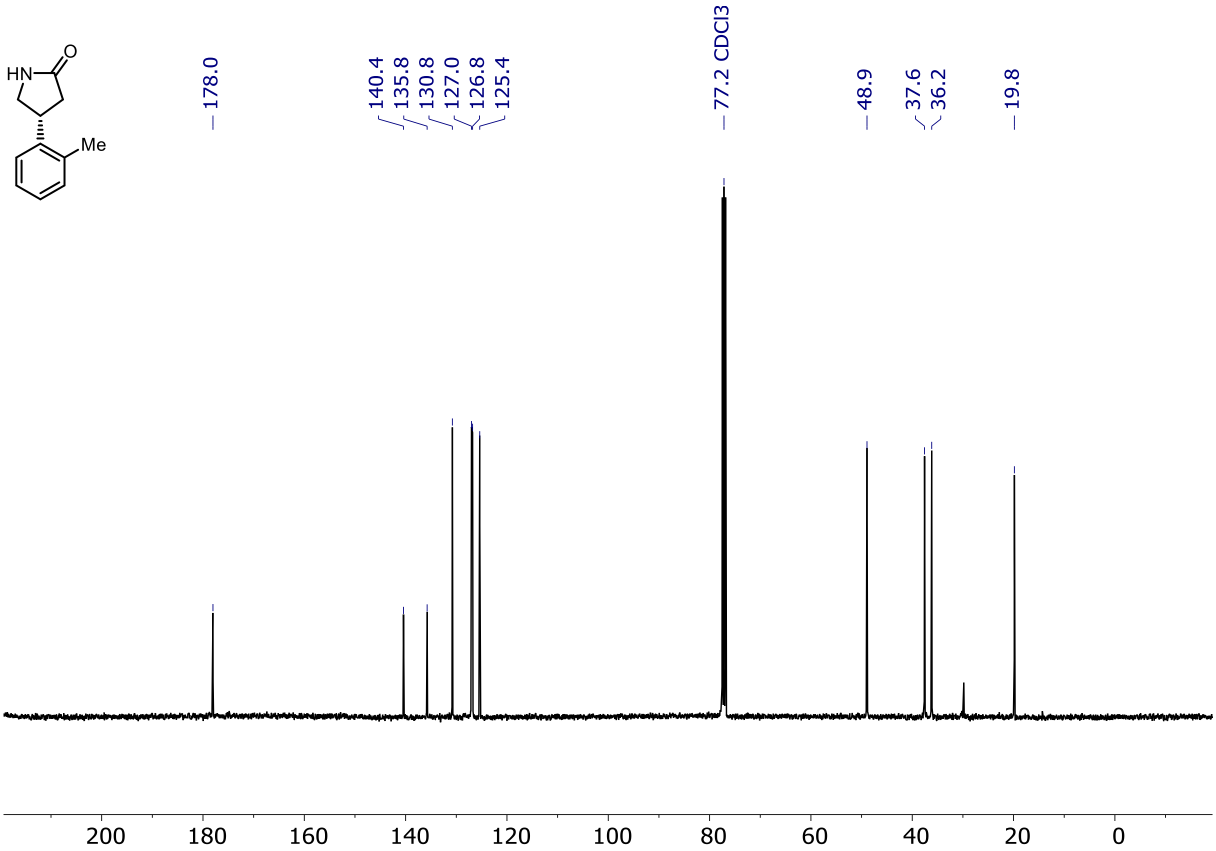

[2c] ^{1}H, $CDCl_3$, 400 MHz

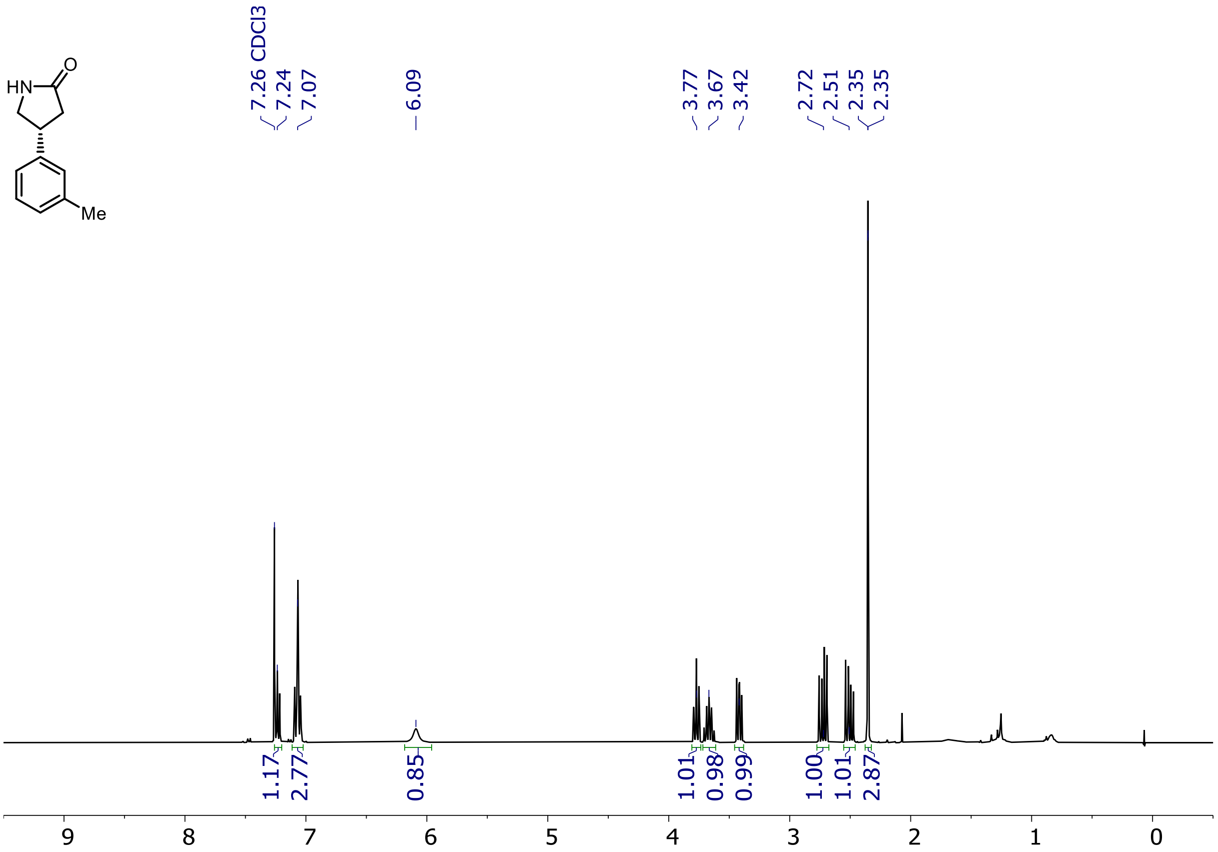

[2c] ^{13}C, $CDCl_3$, 101 MHz

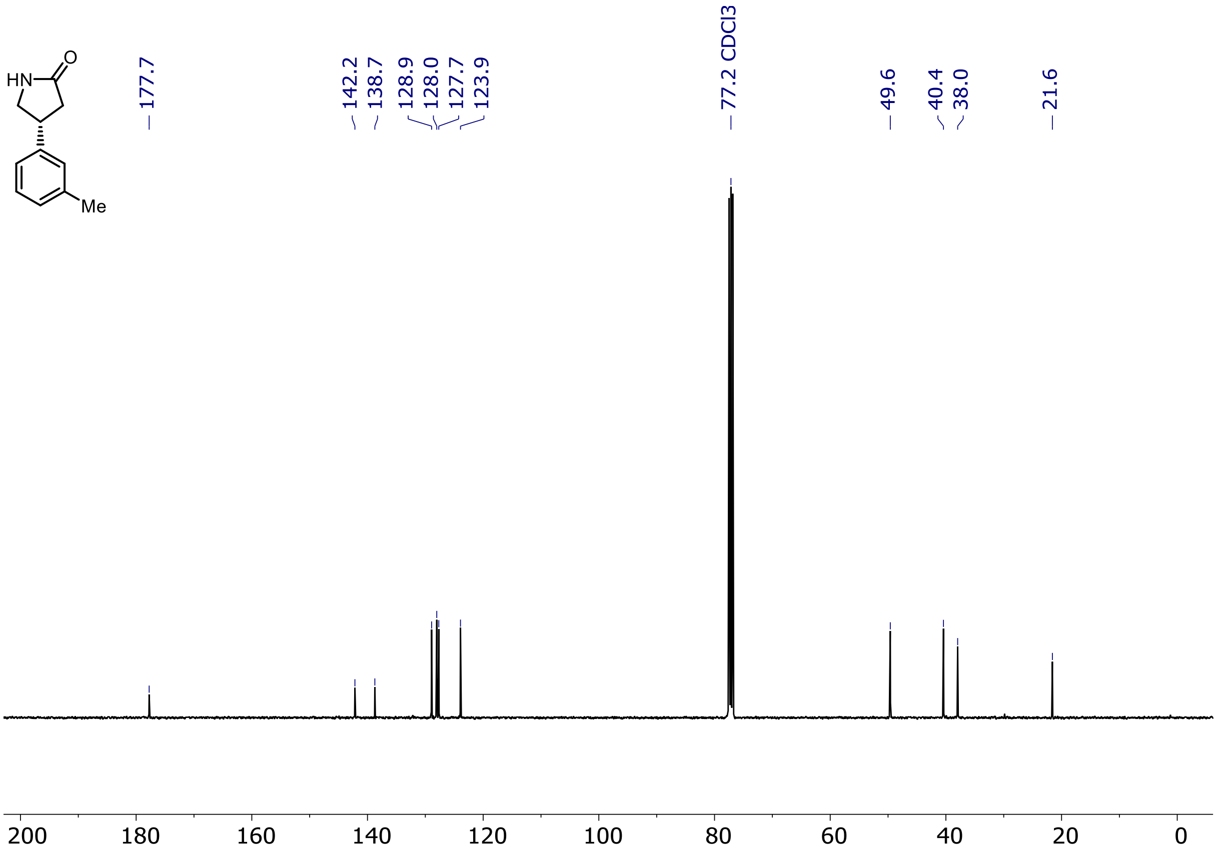

[2d] ^{1}H, $CDCl_3$, 400 MHz

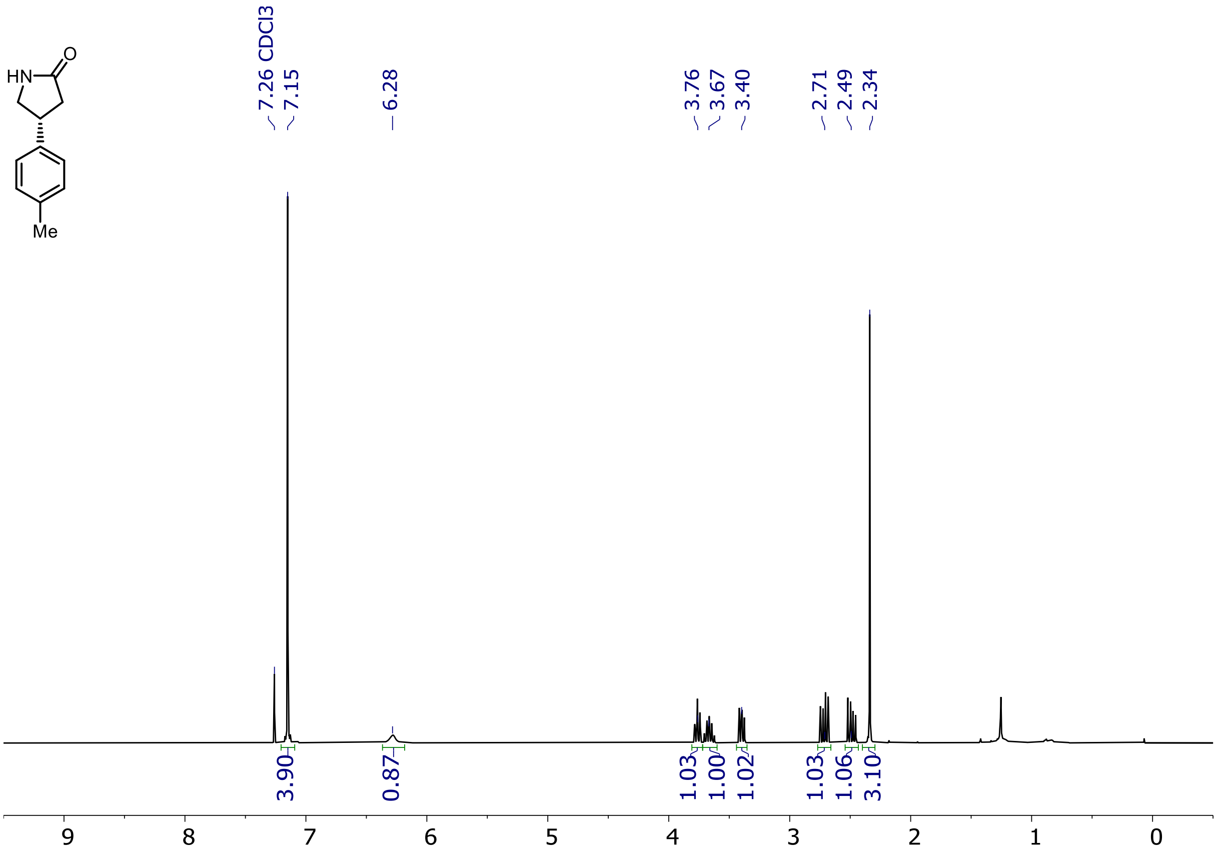

[2d] ^{13}C, $CDCl_3$, 101 MHz

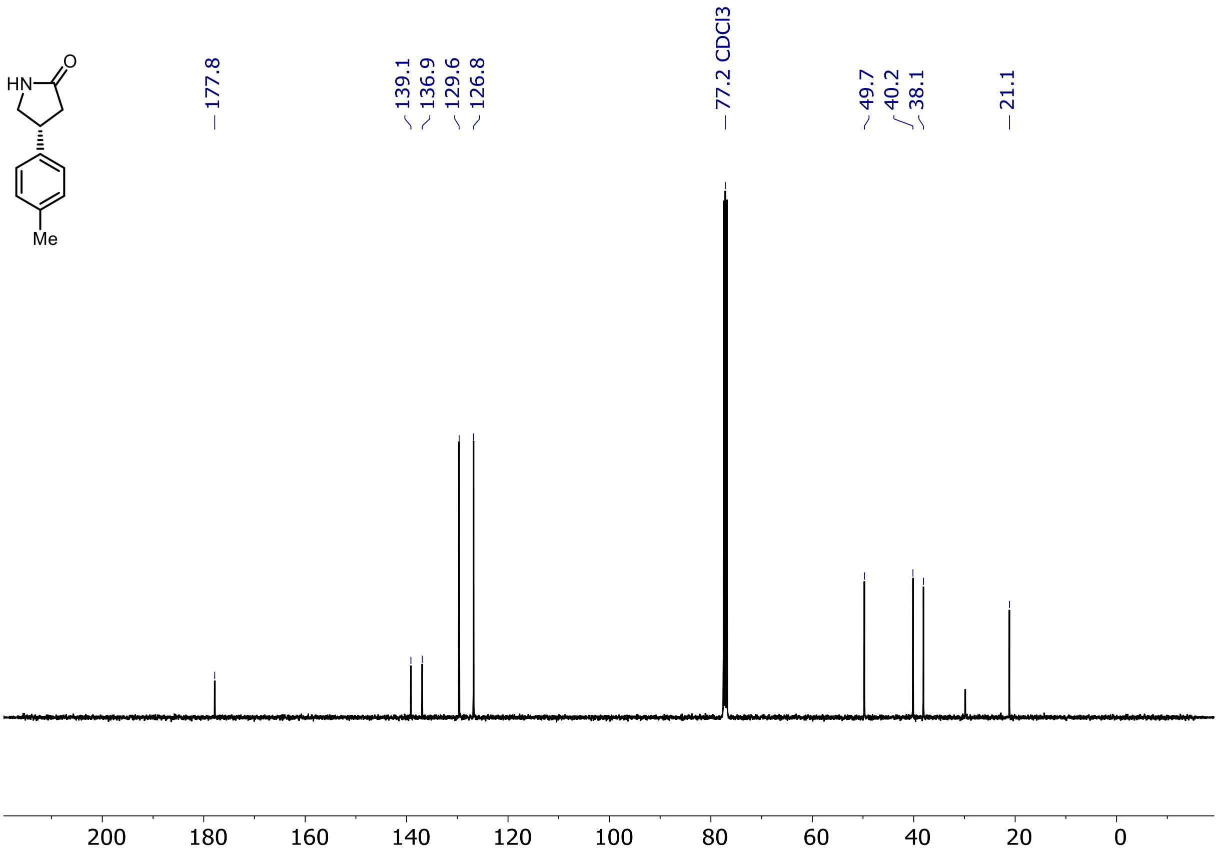

[2e] ^{1}H, $CDCl_3$, 400 MHz

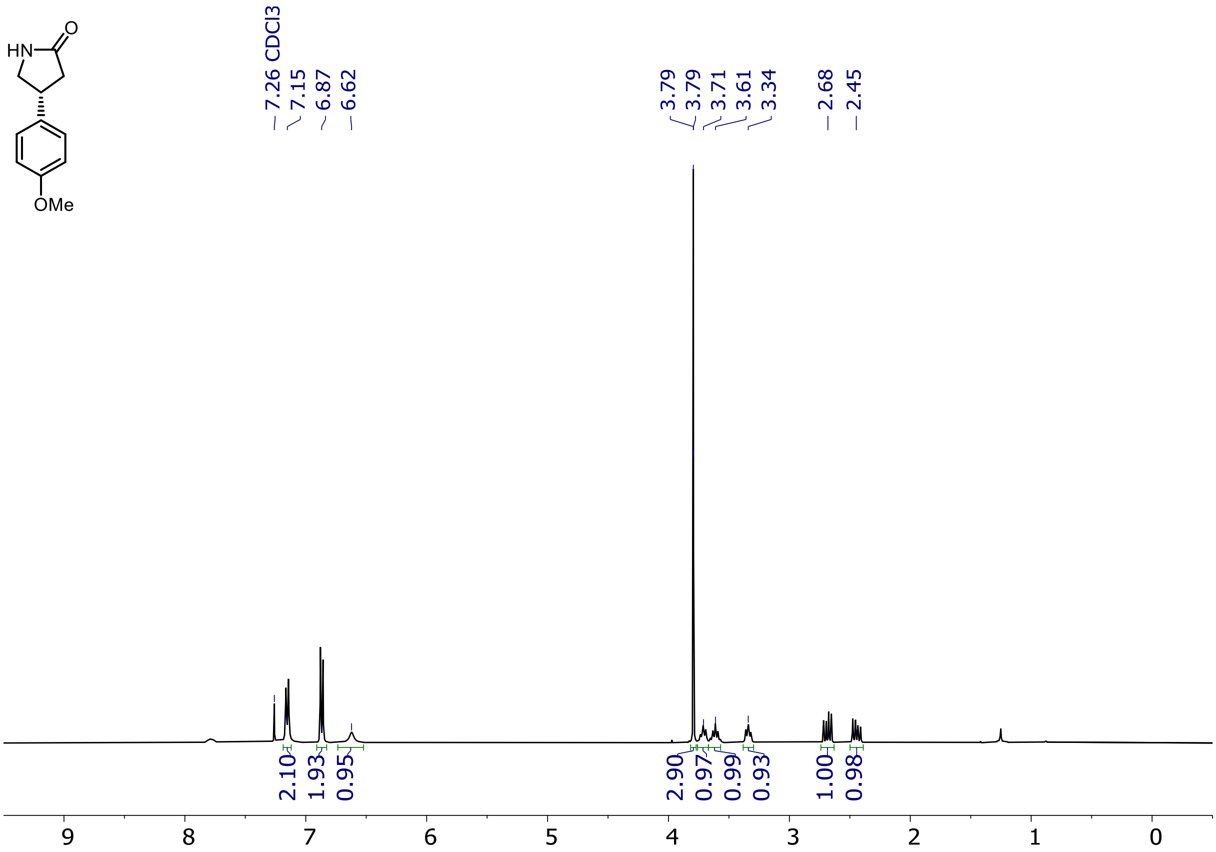

[2e] ^{13}C, $CDCl_3$, 101 MHz

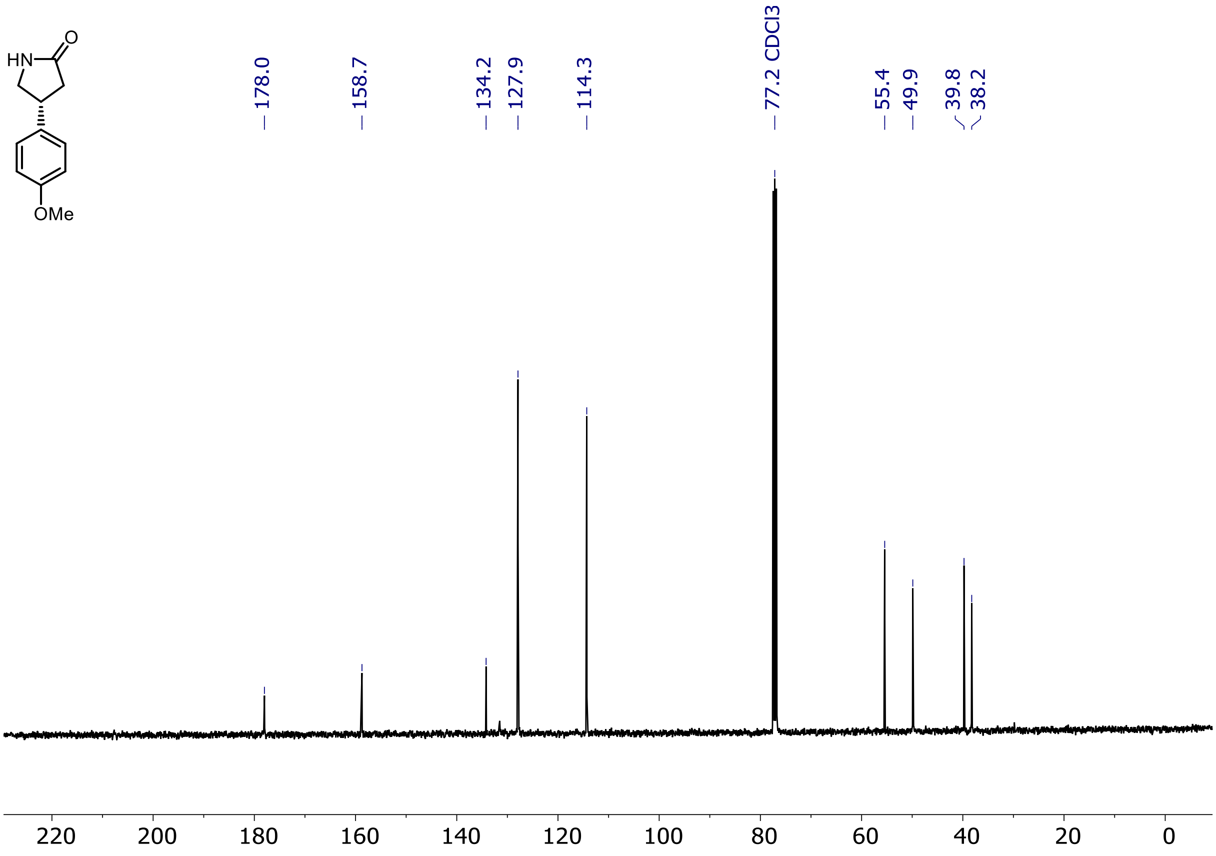

[2f] ^{1}H, $CDCl_3$, 400 MHz

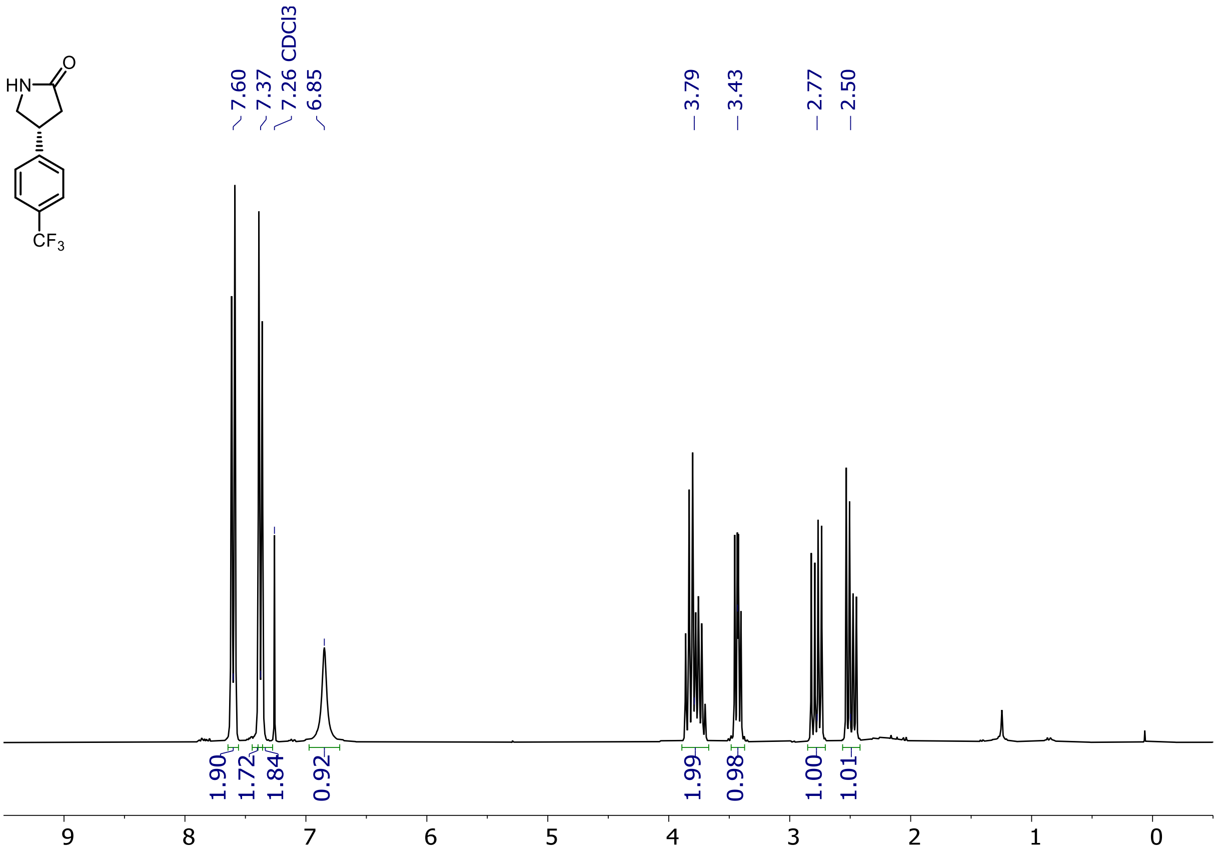

[2f] ^{13}C, $CDCl_3$, 101 MHz

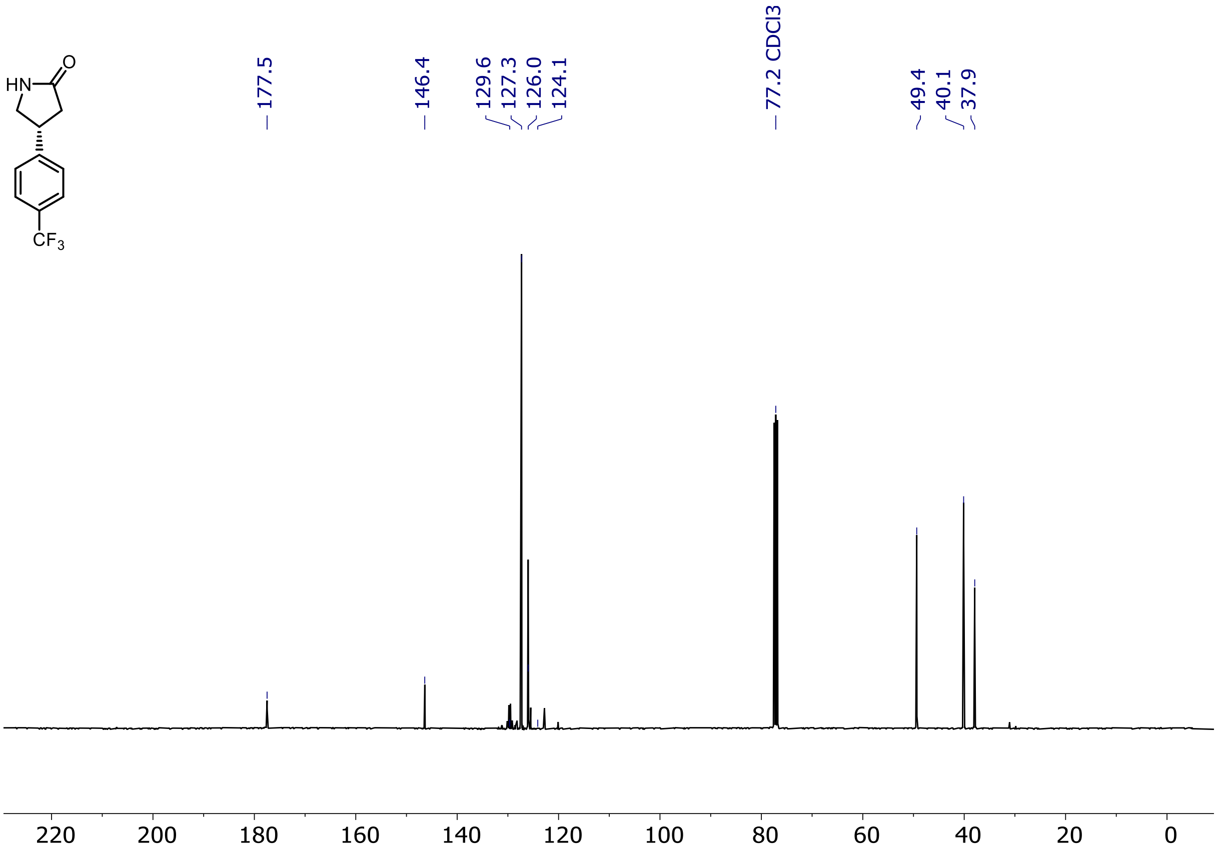

[2f] ^{19}F, $CDCl_3$, 377 MHz

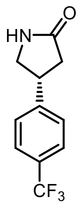

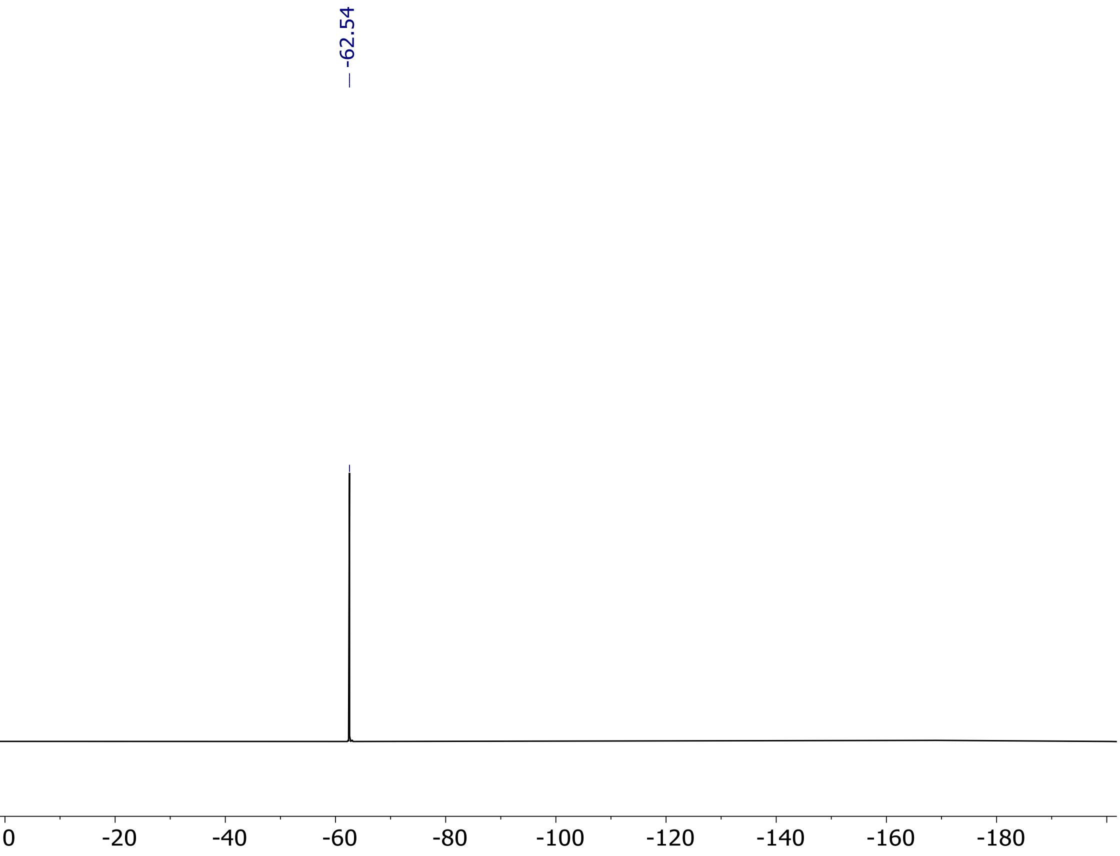

[2g] ^1H, $CDCl_3$, 400 MHz

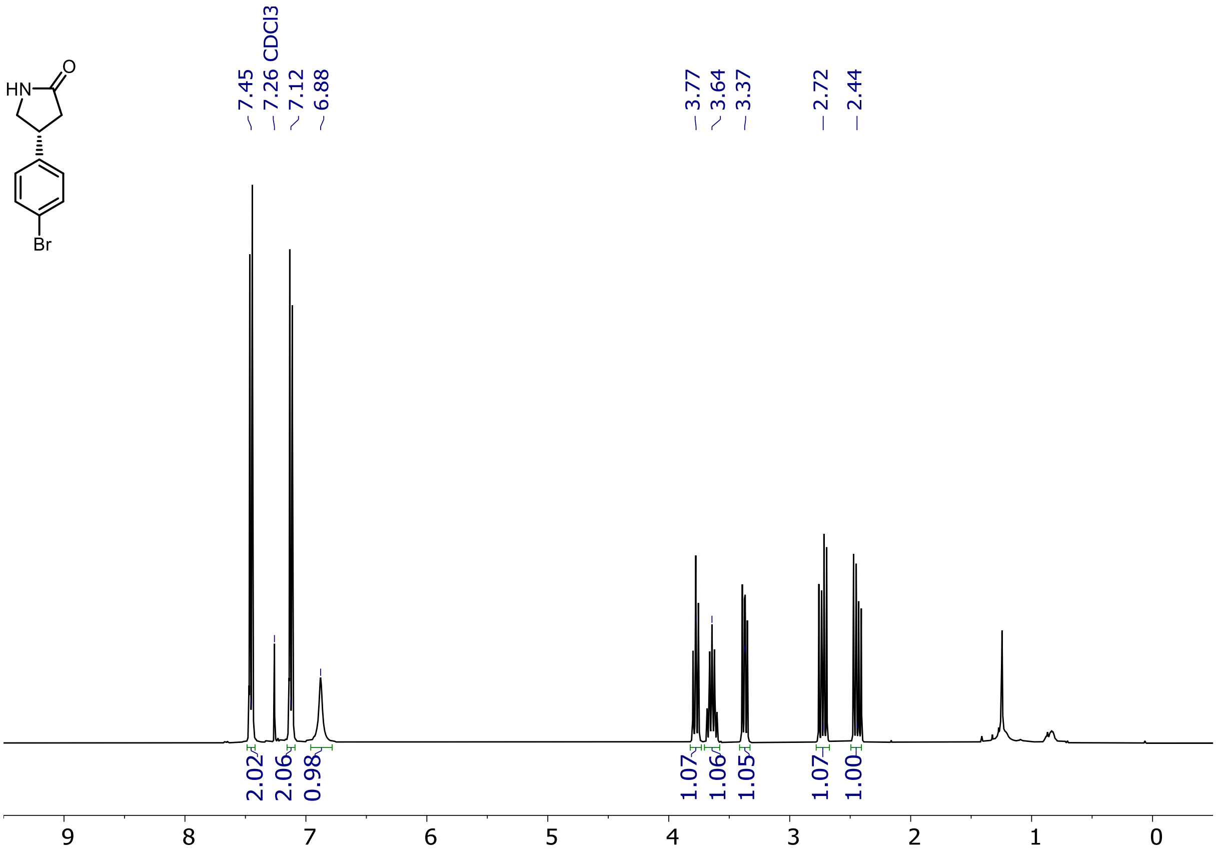

[2g] ^{13}C, $CDCl_3$, 101 MHz

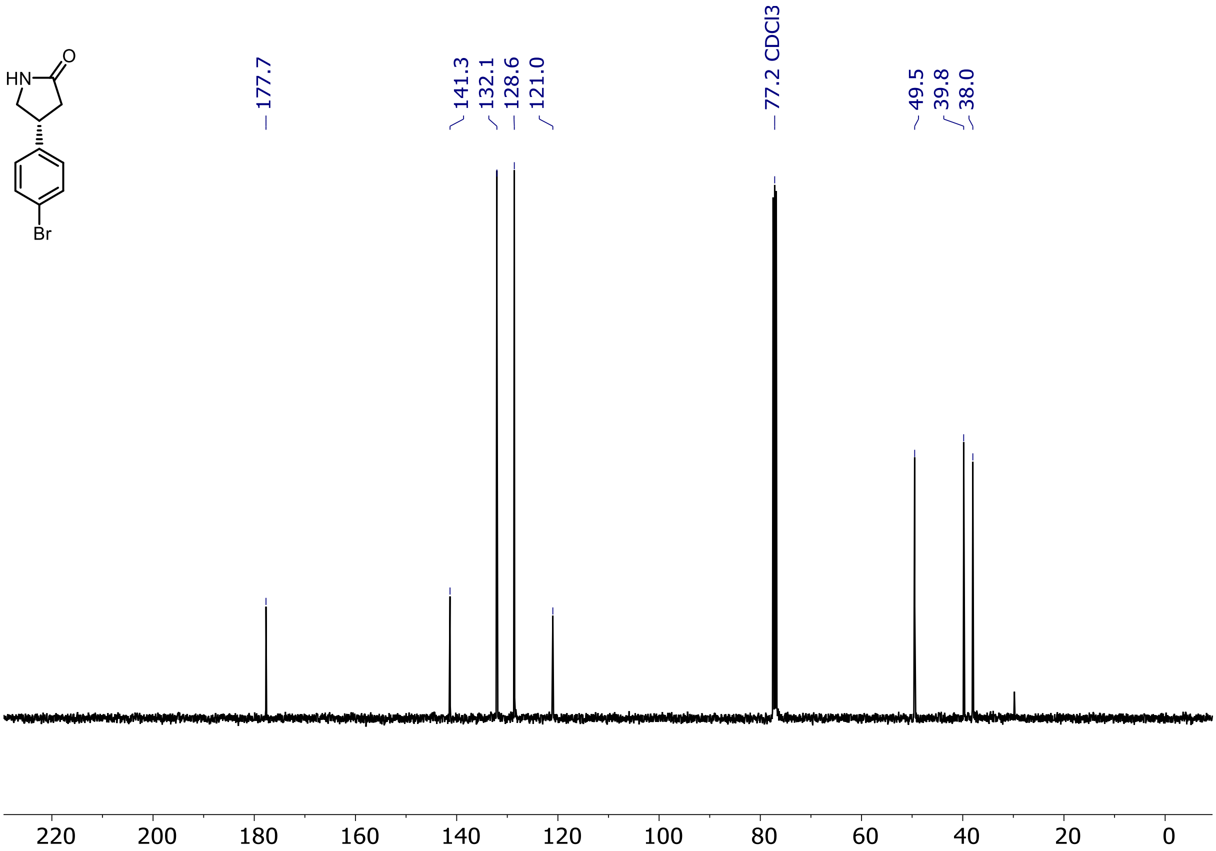

[2h] ^{1}H, $CDCl_3$, 400 MHz

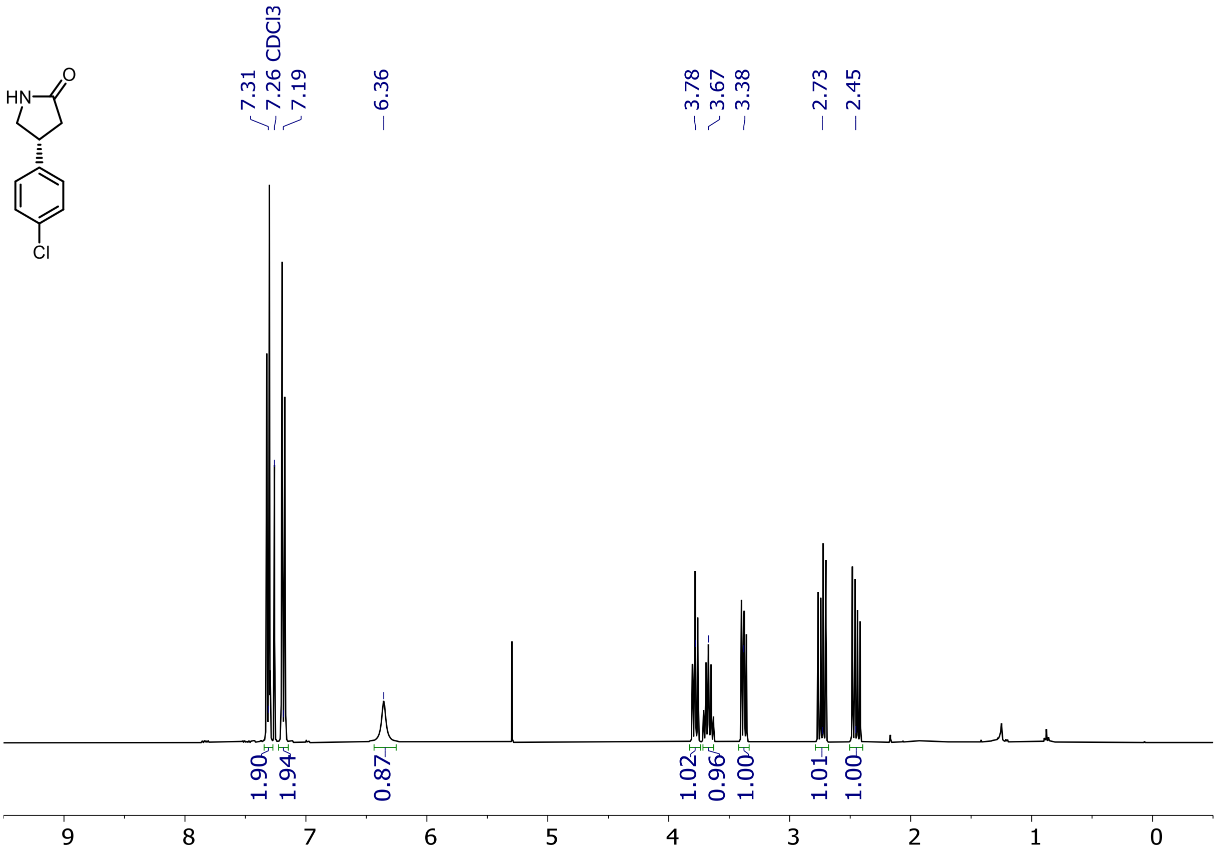

[2h] ^{13}C, $CDCl_3$, 101 MHz

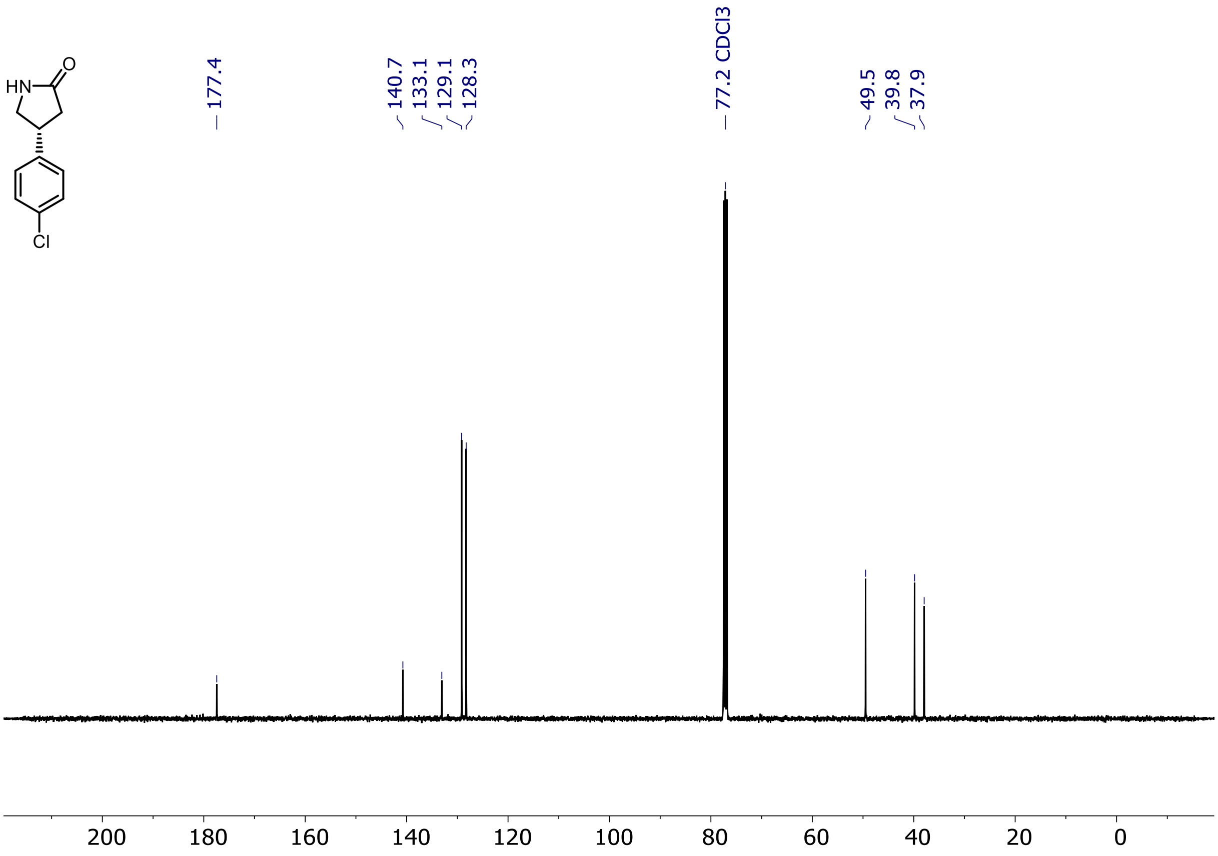

[2i] ^{1}H, $CDCl_3$, 400 MHz

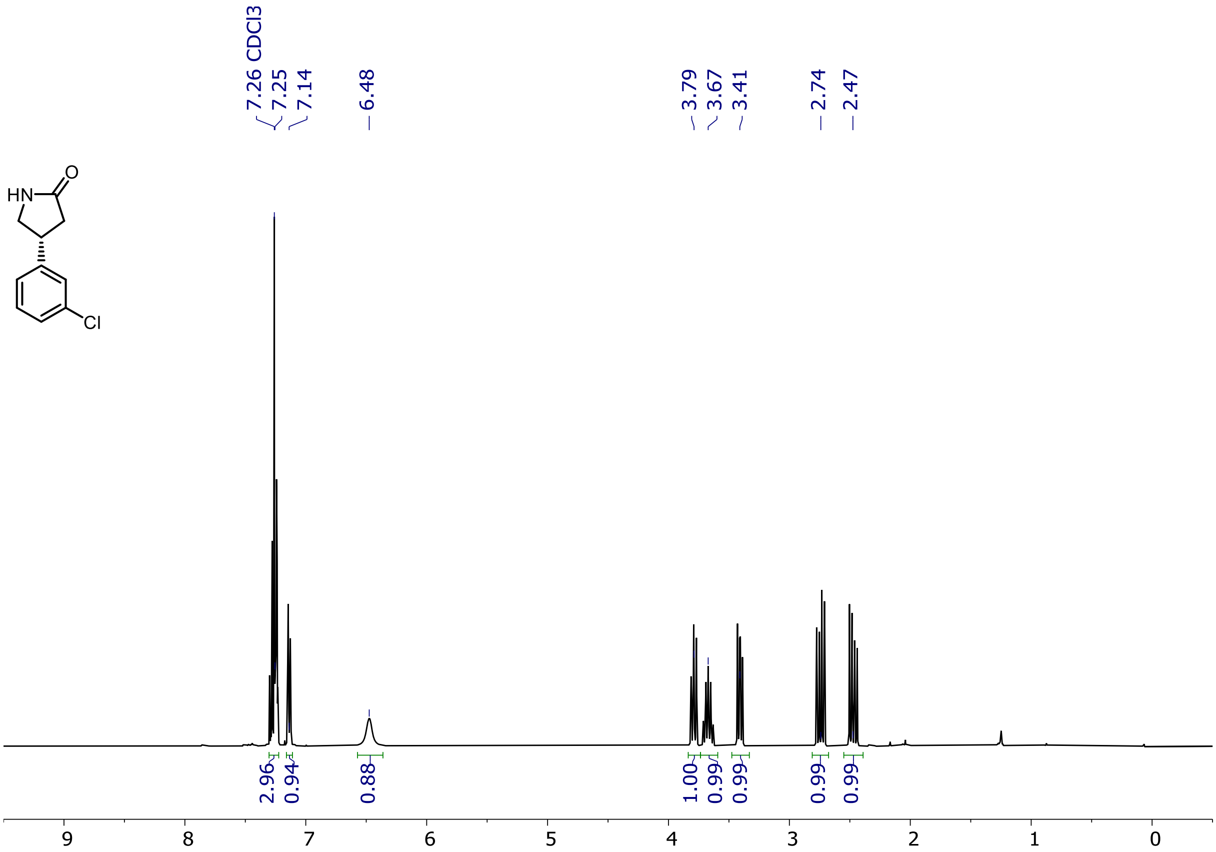

[2i] ^{13}C, $CDCl_3$, 101 MHz

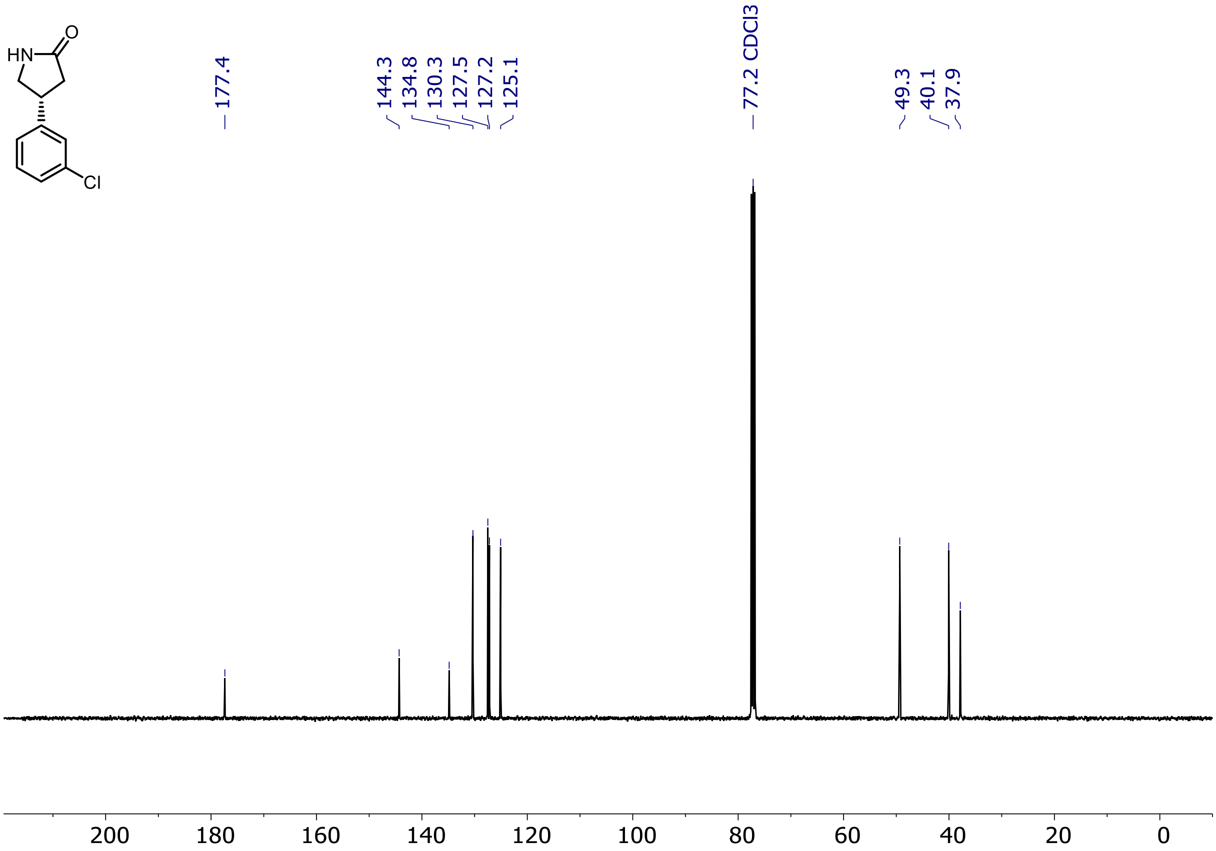

[2j] ^{1}H, $CDCl_3$, 400 MHz

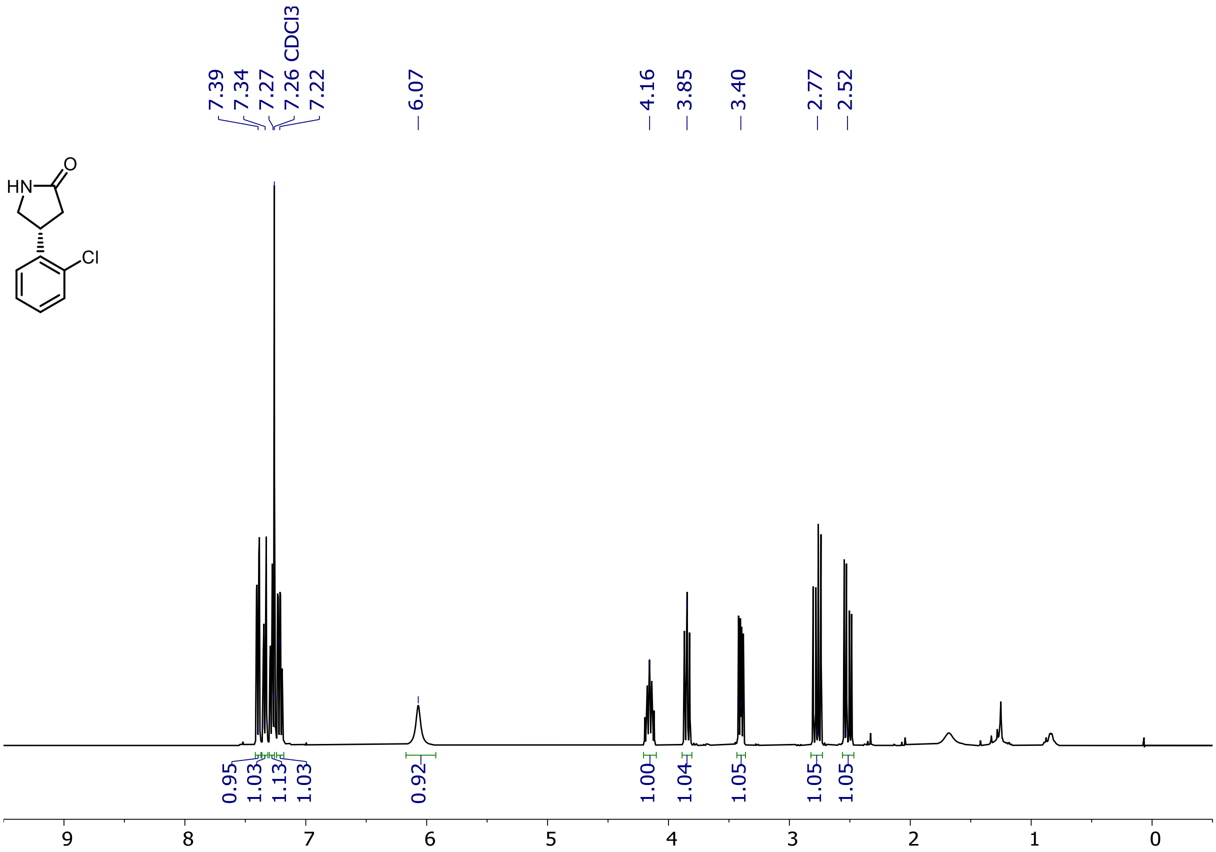

[2j] ^{13}C, $CDCl_3$, 101 MHz

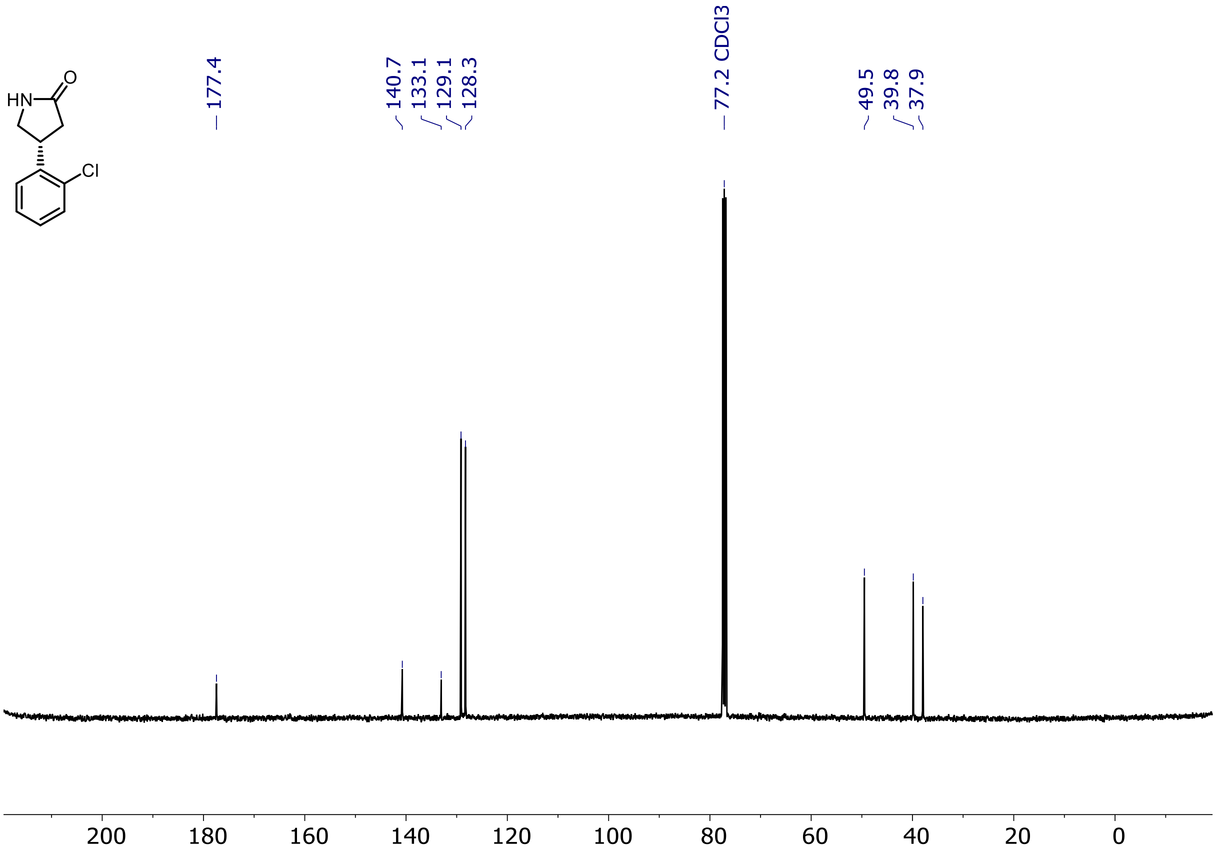

[2k] ^1H, $CDCl_3$, 400 MHz

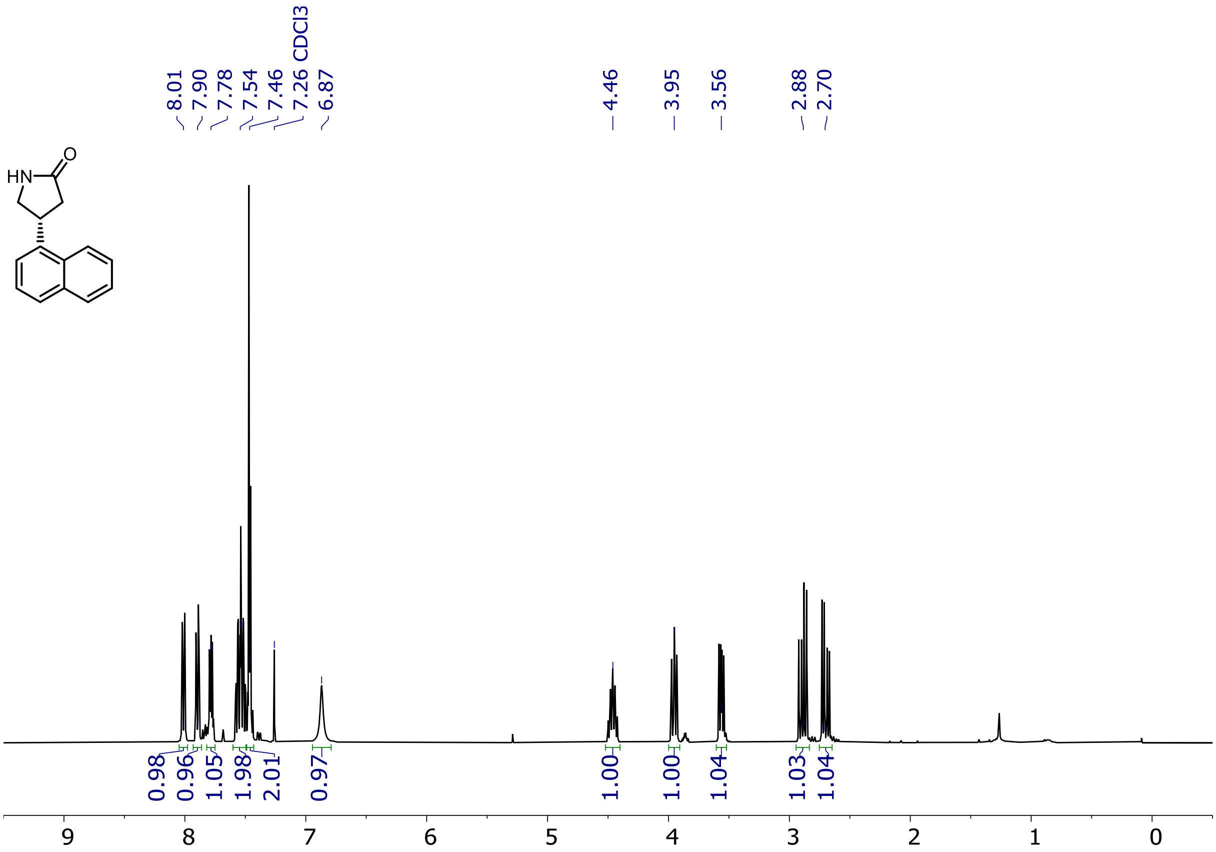

[2k] ^{13}C, $CDCl_3$, 101 MHz

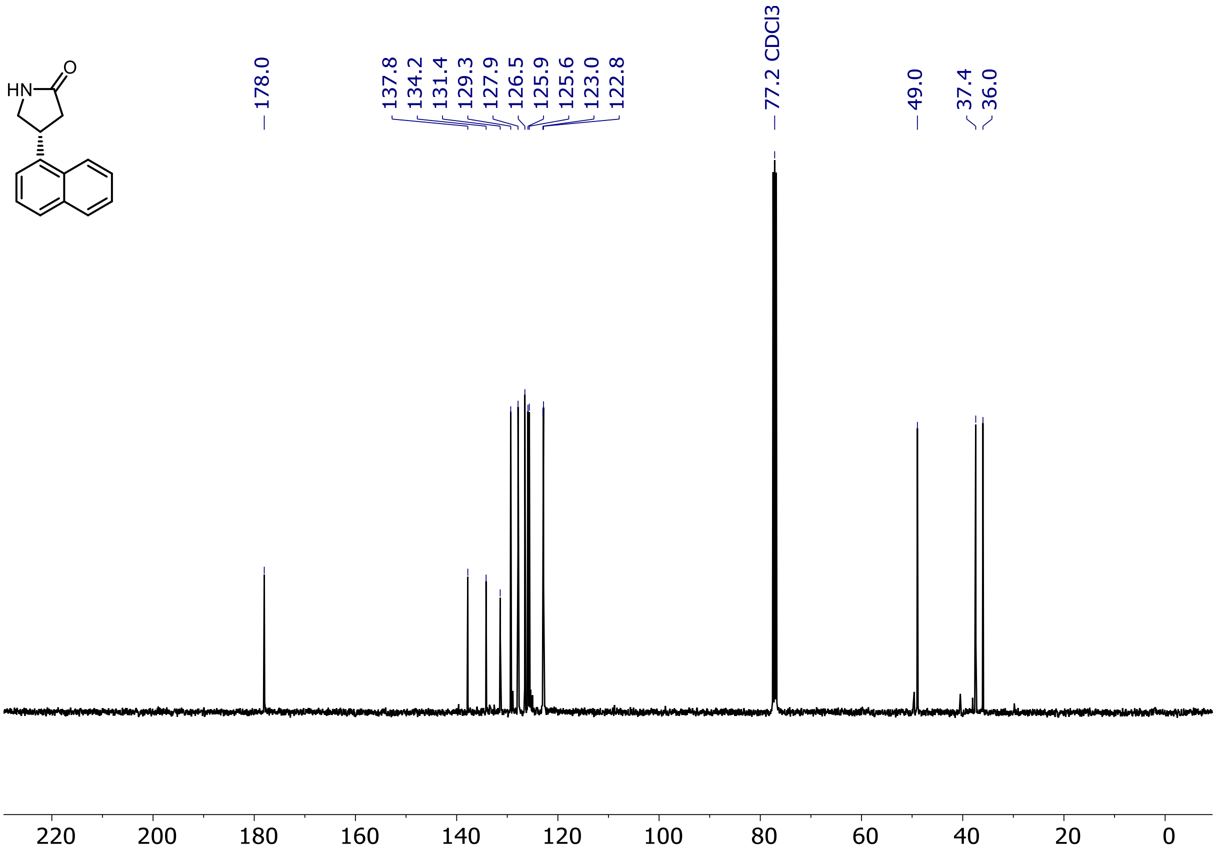

[2l] ^{1}H, $CDCl_3$, 400 MHz

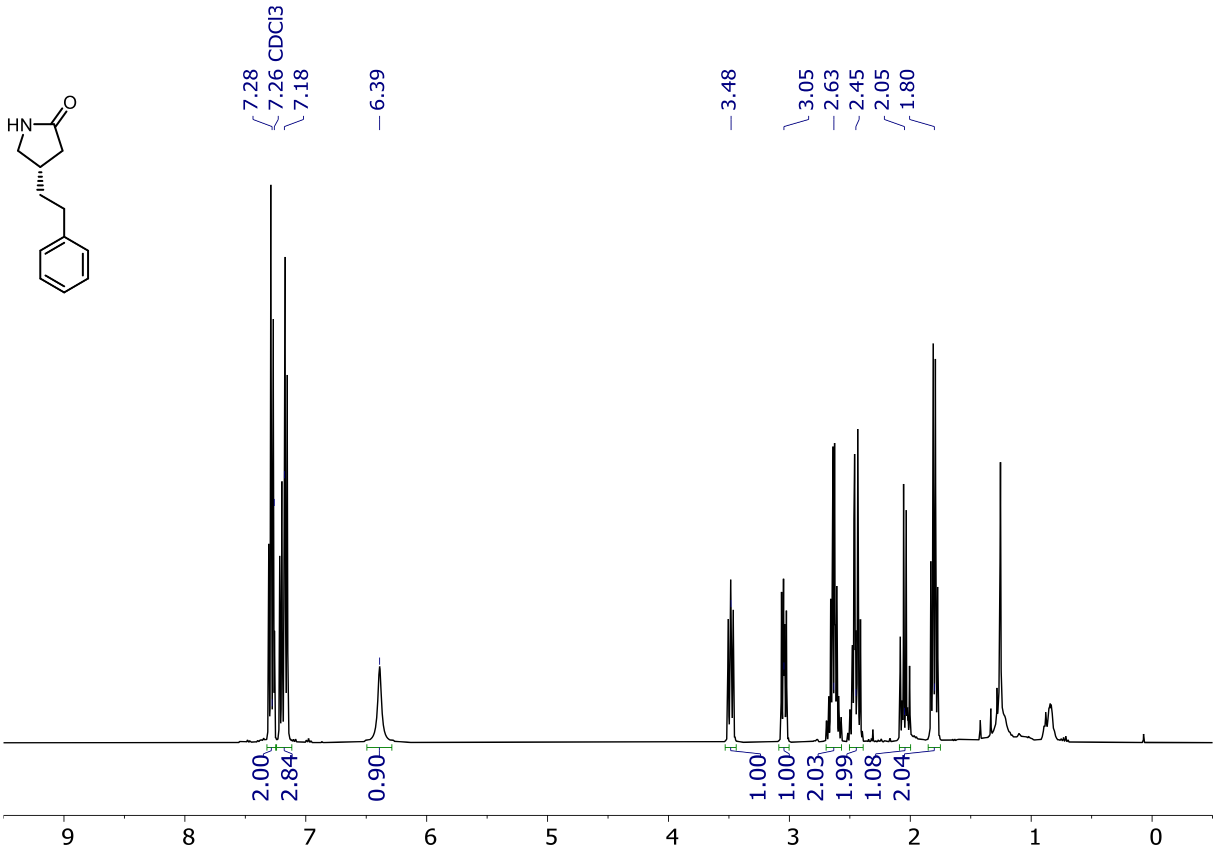

[2l] ^{13}C, $CDCl_3$, 101 MHz

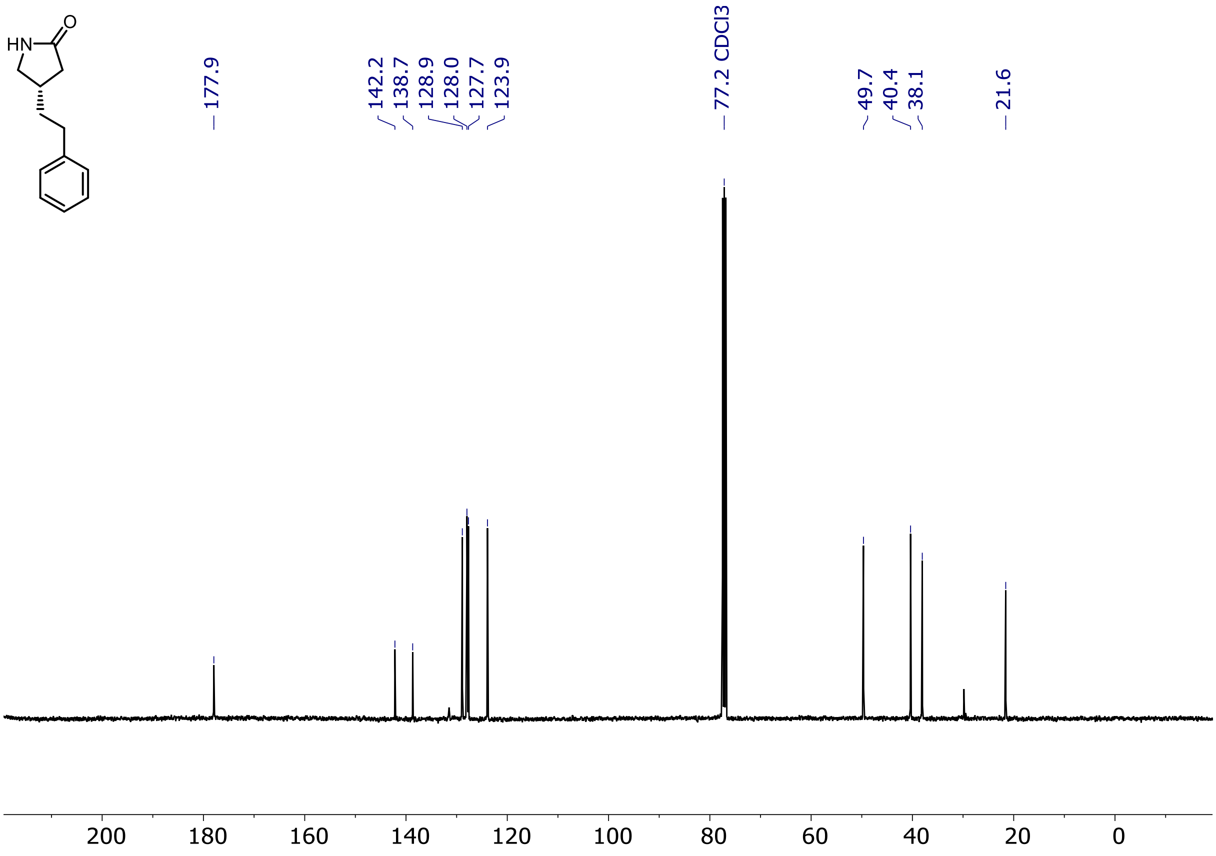

[2m] ^{1}H, $CDCl_3$, 400 MHz

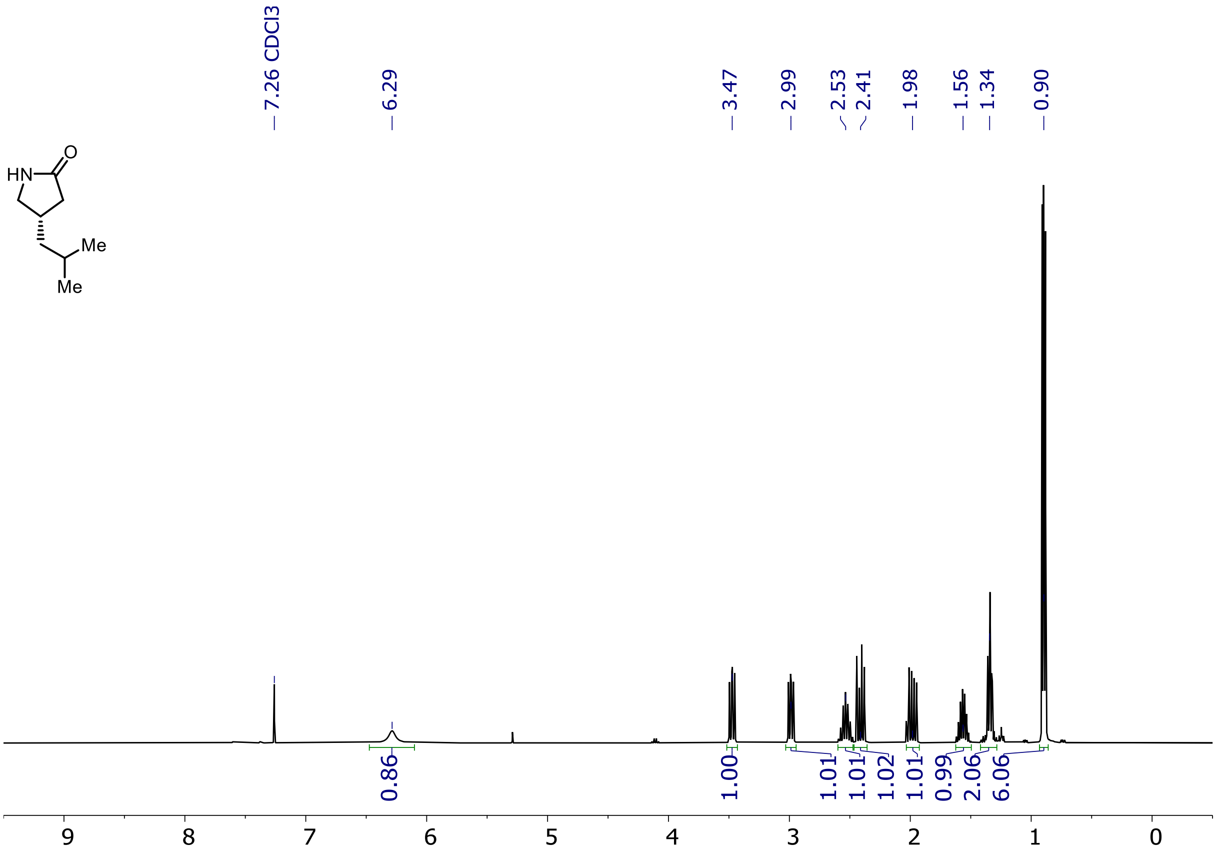

[2m] ^{13}C, $CDCl_3$, 101 MHz

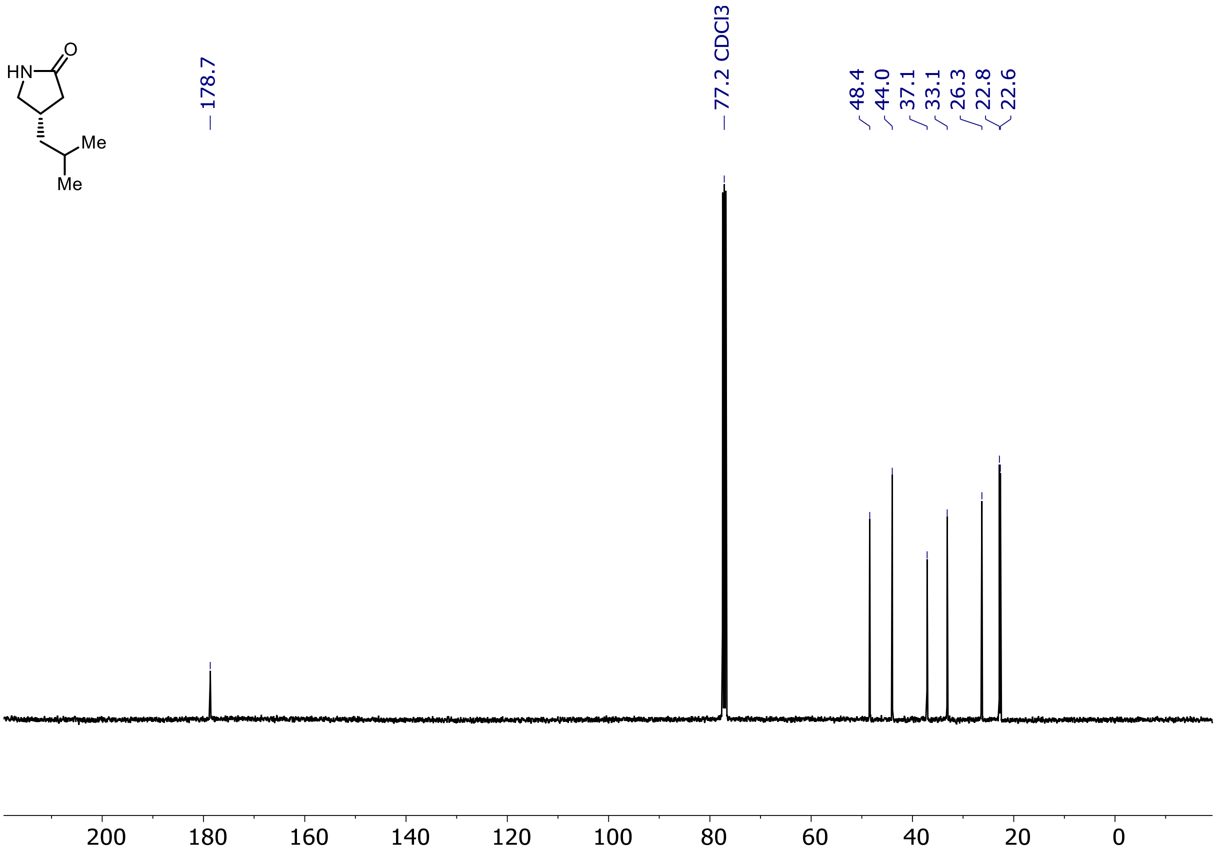

[6n] ^{1}H, $CDCl_3$, 400 MHz

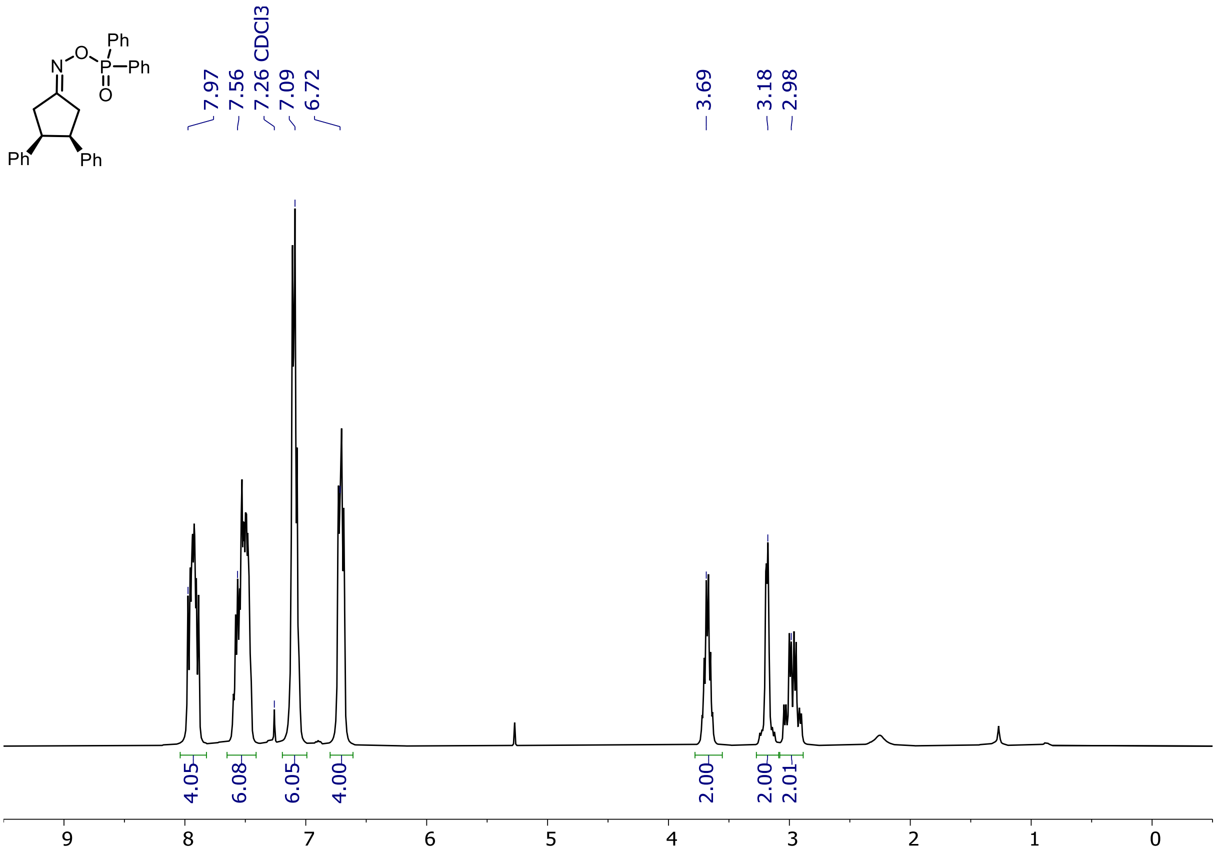

[6n] ^{13}C, $CDCl_3$, 101 MHz

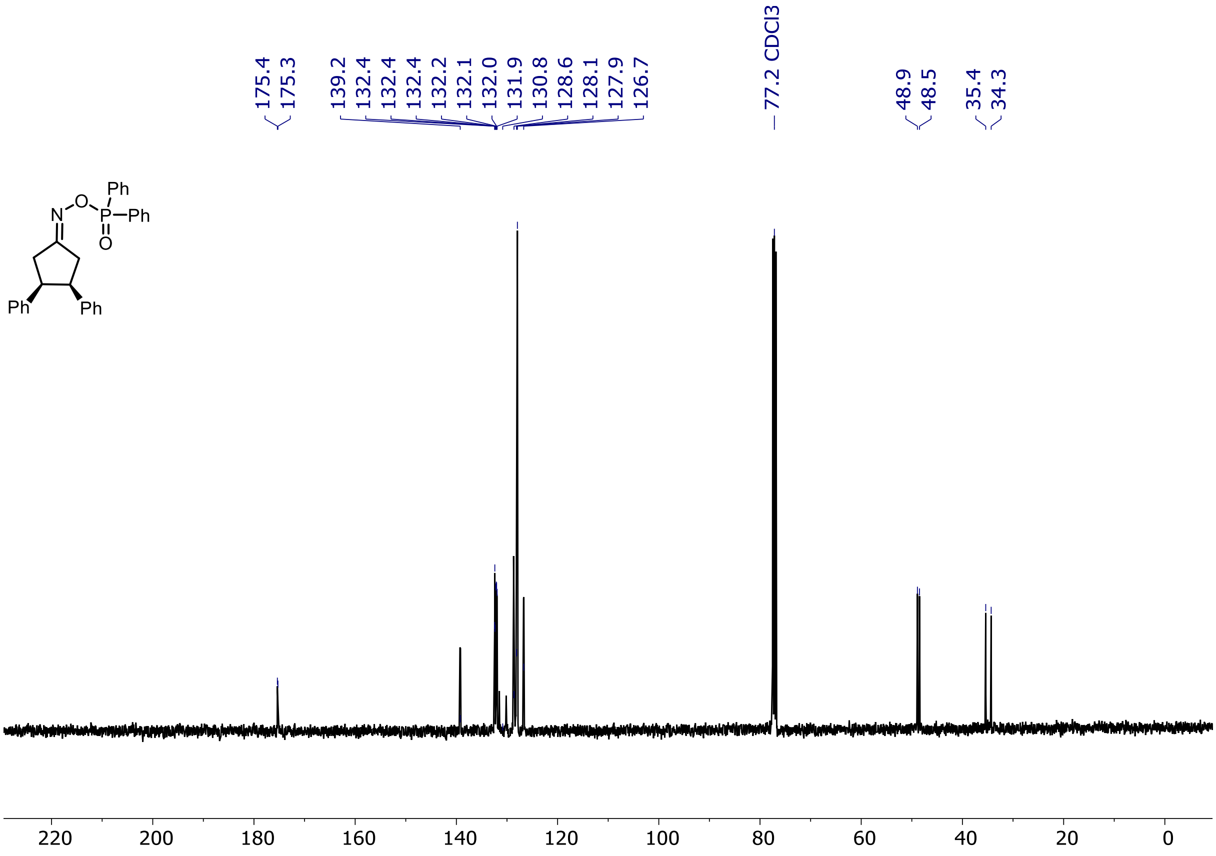

[6n] ^{31}P, $CDCl_3$, 162 MHz

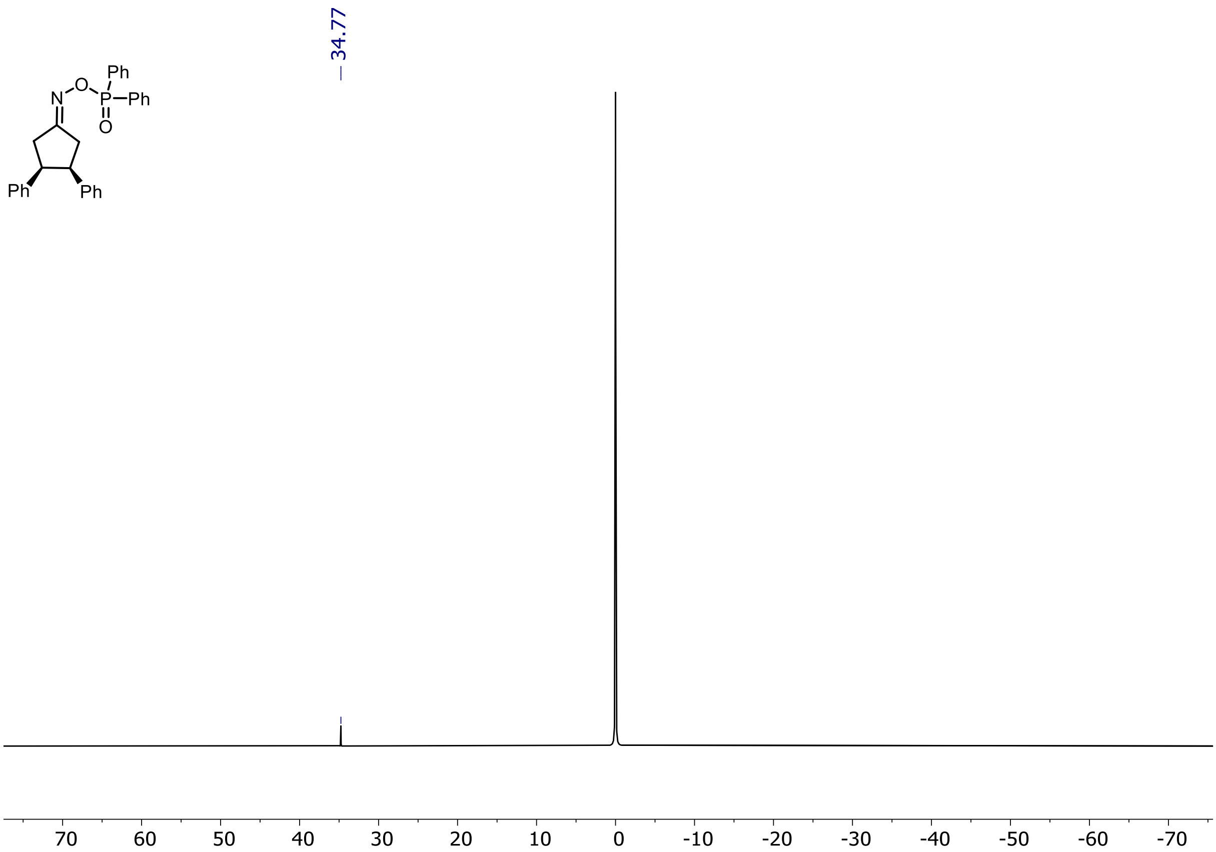

[2n] ^{1}H, $CDCl_3$, 400 MHz

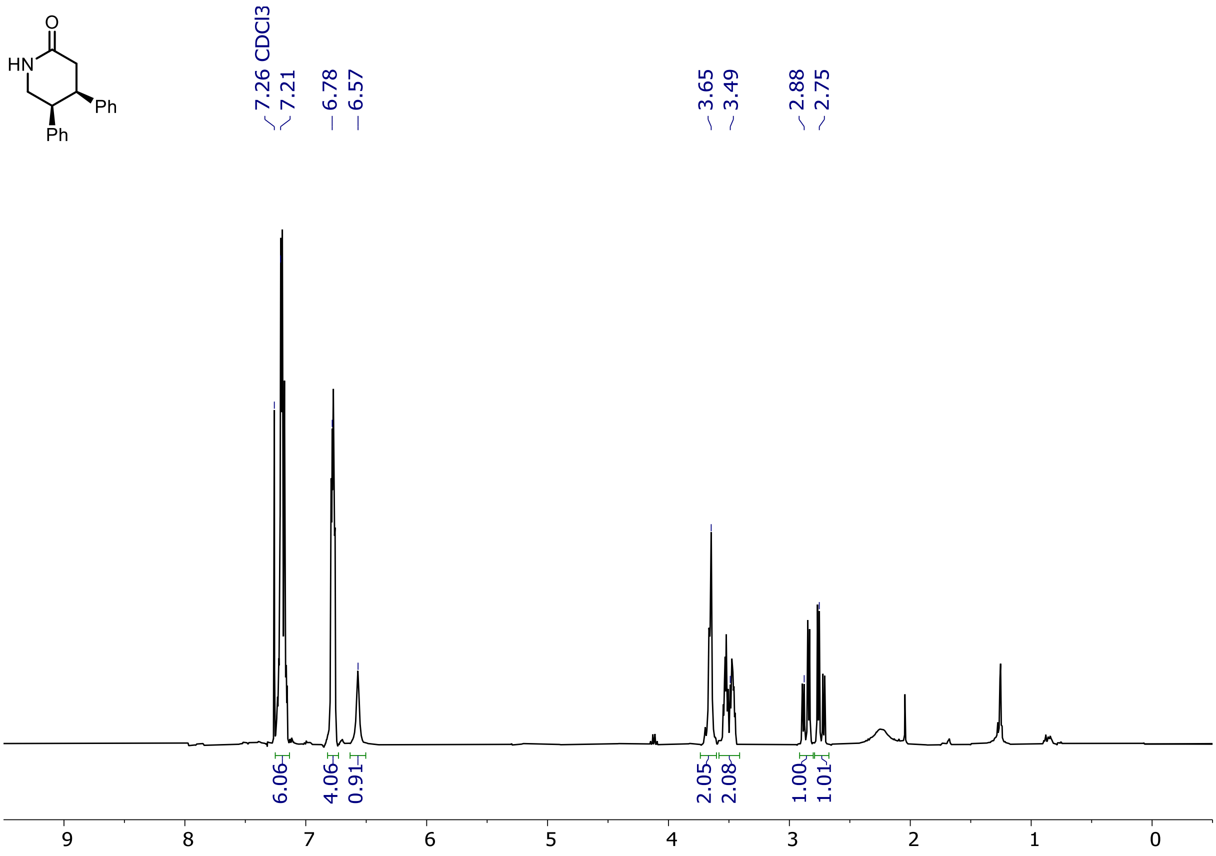

[2n] ^{13}C, $CDCl_3$, 101 MHz

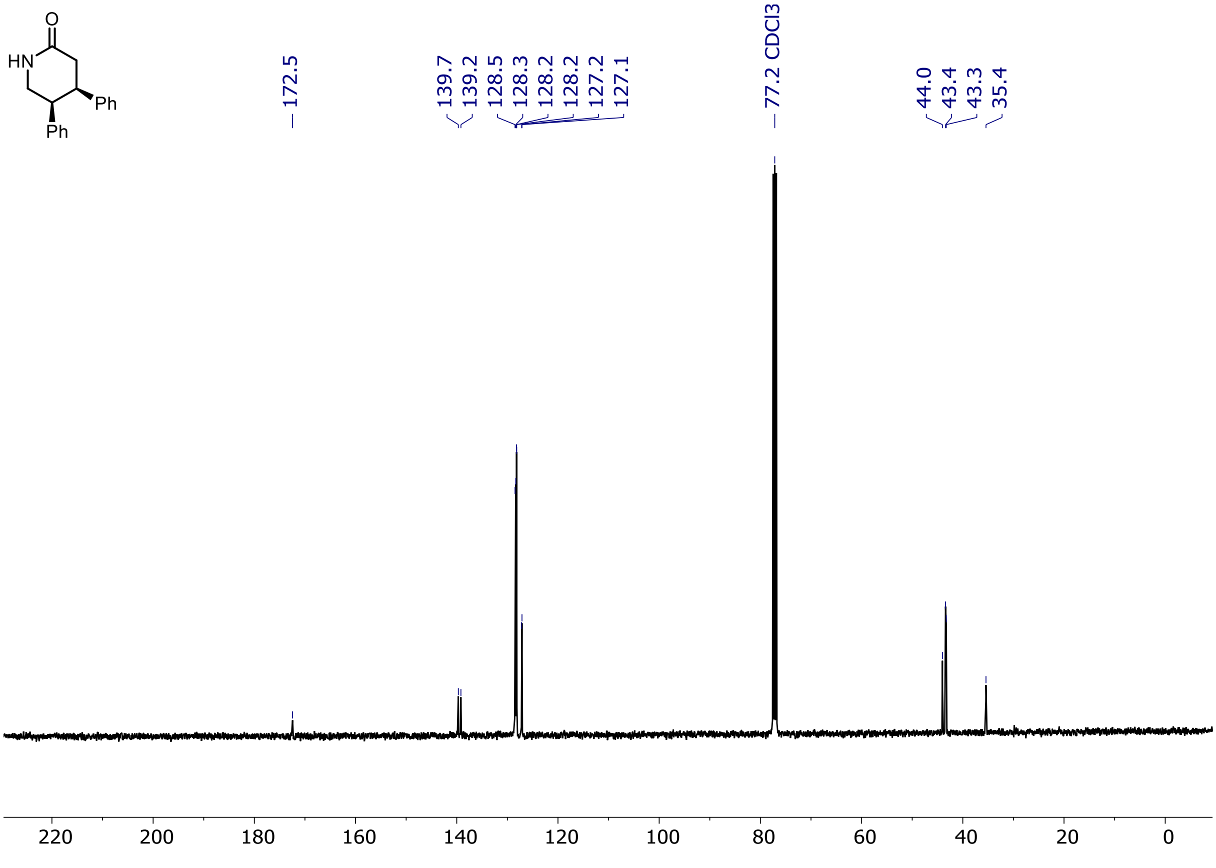

[6o] ^{1}H, $CDCl_3$, 400 MHz

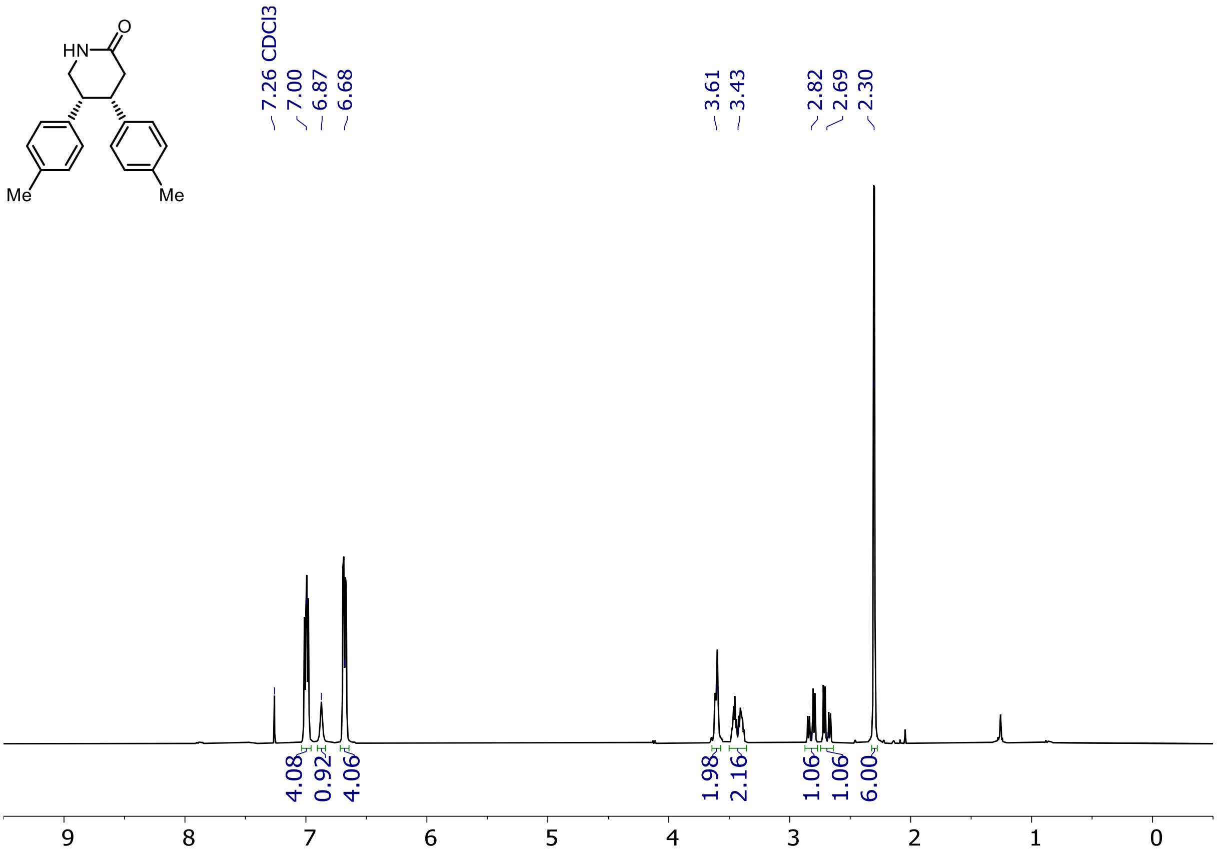

[6o] ^{13}C, $CDCl_3$, 101 MHz

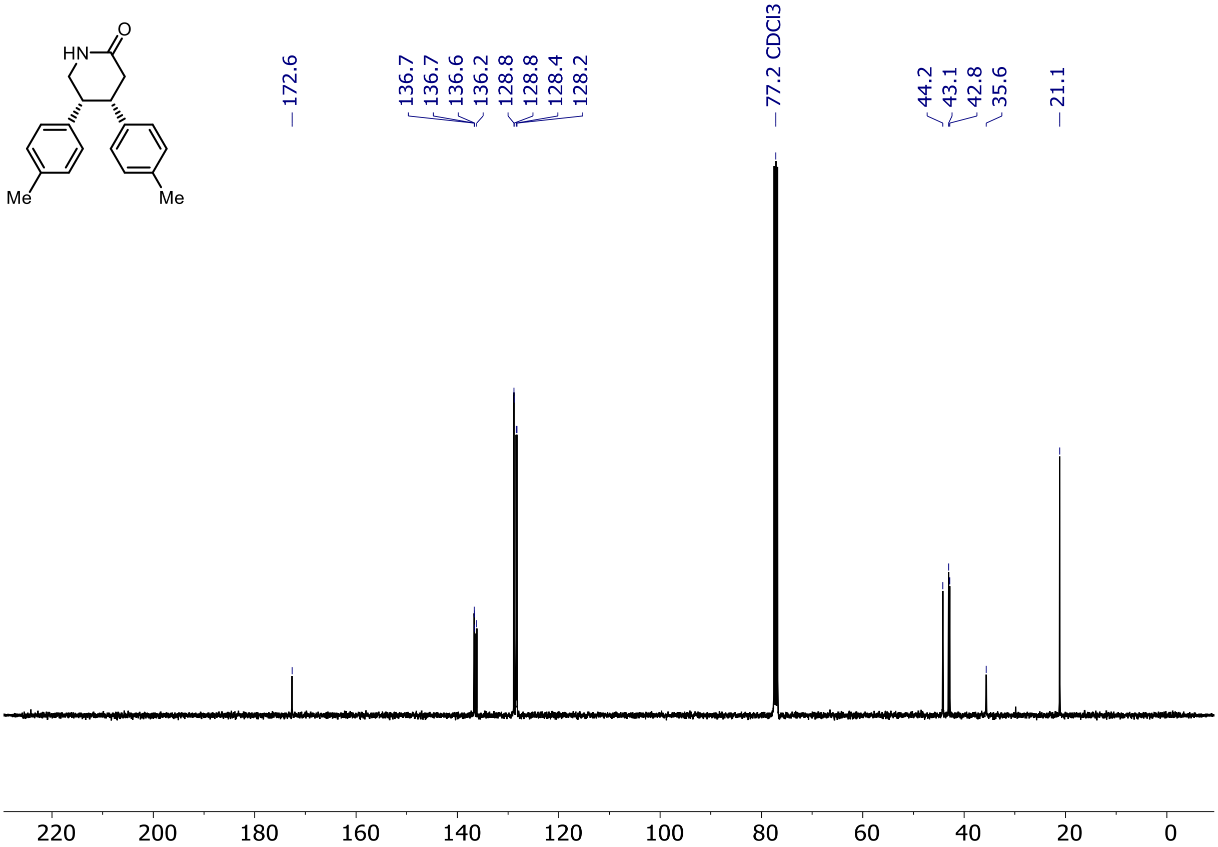

[6p] ^{1}H, $CDCl_3$, 400 MHz

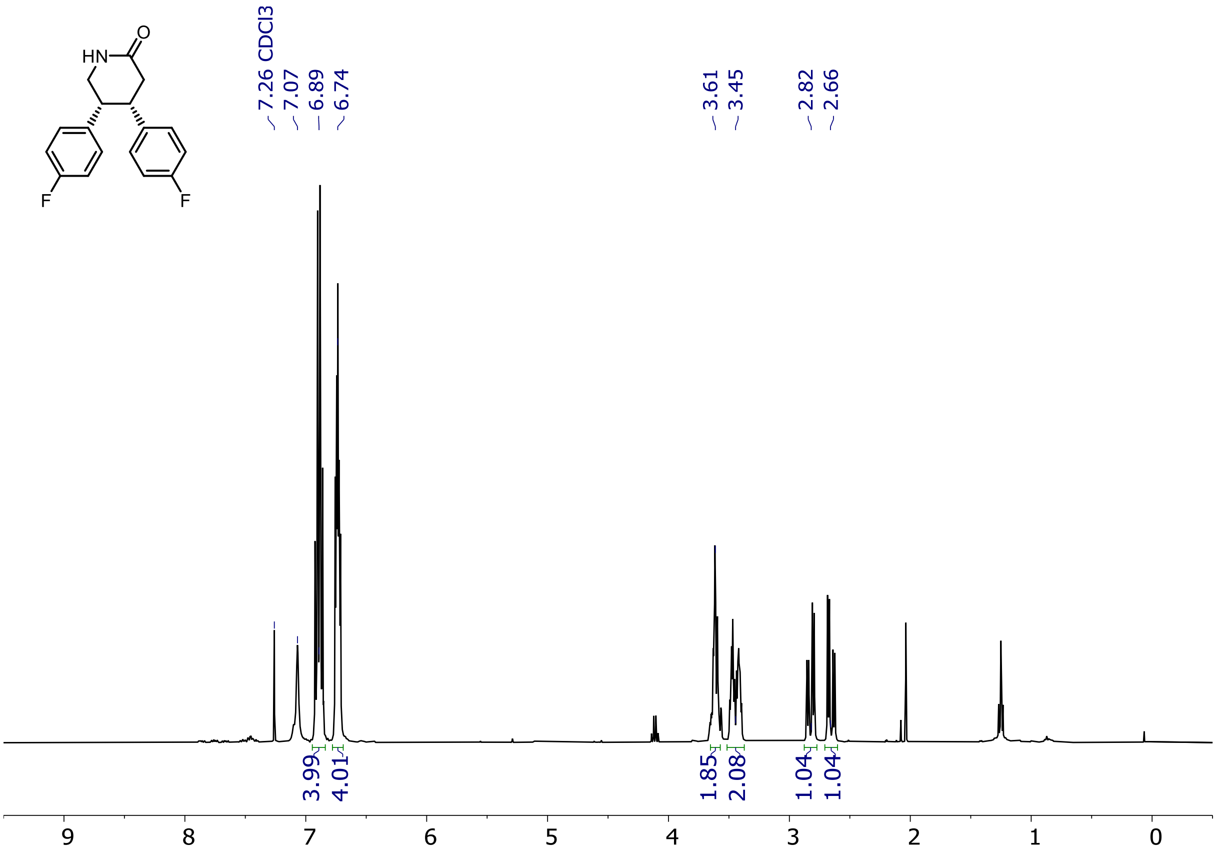

[6p] ^{13}C, $CDCl_3$, 101 MHz

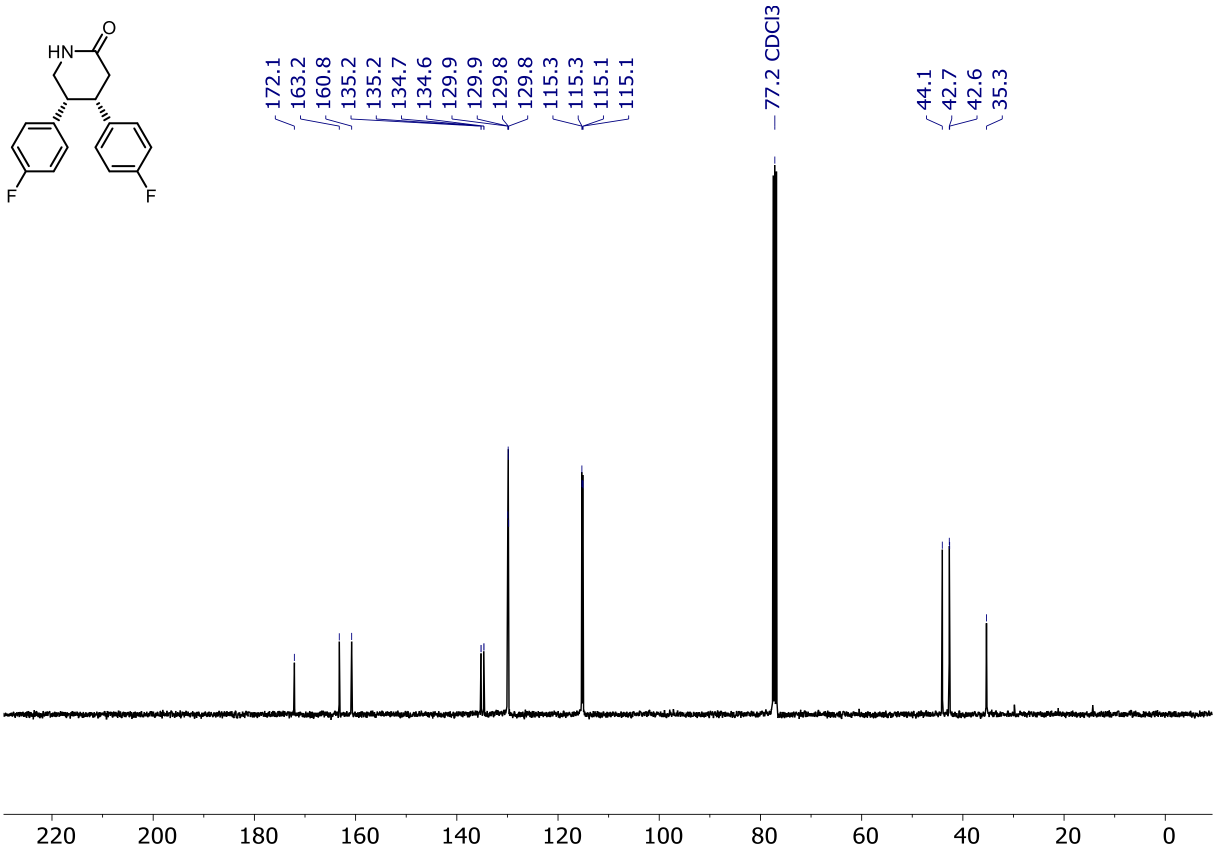

[2p] ^{19}F, $CDCl_3$, 377 MHz

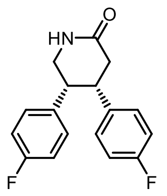

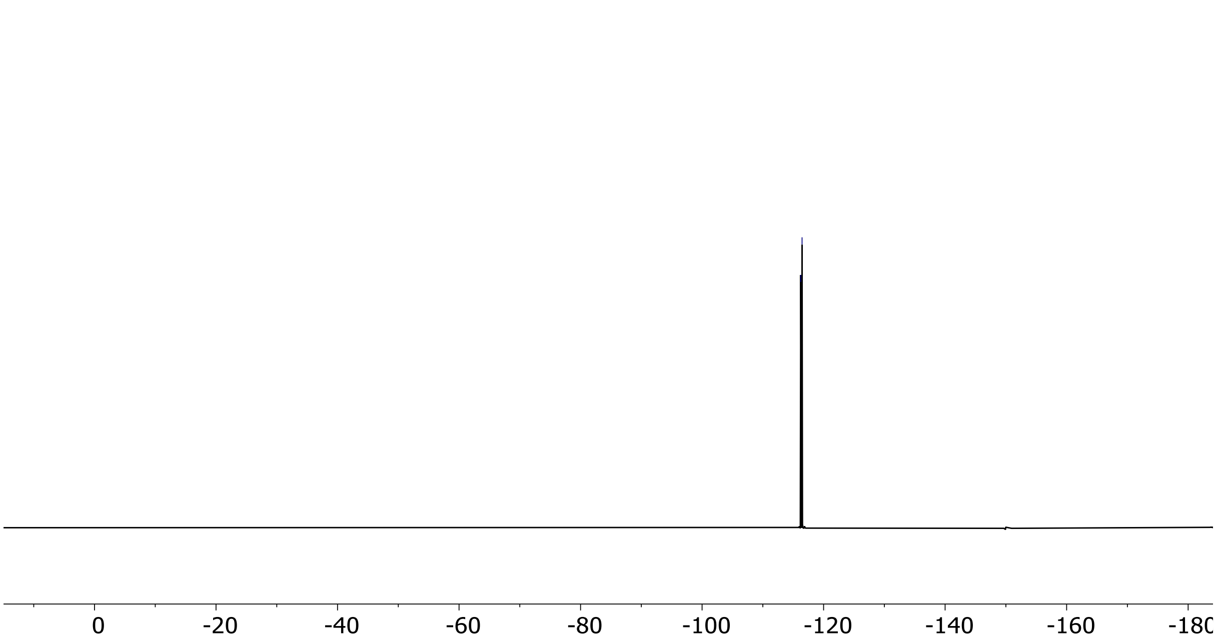

[6q] ^1H, $CDCl_3$, 400 MHz

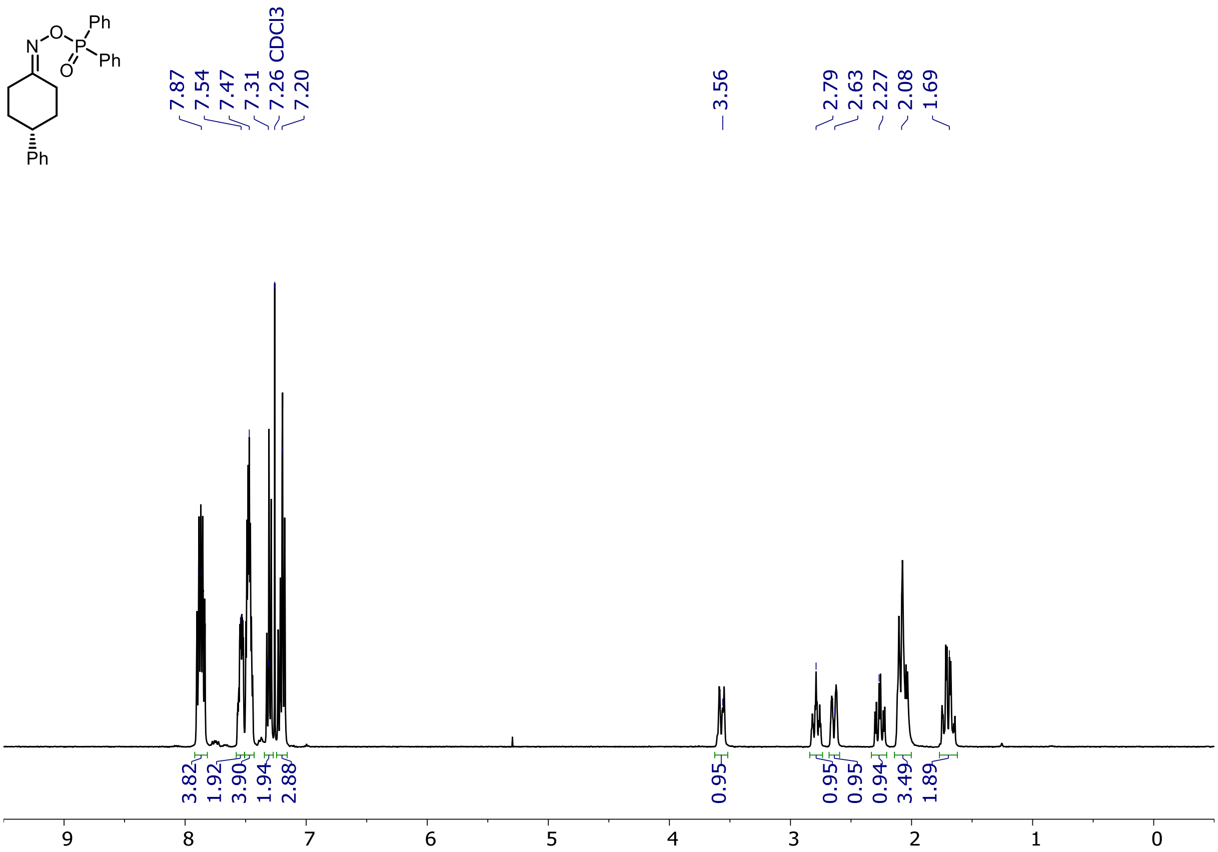

[6q] ^{13}C, $CDCl_3$, 101 MHz

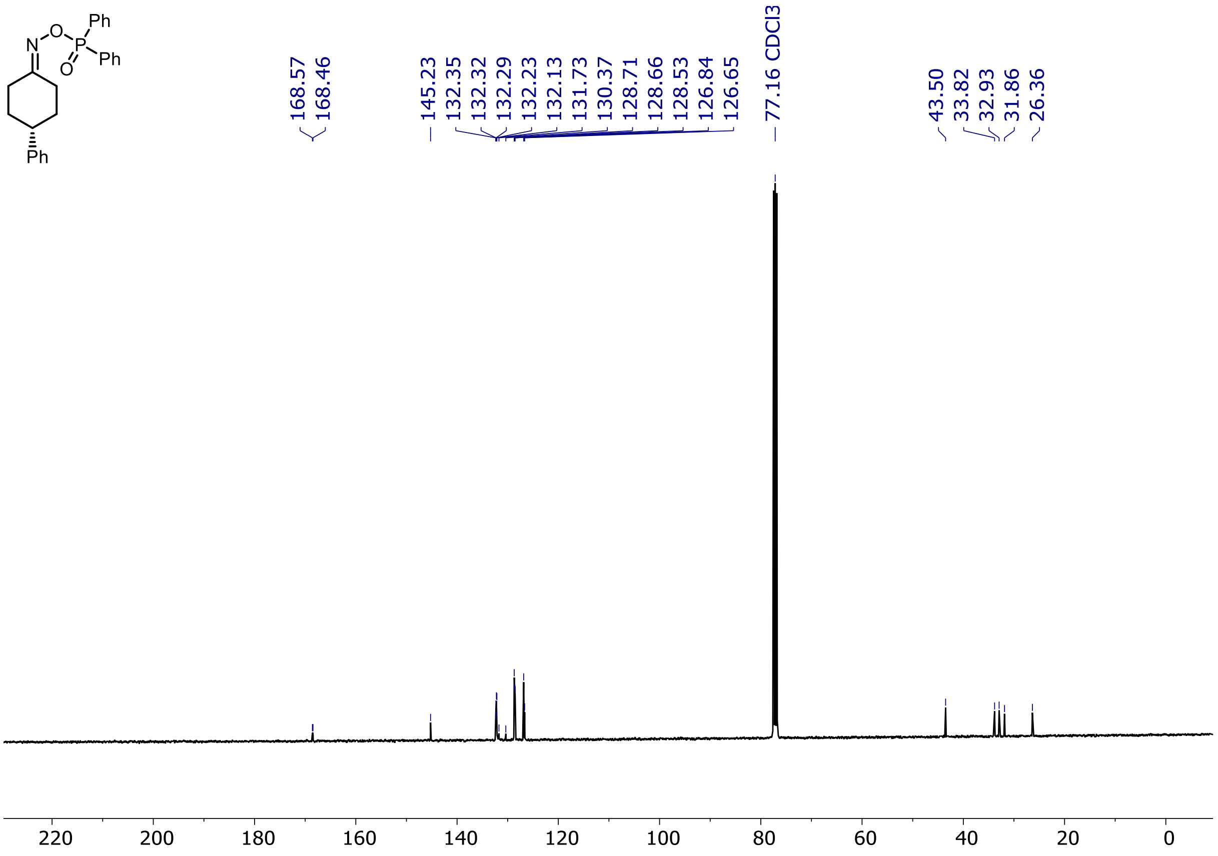

[6q] ^{31}P, $CDCl_3$, 162 MHz

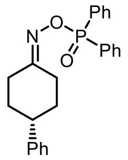

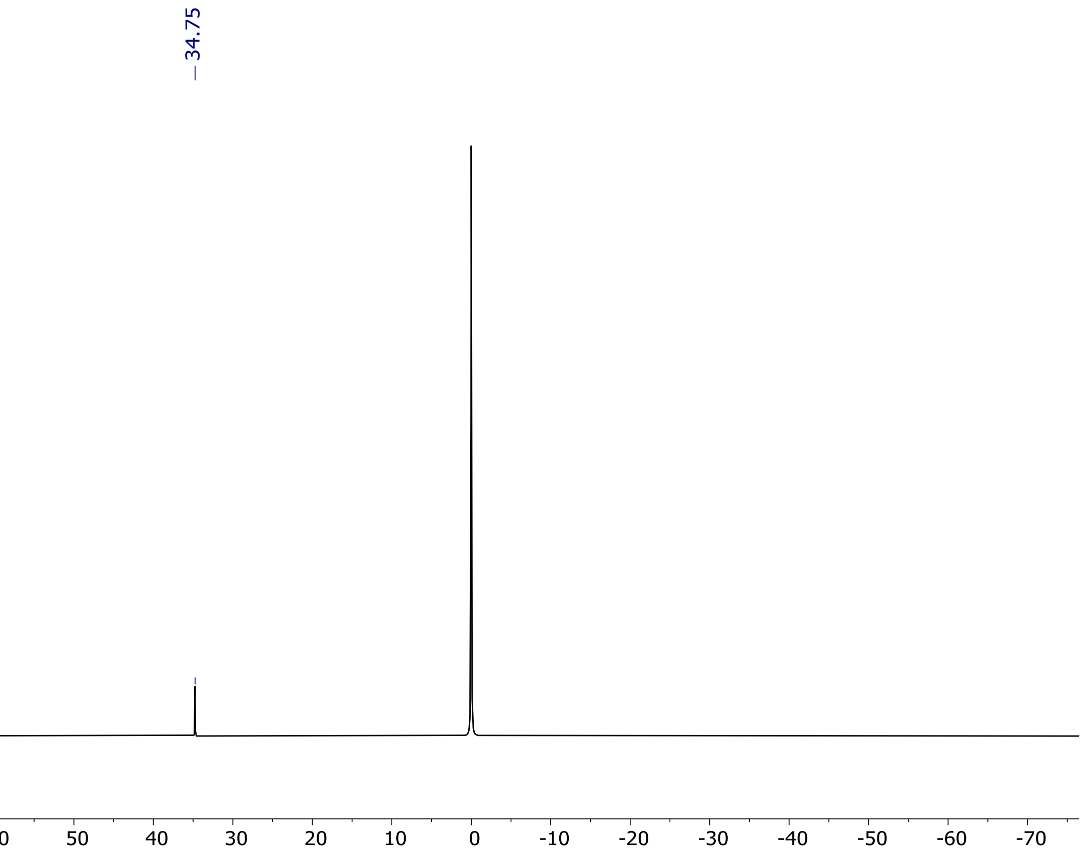

[2q] ^{1}H, $CDCl_3$, 400 MHz

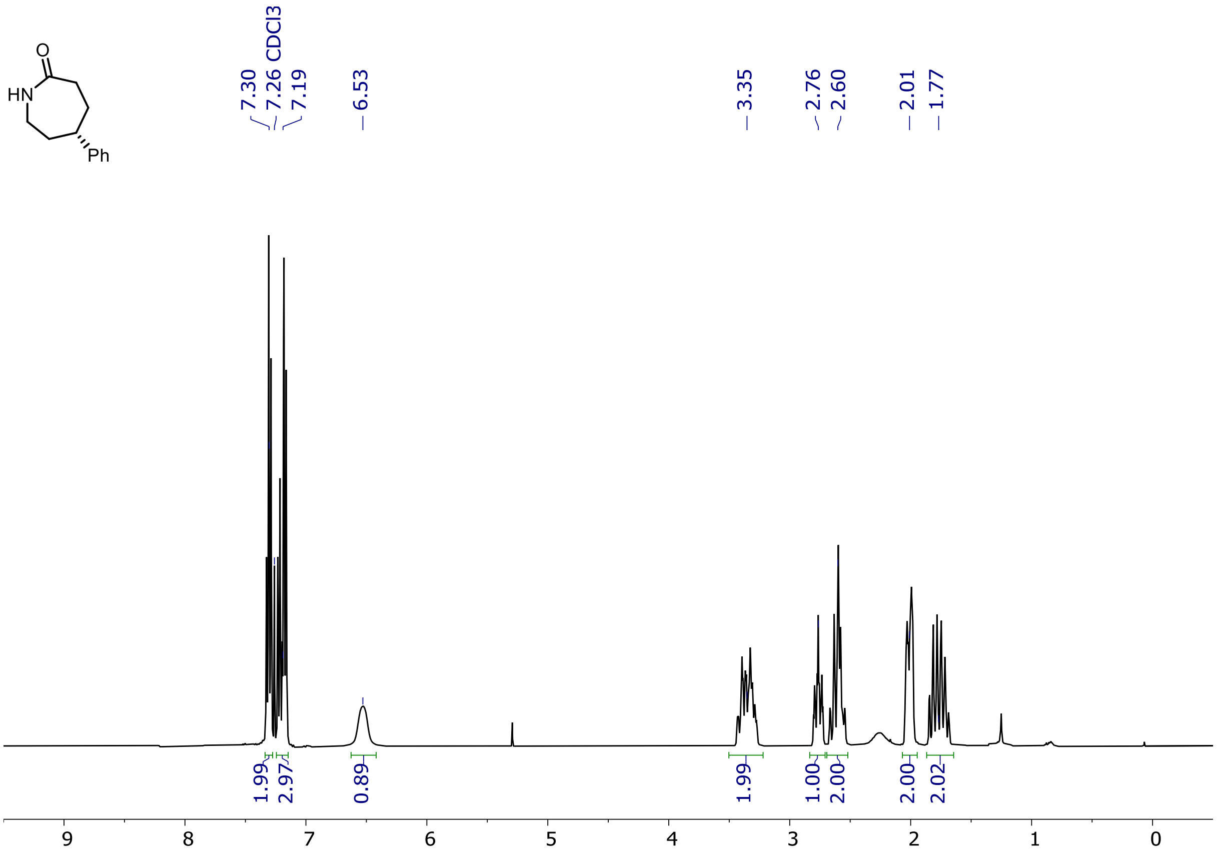

[2q] ^{13}C, $CDCl_3$, 101 MHz

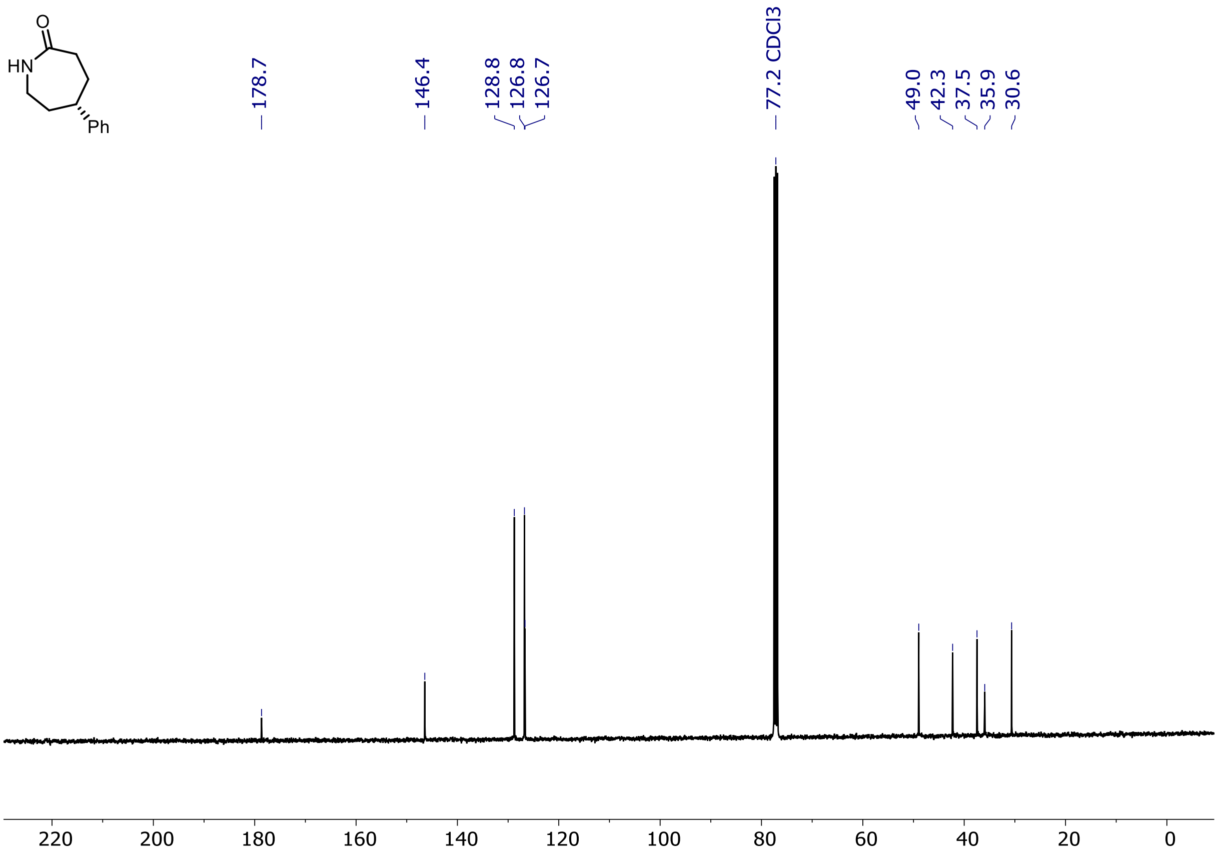

[6r] ^{1}H, $CDCl_3$, 400 MHz

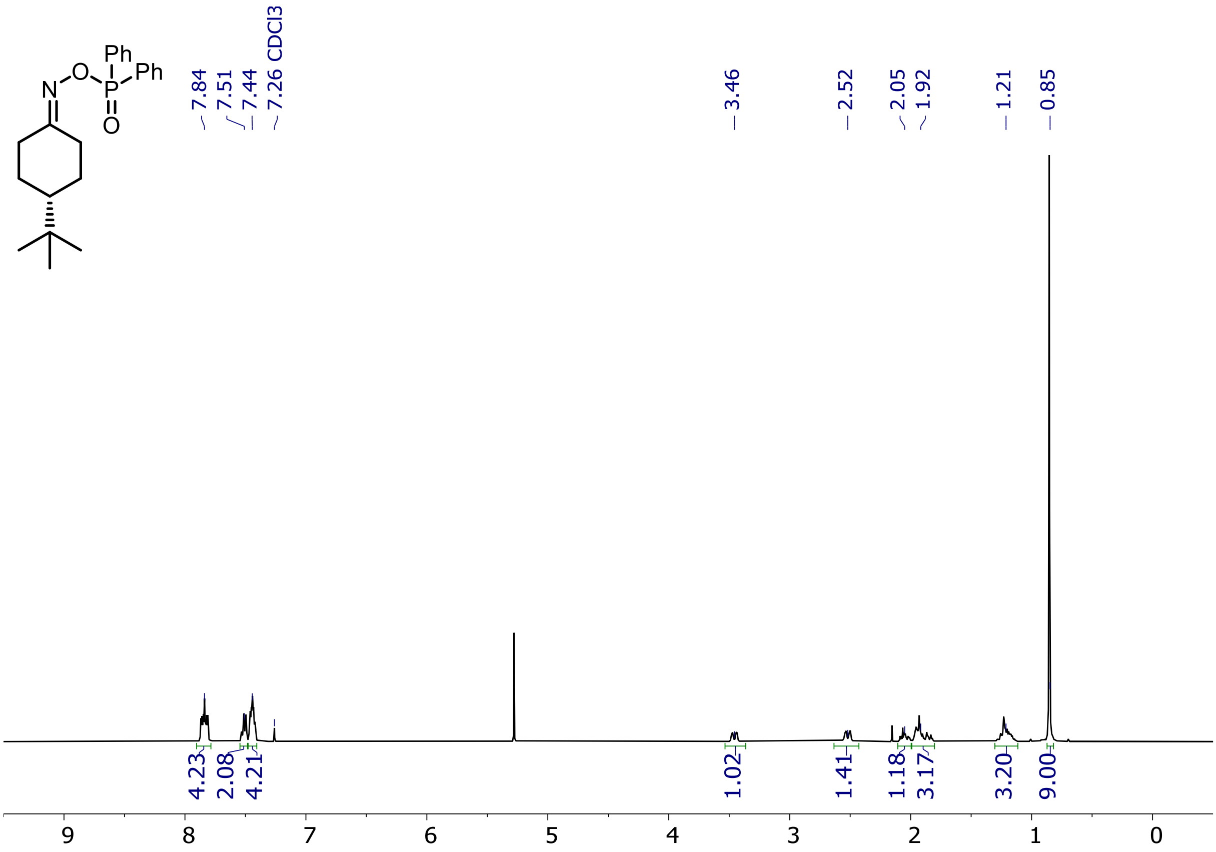

[6r] ^{13}C, $CDCl_3$, 101 MHz

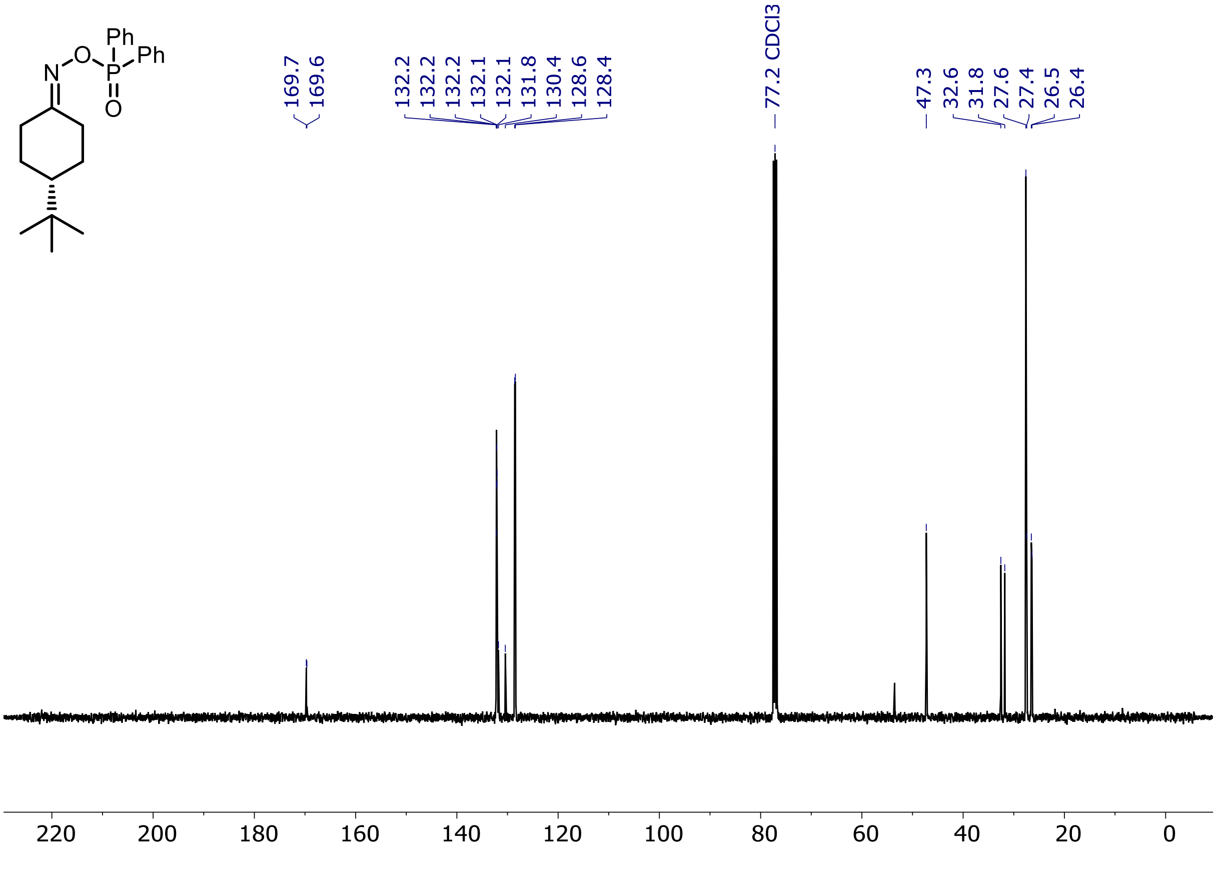

[6r] ^{31}P, $CDCl_3$, 162 MHz

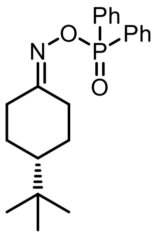

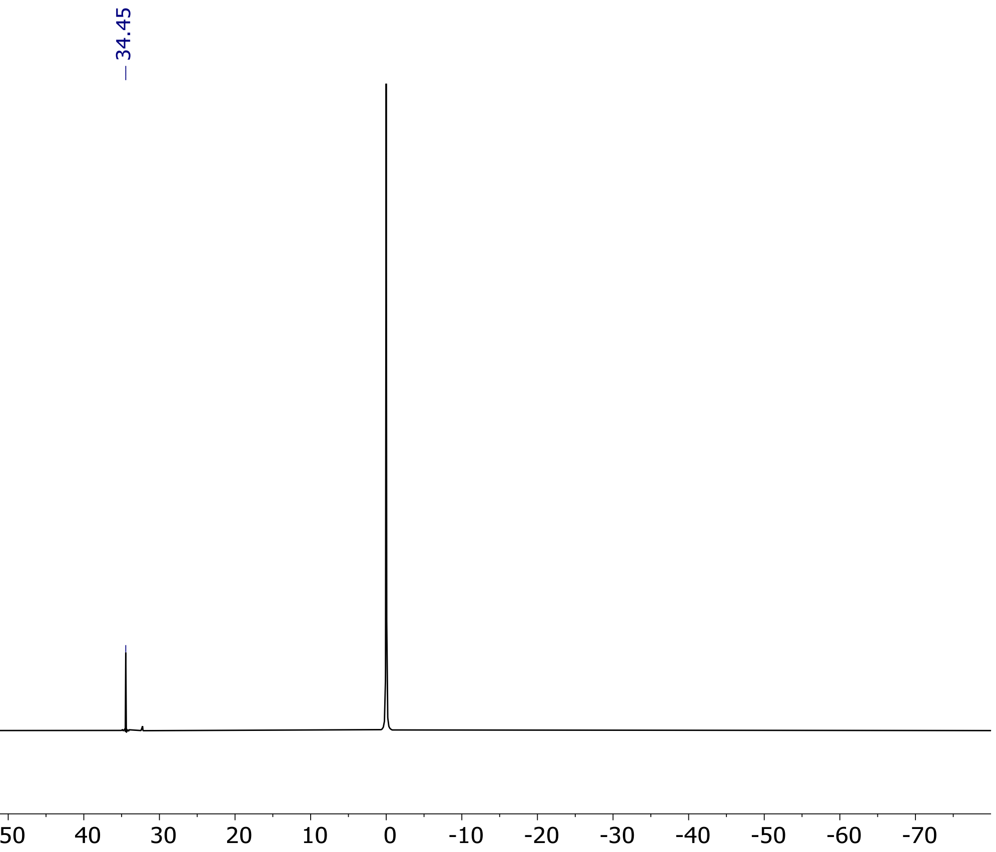

[2r] ^{1}H, $CDCl_3$, 400 MHz

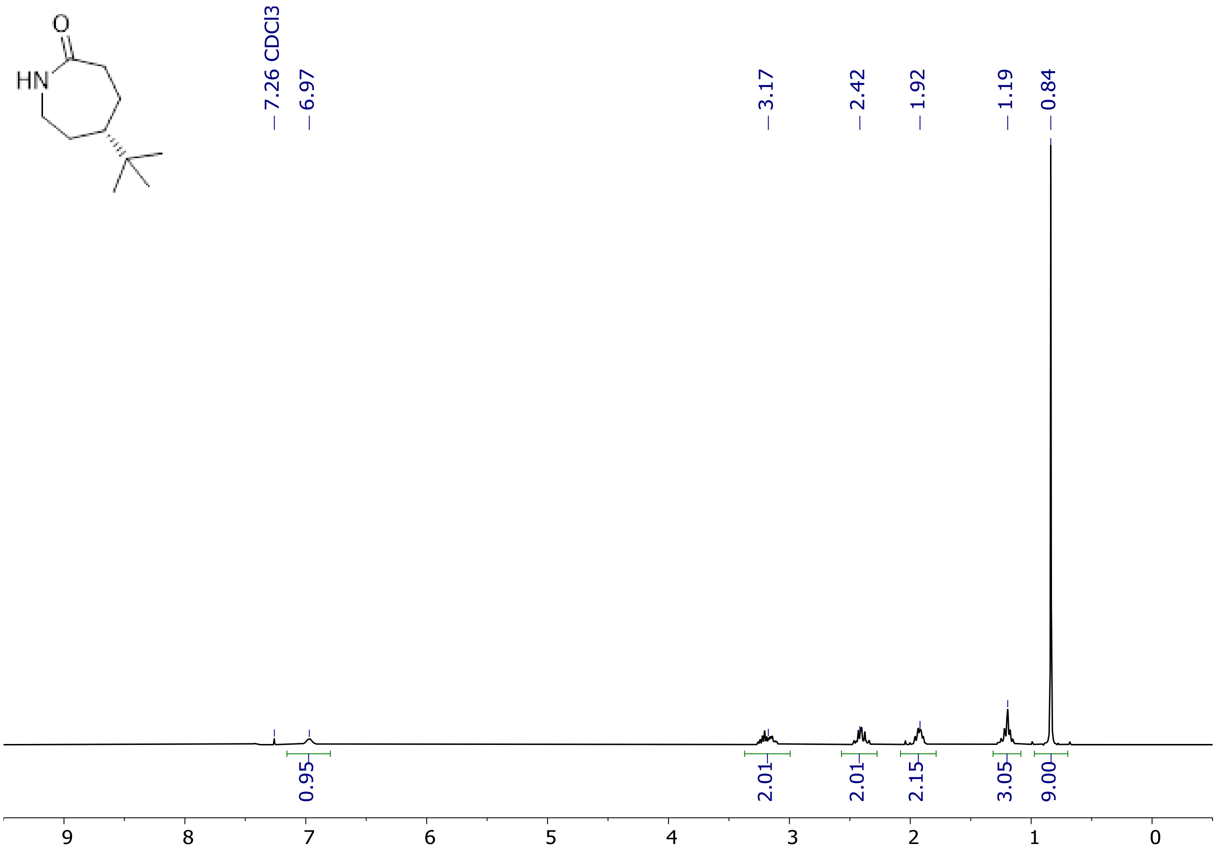

[2r] ^{13}C, $CDCl_3$, 101 MHz

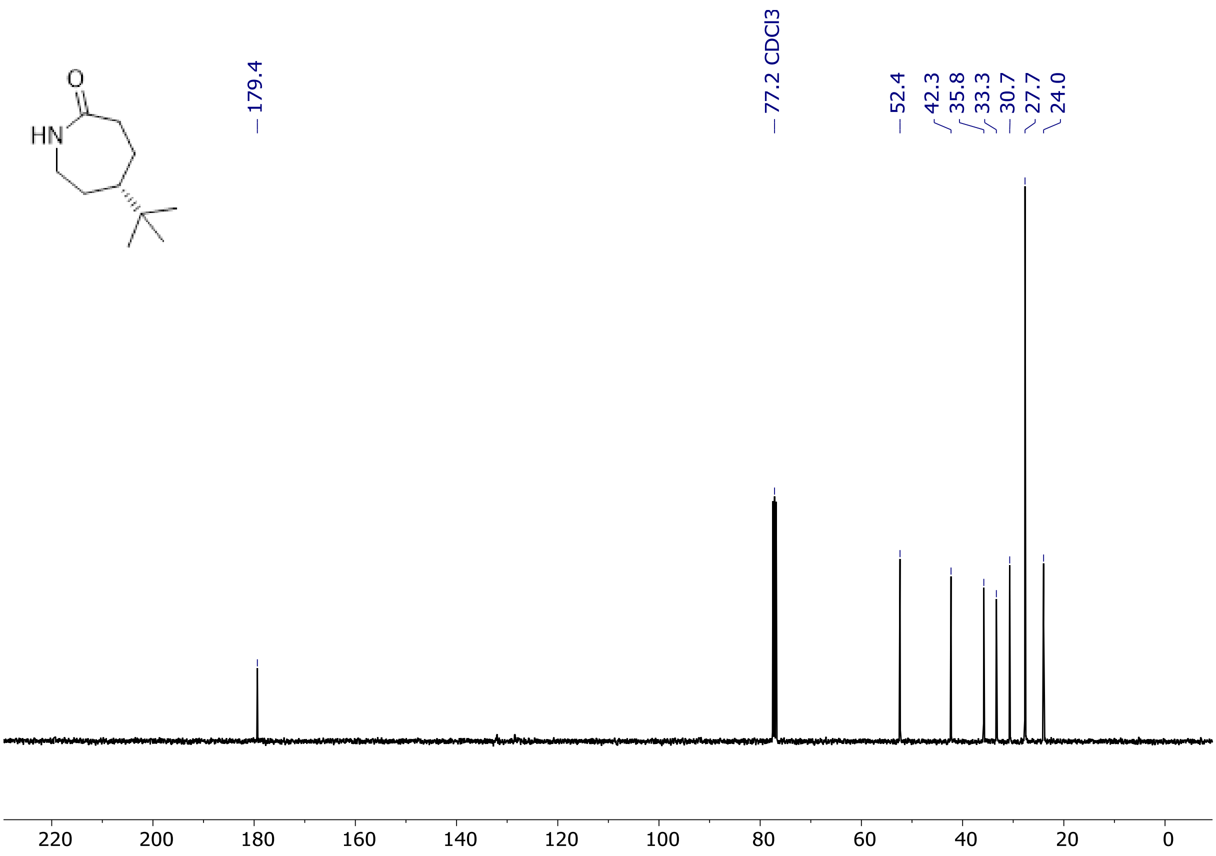

[10] ^{1}H, $CDCl_3$, 400 MHz

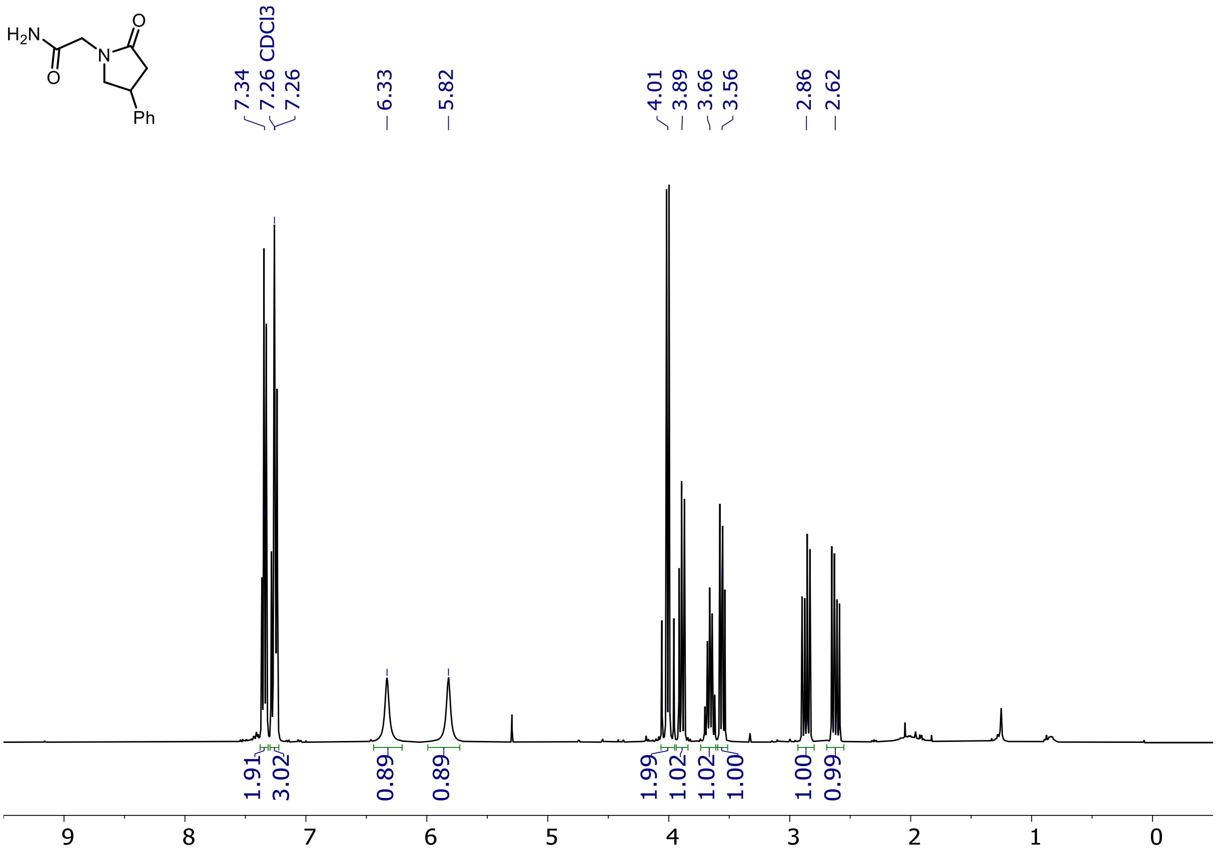

[10] ^{13}C, $CDCl_3$, 101 MHz

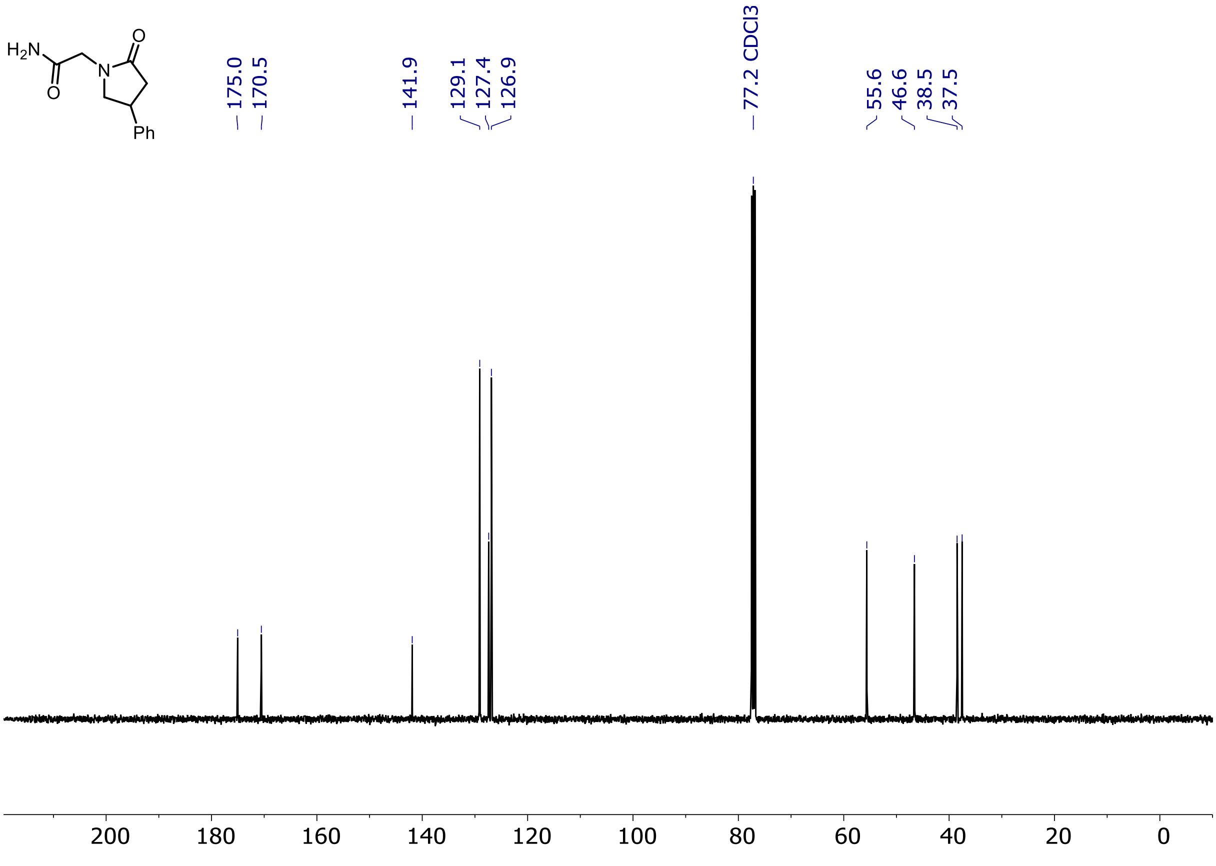

[12] ^{1}H, $CDCl_3$, 400 MHz

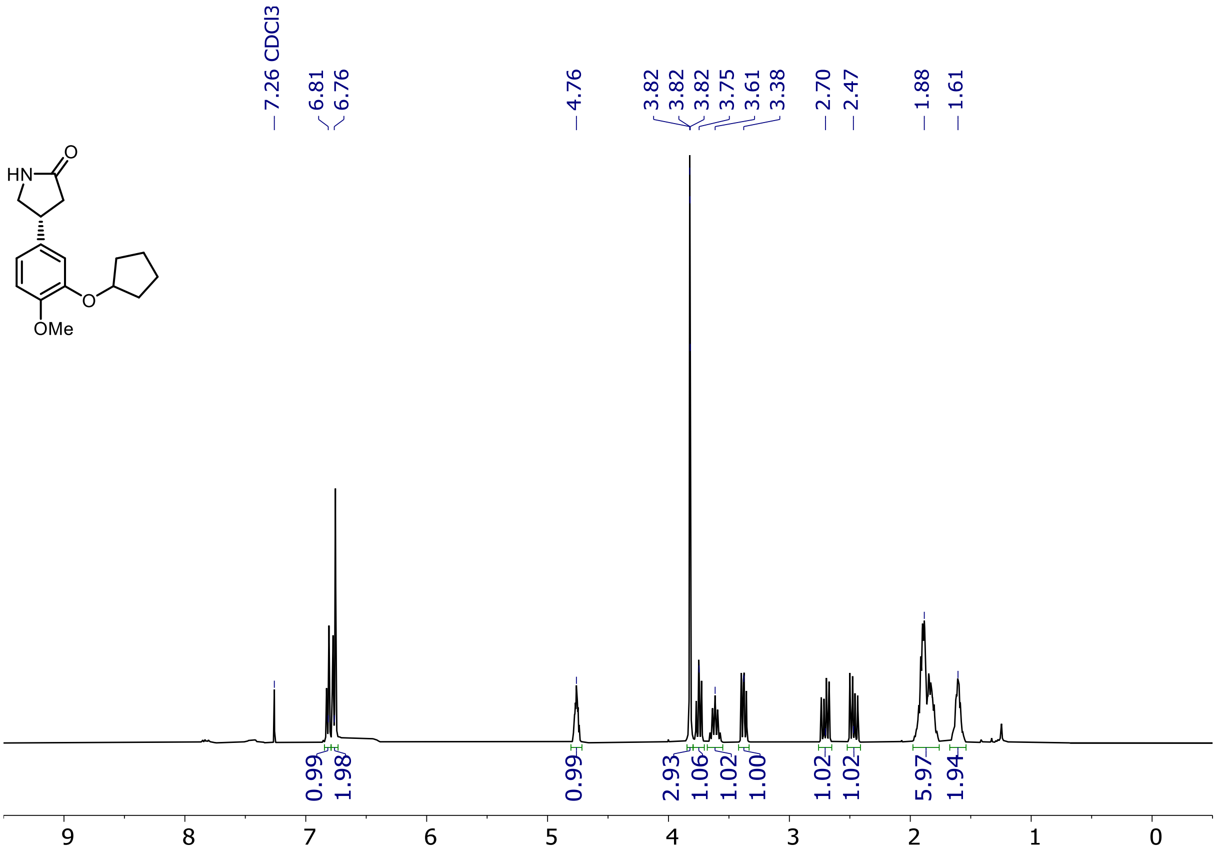

[12] ^{13}C, $CDCl_3$, 101 MHz

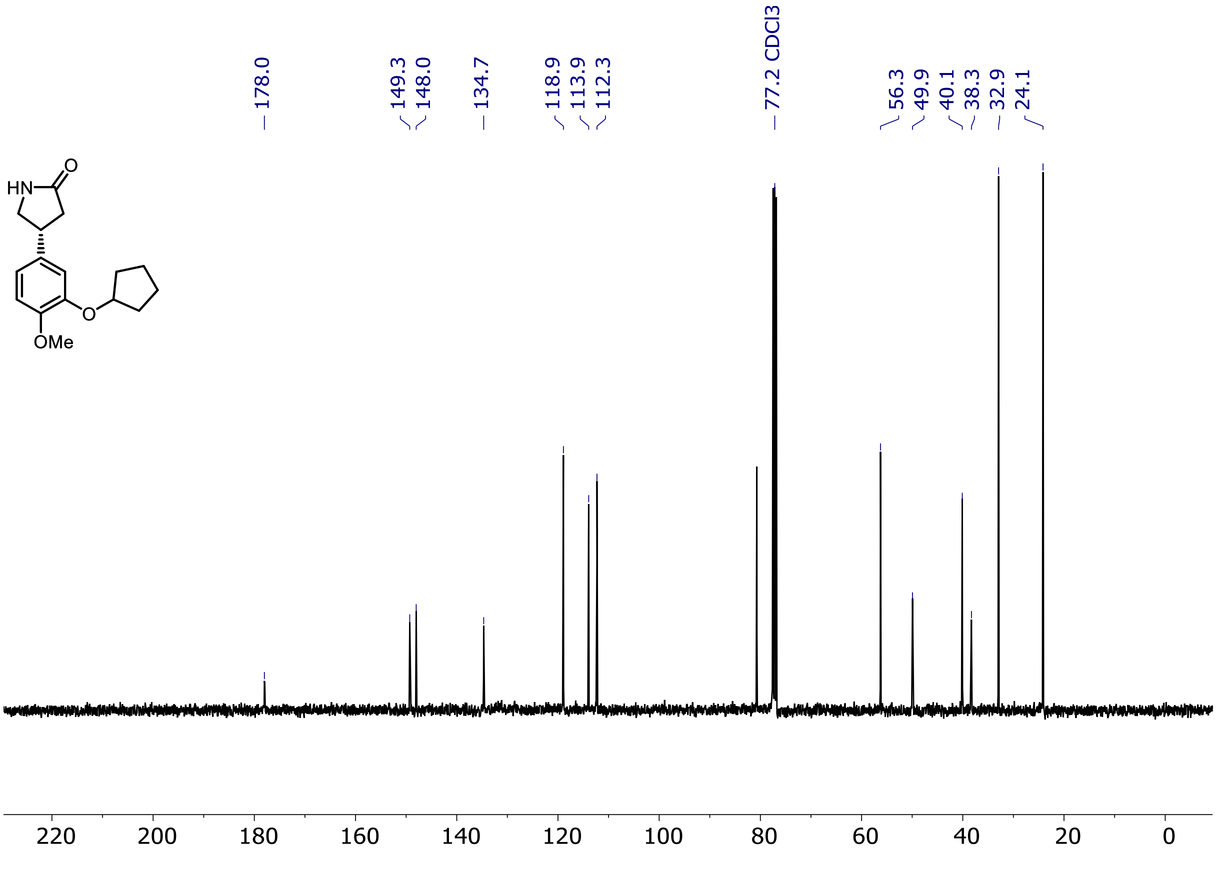

[13] ^1H, DMSO-d6, 400 MHz

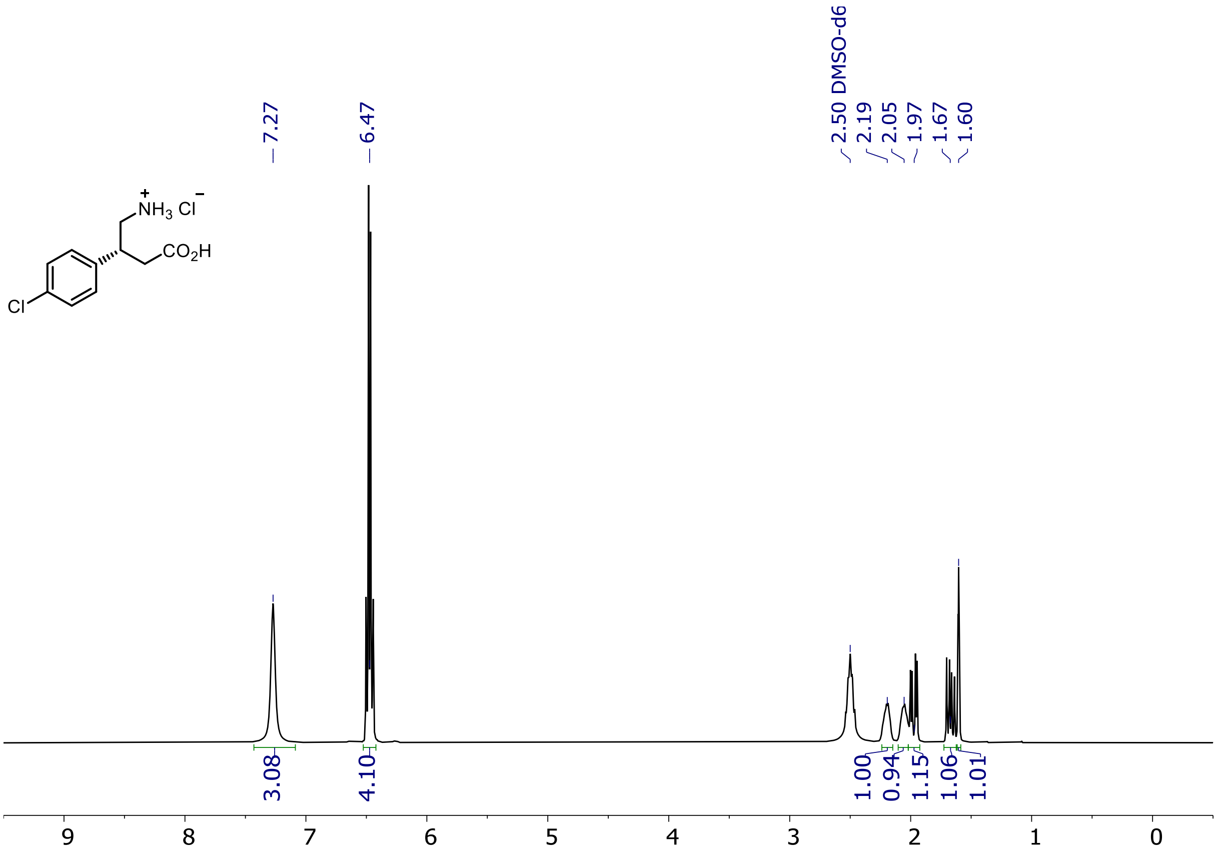

[13] ^{13}C, DMSO-d6, 101 MHz

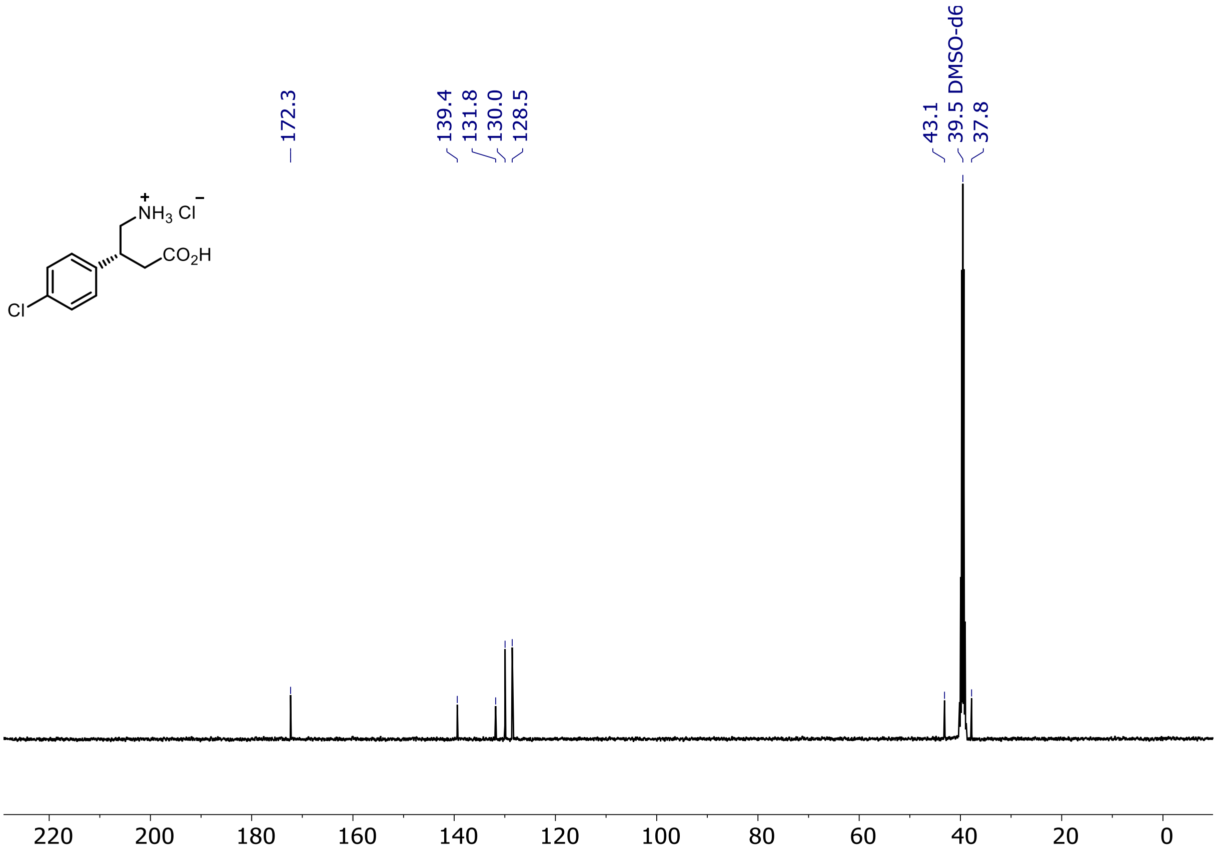

[14] ^{1}H, CD_3OD, 400 MHz

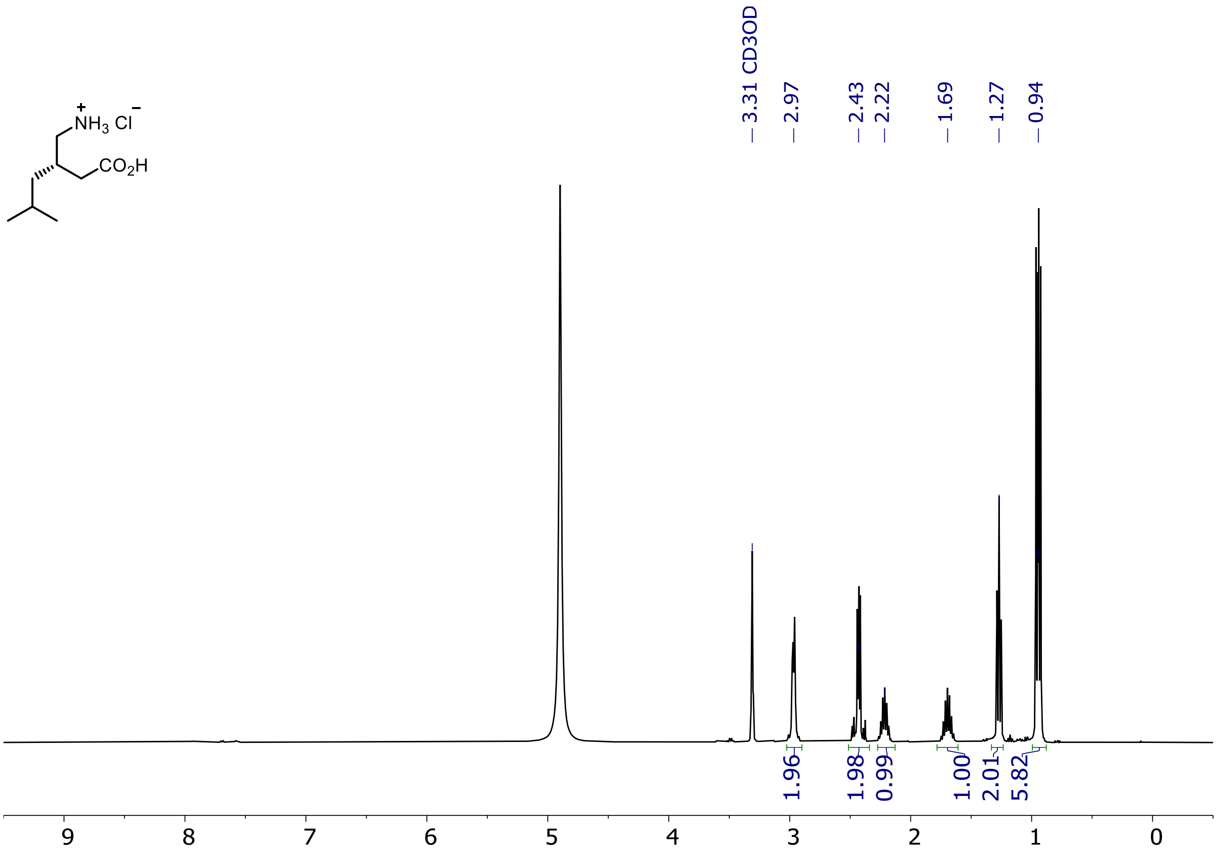

[14] ^{13}C, CD_3OD, 101 MHz

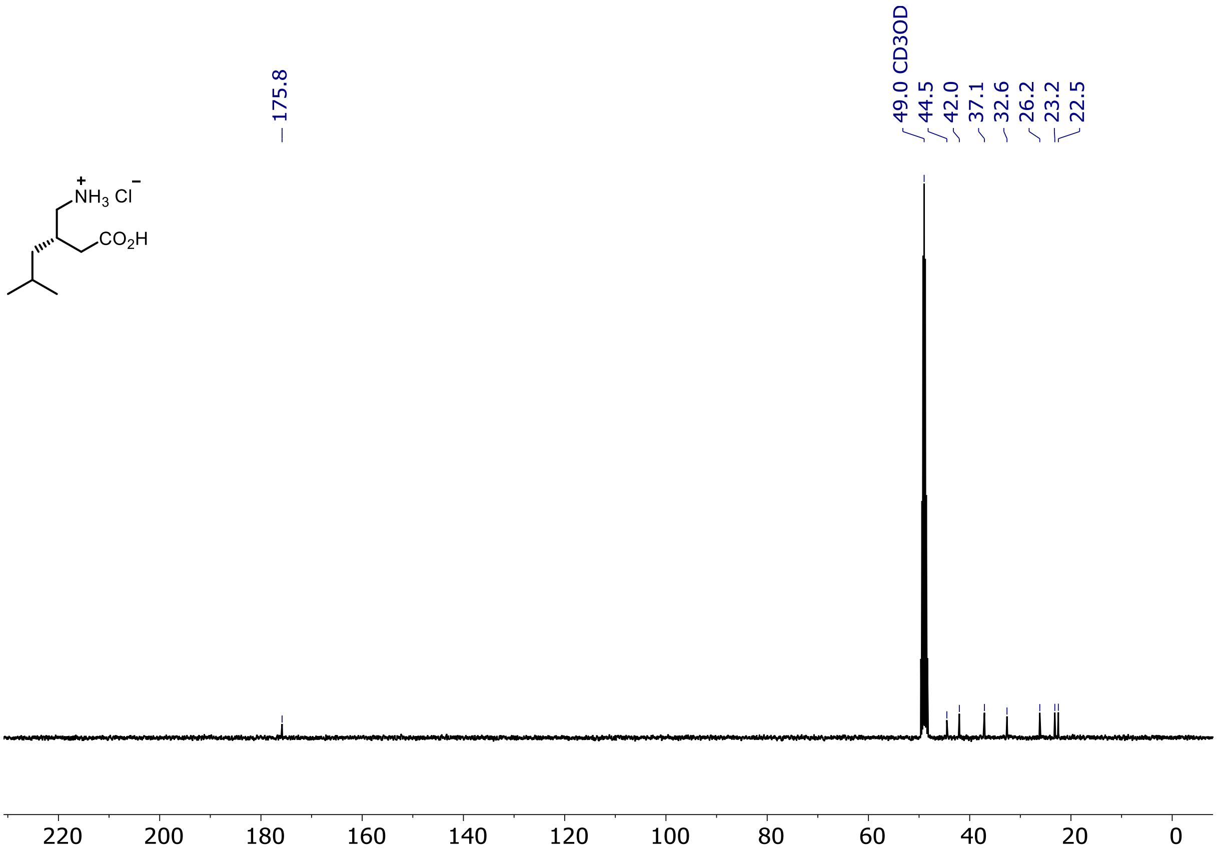

HPLC data

[2a]

top: racemic sample

bottom: enantioenriched sample

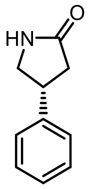

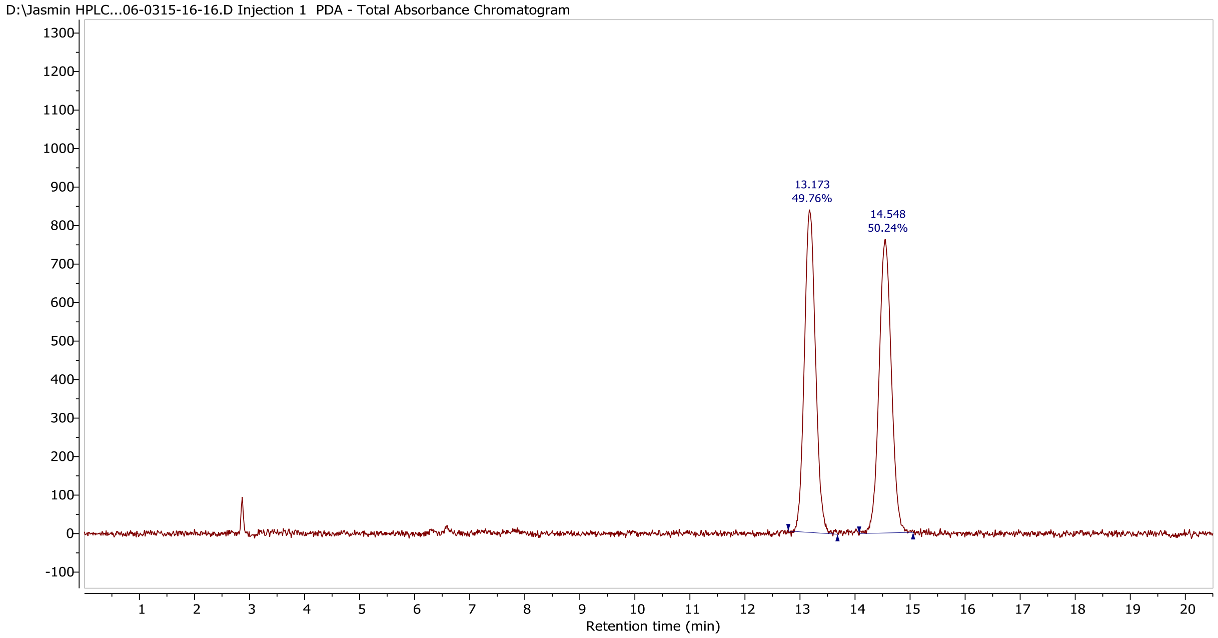

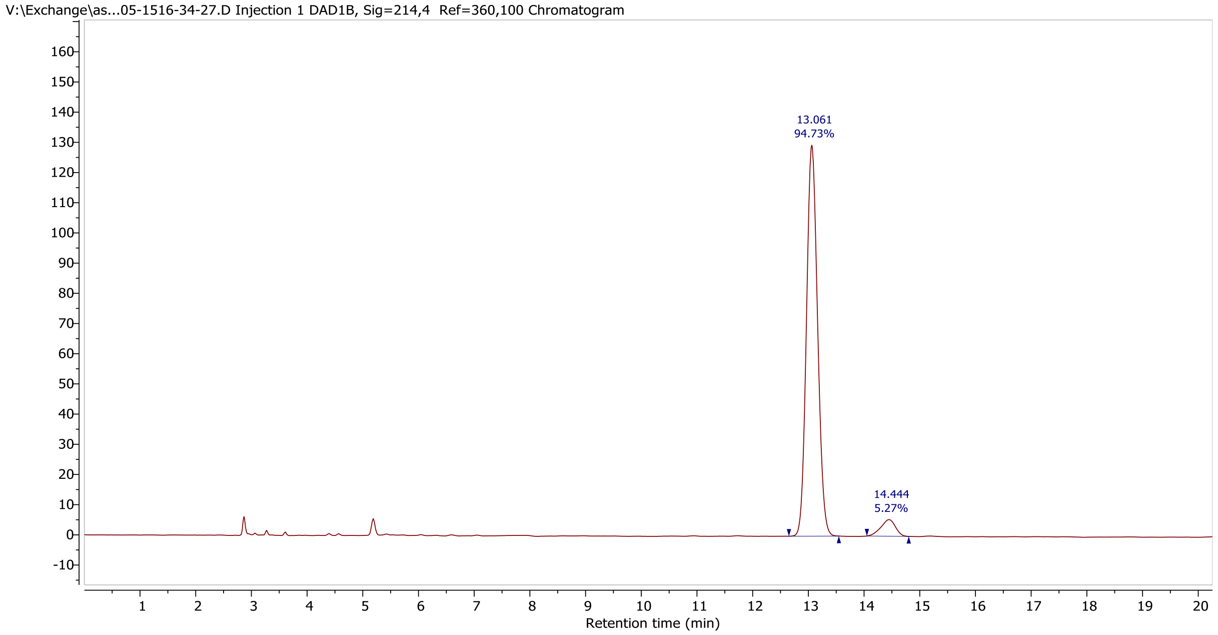

[2b]

top: racemic sample

bottom: enantioenriched sample

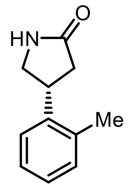

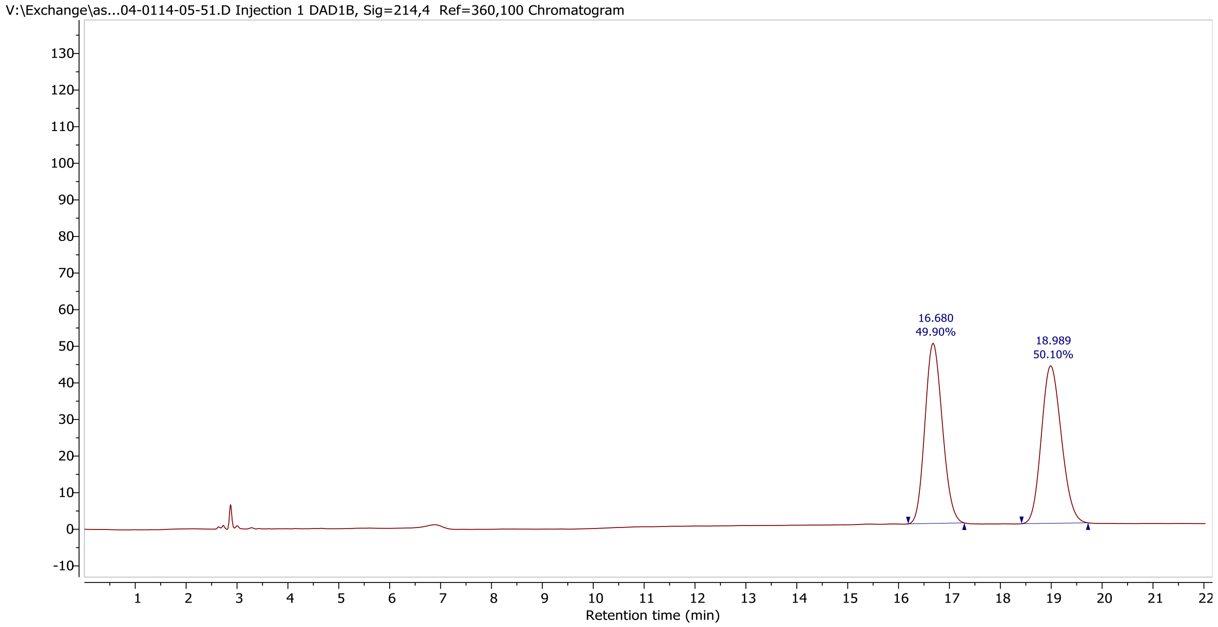

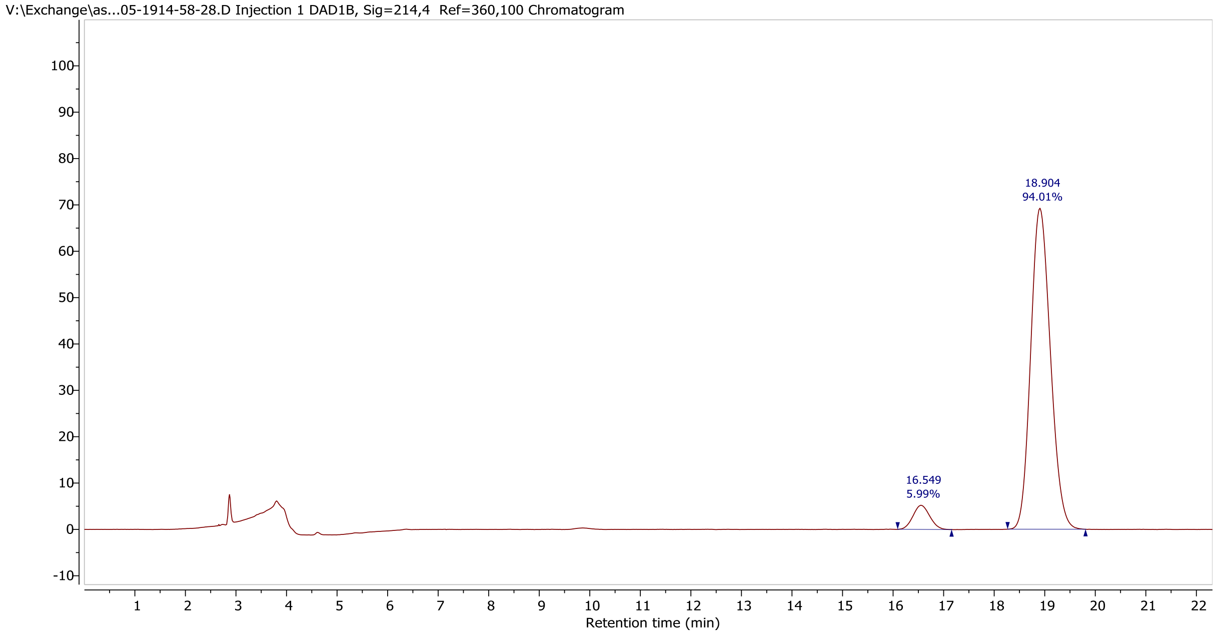

[2c]

top: racemic sample

bottom: enantioenriched sample

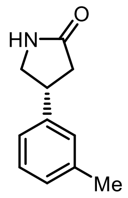

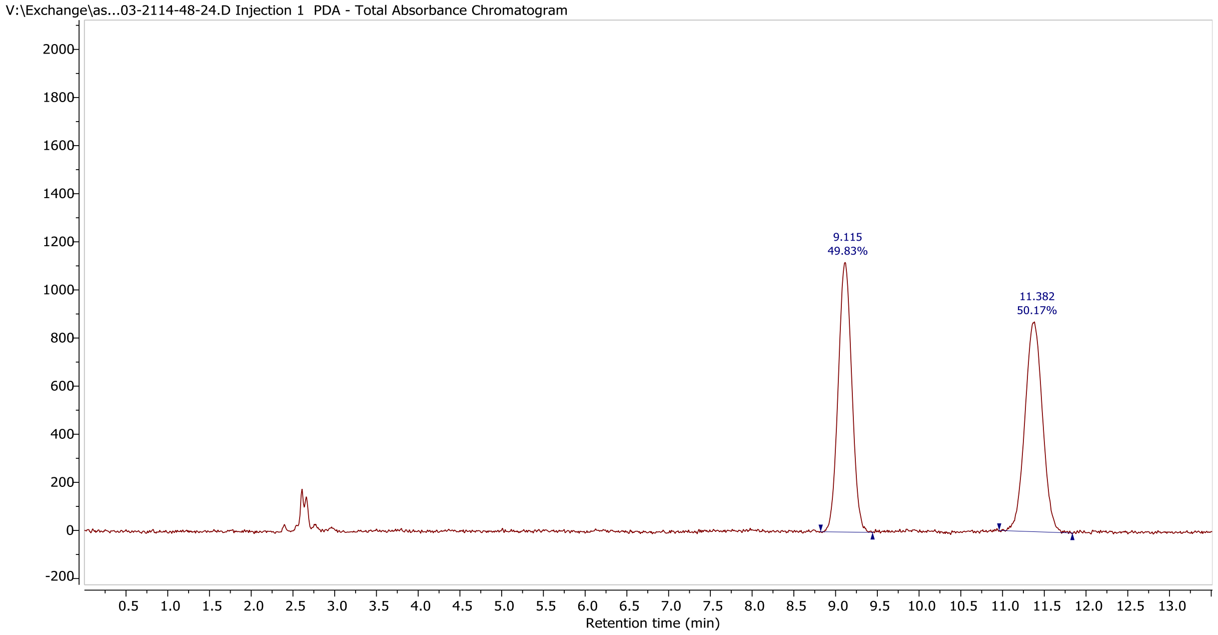

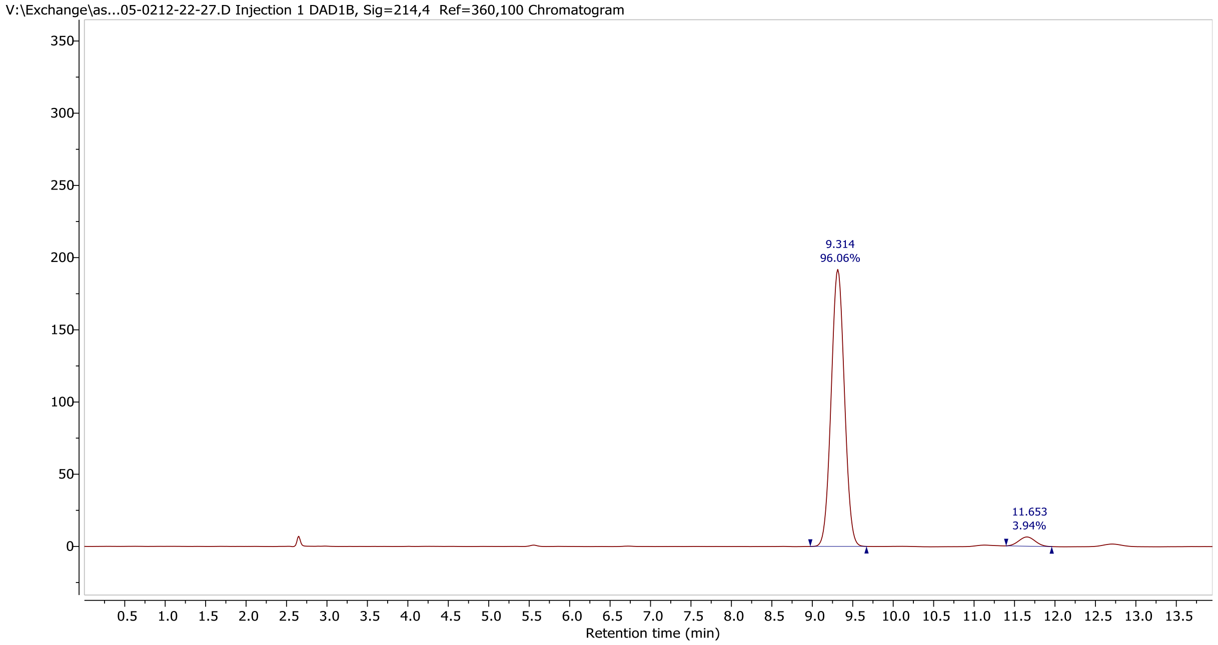

[2d]

top: racemic sample

bottom: enantioenriched sample

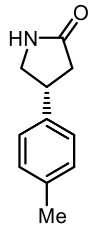

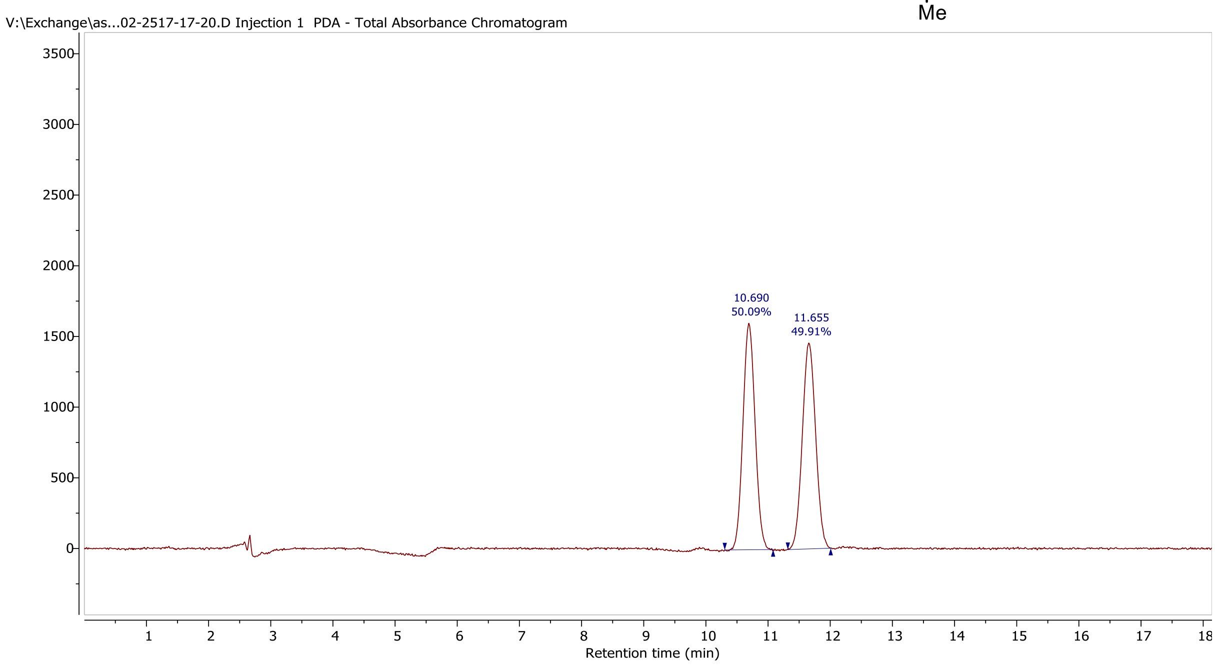

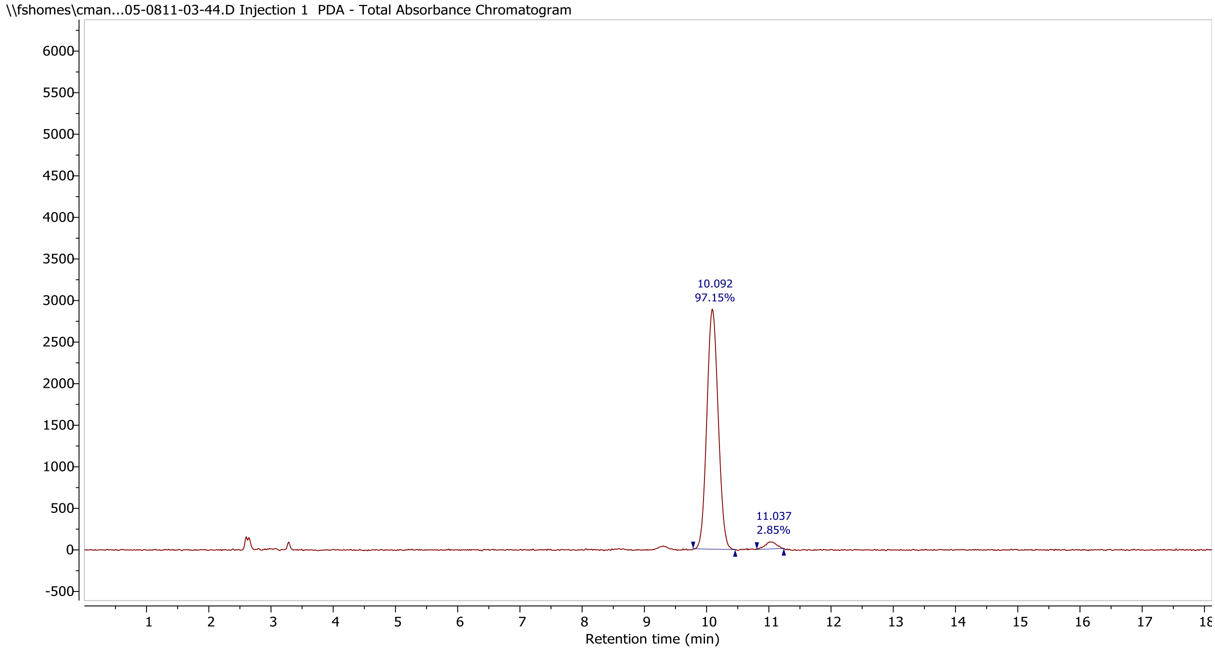

[2e]

top: racemic sample

bottom: enantioenriched sample

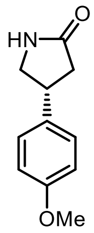

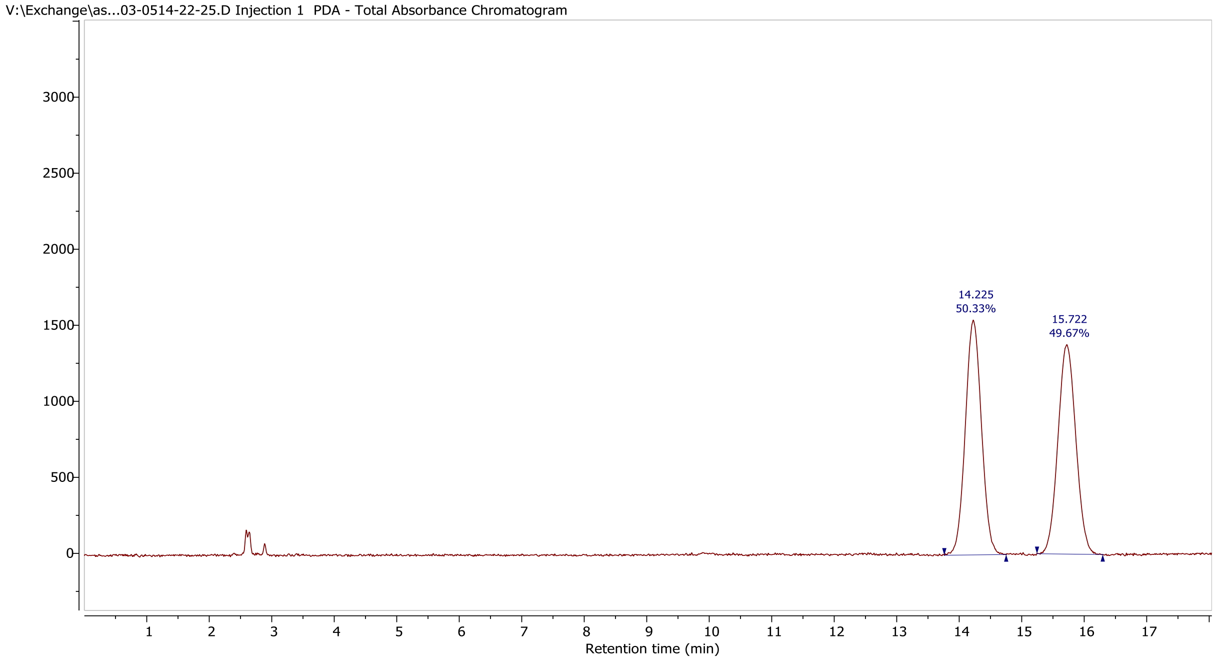

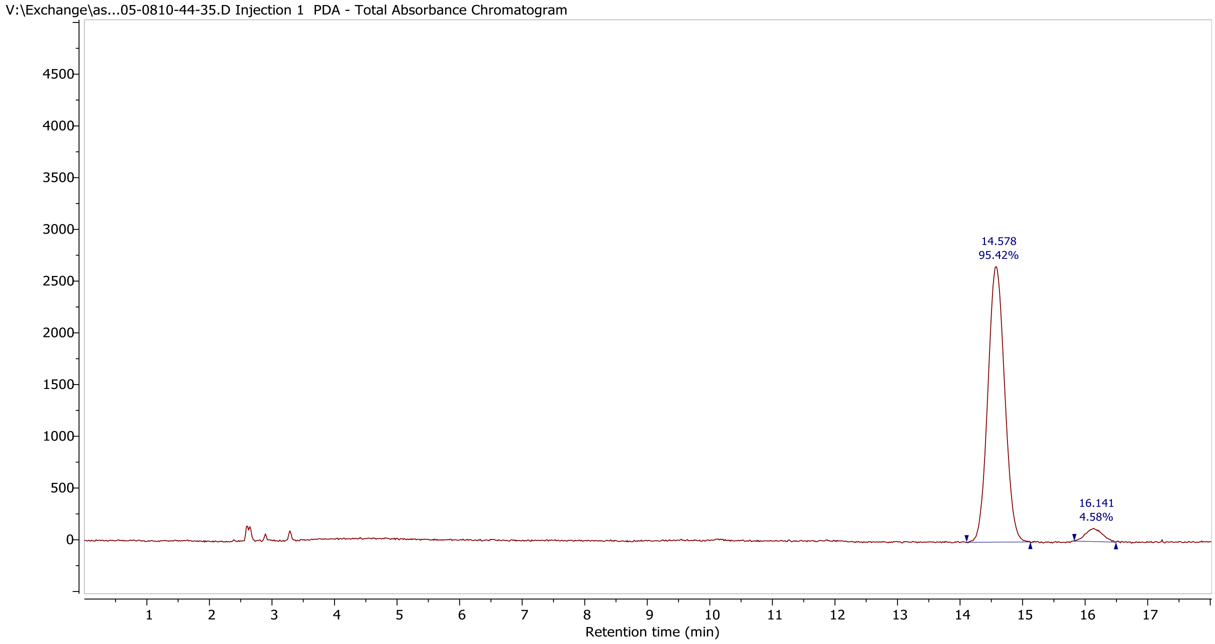

[2f]

top: racemic sample

bottom: enantioenriched sample

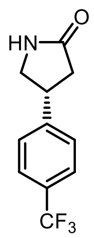

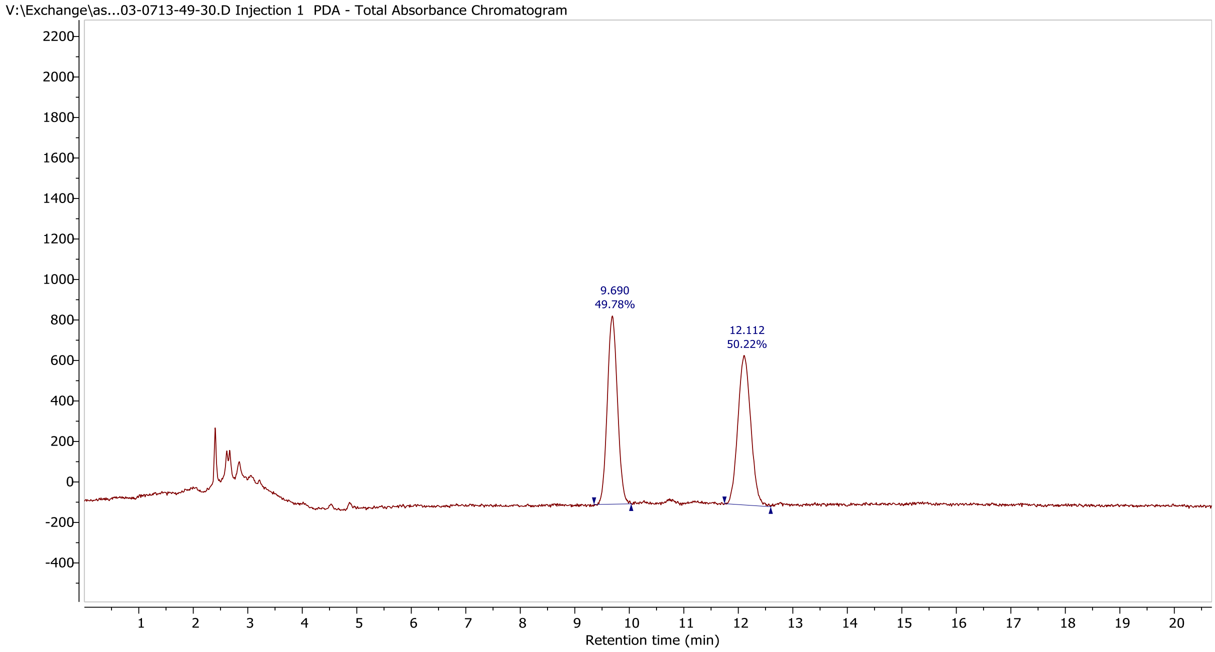

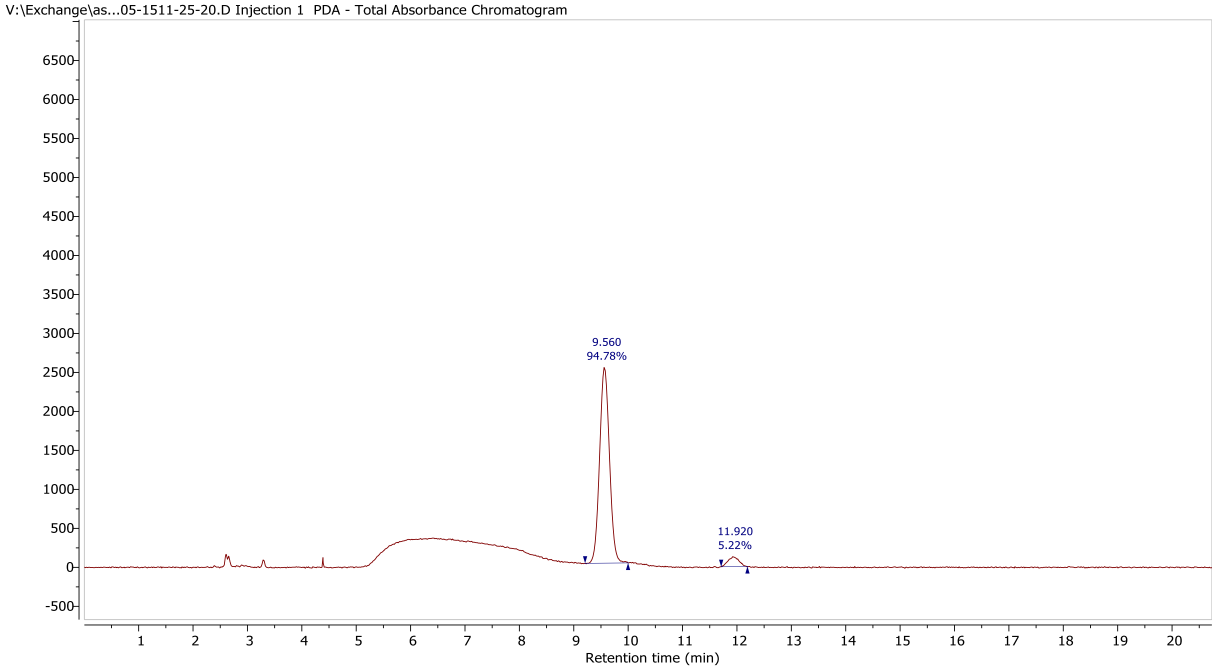

[2g]

top: racemic sample

bottom: enantioenriched sample

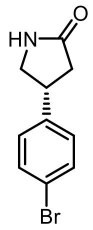

V:\Exchange\as...03-0513-59-07.D Injection 1 PDA - Total Absorbance Chromatogram

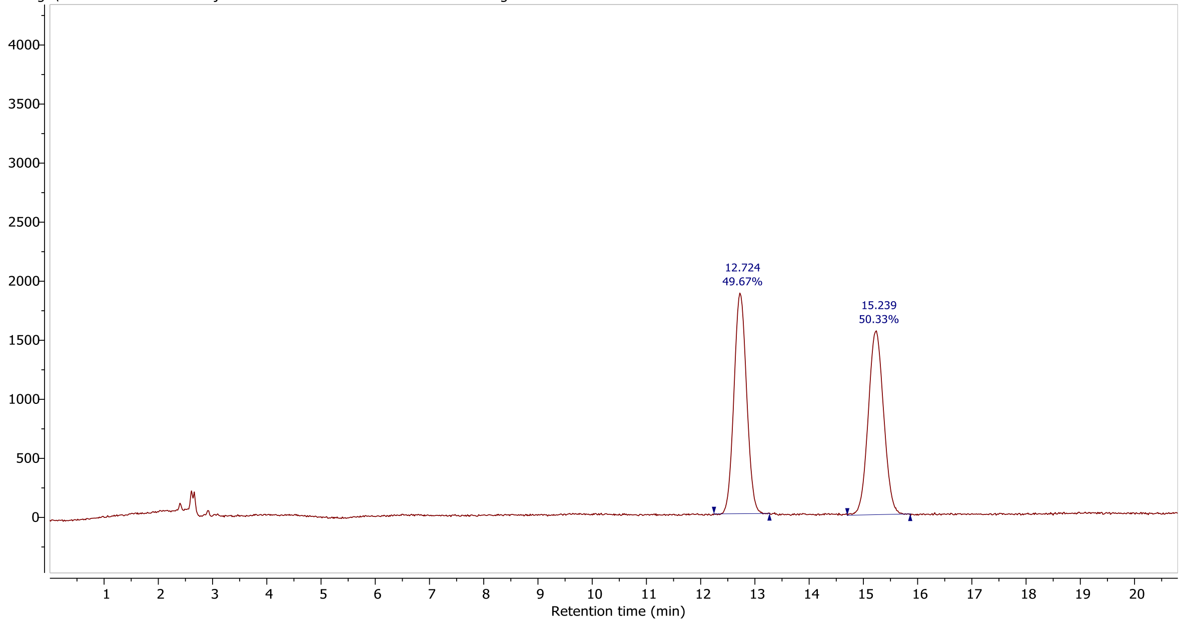

V:\Exchange\as...04-2913-23-00.D Injection 1 DAD1B, Sig=214,4 Ref=360,100 Chromatogram

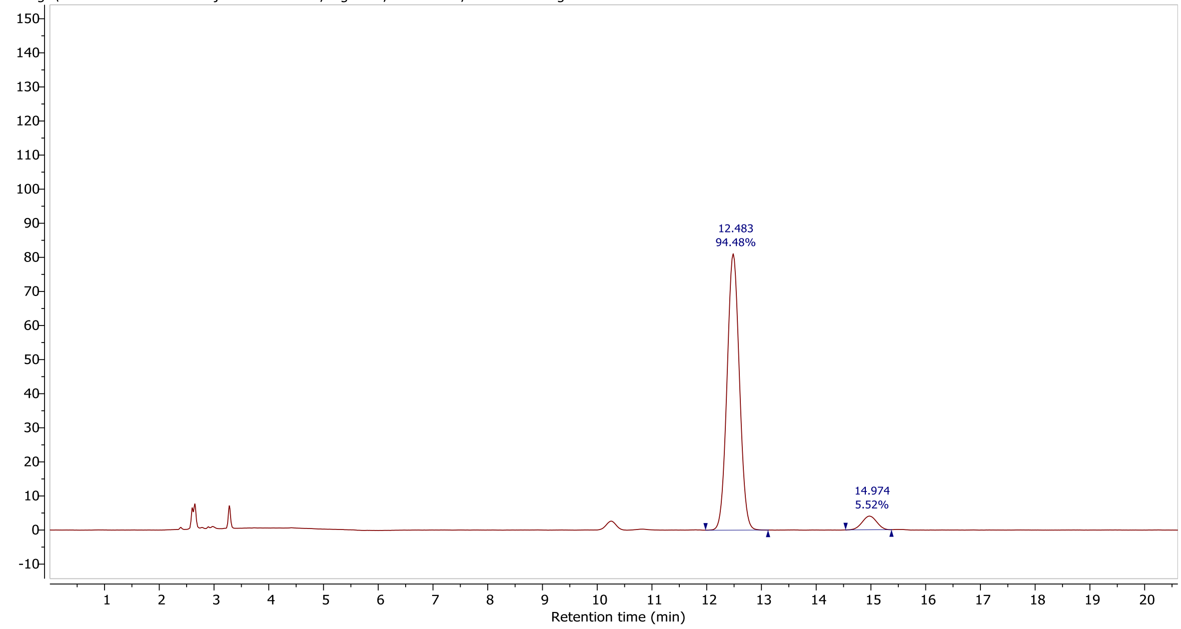

[2h]

top: racemic sample

bottom: enantioenriched sample

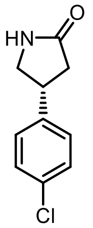

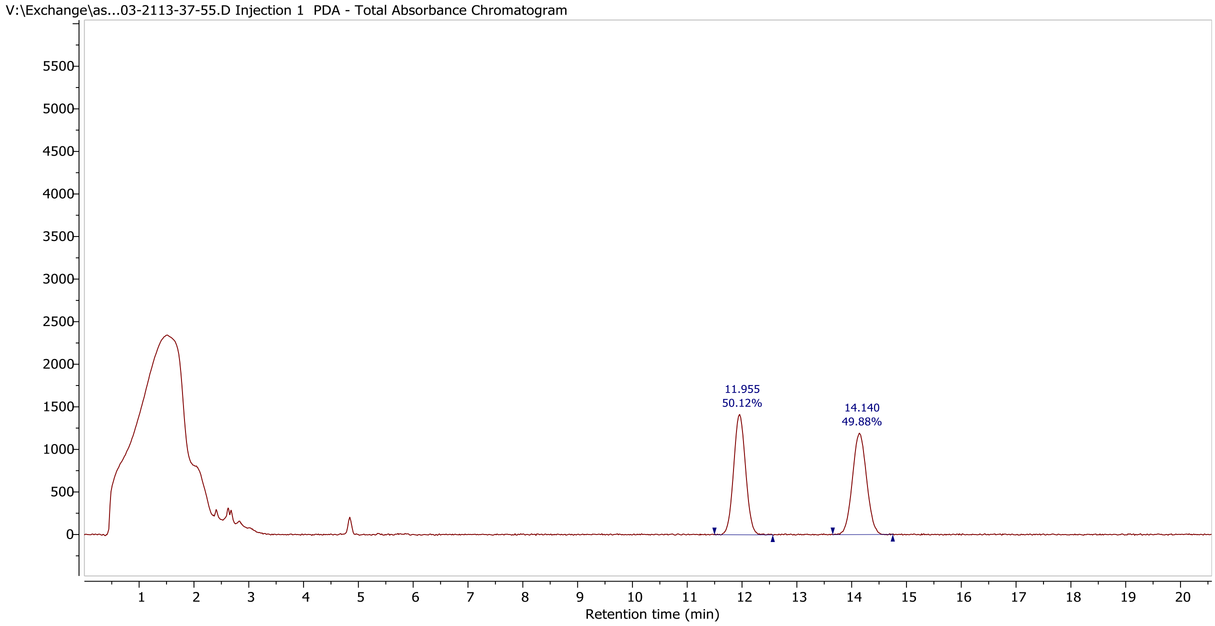

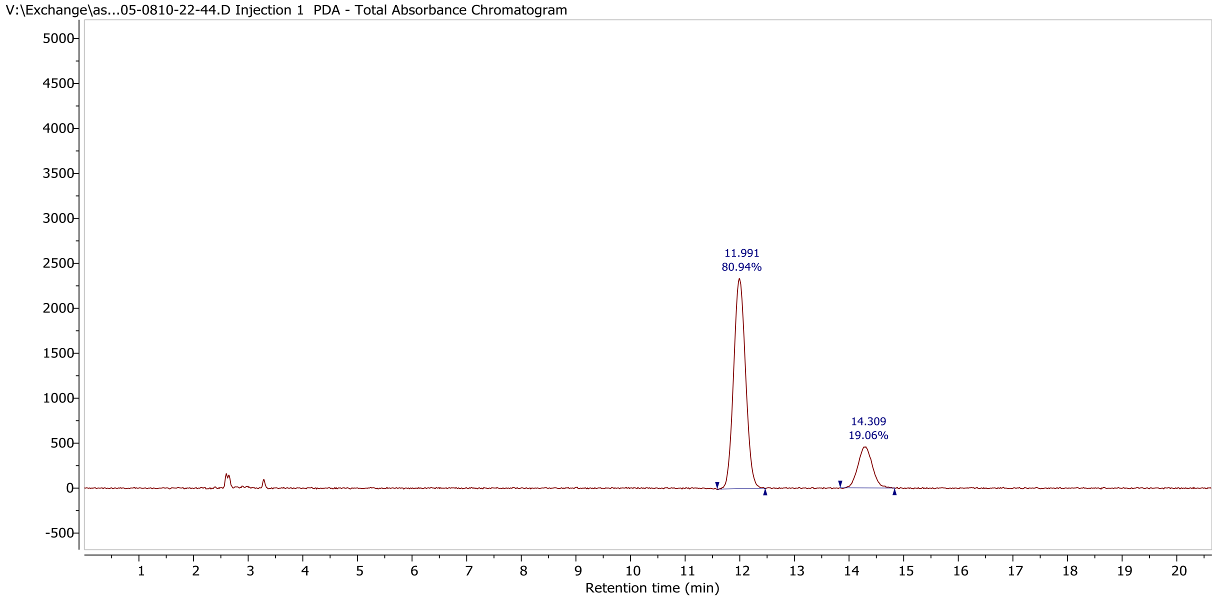

[2i]

top: racemic sample

bottom: enantioenriched sample

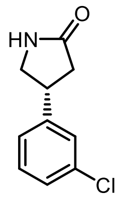

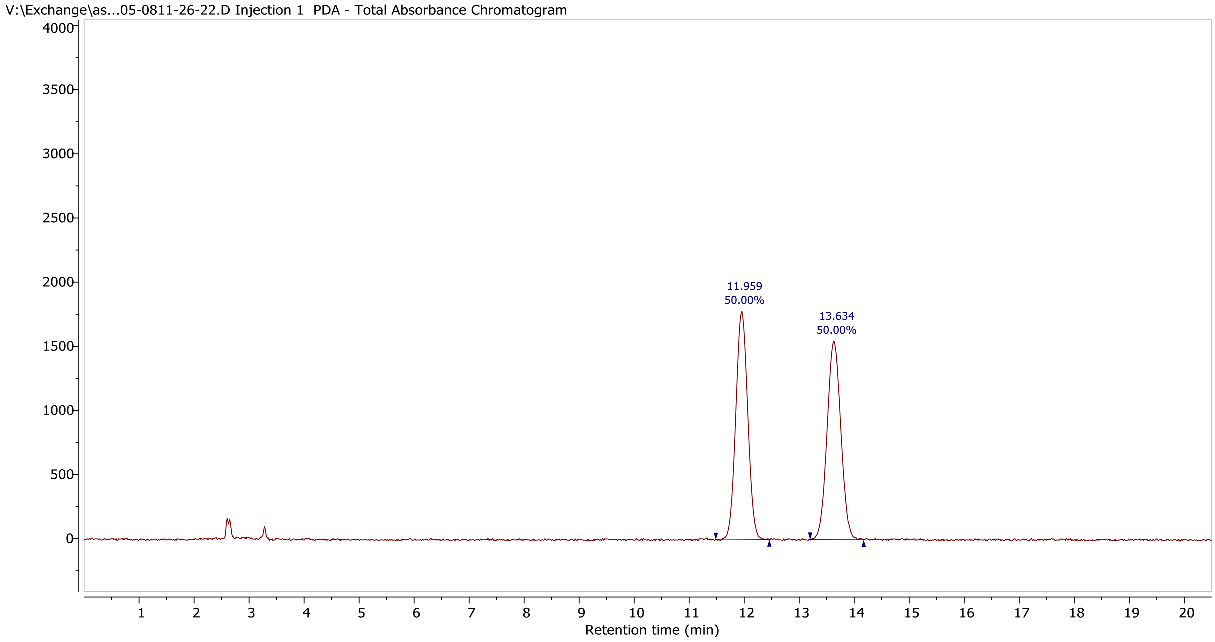

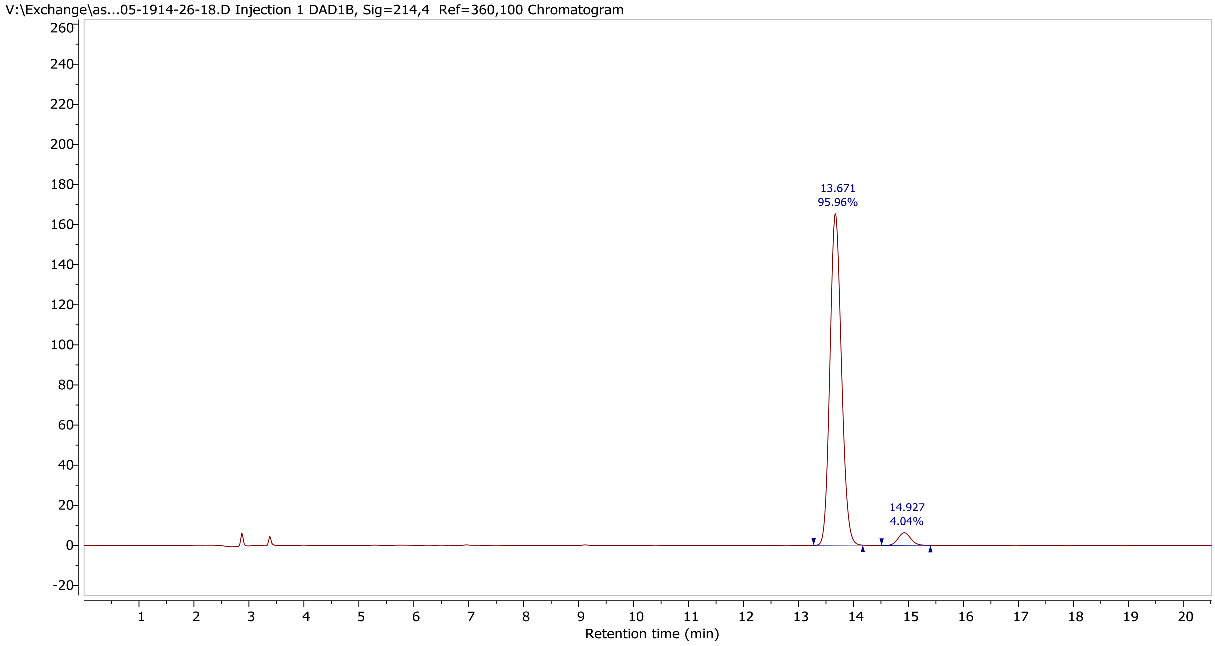

[2j]

top: racemic sample

bottom: enantioenriched sample

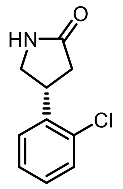

V:\Exchange\as...03-2115-34-08.D Injection 1 PDA - Total Absorbance Chromatogram

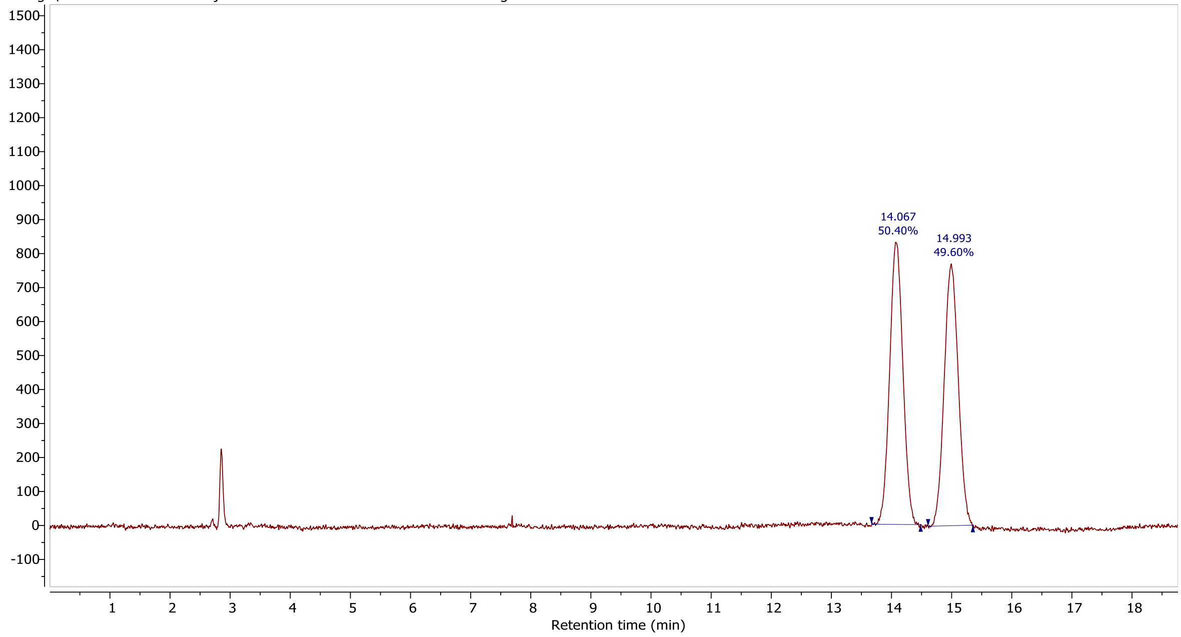

V:\Exchange\as...05-1516-07-15.D Injection 1 DAD1B, Sig=214,4 Ref=360,100 Chromatogram

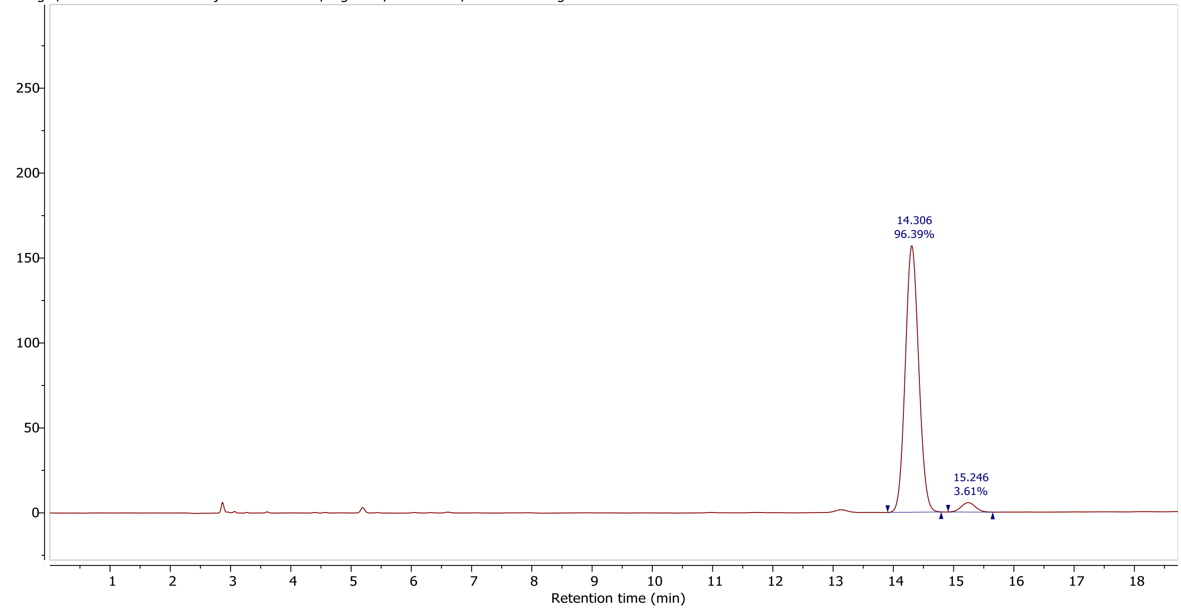

[2k]

top: racemic sample

bottom: enantioenriched sample

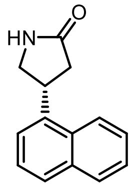

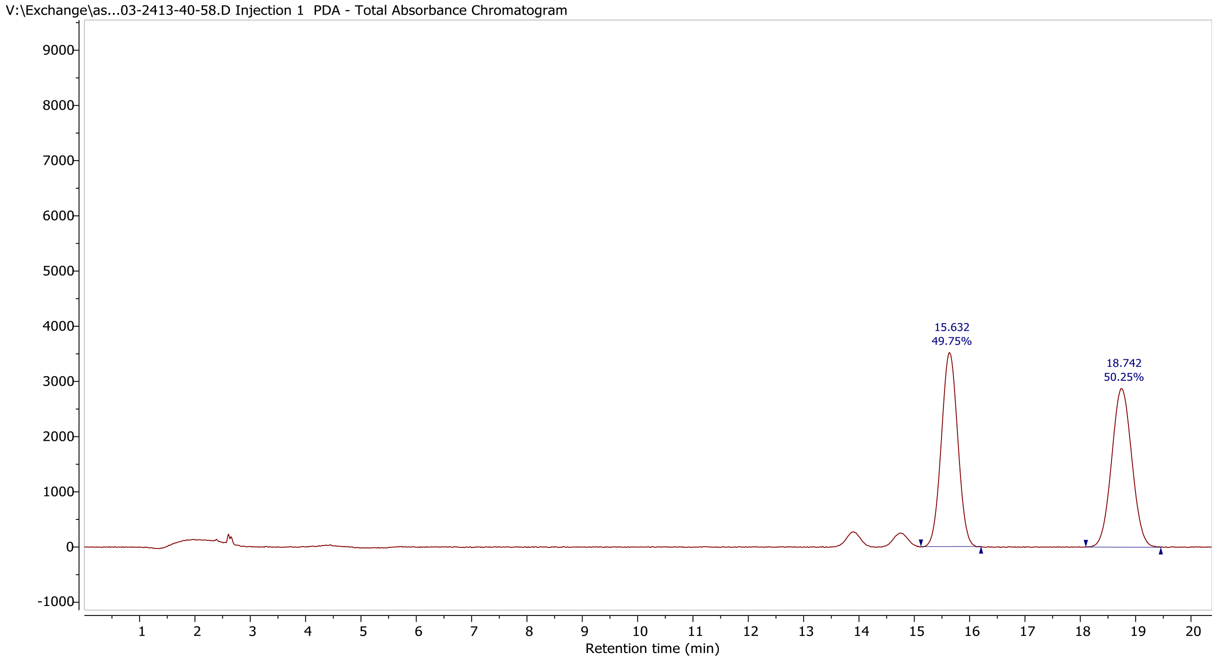

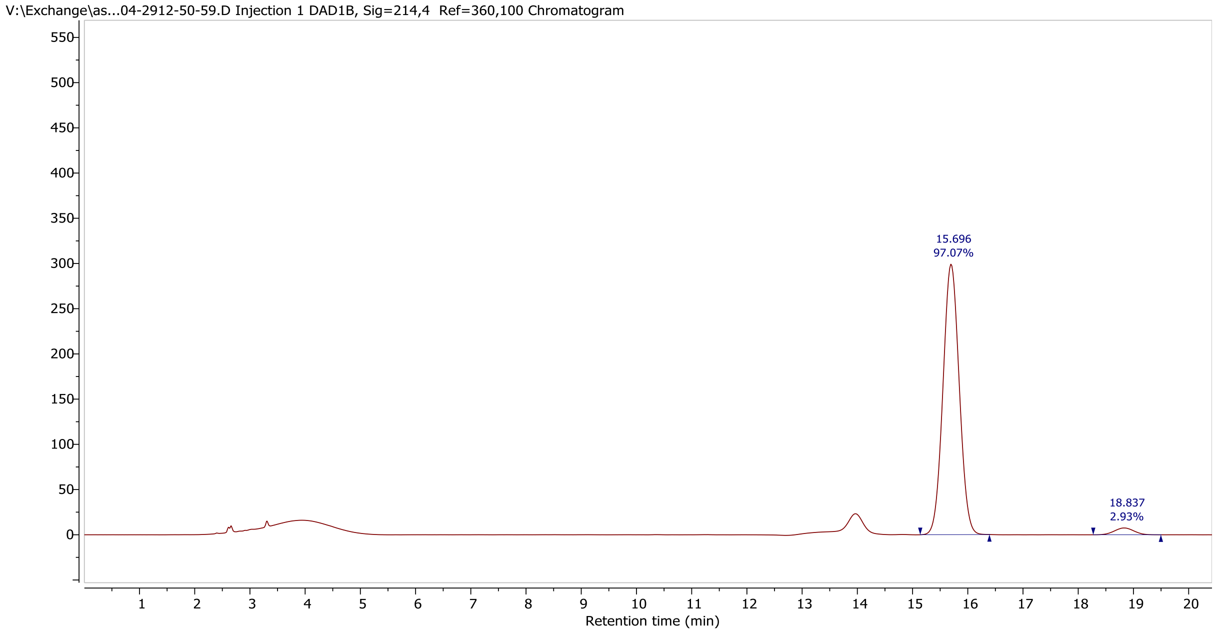

[2l]

top: racemic sample

bottom: enantioenriched sample

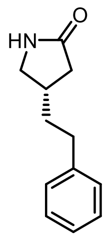

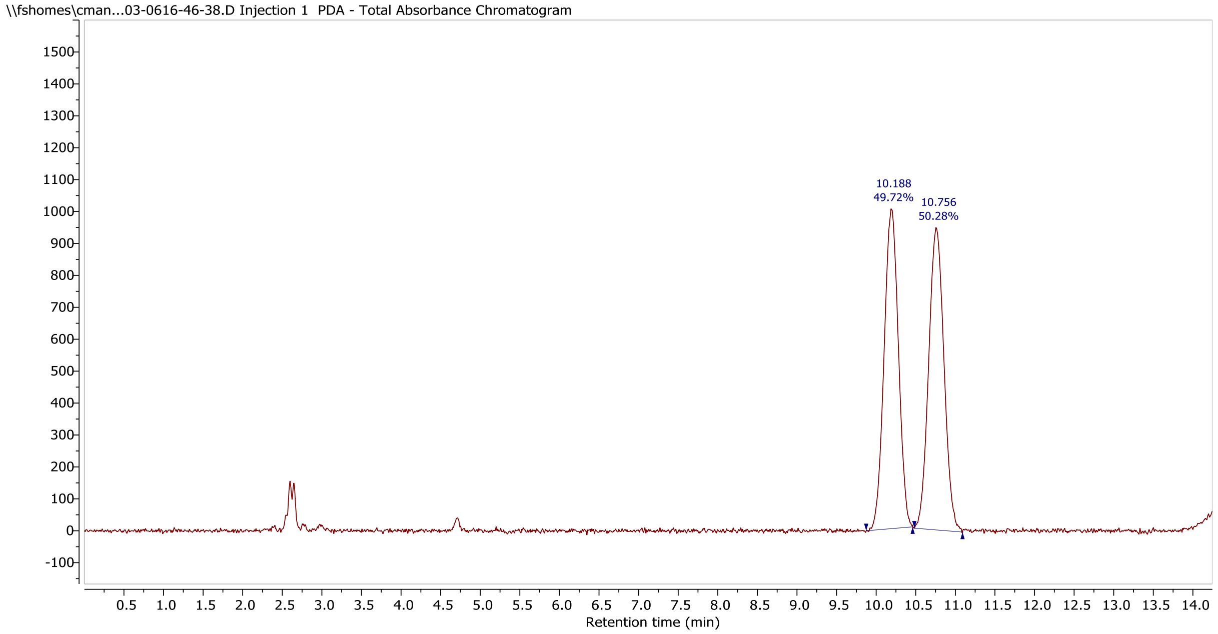

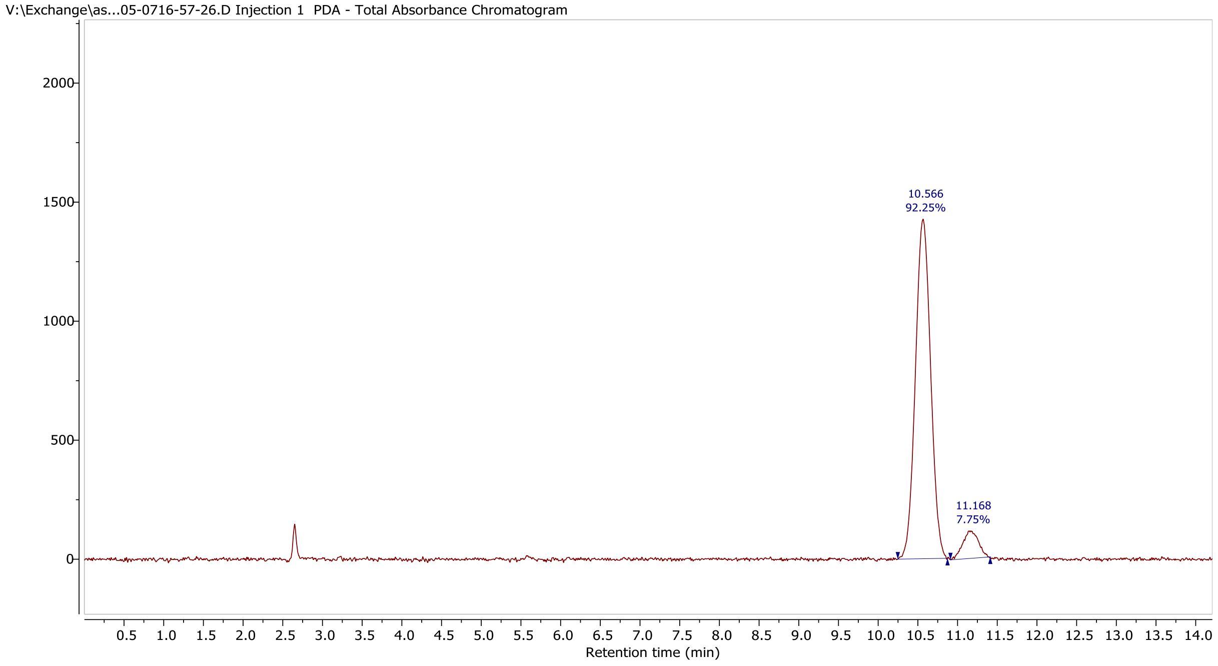

[2m]

top: racemic sample

bottom: enantioenriched sample

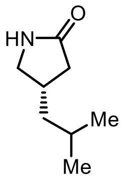

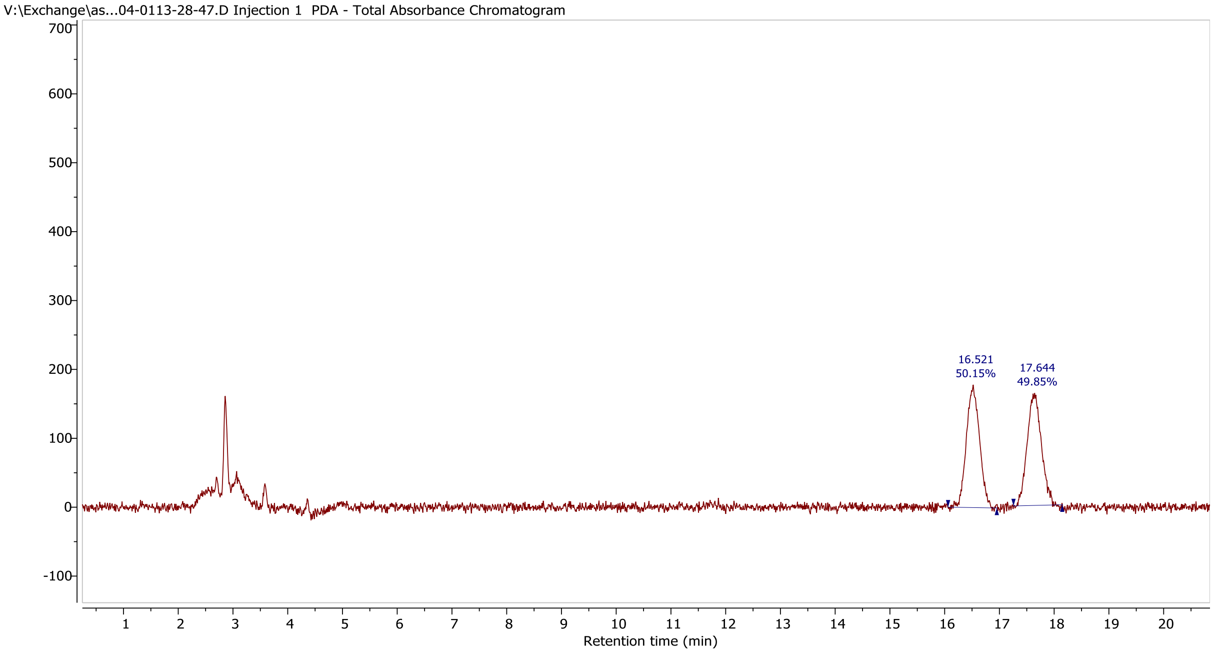

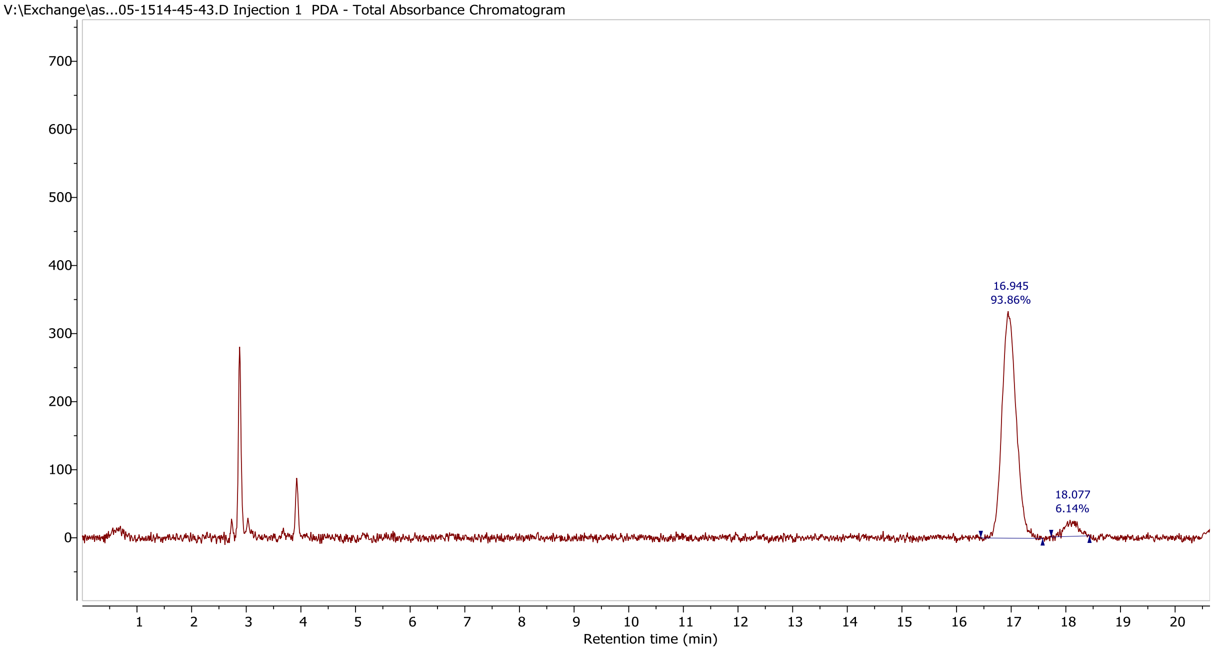

[6n]

top: racemic sample

bottom: enantioenriched sample

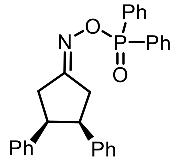

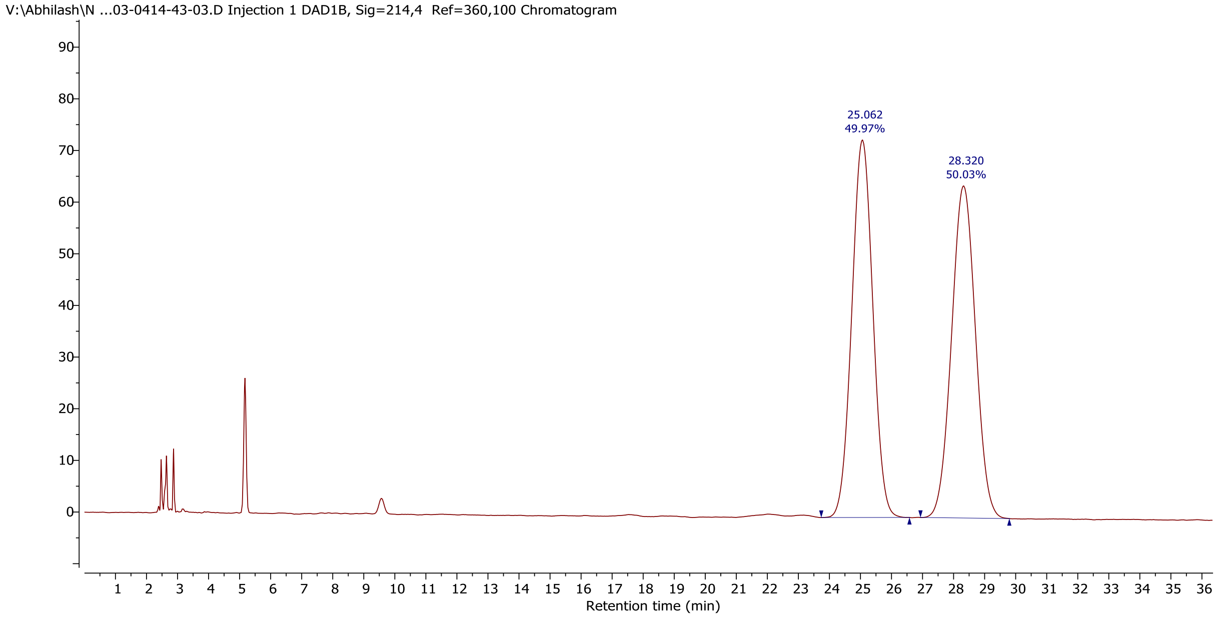

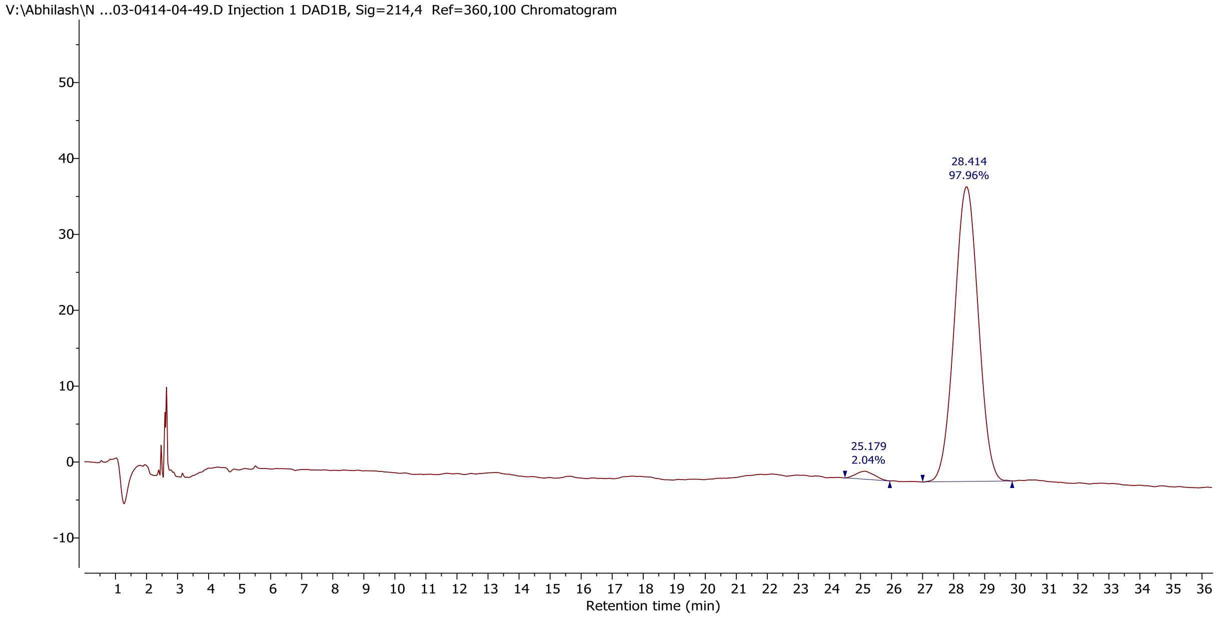

[2n]

top: racemic sample

bottom: enantioenriched sample

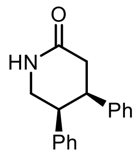

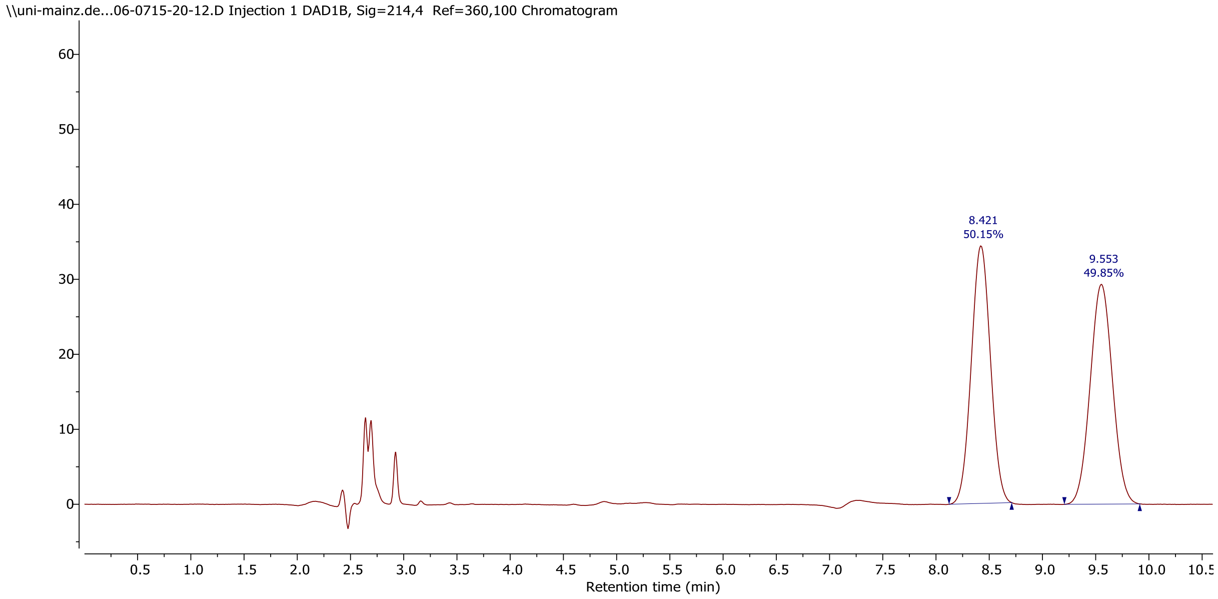

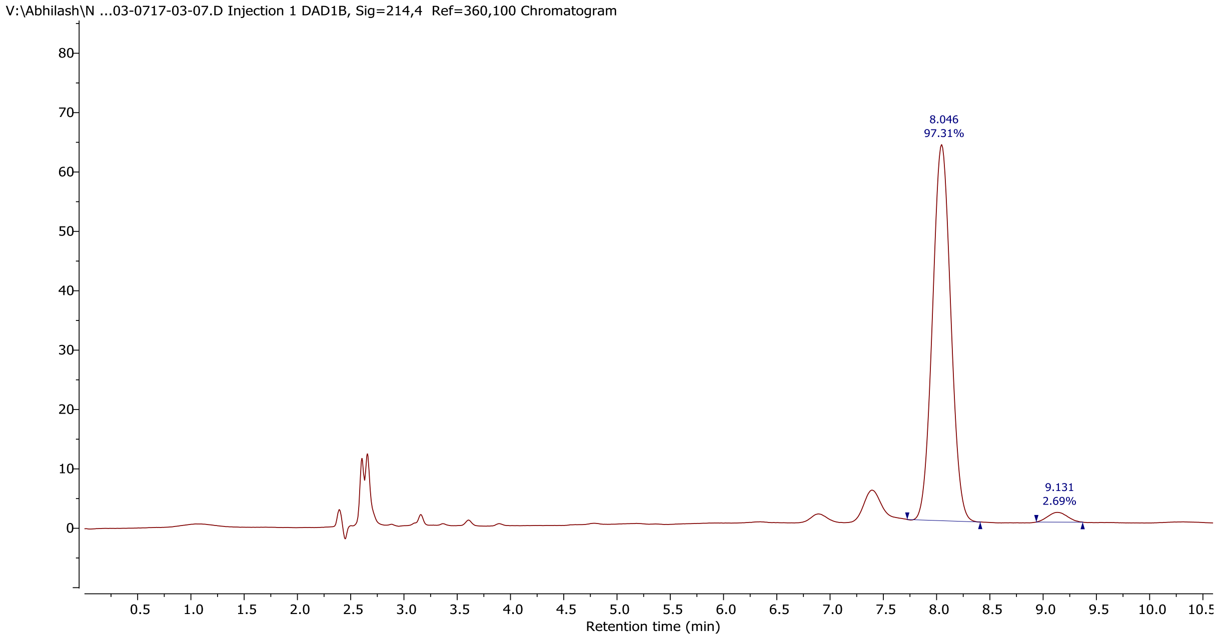

[2o]

top: racemic sample

bottom: enantioenriched sample

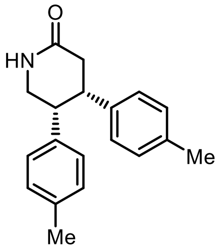

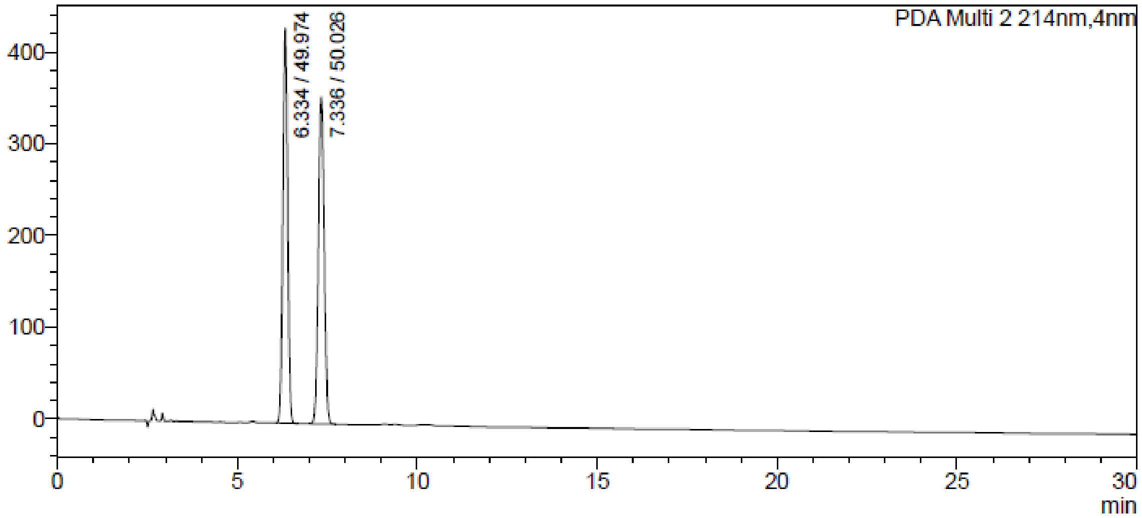

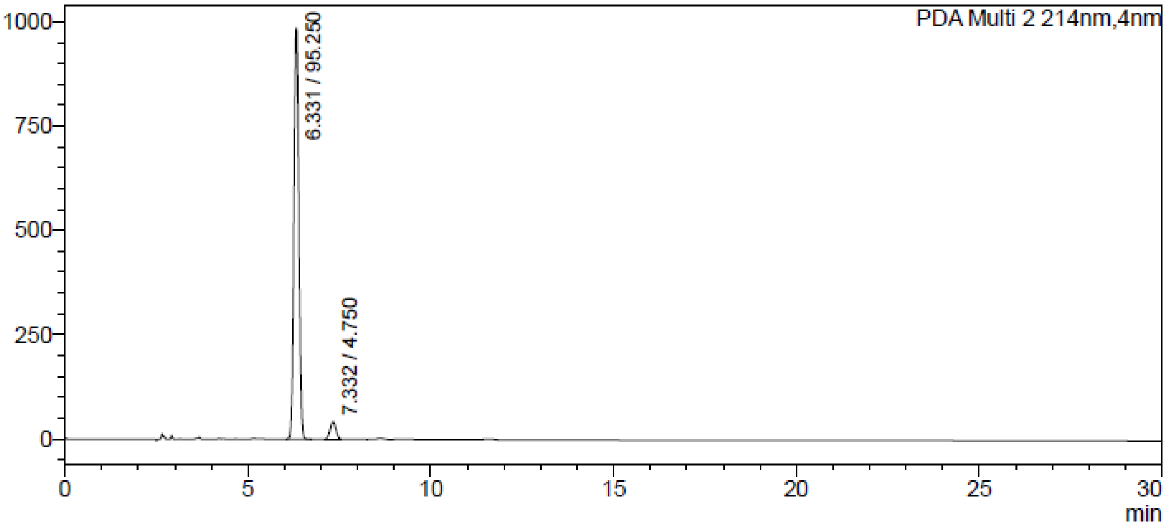

[2p]

top: racemic sample

bottom: enantioenriched sample

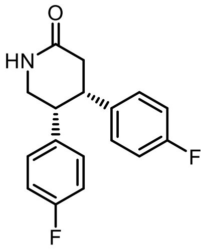

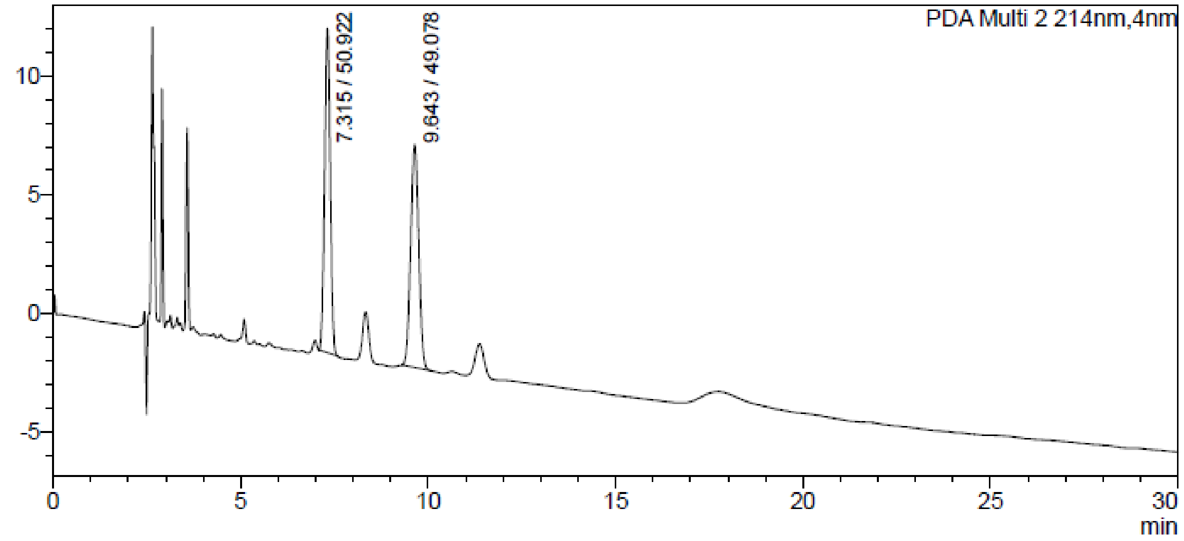

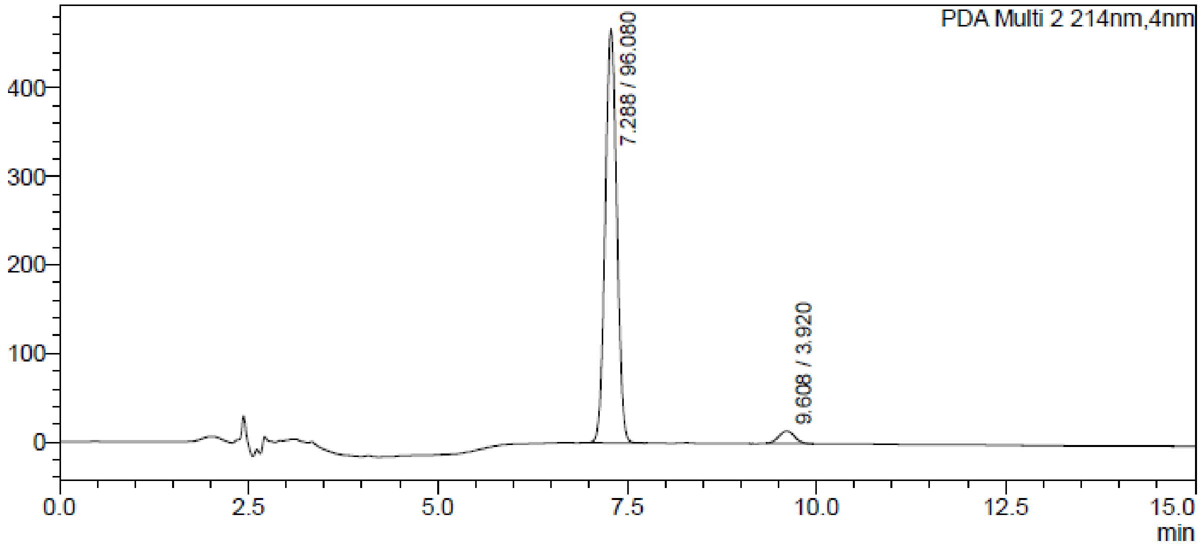

[6q]

top: racemic sample

bottom: enantioenriched sample

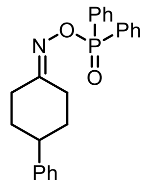

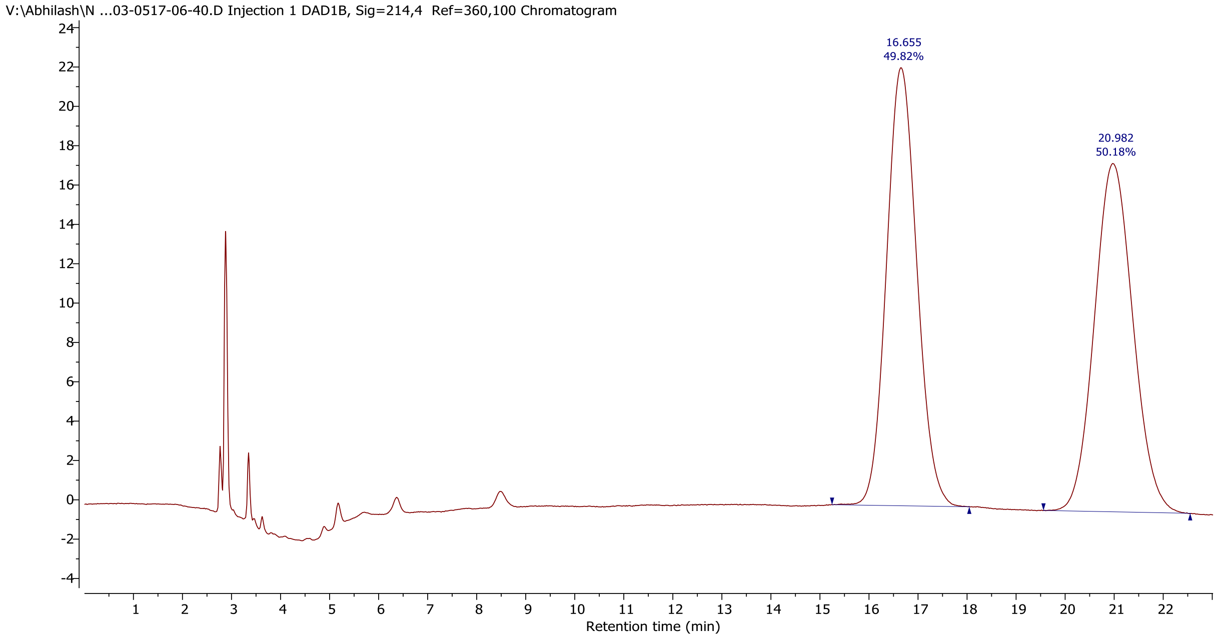

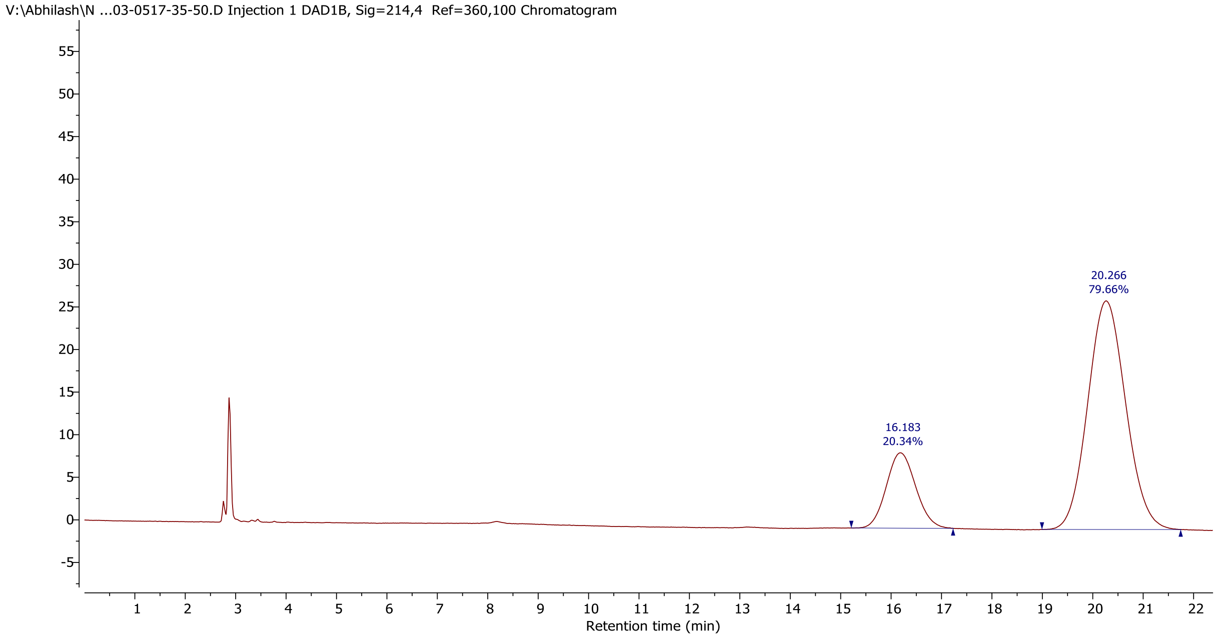

[2q]

top: racemic sample

bottom: enantioenriched sample

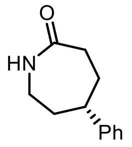

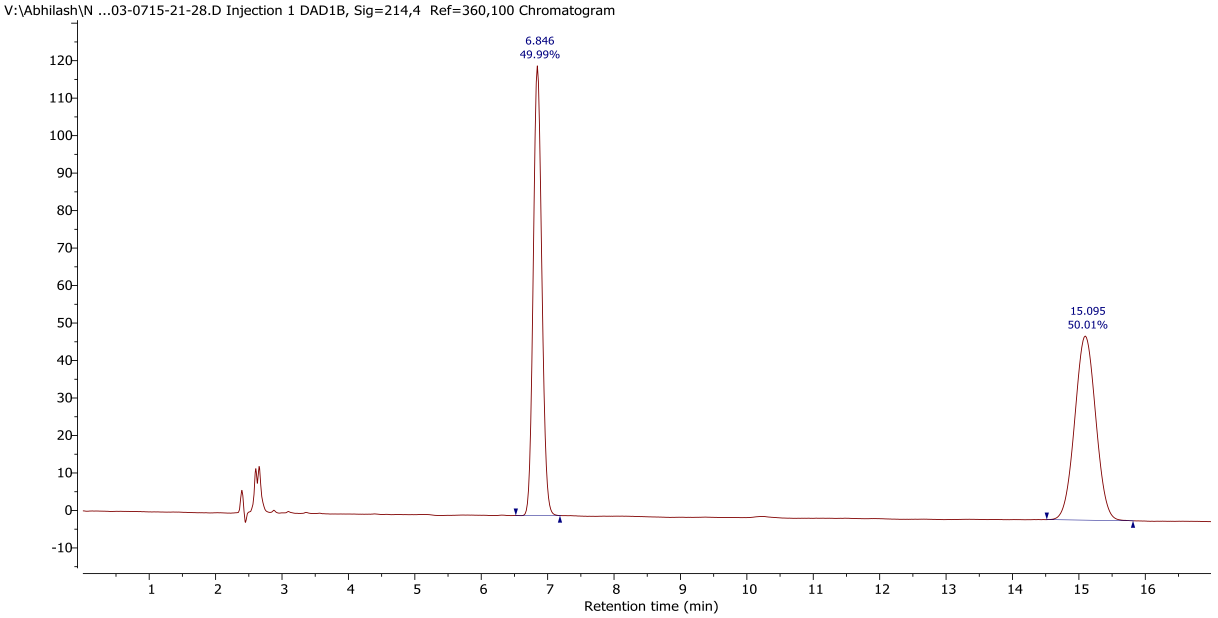

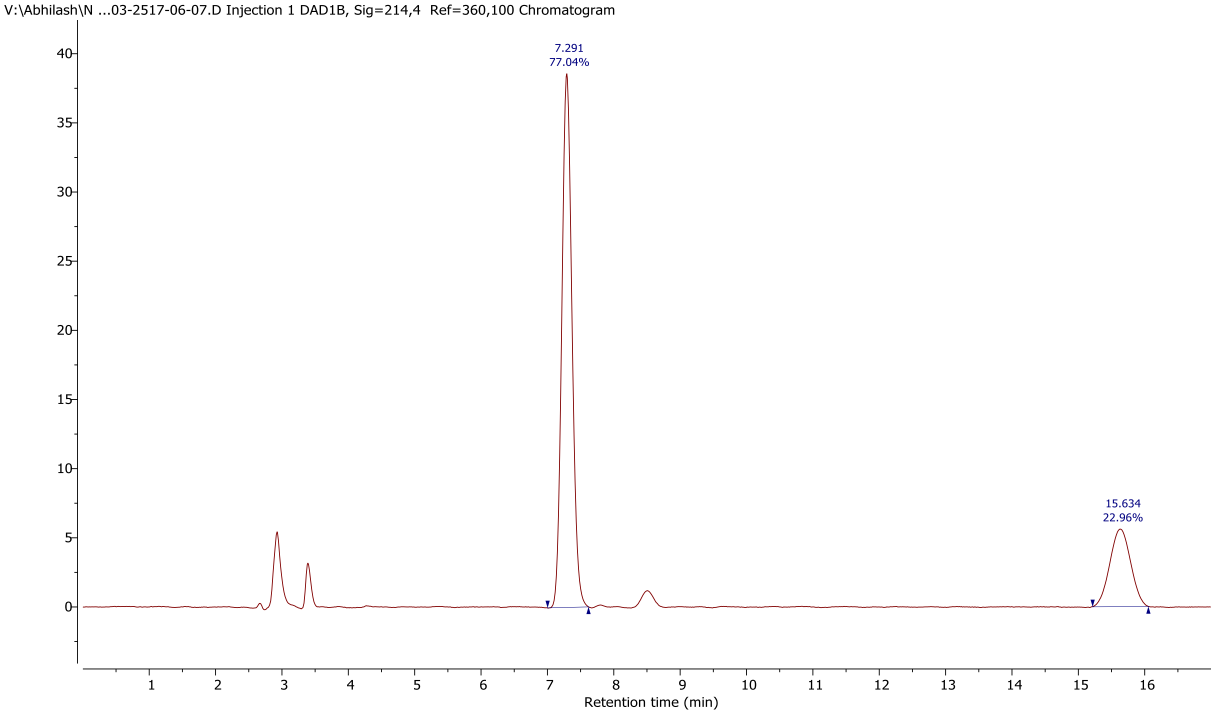

[6r]

top: racemic sample

bottom: enantioenriched sample

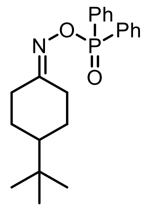

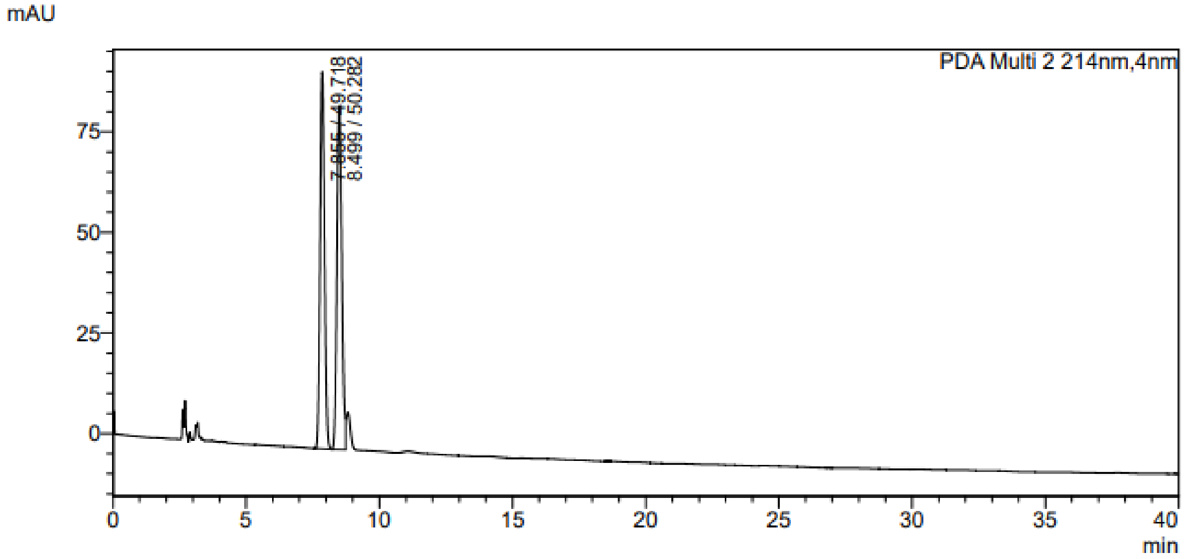

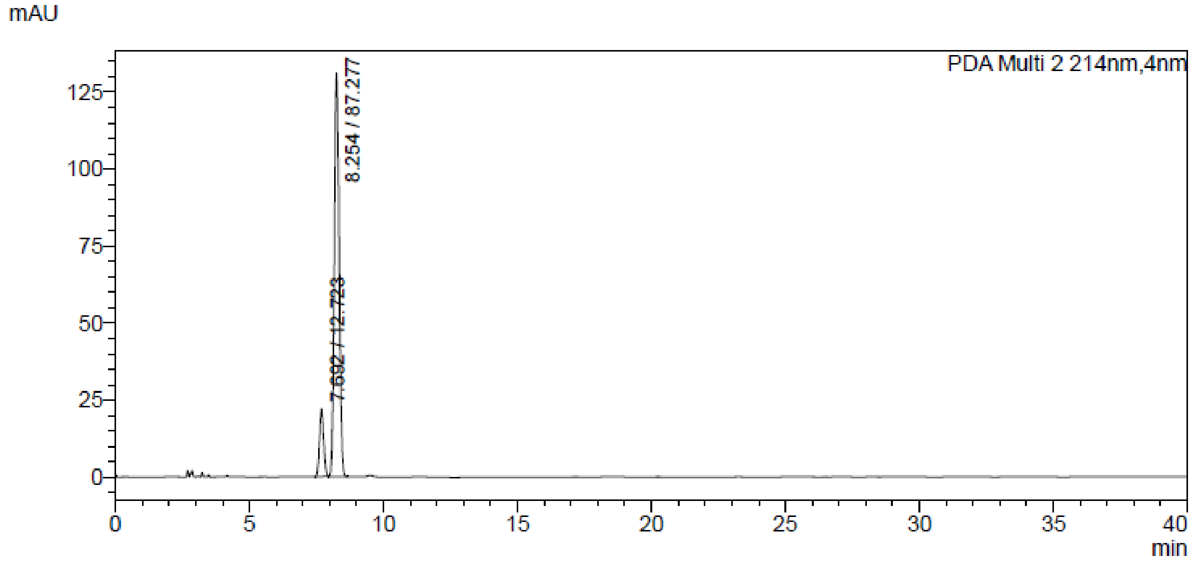

[*N*-Bn-2r]

top: racemic sample

bottom: enantioenriched sample

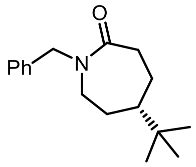

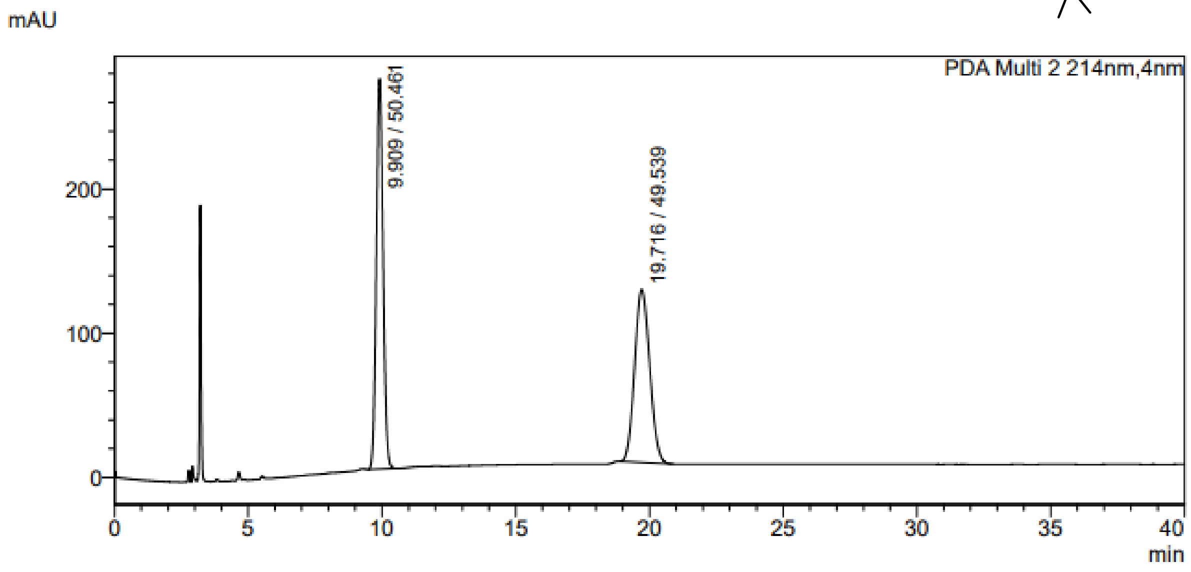

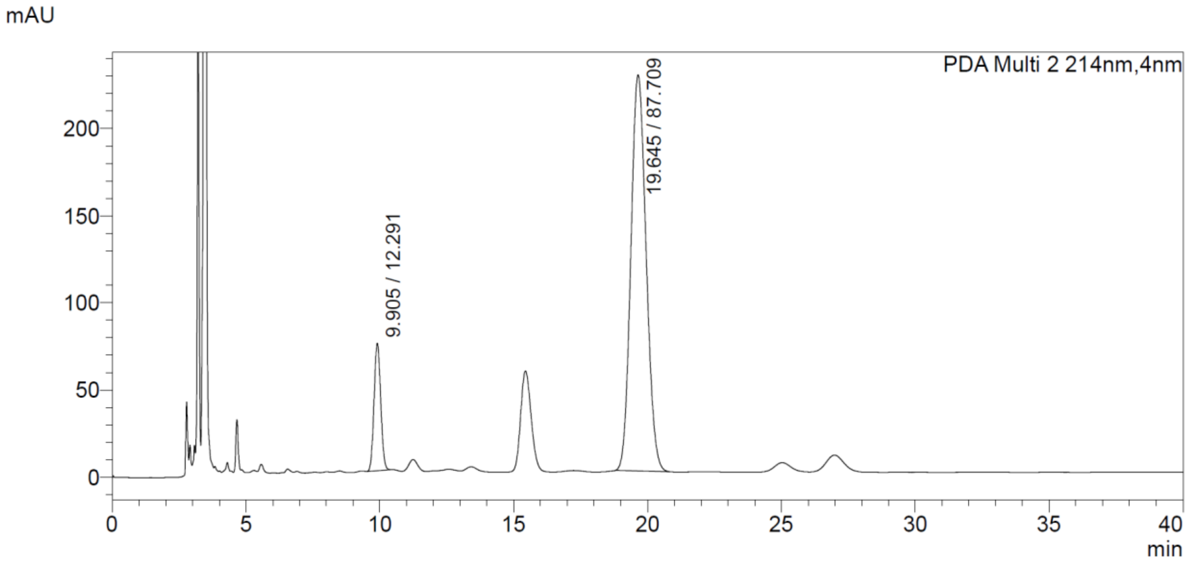

[10]

top: racemic sample

bottom: enantioenriched sample

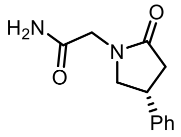

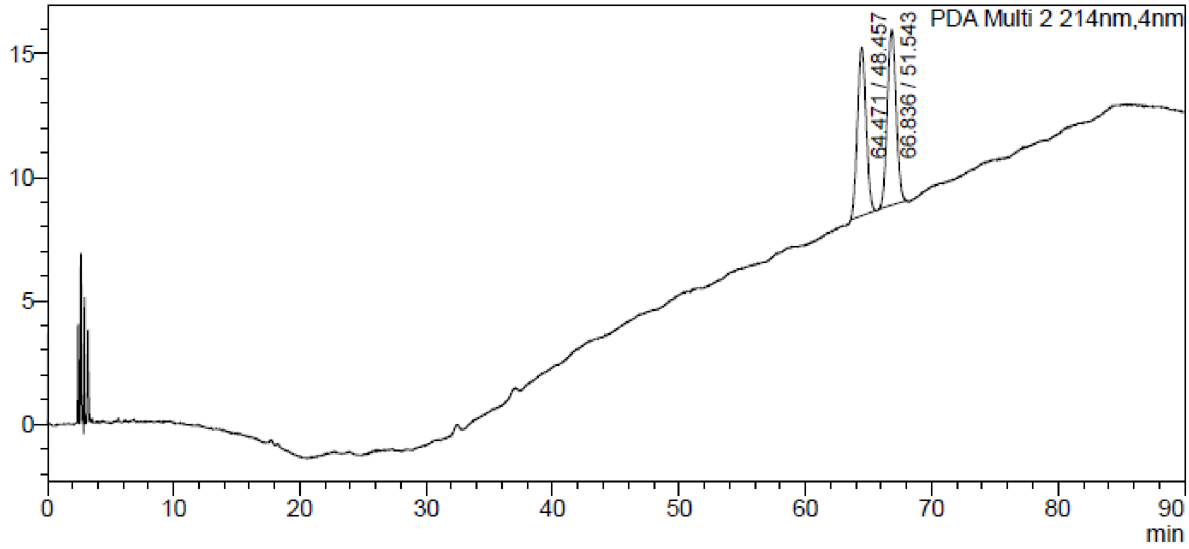

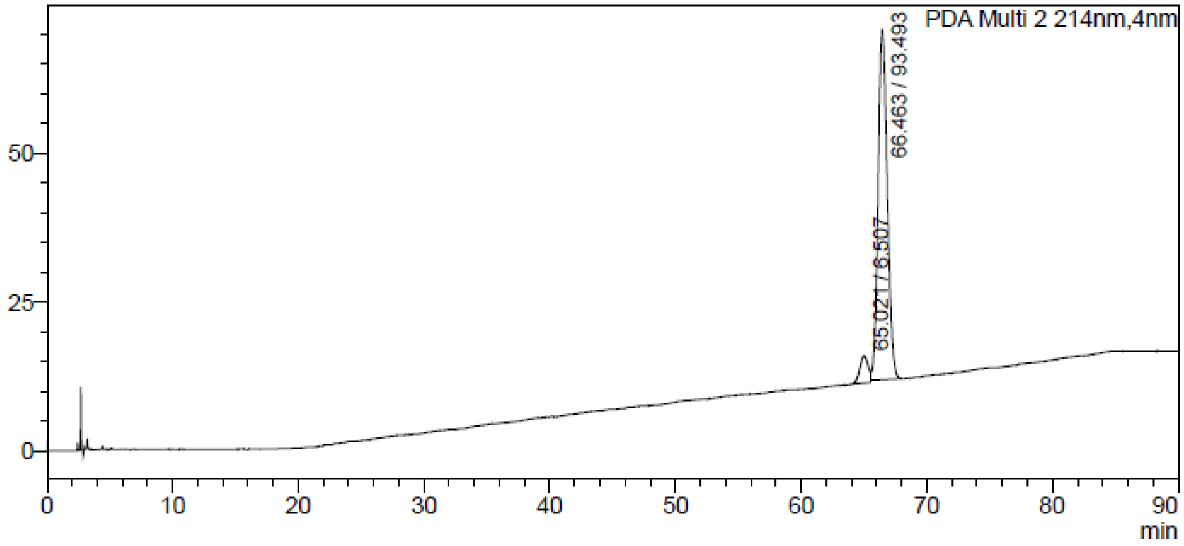

[12]

top: racemic sample

bottom: enantioenriched sample

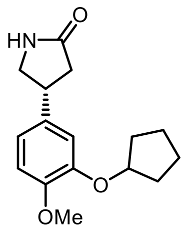

V:\Exchange\as...04-0210-24-29.D Injection 1 PDA - Total Absorbance Chromatogram

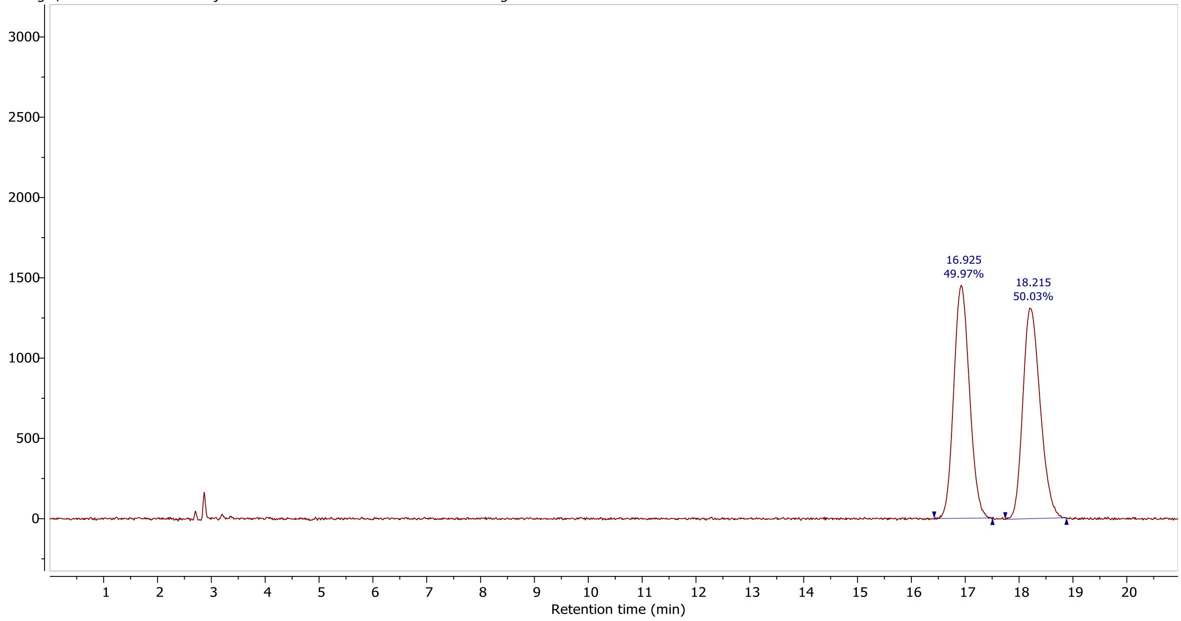

D:\Jasmin HPLC...06-0315-51-35.D Injection 1 PDA - Total Absorbance Chromatogram

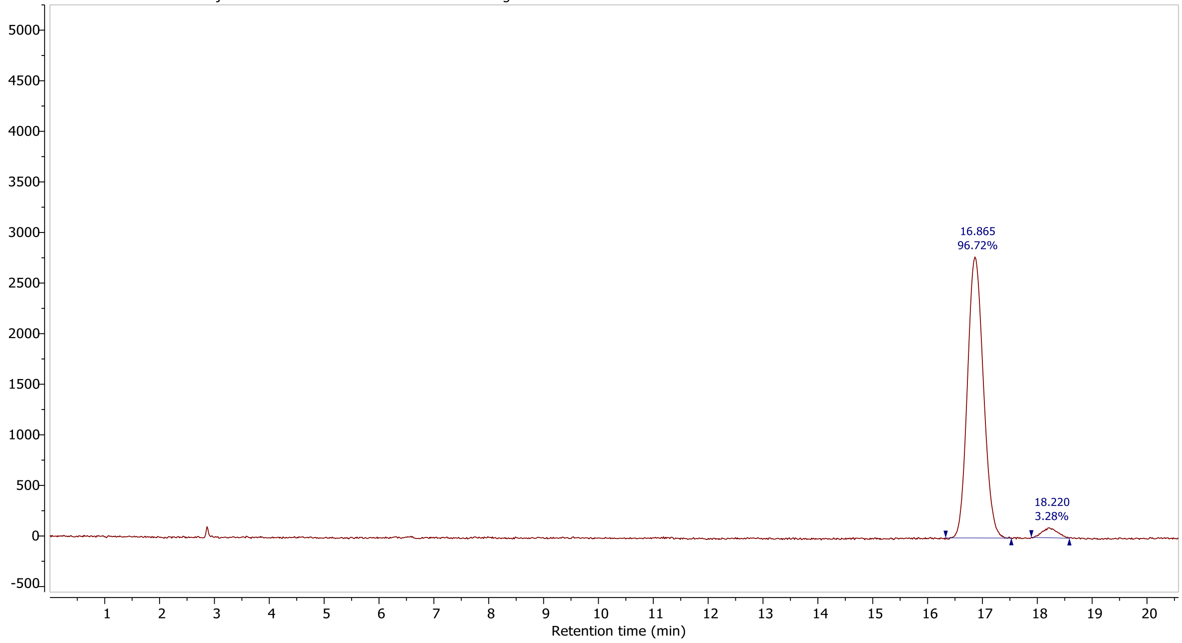

Supplement: SC-017-D5SC08417B-s001 [file SC-017-D5SC08417B-s001.pdf]
